# Herbal Textual Research, Phytochemistry, Pharmacology and Toxicity of Atractylodis Rhizoma: A Comprehensive Review

**DOI:** 10.3390/molecules31061015

**Published:** 2026-03-18

**Authors:** Jin Sun, Juhui Qiao, Jian Tang, Nuo Cheng, Miaomiao Gao, Jingrong Yang, Baixin Kou

**Affiliations:** 1 School of Chinese Medicine, Bozhou University, Bozhou 236800, China; sunjin0509@163.com (J.S.);; 2 School of Medicine, Changchun Sci-Tech University, Changchun 130600, China; 3 School of Pharmaceutical Sciences, Changchun University of Chinese Medicine, Changchun 130117, China

**Keywords:** *Atractylodes lancea*, *Atractylodes chinensis*, ethnopharmacology, atractylodin

## Abstract

This review examines the historical development, ethnopharmacology, traditional applications, phytochemistry, and pharmacological attributes of Atractylodis Rhizoma (AR). Data were collected from a range of electronic databases, academic libraries, and classical literature. In China, AR is highly valued for its medicinal properties. Research has identified 327 compounds, including sesquiterpenes, triterpenes, flavonoids, and phenolics, which contribute to its diverse pharmacological activities, such as antimicrobial, anti-inflammatory, antioxidant, hepatoprotective, and neuroprotective effects. AR is particularly effective in treating modern gastrointestinal disorders and influenza. As a traditional herb with a rich historical background, AR exhibits significant therapeutic potential. This review aims to correlate its active components with its primary therapeutic effects and highlight existing research gaps. Current studies primarily focus on extraction methods and pharmacodynamics. Future research should employ multi-omics and molecular biology techniques to further elucidate active components and their targets, while also addressing the challenge of low bioavailability.

## 1. Introduction

**Figure 1:**
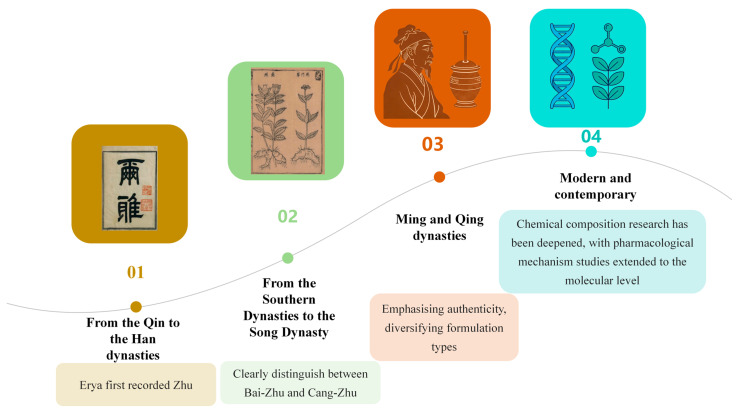
The Historical Application of Atractylodis Rhizoma. Note: Different colors represent different periods: yellow represents the period when the term first originated; green represents the period when species began to be distinguished; orange represents the period of widespread use; and blue represents the period of modern research.

Atractylodis Rhizoma (AR) is a pivotal herb in Traditional Chinese Medicine [1], esteemed for its effectiveness in alleviating dampness, fortifying the spleen, dispelling wind and cold, and enhancing vision (Figure 1). Its historical utilization can be traced back to the Erya (1st–2nd century AD), which documented its properties and nomenclature [2]. Shennong’s Herbal Classic classified it as a “superior” herb for the treatment of wind-cold-damp arthralgia, convulsions, and jaundice [3]. However, herbal texts from the Han and Wei dynasties and earlier periods did not differentiate between AR (Cang-Zhu) and Atractylodis Macrocephalae Rhizoma (Bai-Zhu) [4], referring to both simply as Zhu. It was only after the Ben Cao Jing Ji Zhu that these terms began to be used distinctively. Throughout history, numerous classic formulas have employed Cang-Zhu and Bai-Zhu, such as Wan Dai Decoction [5], Ling Gui Zhu Gan Decoction [6], Ping Wei Powder [7], and Wei Ling Decoction [8]. To prevent confusion and misuse, conducting thorough herbal verification is particularly crucial. The *Atractylodis* genus primarily includes *A*. *carlinoides* (Hand. -Mazz.) Kitam., *A. coreana* (Nakai) Kitam., *A. japonica* Koidz. ex Kitam., *A. lancea* (Thunb.) DC., *A. chinensis* (DC.) Koidz., and *A. macrocephala* Koidz., primarily distributed throughout eastern Asia. *A. lancea* (Thunb.) DC. (Mao-Cang-Zhu) or *A. chinensis* (DC.) Koidz (Bei-Cang-Zhu) are the two sources of AR, distributed in Shandong, Jiangsu, Zhejiang, Hubei, Sichuan, and other regions of China [9].

Significant progress has been made in the isolation, identification, and investigation of potential pharmacological activities of AR compounds [10,11]. While extensive research exists on *A. lancea*, reports on *A. chinensis* remain scarce. Given the expanding demand for AR, it is gradually gaining greater attention. A review article published in 2021 systematically summarized the botanical characteristics, traditional uses, phytochemical composition, pharmacological effects, and quality control standards of AR [12]. However, comprehensive research on the structural composition of compounds in AR and their potential mechanisms of action remains insufficient to date.

Against this backdrop, a comprehensive review and analysis of AR is urgently needed. This study begins with the traditional usage of AR, integrates findings from modern technological discoveries, and comprehensively examines the connections and distinctions between traditional applications and contemporary uses. It aims to provide a reference and basis for AR research, offering insights for innovative studies and comprehensive collation of similar Chinese herbal medicines.

## 2. Materials and Methods

### 2.1. Search Strategy

A comprehensive online literature search was conducted across multiple databases, including ScienceDirect, Google Scholar, PubMed, Web of Science, CNKI, WFO, MPNS, the Changchun University of Chinese Medicine Library collections, and SciFinder, covering the period from 1996 to 2026. For the methodology outlined in reference [13], the search employed the keywords “Atractylodis Rhizoma” in conjunction with “phytochemistry”, “pharmacology”, or “toxicity”. The references of all retrieved articles were meticulously reviewed to ensure the inclusion of pertinent literature. Uniform selection criteria were consistently applied across all databases, and duplicate studies were systematically eliminated through a two-step process involving automated detection via Zotero 7, followed by manual cross-verification. Additionally, traditional and historical applications were corroborated through a systematic analysis of classical Chinese medical texts. The search strategy comprehensively covered medicinal and dietary records using the nomenclature “Cang Zhu,” “Zhu,” “Ji,” “Shan Ji,” “Tian Su,” and “Yang Bao.” Relevant prescriptions were compiled after the removal of duplicates through cross-database verification.

### 2.2. Selection Criteria

The inclusion criteria for this study are delineated as follows: the primary focus is on AR, including its extracts and constituent compounds; the investigation encompasses diseases and physiological processes influenced by AR; the study design must be clearly articulated, with results that explore pertinent mechanisms; the research incorporates the most recent findings on the clinical applications and formulations of AR; only literature published within the last 30 years is considered, except in cases of significant historical relevance; and the research must explicitly detail molecular mechanisms or signaling pathways, along with their impact on bioavailability or efficacy.

### 2.3. Analytical Methods and Software

The graphical illustrations in this paper were created using multiple software tools, including the Home for Researchers (https://professional.home-for-researchers.com/) and Microsoft PowerPoint 2024 (https://www.microsoft.com/). Visualizations presenting the investigation of classical herbal formulas and their modern pharmaceutical applications were generated with Origin 2024 (https://www.originlab.com/). All chemical structures depicted in this study were drawn using ChemDraw 22.0.0.

## 3. Herbal Textual Research

### 3.1. Origin

To elucidate the origin of AR, the morphological records from ancient Chinese herbal texts have been systematically compiled and analyzed, as presented in Table 1. This compilation reveals a progressive refinement in the understanding of this herb, transitioning from an undifferentiated archetype to a pharmacognostically distinct entity. The earliest references, such as the Er Ya from the Warring States period, establish the foundational concept of “Zhu”, associating it with thistle-like plants characterized by elliptical leaves, spiny-toothed margins, and capitate inflorescences—morphological features that precisely align with the Asteraceae family to which *Atractylodes* genus. This initial ambiguity between Cangzhu and Baizhu reflects a holistic view of the genus before species-level differentiation. The critical shift occurred during the Northern and Southern Dynasties, as documented in the Ben Cao Jing Ji Zhu, where the name “Cangzhu” first appeared alongside explicit descriptions of “slender, branchless leaves” and roots that are “small, bitter, and rich in sap”. The emphasis on “rich in sap” directly corresponds to the high volatile oil content characteristic of *A. lancea*, distinguishing it organoleptically from the milder Baizhu. This text also anchors the herb geographically to Jiangsu Province, particularly the Maoshan region, establishing a provenance that would persist for centuries as the benchmark for superior quality. Subsequent Ming and Qing dynasties texts refined this knowledge with remarkable precision. The Ben Cao Yuan Shi from the Ming dynasty provides an exemplary pharmacognostic profile, extolling “Maoshan Cangzhu” for its “black bark and yellow flesh dotted with red spots”—a direct observation of oil cavities that remains a key identification marker today. It further distinguishes inferior variants by their larger size and excessively pungent taste, demonstrating a sophisticated understanding of intra-specific variation and quality gradation. The Qing dynasty Ben Cao Chong Yuan adds complementary morphological details, such as leaves near the root dividing into three or five forks and purple stems, traits that align closely with *A. chinensis*, indicating that while *A. lancea* from Maoshan was considered supreme, related species were also recognized and utilized under the same nomenclature. Throughout this historical trajectory, the consistent recording of production areas—Jiangsu, Henan, Shaanxi, Zhejiang, Anhui, and Hubei—underscores an early awareness of geo-herbalism, with the persistent emphasis on Jiangsu corroborating modern botanical knowledge that *A. lancea* thrives in the specific pedological conditions of this region. Collectively, the ancient texts demonstrate a cumulative empirical process: the core thistle-like morphology was established in the Er Ya, the pungent and oily nature characteristic of *A. lancea* was delineated by the Northern and Southern Dynasties, and the final refinement of quality standards based on organoleptic and physical traits—such as the presence of “oil spots” and “frosting”—was achieved by the Ming and Qing scholars. This 2000-year continuum of observation and documentation provides robust philological and botanical evidence that the primary origin of AR is *A. lancea*, with *A. chinensis* serving as a geographically and morphologically distinct variant, thereby affirming the foundational accuracy of Chinese Pharmacognosy.

All prescriptions documented in the Han Dynasty medical text *Wu Shi Er Bing Fang* employ Zhu. Shennong’s Herbal Classic states: “Zhu, bitter and warm in nature, treats wind-cold-dampness paralysis with deadened muscles, convulsions, jaundice, stops sweating, clears heat, and aids digestion.” Its effectiveness in dispelling wind and eliminating dampness parallels that of modern Cangzhu. Ming Yi Bie Lu notes: “It treats severe wind affecting the body and face, and wind-induced dizziness.” This description of efficacy also corresponds with modern Cangzhu. The aforementioned accounts indicate that medical texts from the Han and Wei dynasties, as well as earlier sources, exclusively documented Zhu. During the Northern and Southern Dynasties, Ben Cao Jing Ji Zhu recorded: “Baizhu has large, hairy leaves that branch out. Its root is sweet with little resin and can be used in pills and powders. Cangzhu has fine leaves without branching, a small root that is bitter with abundant resin, and is suitable for decoctions”. From this point onward, the two varieties of Zhu, Cangzhu and Baizhu, began to be differentiated. During the Song Dynasty, Ben Cao Tu Jing categorized them under the entry Zhu as Cangzhu and Baizhu. Zhu is characterized as follows: “Sprouting in spring, green in color without branches. Also known as Shanji, its leaves resemble thistles. The stem, which is greenish-red, resembles mugwort stalks and can grow to two or three feet in length. It blooms in summer, producing purple-green flowers similar to thistle blossoms, although some may have yellow-white flowers. The plant bears fruit after the summer solstice, and the shoots wither by autumn. Its root resembles ginger, featuring lateral fine roots, black skin, a yellowish-white core, and purple sap”. Based on these morphological descriptions—“yellow-white flowers, root resembling ginger, black skin, yellowish-white core, purple oily sap”—it is clear that Zhu corresponds precisely to the modern plants *A. lancea* (Mao-Cang-Zhu) or *A. chinensis* (Bei-Cang-Zhu). Tang Ye Ben Cao of the Yuan Dynasty classifies Baizhu and Cangzhu separately, outlining their distinct functions. According to the *Ben Cao Pin Hui Jing Yao* from the Ming Dynasty, Cangzhu produces shoots and leaves during spring. The leaves are slim and smooth, growing in pairs opposite each other. The stem resembles wormwood stalks, displaying a greenish-red hue and reaching a length of two to three feet. In the summer, it blossoms with thistle-like flowers in shades of purple and blue. Following the summer solstice, it yields fruit, and as autumn approaches, the shoots wilt. The root resembles ginger but lacks branches, featuring delicate lateral roots. Its skin is black, the flesh is yellow, and the core contains abundant sap. It offers a taste that is bitter, sweet, and pungent. The most esteemed roots are those harvested in spring, autumn, or winter, especially those easily frosted white. The Qing Dynasty’s Ben Cao Chong Yuan provides a detailed account of the stems, leaves, and rhizomes of Cangzhu, stating that near the root of *A. chinensis*, the leaves divide into three to five forks, with the upper leaves being narrow, elongated, and possessing a green, glossy sheen. The classification standards for Cangzhu and Baizhu during the Qing Dynasty correspond to contemporary norms, where Cangzhu represents a wild variety, while ancient Baizhu encompasses Zhezhu from the Ben Cao Meng Quan and Wuzhu from the Compendium of Materia Medica, both being cultivated types with their cultivation attributed to *Tao Hongjing* during the Northern and Southern Dynasties. Consequently, the Zhu utilized in the Han Dynasty’s Shang Han Lun should be Cangzhu rather than the subsequently cultivated Baizhu.

Herbal texts prior to the Song Dynasty only documented the regions where Zhu was produced. For instance, the Compendium of Famous Physicians’ Supplementary Records states: “Zhu grows in the valleys of Mount Na, Hanzhong”. The Collected Notes on Ben Cao Jing Ji Zhu states: “It is now found everywhere, but those from Jiangshan, Zishan, and Mount Mao are considered superior.” This indicates that Zhu was produced in Shaanxi and Jiangsu provinces, which remain the primary production areas for Cangzhu today. After the Song Dynasty, ancient herbal texts often distinguished the origins of Cangzhu and Baizhu. For instance, Ben Cao Tu Jing from the Song Dynasty records: “Zhu grows in the valleys of Mountain Zheng, Hanzhong, now known as Shaanxi Province. Now it is found everywhere, with those from Mountain Song and Mountain Mao being the finest”. The Compendium of Materia Medica records: “Cangzhu from Mount Mao is now considered the finest. Its root bark is black with white flesh and yellow spots. Cangzhu from other mountains has larger roots with yellow flesh and a fiercely pungent aroma”. The Republican-era Yao Wu Chan Chu Bian notes: “Tianshengzhu is originally produced in Xiushui County, Jiangxi Province”. As described above, Cangzhu has a broad distribution range. Its earliest recorded origin is Hanzhong, Shaanxi, later gradually expanding to Jiangsu, Henan, and Hubei. The highest quality Cangzhu comes from Mountain Mao, Jiangsu. Currently, it is primarily distributed in Shandong, Jiangsu and Zhejiang, Hubei, Sichuan, and other provinces. With increasing recognition, Bei-Cang-Zhu (*A. chinensis*) has gradually become mainstream, and its functions and effects are comparable to those of Mao-Cang-Zhu (*A. lancea*).

### 3.2. Textual Research of Traditional Ethnopharmacology, Uses and Prescriptions

Ethnopharmacology and ethnic pharmacology are integral components of the theoretical framework of Traditional Chinese Medicine and serve as foundational principles for clinical prescriptions. AR is characterized by its pungent and warm properties, accompanied by a bitter taste, and is associated with the spleen, stomach, and liver meridians. It functions to eliminate dampness, strengthen the spleen, dispel wind, disperse cold, and enhance vision. This herb is predominantly utilized in the treatment of conditions such as dampness obstructing the middle jiao, epigastric and abdominal distension, diarrhea, edema, beriberi paralysis, rheumatic arthralgia, wind-cold common cold, night blindness, and blurred vision.

**Figure 2:**
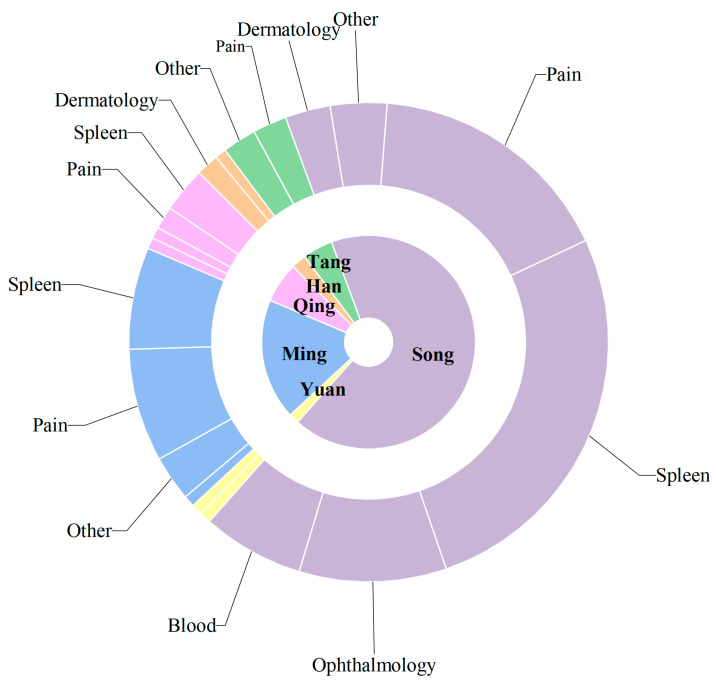
Historical Application of Atractylodis Rhizoma: A Sunburst Diagram of Therapeutic Categories by Dynasty. Note: (1) Inner Ring: Dynasties, arranged in historical sequence including Han, Tang, Song, Yuan, Ming, Qing. (2) Outer Ring: Therapeutic Categories, derived from the “Traditional uses/Efficacy” field in each formula description. Primarily divided into six categories: Gastrointestinal: Addresses spleen-stomach deficiency, diarrhea, vomiting, abdominal distension, etc. External/Pain: Includes typhoid fever, headache, body pain, rheumatism, joint pain, etc. Ophthalmology: Treats redness and swelling of the eyes, corneal opacity, night blindness, etc. Gynecology/Blood: Covers pregnancy, postpartum conditions, blood stasis, etc. Dermatology: Treats sores, hives, scabies, and tinea. Other: Includes tonifying, qi regulation, phlegm-fluid retention, and other non-categorizable uses. (3) Values: Represents the number of formulas in each category per dynasty. The sum equals the total number of formulas for that dynasty. (4) Colors: Different colors represent different dynasties.

Upon verification, a total of 197 internal formulas and 3 external formulas were identified, of which 54 formulas incorporated rice water-processed AR to address spleen-stomach disharmony and related conditions (refer to Table S1). An analysis of the historical application of AR (Figure 2) spans six dynasties and six disease categories. The Song Dynasty represents the peak period for AR utilization, with the highest number of formulas (88) and the broadest therapeutic scope, particularly emphasizing gastrointestinal disorders and external pathogen/pain conditions, while ophthalmic formulas also occupied a significant proportion. The Ming Dynasty follows with 24 formulas, where external pathogen/pain applications slightly outnumber gastrointestinal ones, reflecting the continued use of AR in treating rheumatic arthralgia. Although fewer formulas are recorded in the Qing Dynasty, gastrointestinal applications remain dominant. Notably, ophthalmic uses are predominantly concentrated in the Song Dynasty and rarely appear in other periods, possibly due to the extensive inclusion of ophthalmic formulas in the comprehensive text Sheng Ji Zong Lu. These findings demonstrate that AR has been consistently applied to gastrointestinal disorders throughout history, further validating the scientific basis and continuity of its traditionally recognized functions of “drying dampness and strengthening the spleen” that persist in modern clinical practice.

### 3.3. Research on Modern Preparations

AR is renowned for its distinctive therapeutic properties, including the ability to dry dampness, fortify the spleen, dispel wind, and alleviate cold. These properties have been validated through extensive historical use, resulting in the preservation of numerous traditional formulations that persist to the present day. With the progression of modern pharmacology and advancements in Traditional Chinese Medicine, these compound formulations have been further refined and applied (see Table S2). Consequently, a variety of formulations, such as granules, capsules, tablets, and pills, have been developed. These are extensively employed to treat conditions like dampness obstructing the middle burner and spleen-stomach disharmony, effectively drying dampness and enhancing spleen function.

**Figure 3:**
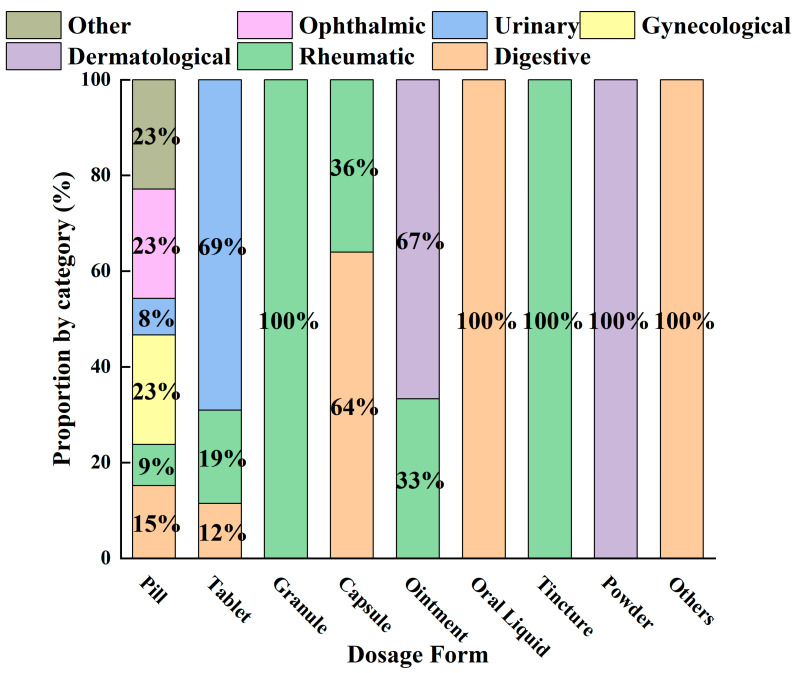
Distribution of Dosage Forms and Therapeutic Categories in Modern Atractylodis Rhizoma-Containing Patent Medicines.

Currently, a statistical analysis of the 54 AR-containing formulations recorded in the Chinese Pharmacopoeia reveals the overall distribution characteristics of this herb in modern patent medicines (Figure 3). The findings demonstrate a pattern characterized by diverse dosage forms, concentrated therapeutic indications, and the integration of traditional and modern pharmaceutical approaches. Pills serve as the core carrier, supporting the extensive application of AR in the digestive, urinary, and rheumatic systems. Meanwhile, modern dosage forms—such as granules, capsules, and tablets—expand the routes of administration and scope of indications while preserving its traditional efficacy. This distribution not only validates the millennium-old medicinal use of AR in a contemporary context but also provides data support and directional guidance for future drug development.

## 4. Phytochemistry

To date, a total of 327 small molecules have been identified from AR, encompassing a diverse range of structural classes including sesquiterpenoids, mono-terpenoids, polyacetylenes, triterpenoids, phenolic acids, flavonoids, and steroidal compounds [3]. While this extensive inventory provides a valuable foundation, a critical analytical evaluation is necessary to differentiate compounds based on their abundance, biosynthetic origin, and distribution between the two pharmacopoeial species, *A. lancea* (AL) and *A. chinensis* (AC). Such stratification enhances our understanding of the chemotaxonomic markers and the true pharmacologically active principles.

From the perspective of relative abundance, the 327 compounds can be stratified into major bioactives (Table 2), minor components, and trace constituents. The primary and most studied components of AR are lipophilic and accumulate in the essential oil and resin, predominantly comprising sesquiterpenoids and polyacetylenes. Among sesquiterpenoids, atractylon, hinesol, β-eudesmol, and the atractylenolides (I, II, III) are consistently reported as the dominant compounds and form the core bioactive foundation for pharmacological effects such as gastroprotection and anti-inflammation [14]. Quantitative analyses have demonstrated that hinesol and β-eudesmol are characteristically high in AL, while atractylon and γ-eudesmol are notably abundant in AC. Polyacetylenes, particularly atractylodin and its derivatives such as atractylodinol, represent another key group of major bioactives with hepatoprotective properties; these compounds are significantly more concentrated in AL compared to AC. In contrast, a substantial portion of the identified compounds—including numerous saturated and unsaturated hydrocarbons (e.g., various alkanes) and many mono- and sesquiterpene hydrocarbons—are present only in trace amounts. While these trace constituents contribute to the complex aromatic profile of the herb, they are unlikely to be primary drivers of its classical therapeutic actions and are more relevant for establishing complete volatile fingerprints for species differentiation or authenticity testing.

A critical assessment of the phytochemical dataset must also consider the distinction between genuine plant metabolites and potential analytical artifacts. The biosynthetic relationship between atractylon and the atractylenolides warrants particular attention. Atractylon is chemically unstable and readily undergoes autoxidation upon exposure to air, light, or heat [15]. Mechanistic studies have elucidated that atractylon undergoes oxidative conversion through a series of reactions involving water addition, ring cleavage, and carboxylation to generate atractylenolide I, which can further transform into atractylenolides II and III. Consequently, while these lactones are certainly present in processed crude drugs, their quantified levels may not fully represent the native metabolic profile of the fresh rhizome, and a proportion of the measured atractylenolides—particularly in dried or aged samples—may be artifacts derived from atractylon oxidation. This phenomenon is further supported by processing studies demonstrating that stir-frying with bran, a common traditional processing method, leads to decreased levels of both sesquiterpenoids and polyacetylenes, likely due to thermal degradation and oxidative transformations [16]. Additionally, the presence of phthalate esters such as diethyl phthalate, diisobutyl phthalate, and bis(2-ethylhexyl) phthalate is a strong indicator of contamination rather than genuine phytochemical production, as these compounds are ubiquitous plasticizers that readily leach from plastic labware and storage containers during analytical workflows [17].

Comparative analysis between the two official species reveals distinct chemical profiles that underpin their differentiation in traditional medicine and necessitate species specification in modern pharmacological research. The most prominent chemotaxonomic difference lies in the dominant sesquiterpenoid patterns: AL is characterized by high concentrations of hinesol, β-eudesmol, and atractylol, whereas AC is defined by its high content of atractylon and γ-eudesmol, with compounds like β-selinene also showing higher relative abundance in AC. Furthermore, AL exhibits significantly higher accumulation of polyacetylenes, particularly atractylodin and acetylatractylodinol, compared to AC, making polyacetylene content a key differentiating factor that may contribute to variations in reported bioactivities such as hepatoprotection. While most of the remaining compounds, especially trace hydrocarbons and ubiquitous fatty acids, are present in both species, certain compounds such as atrachinenins D, E, F, and G have been reported exclusively in AC based on current literature, representing potential unique biomarkers. These clear qualitative and quantitative differences underscore the absolute necessity of specifying the species used in any experimental study to ensure reproducibility and the correct attribution of observed pharmacological effects.

**Table 2:** Some of the chemical constituents from Atractylodis Rhizoma.

NO.	Compounds	Structure	AL	AC	Class	References
1	α-Pinene	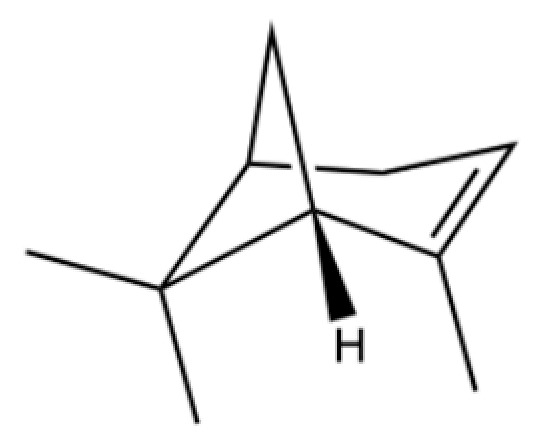	*	*	Monoterpenoid	[18,19]
2	Nonane, 2,6-dimethyl-	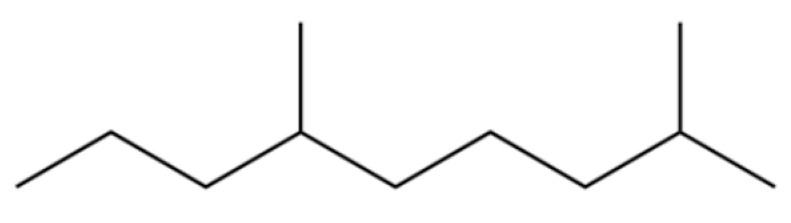	*	*	Alkane	[20]
3	Decane, 4-methyl-	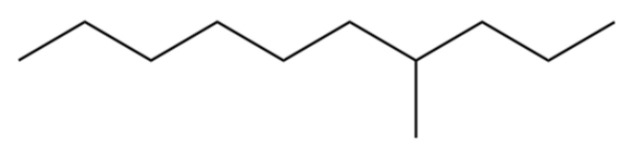	*	*	Alkane	[20]
4	2-Hexanone	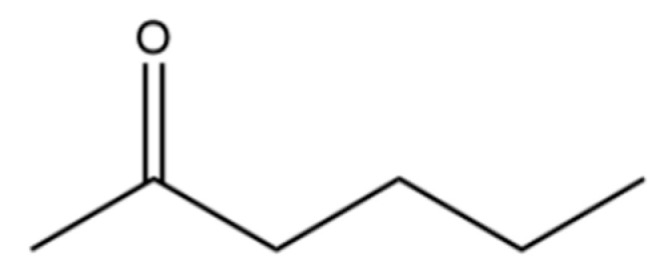	*	*	Ketone	[20]
5	α-Phellandrene	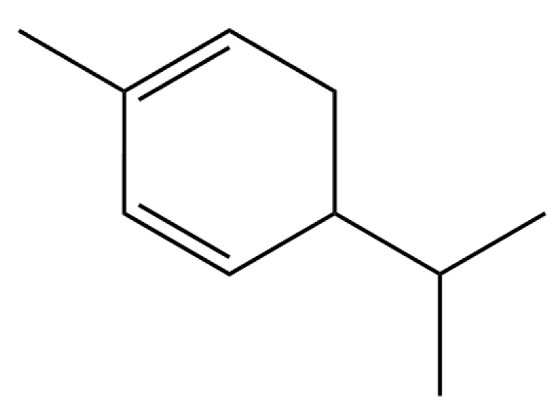	*	*	Monoterpenoid	[21]
6	Undecane	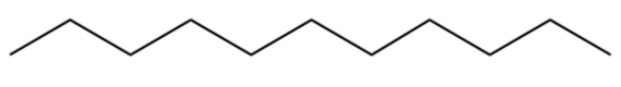	*	*	Alkane	[20]
7	3-Hexanol	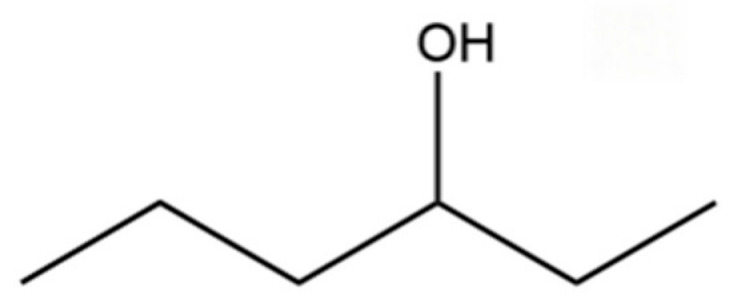	*	*	Alcohol	[20]
8	Dodecane	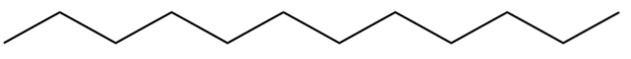	*	*	Alkane	[20]
9	2,6-Dimethylundecane	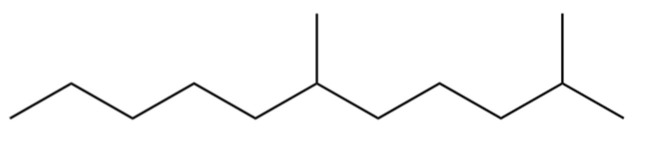	*	*	Alkane	[20]
10	2-Hexanol	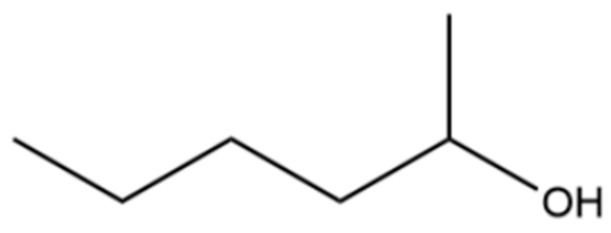	*	*	Alcohol	[20]
11	Tridecane	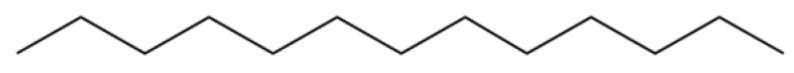	*	*	Alkane	[20]
12	2,7,10-trimethyl-Dodecane	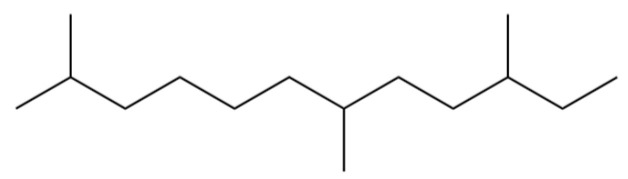	*	*	Alkane	[20]
13	Silphiperfol-5-ene	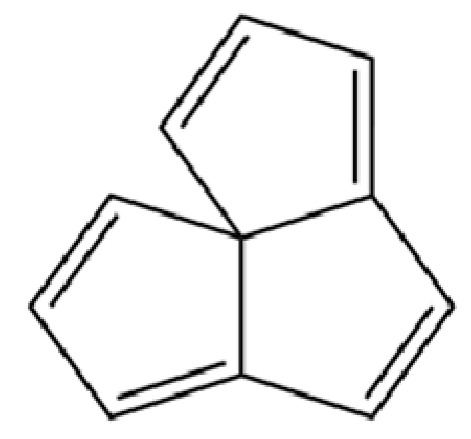	*	*	Sesquiterpenoid	[22]
14	2-Methyltridecane	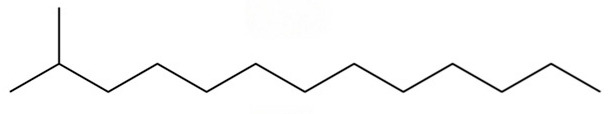	*	*	Alkane	[20]
15	Atrachinenins G	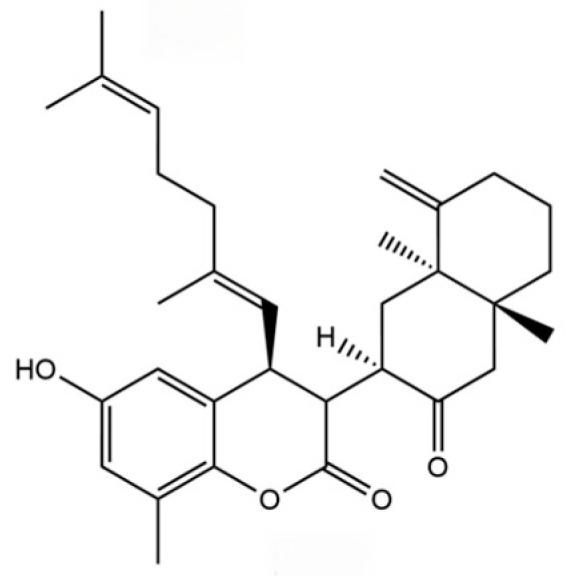	/	**	Sesquiterpenoid	[23]
16	2,6,10-Trimethyltridecane	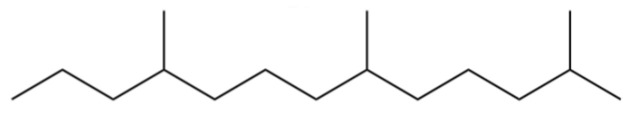	*	*	Alkane	[20]
17	Tetradecane	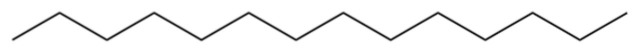	*	*	Alkane	[20]
18	α-Guaiene	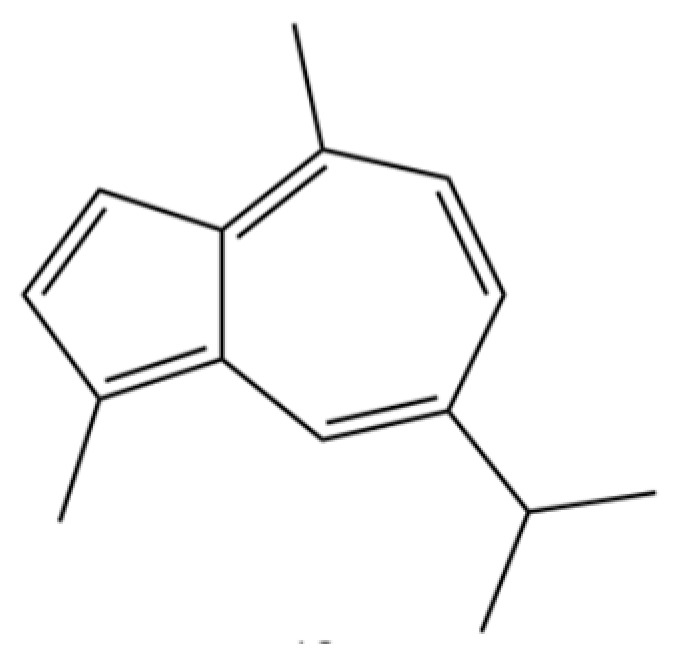	*	*	Sesquiterpenoid	[3]
19	Pentadecane	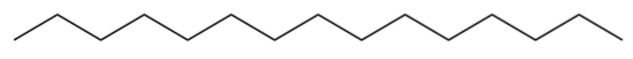	*	*	Alkane	[20]
20	Modephene	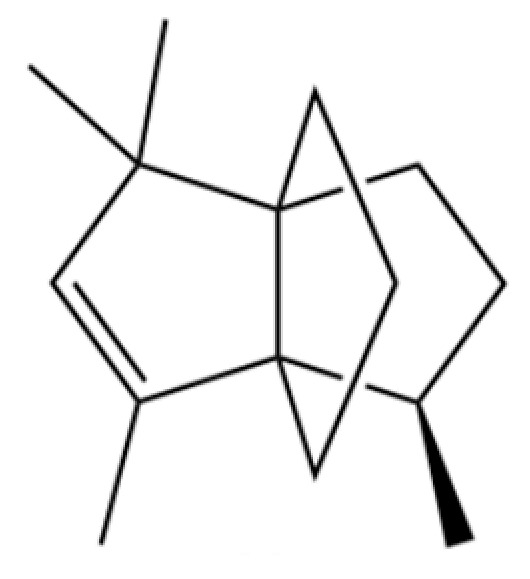	*	*	Sesquiterpenoid	[24]
21	Cyperene	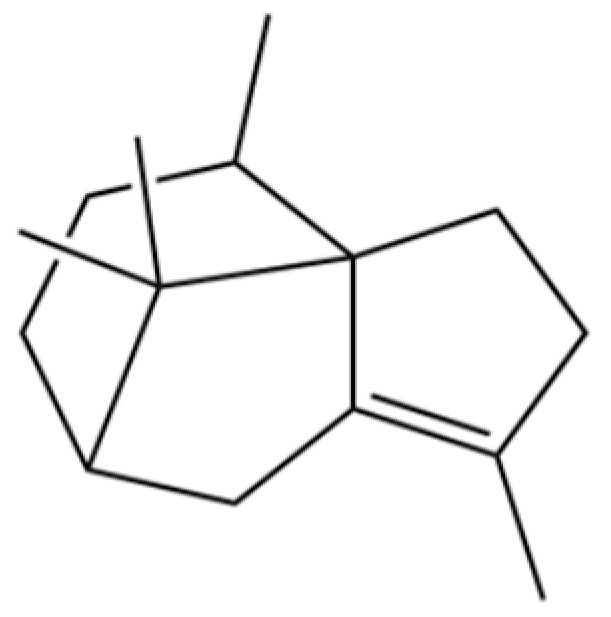	*	*	Sesquiterpenoid	[25]
22	β-Elemene	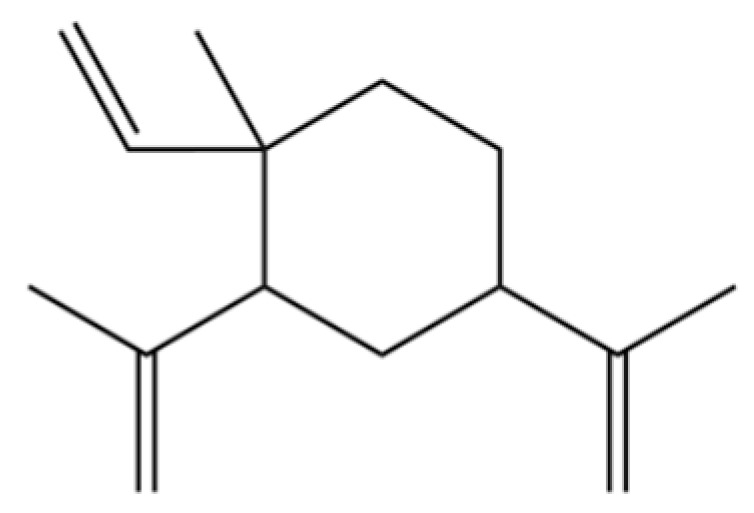	*	*	Sesquiterpenoid	[21]
23	Isocomene	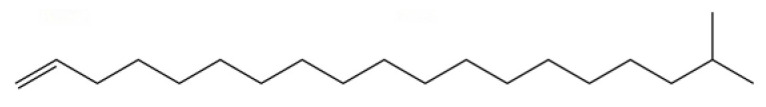	*	*	Sesquiterpenoid	[20]
24	β-Isocomene	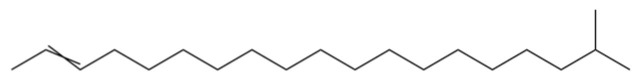	*	*	Sesquiterpenoid	[20]
25	Caryophyllene	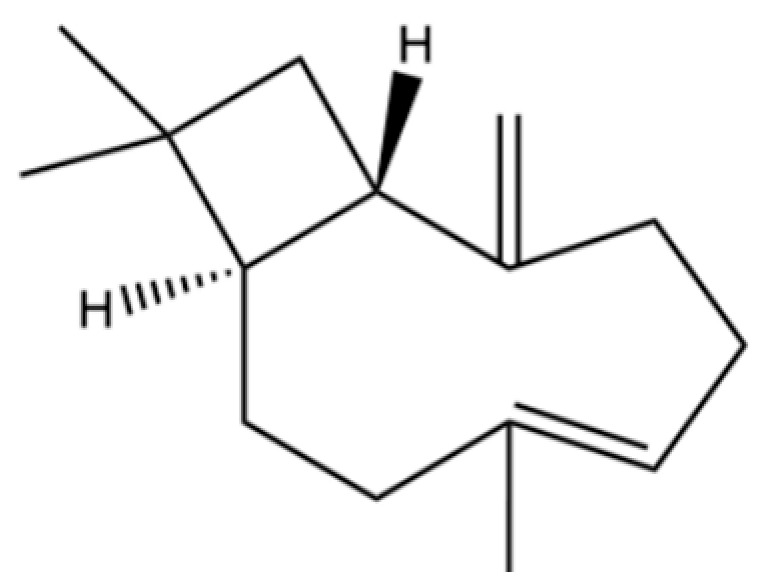	*	*	Sesquiterpenoid	[25]
26	Aciphyllene	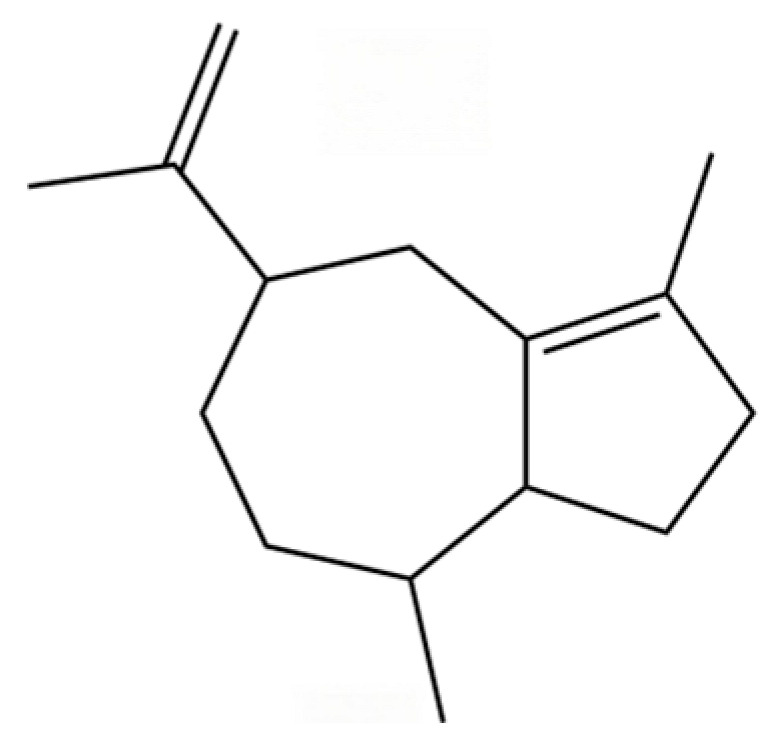	*	*	Sesquiterpenoid	[20]
27	β-Famesene	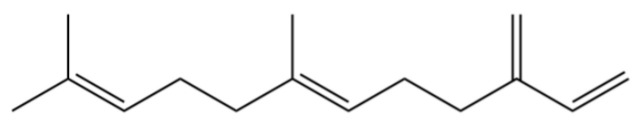	*	*	Sesquiterpenoid	[20]
28	Atrachinenins F	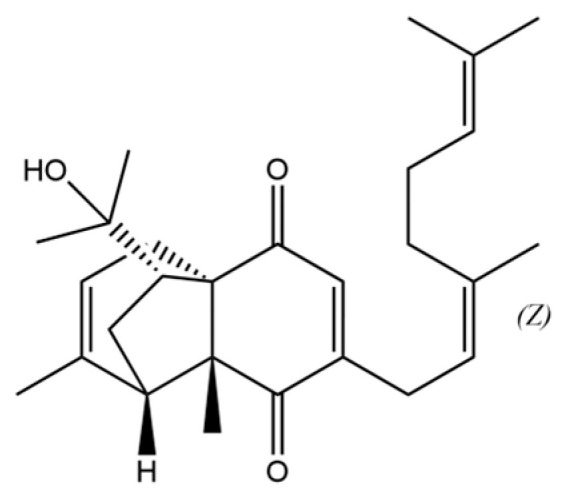	/	**	Sesquiterpenoid	[23]
29	Octadecane	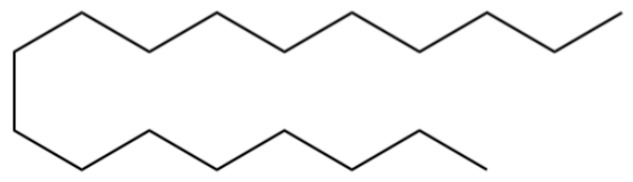	*	*	Alkane	[26]
30	Humulene	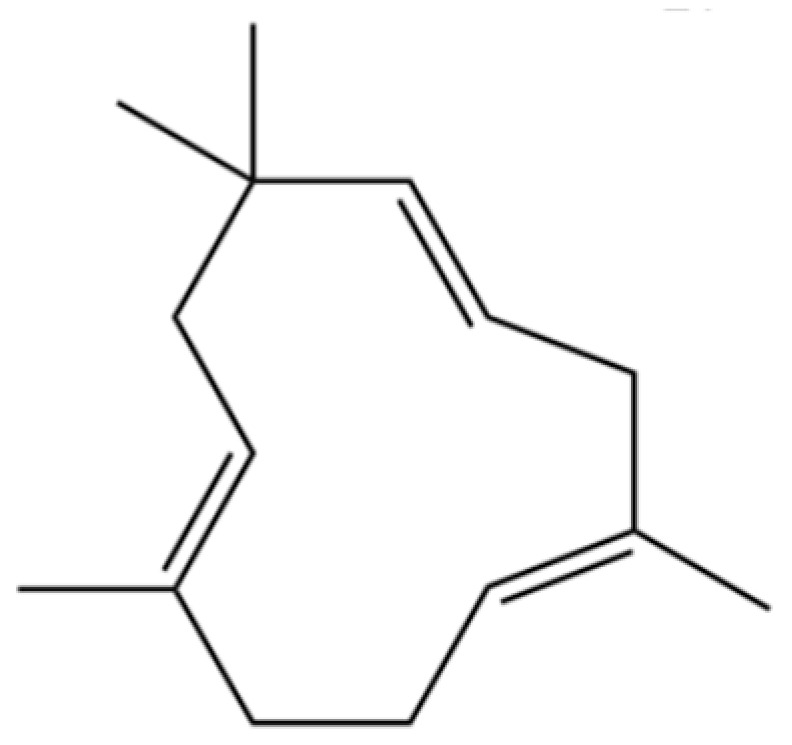	*	*	Sesquiterpenoid	[21]
31	2-Isopropenyl-4a, 8-dimethyl-1,2,3,4,4a, 5,6,7-octahydronaphthalene	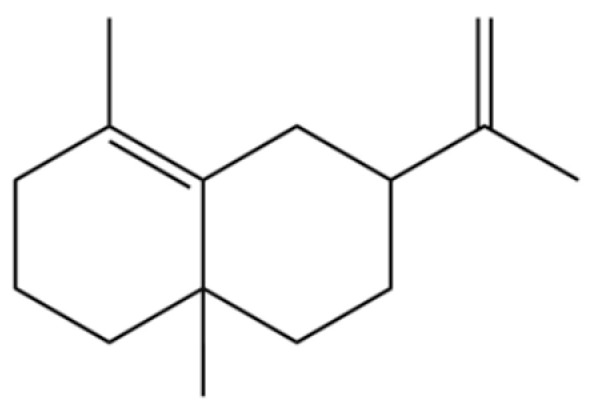	*	*	Sesquiterpenoid	[20]
32	(+)-Eudesma-4(14),7(11)-dien- 8-one	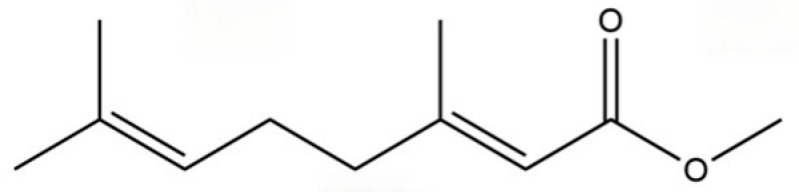	*	*	Sesquiterpenoid	[25]
33	Isoborneol	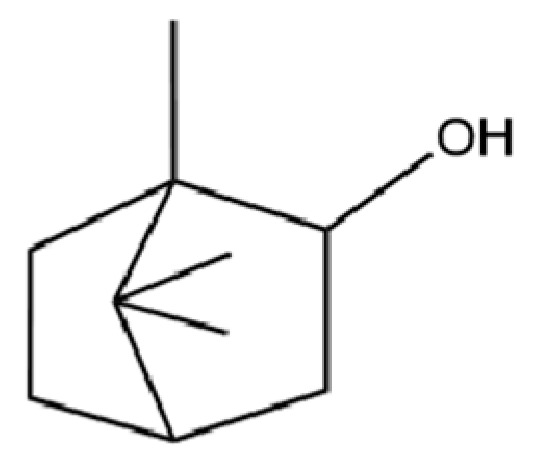	*	*	Monoterpenoid	[20]
34	1-Methyl-4-(6-methylhept-5-en-2-yl) cyclohexa-1,3-diene	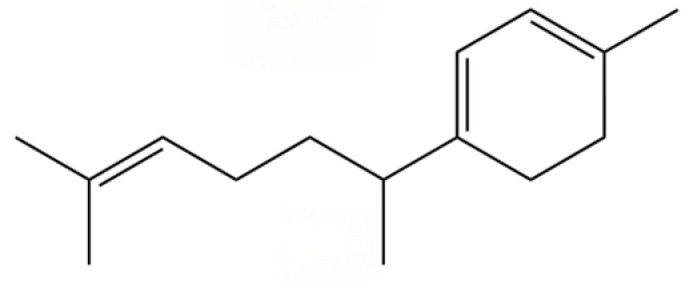	*	*	Sesquiterpenoid	[20]
35	Germacrene D	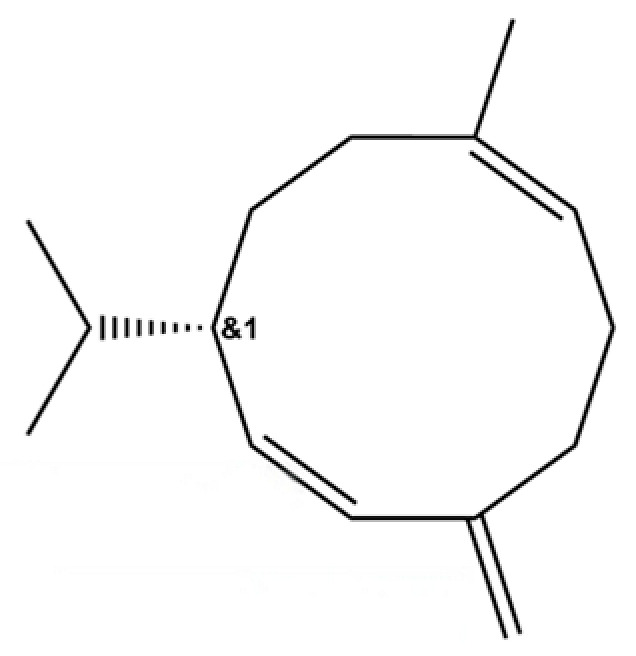	*	*	Sesquiterpenoid	[27]
36	β-selinene	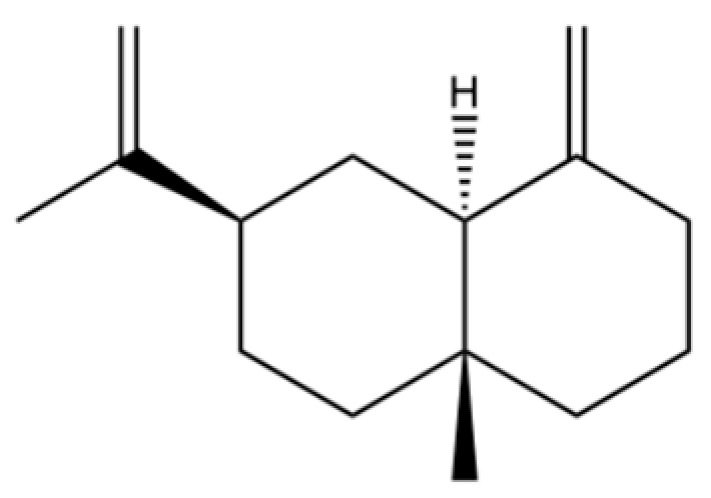	*	**	Sesquiterpenoid	[21]
37	2-Cyclohexen-1-ol, 3-methyl-6-(1-methylethyl)-, cis-	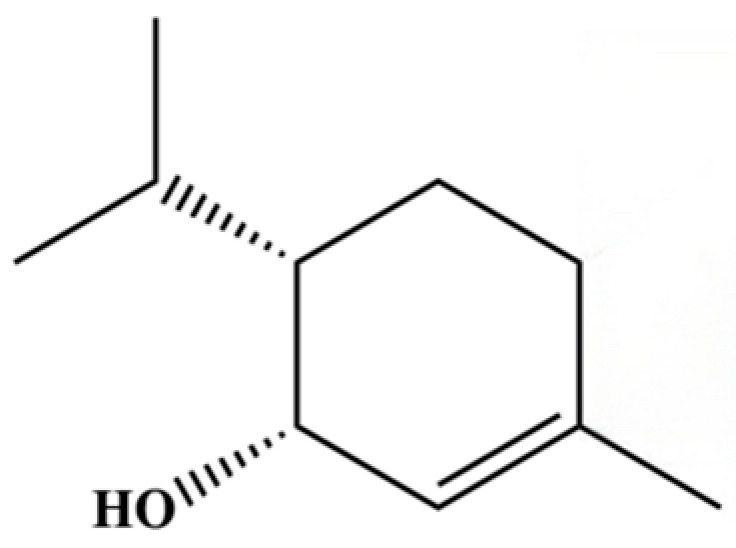	*	*	Monoterpenoid	[20]
38	β-Curcumen	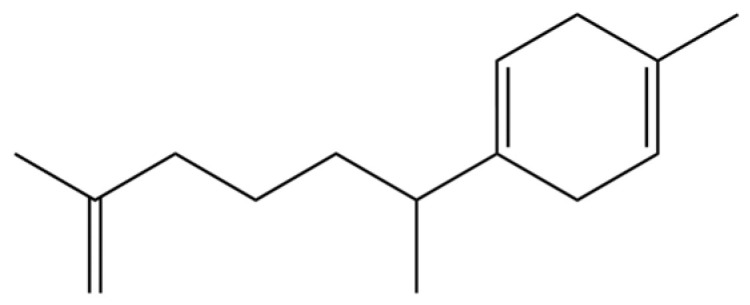	*	*	Sesquiterpenoid	[20]
39	Guaia-1 (10),11-diene	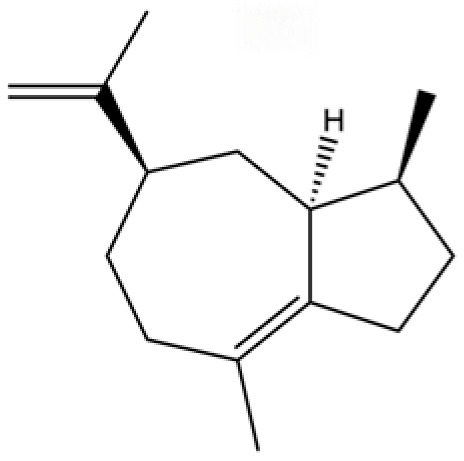	*	*	Sesquiterpenoid	[20]
40	2,6-Octadien-1-ol, 3,7-dimethyl-, acetate	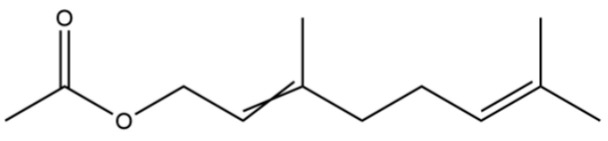	*	*	Monoterpenoid	[25]
41	Citronellol	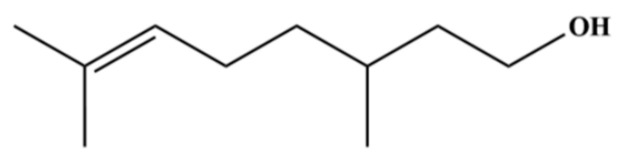	*	*	Monoterpenoid	[20]
42	γ-Cadinene	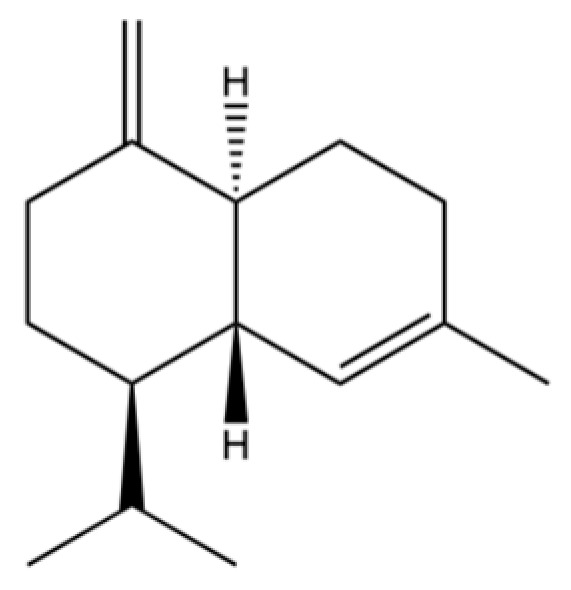	*	*	Sesquiterpenoid	[21]
43	Methyl salicylate	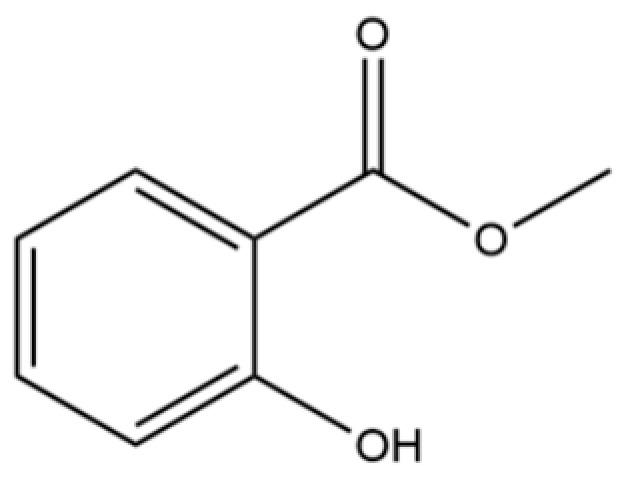	*	*	Phenolic ester	[20]
44	β-Sesquiphellandrene	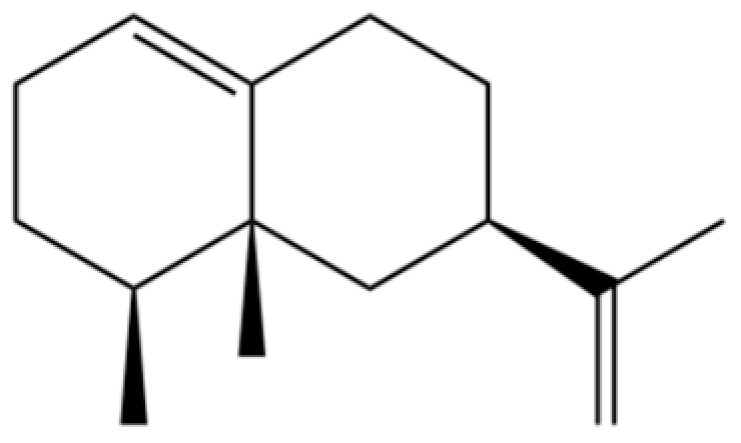	*	*	Sesquiterpenoid	[25]
45	β-Vatirenene	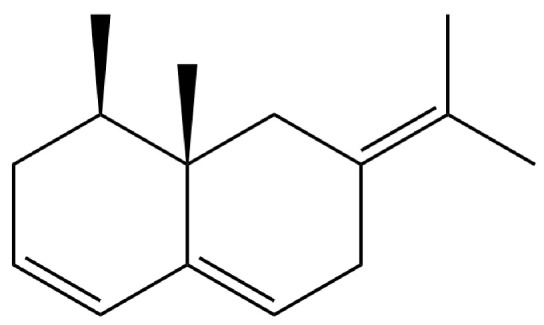	*	*	Sesquiterpenoid	[25]
46	α-Curcumene	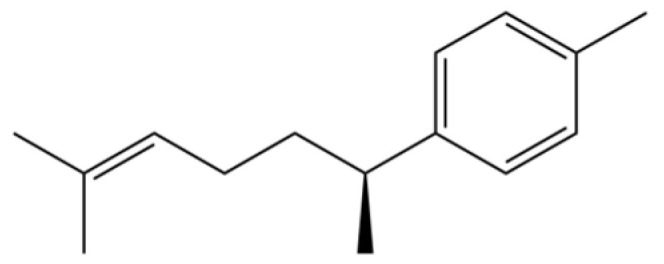	*	*	Sesquiterpenoid	[21]
47	Eremophilene	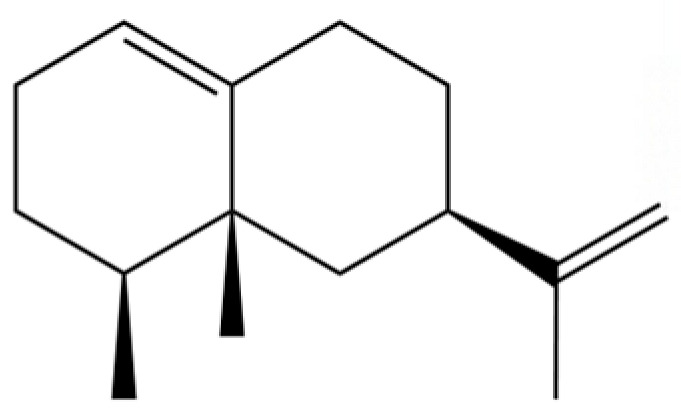	*	*	Sesquiterpenoid	[20]
48	cis-Sabinol	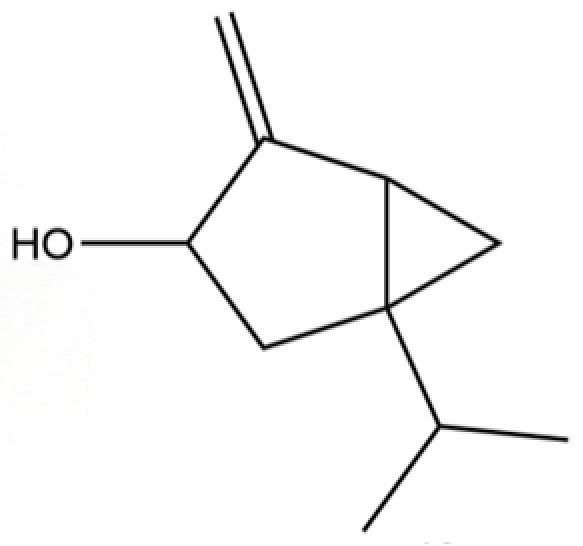	*	*	Monoterpenoid	[20]
49	γ-Elemene	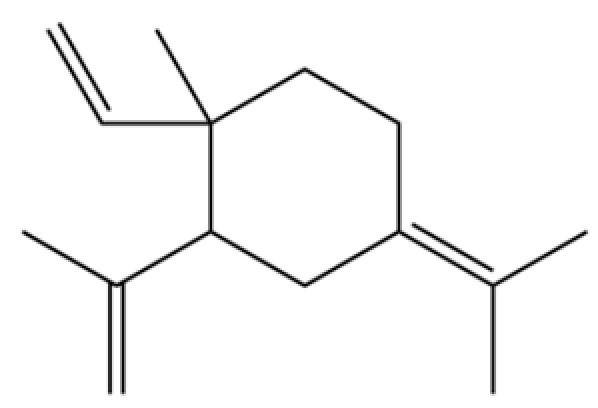	*	*	Sesquiterpenoid	[25]
50	Nerol	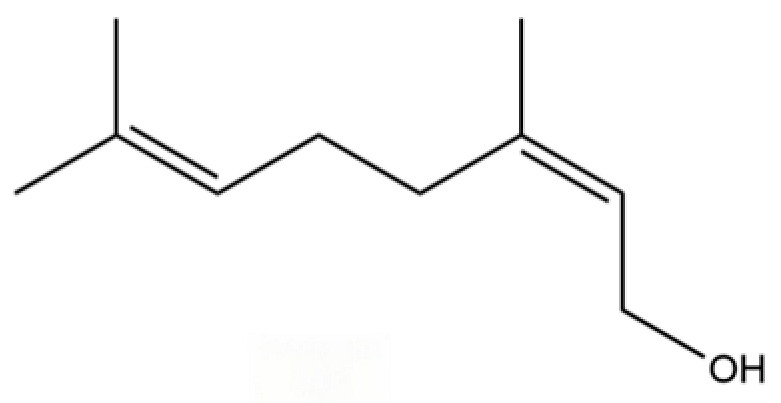	*	*	Monoterpenoid	[20]
51	Heptadecane, 9-hexyl-	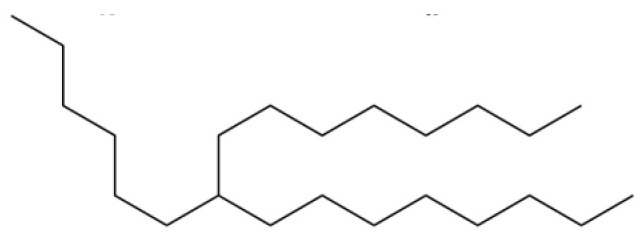	*	*	Alkane	[20]
52	Butylated hydroxytoluene	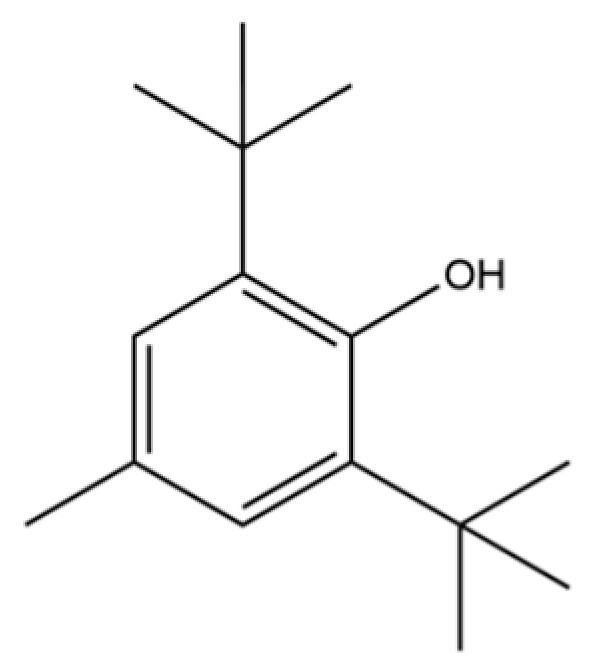	*	*	Phenolic antioxidant	[20]
53	Cedrene epoxide	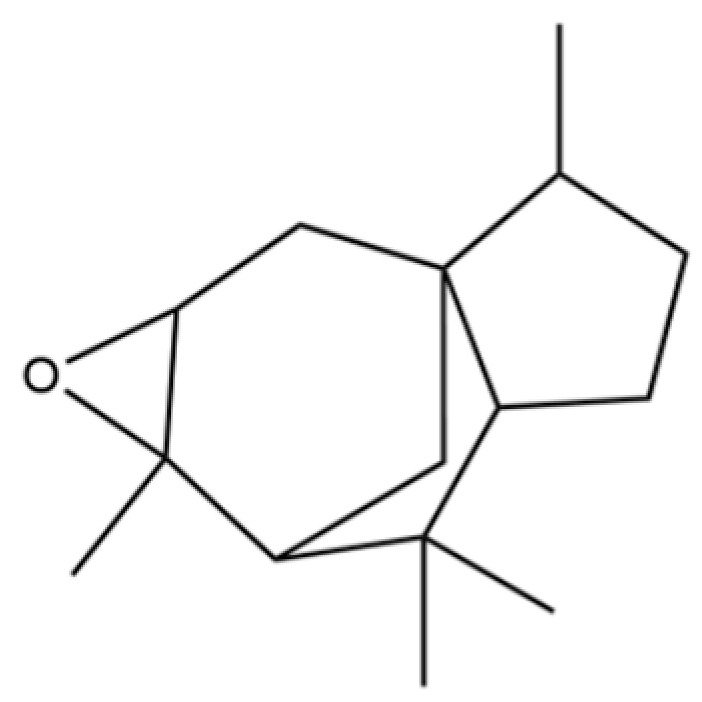	*	*	Sesquiterpenoid	[20]
54	(3S,3aR,3bR,4S,7R,7aR)-4-Isopropyl-3,7-dimethyloctahydro -1H-cyclopenta[1,3]cyclopropa[1,2]benzen-3-ol	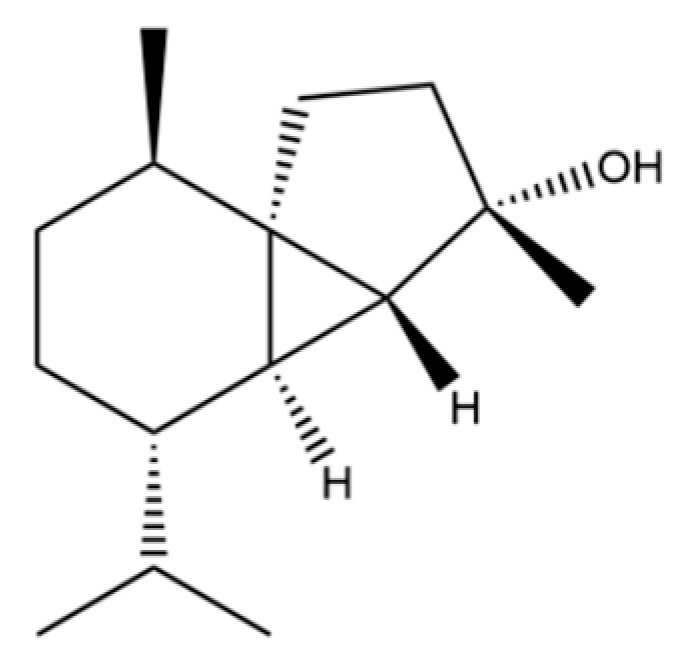	*	*	Sesquiterpenoid	[20]
55	trans-Longipinocarveol	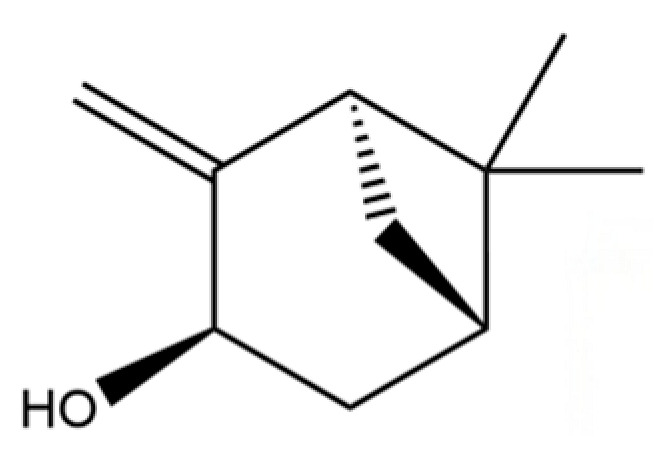	*	*	Sesquiterpenoid	[20]
56	Nerolidol	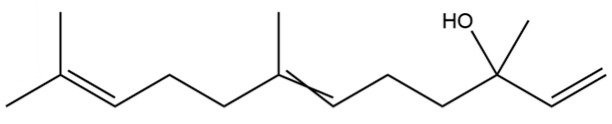	*	*	Sesquiterpenoid	[28]
57	Humulene epoxide II	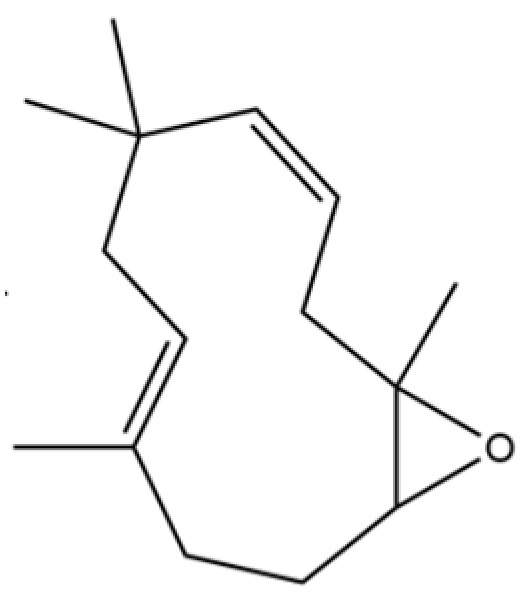	*	*	Sesquiterpenoid	[20]
58	Epicubenol	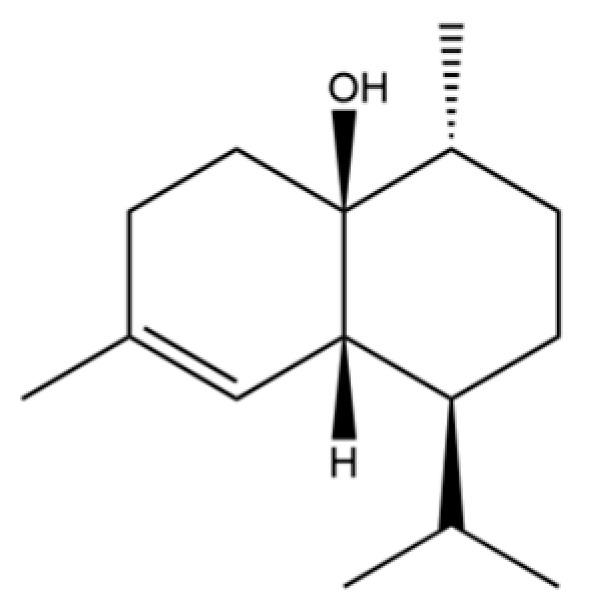	*	*	Sesquiterpenoid	[20]
59	5-Azulenemethanol, 1,2,3,4,5,6,7,8-octahydro-α,α,3,8-tetramethyl-	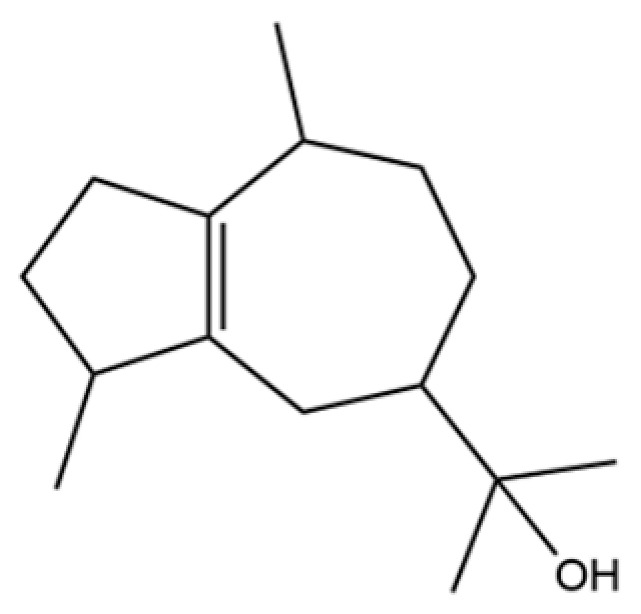	*	*	Sesquiterpenoid	[20]
60	1 (2H)-Naphthalenone, octahydro-4a, 8a-dimethyl-7-(1-methylethyl)-,[4aR-(4aα,7ß,8aα)]-	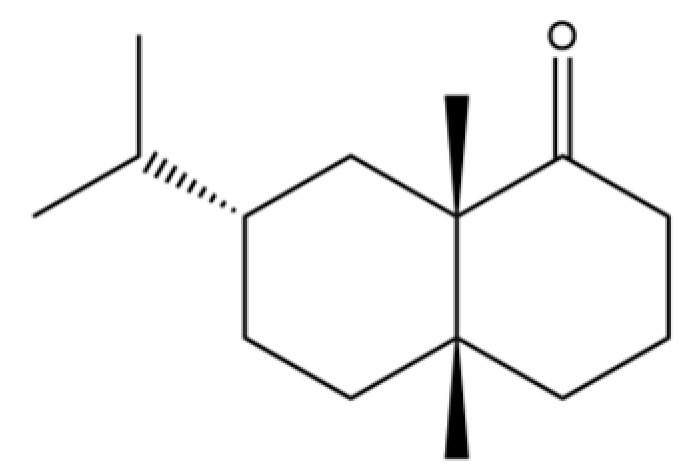	*	*	Sesquiterpenoid	[20]
61	Atractylon	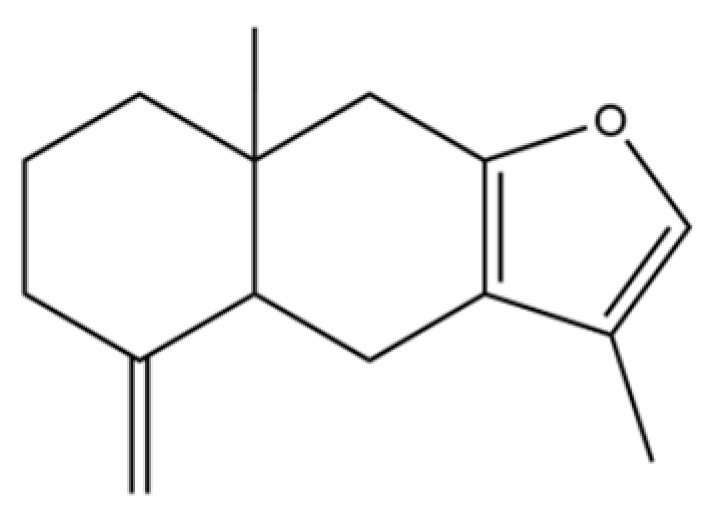	*	**	Sesquiterpenoid	[21]
62	γ-eudesmol	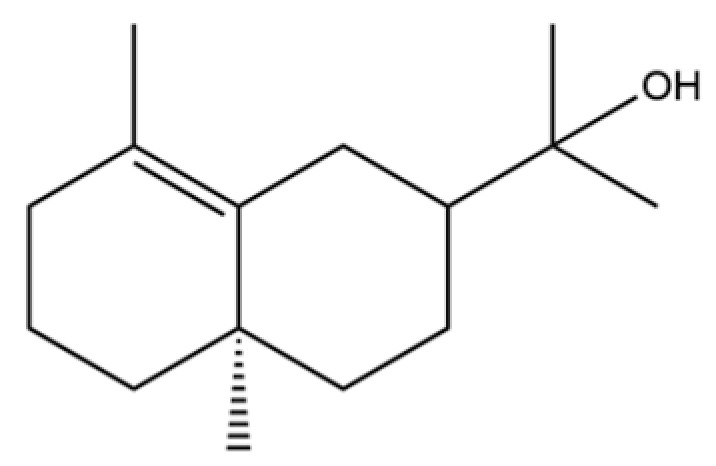	*	**	Sesquiterpenoid	[25]
63	8,14-Cedranoxide	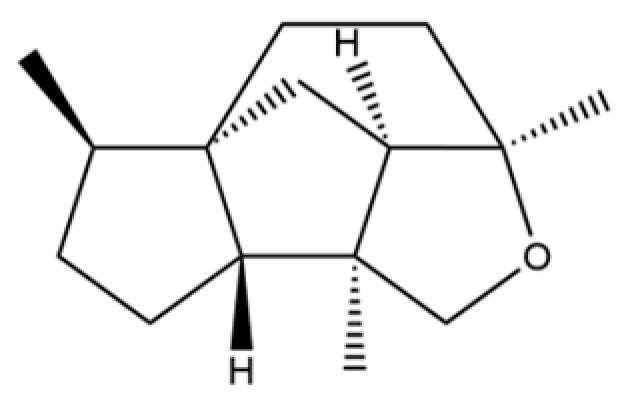	*	*	Sesquiterpenoid	[20]
64	Thymol	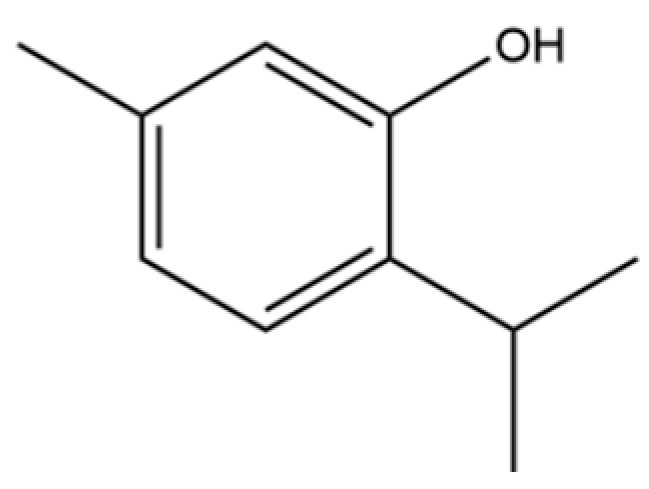	*	*	Monoterpenoid phenol	[25]
65	Agarospirol	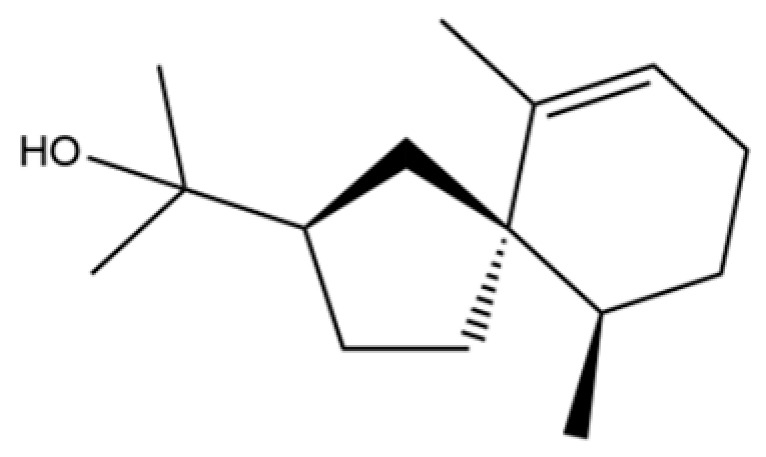	*	*	Sesquiterpenoid	[29]
66	Hinesol	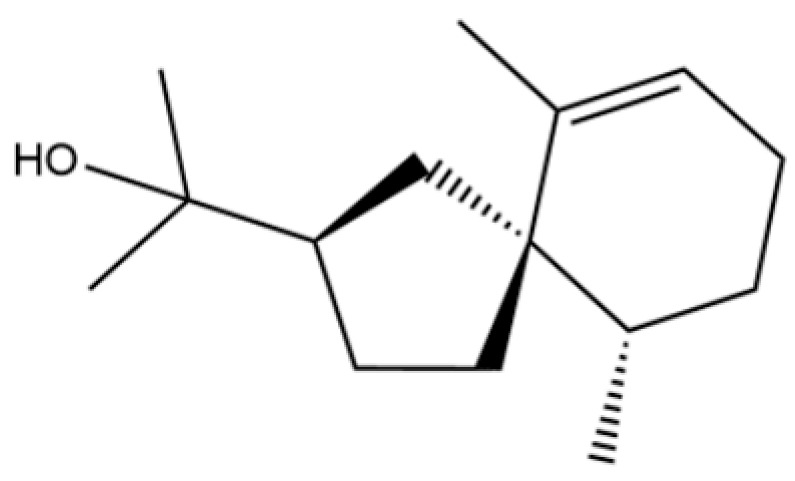	**	*	Sesquiterpenoid	[21]
67	trans-Valerenyl acetate	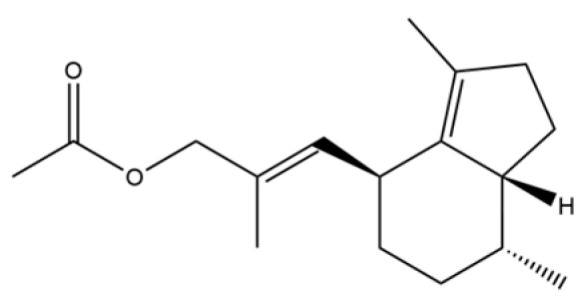	*	*	Sesquiterpenoid	[20]
68	2-methyl-5-(1-methylethyl)-Phenol	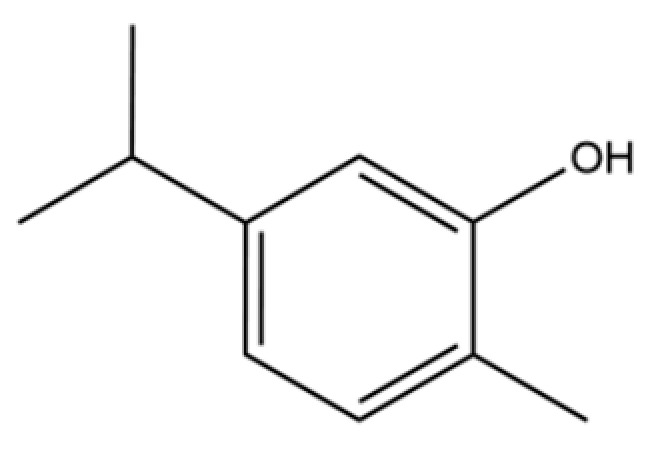	*	*	Phenolic	[20]
69	α-Bisabolol	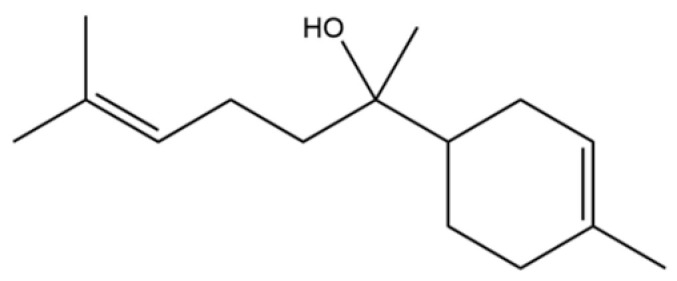	*	*	Sesquiterpenoid	[25]
70	Atractylol	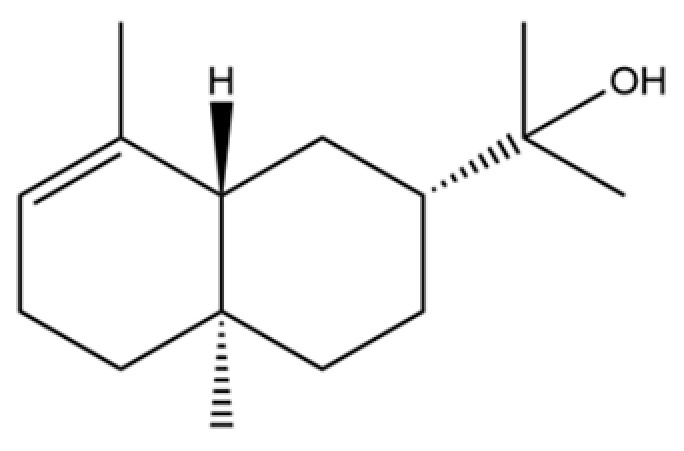	**	*	Sesquiterpenoid	[30]
71	β-Eudesmol	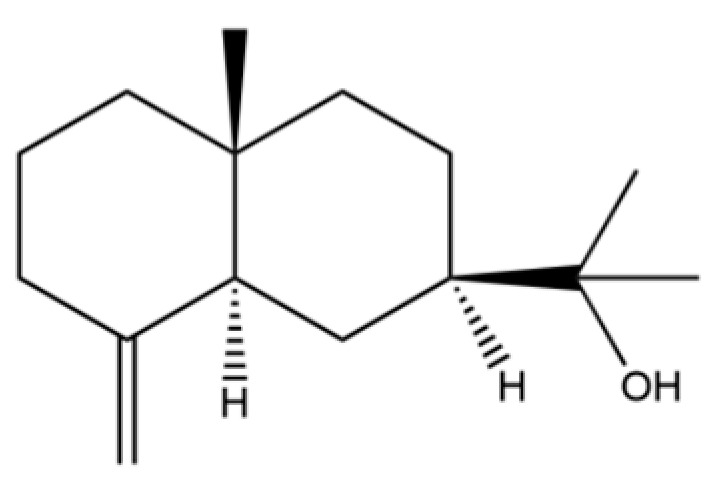	**	*	Sesquiterpenoid	[21]
72	3,7-dimethyl-6-Octenoic acid	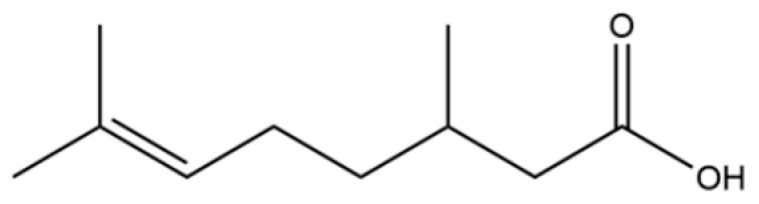	*	*	Monoterpenoid acid	[20]
73	α-Elemol	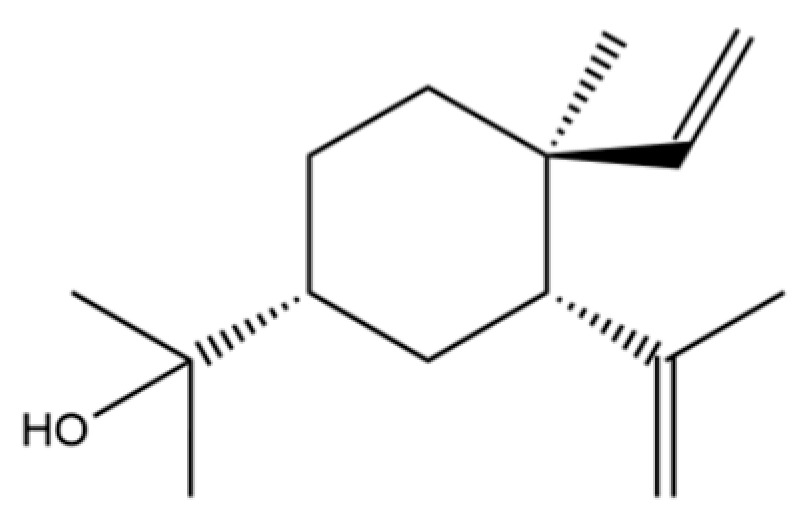	*	*	Sesquiterpenoid	[31]
74	Neointermedeol	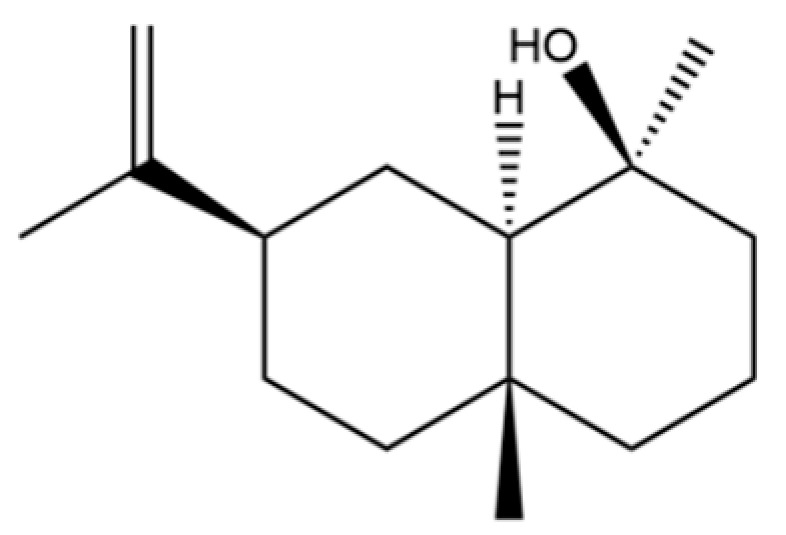	*	*	Sesquiterpenoid	[20]
75	Dehydrofukinone	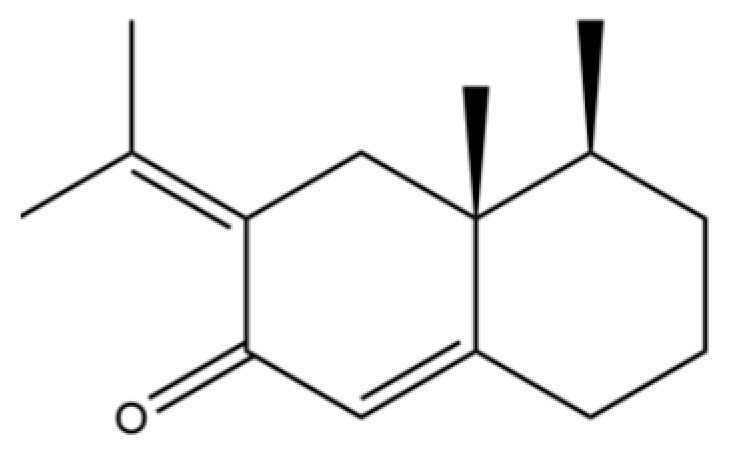	*	*	Sesquiterpenoid	[20]
76	Isoaromadendrene epoxide	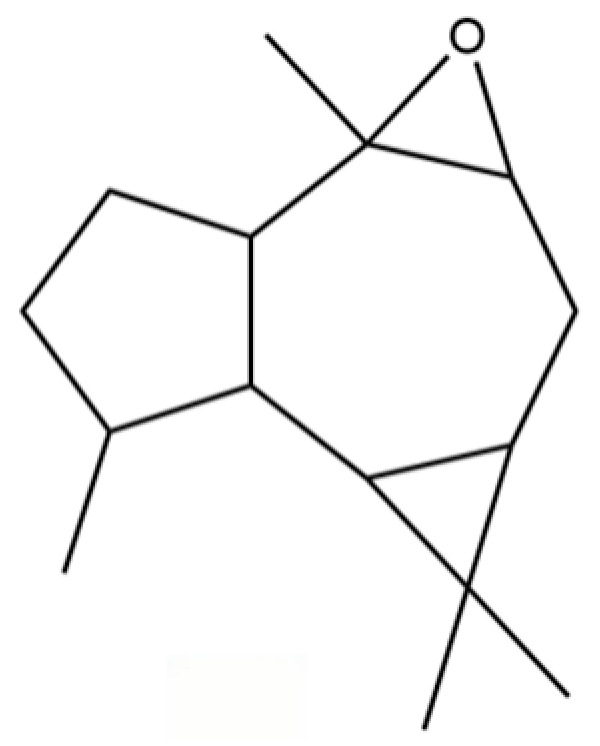	*	*	Sesquiterpenoid	[20]
77	Juniper camphor	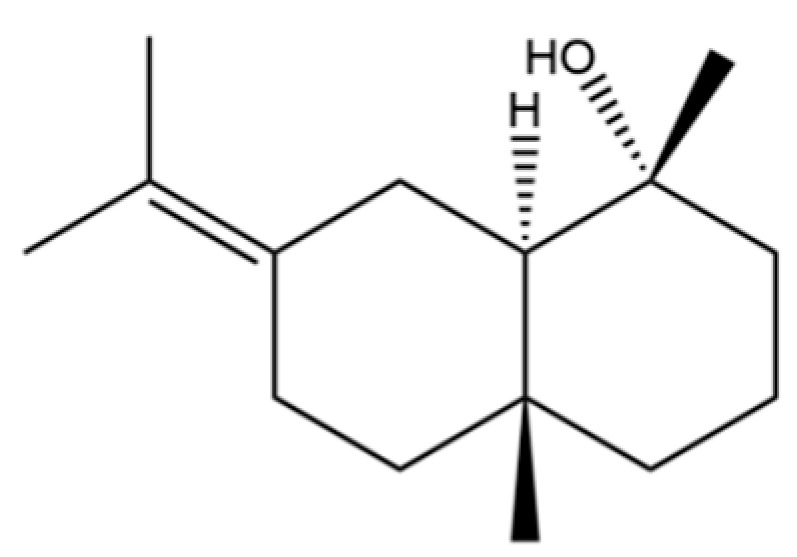	*	*	Sesquiterpenoid	[3]
78	2,4-Di-tert-butylphenol	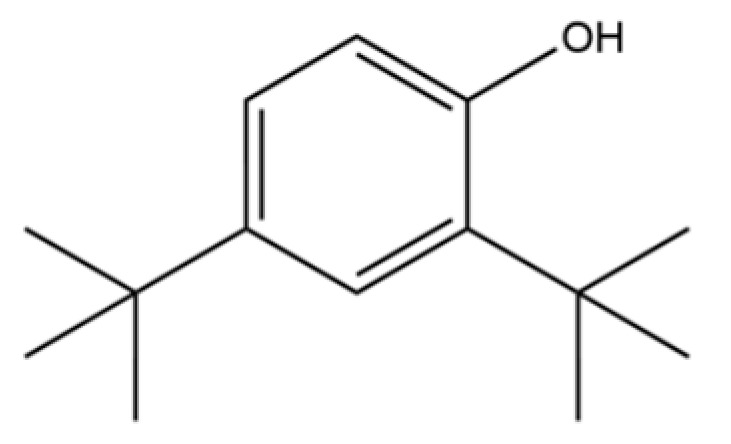	*	*	Phenolic	[32]
79	2 (1H) Naphthalenone, 3,5,6,7,8,8a-hexahydro-4,8a-	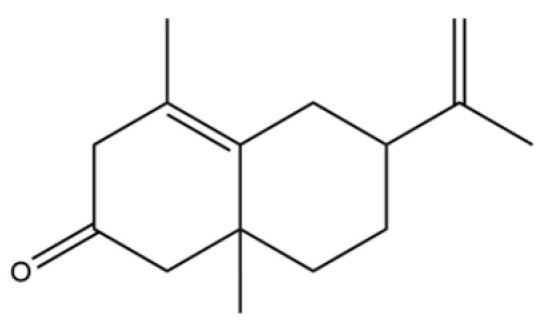	*	*	Sesquiterpenoid	[20]
80	Aromadendrene oxide-(1)	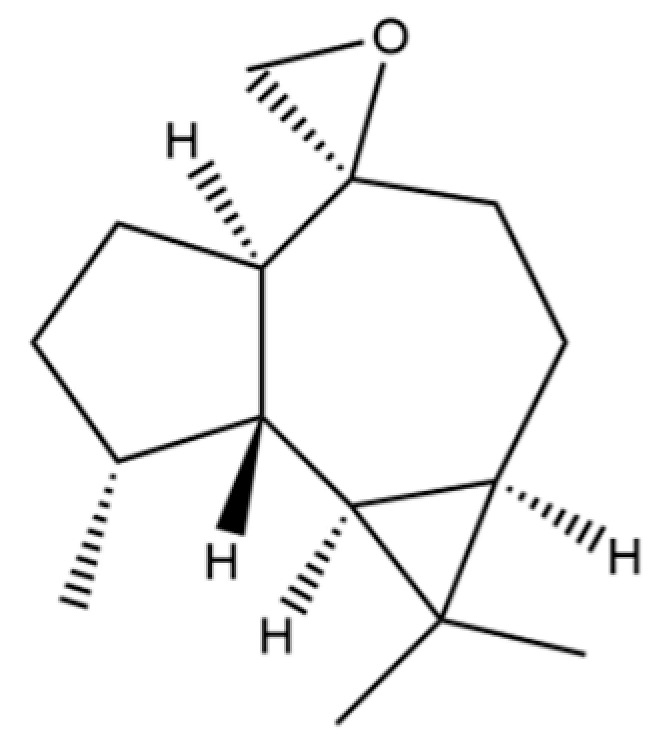	*	*	Sesquiterpenoid	[20]
81	Valerenol	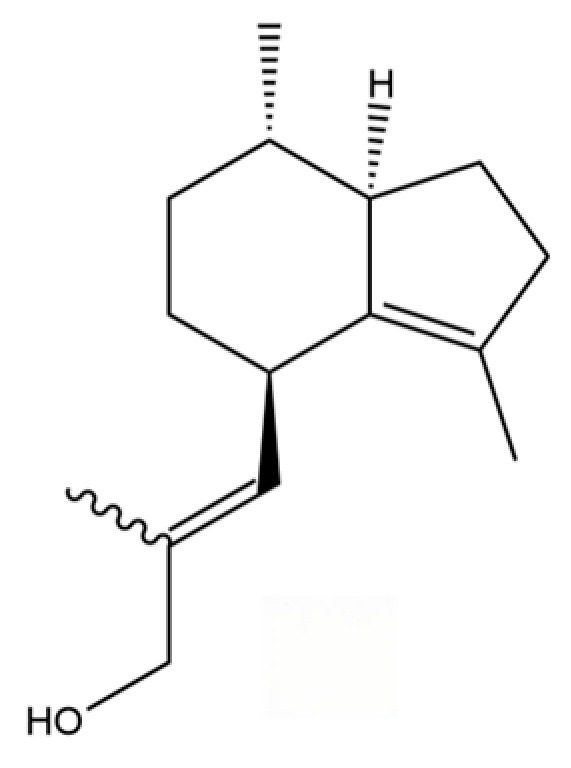	*	*	Sesquiterpenoid	[20]
82	3-Decenoic acid	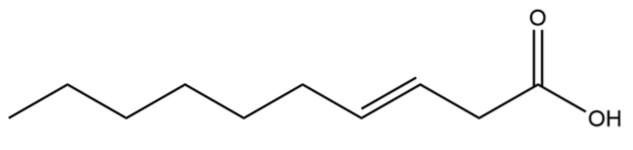	*	*	Fatty acid	[20]
83	Heneicosane	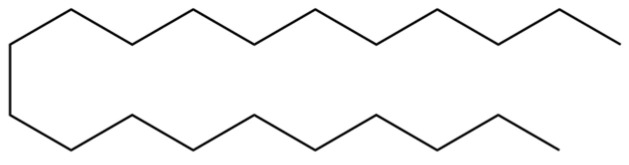	*	*	Alkane	[26]
84	Spathulel	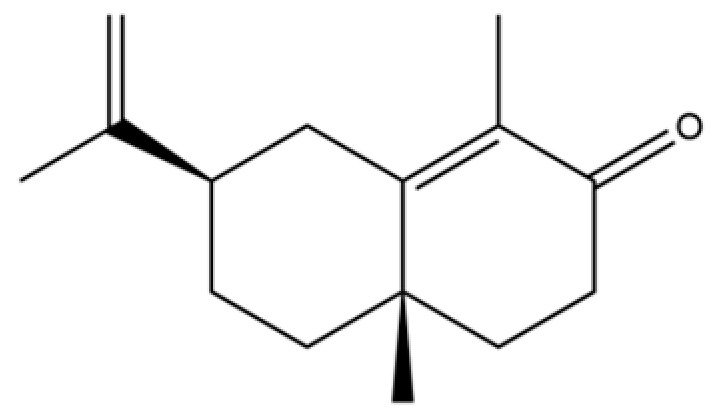	*	*	Sesquiterpenoid	[20]
85	Diethyl Phthalate	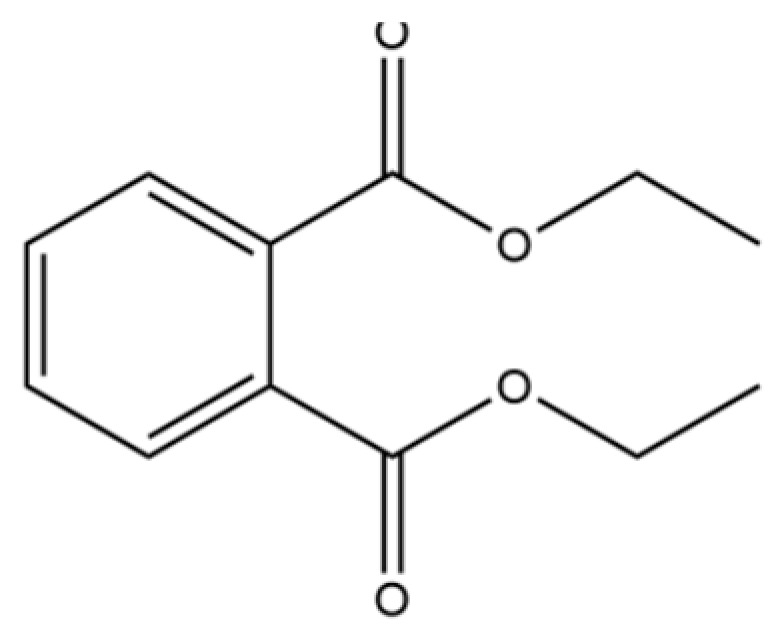	*	*	Phthalate ester (artifact)	[33]
86	1,1,4,7-Tetramethyldecahydro-1H-cyclopropa[e] azulene-4,7-diol	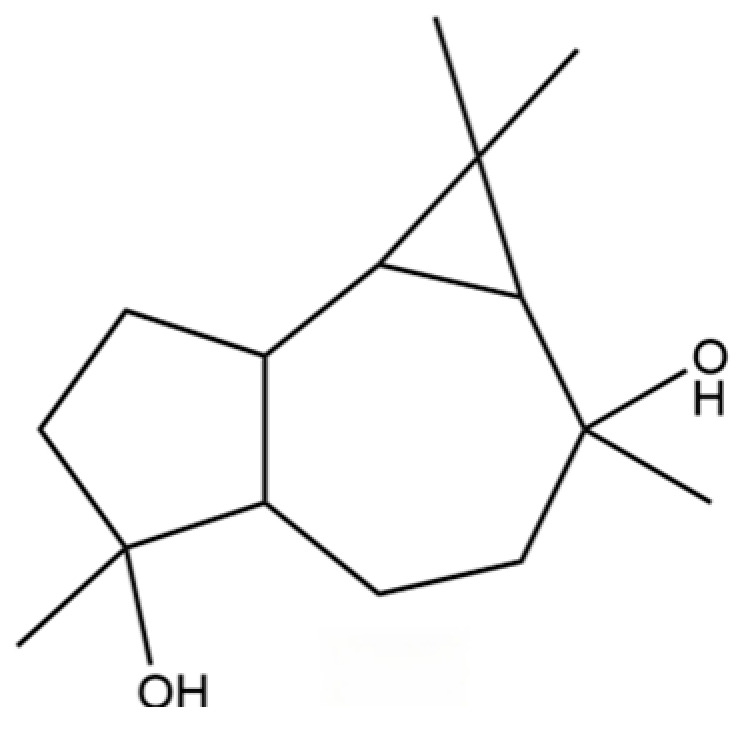	*	*	Sesquiterpenoid	[20]
87	α-Serinene	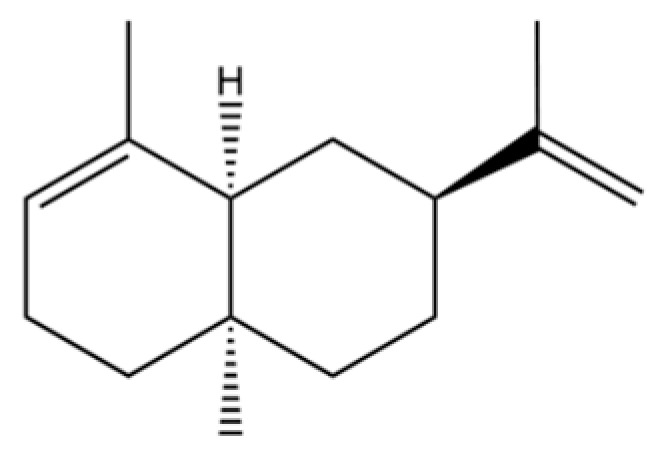	*	*	Sesquiterpenoid	[20]
88	Caryophyllene oxide	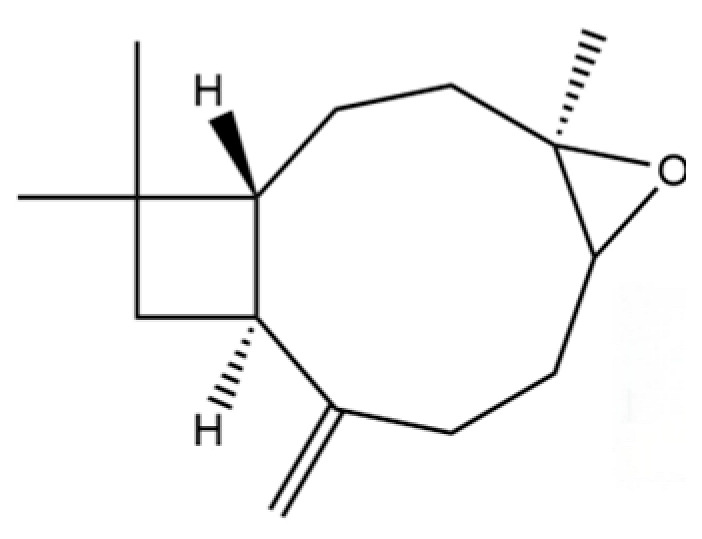	*	*	Sesquiterpenoid	[21]
89	trans-9-Hexadecen-1-ol	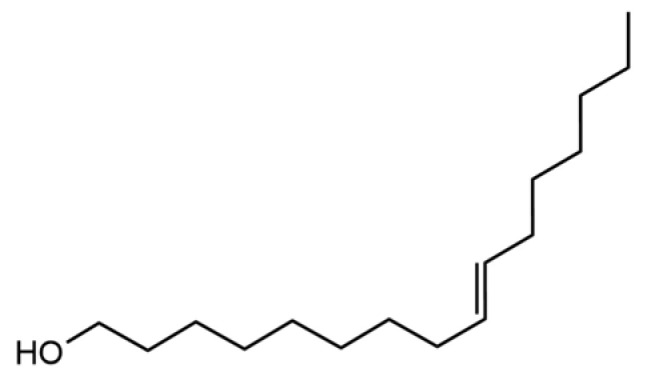	*	*	Fatty alcohol	[20]
90	Kaur-16-ene	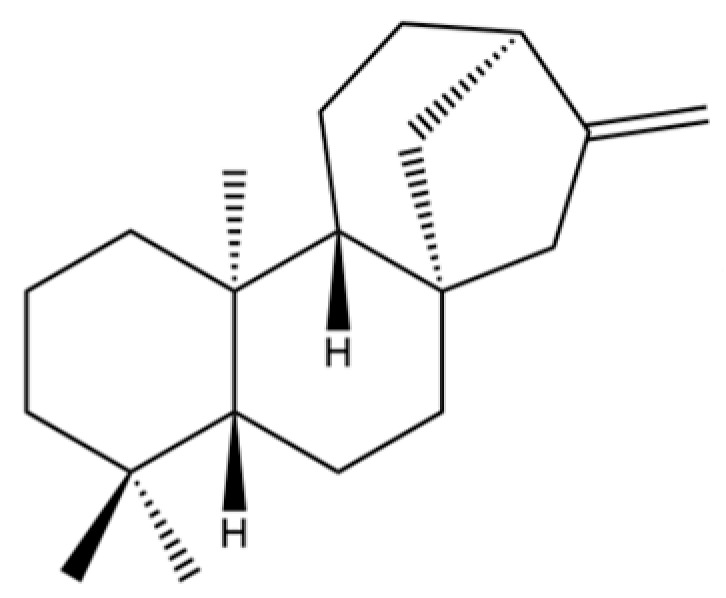	*	*	Diterpenoid	[20]
91	3-Phenyltoluene	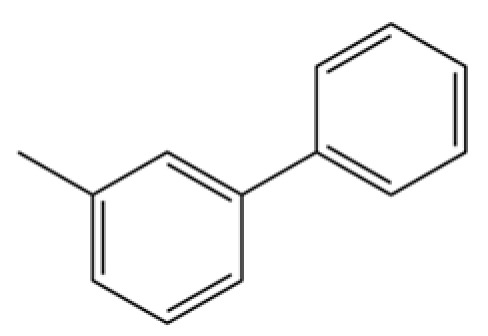	*	*	Aromatic hydrocarbon	[20]
92	4a, 7-Methano-4aH-naphth[1,8a-b] oxirene, octahydro-4,4,8,8-tetramethyl-	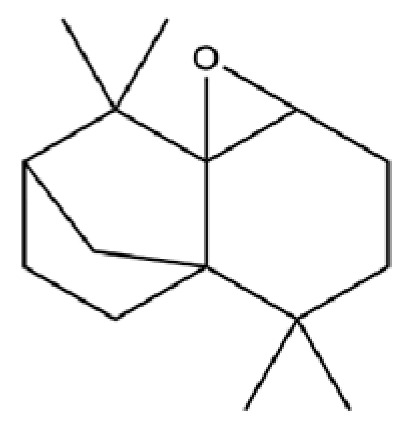	*	*	Sesquiterpenoid	[20]
93	(1R,7S, E)-7-Isopropyl-4,10-dimethylenecyclodec-5-enol	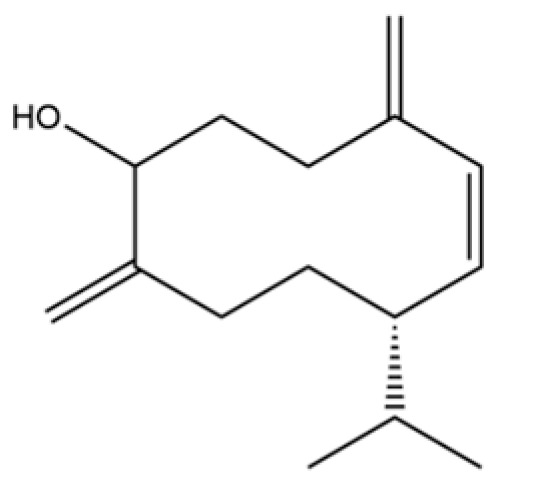	*	*	Sesquiterpenoid	[20]
94	Acetic acid n-octadecyl ester	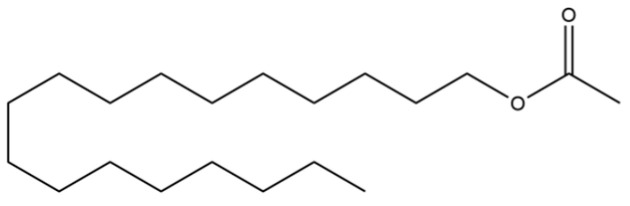	*	*	Fatty ester	[20]
95	Methyl 10-trans, 12-cis- octadecadienoate	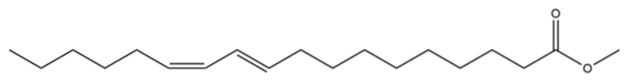	*	*	Fatty acid methyl ester	[20]
96	Oplopane	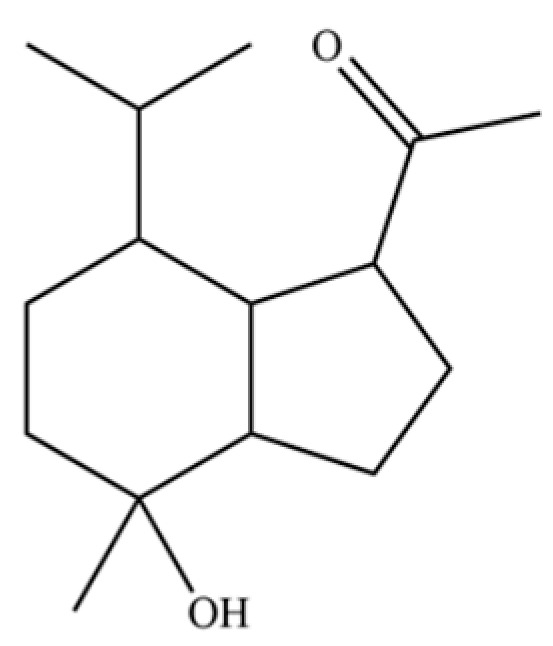	*	*	Sesquiterpenoid	[20]
97	Diisobutyl phthalate	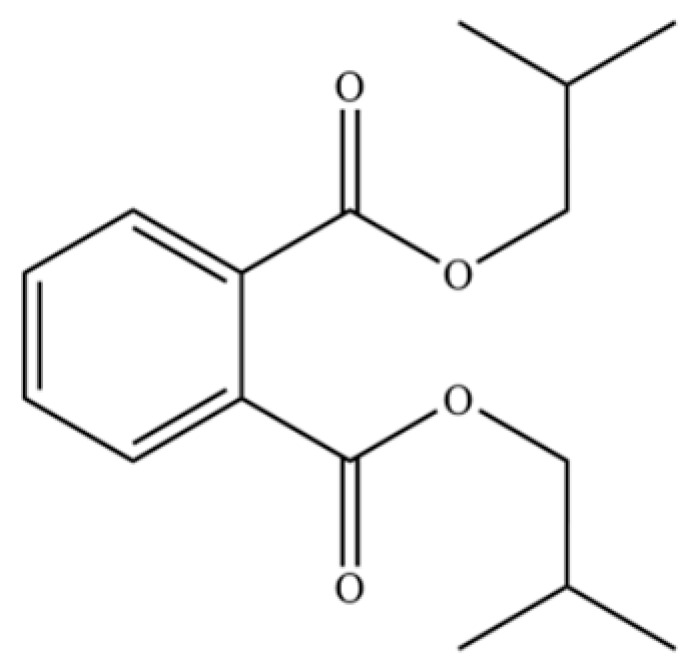	*	*	Phthalate ester (artifact)	[20]
98	2aS,3aR,5aS,9bR)-2a, 5a, 9-Trimethyl-2a, 4,5,5a,	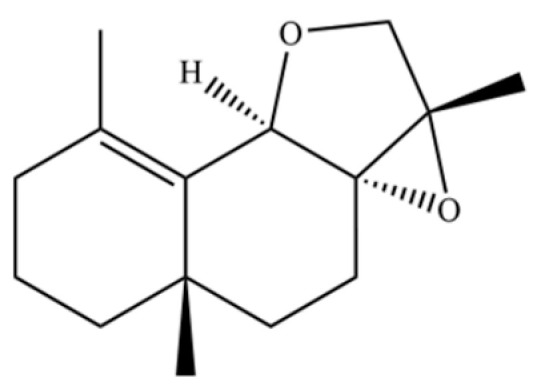	*	*	Sesquiterpenoid	[20]
99	Costol	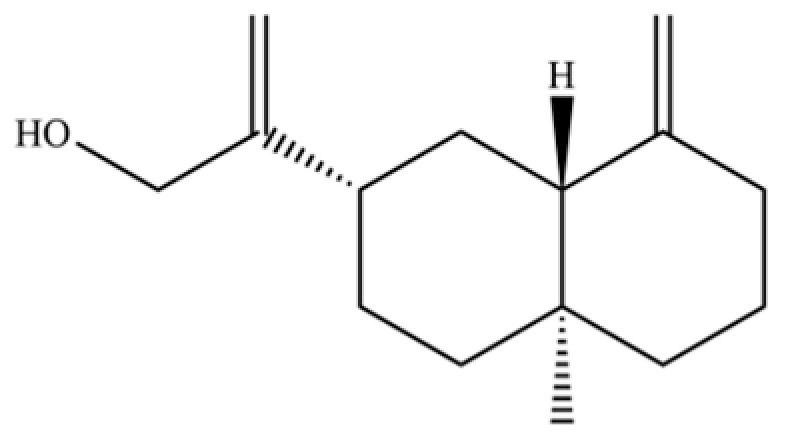	*	*	Sesquiterpenoid	[20]
100	Octacosane	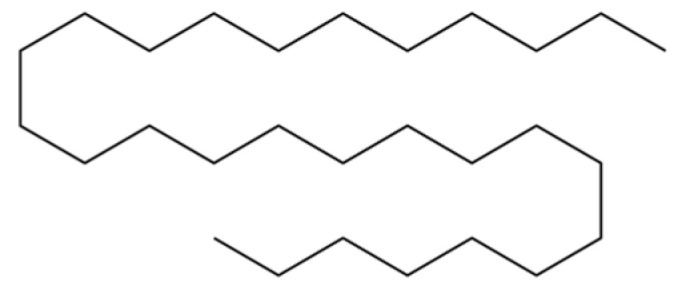	*	*	Alkane	[20]
101	1-Eicosanol	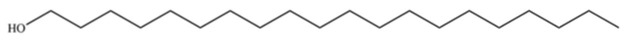	*	*	Fatty alcohol	[20]
102	Valerenyl isovalerate	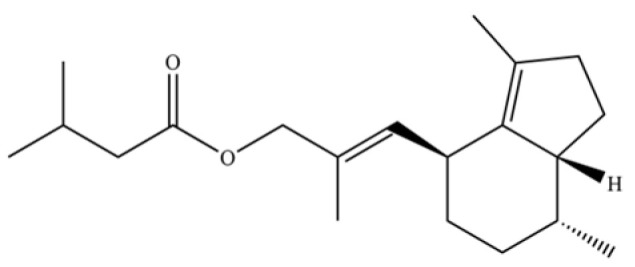	*	*	Sesquiterpenoid ester	[20]
103	longifolene-(V4)	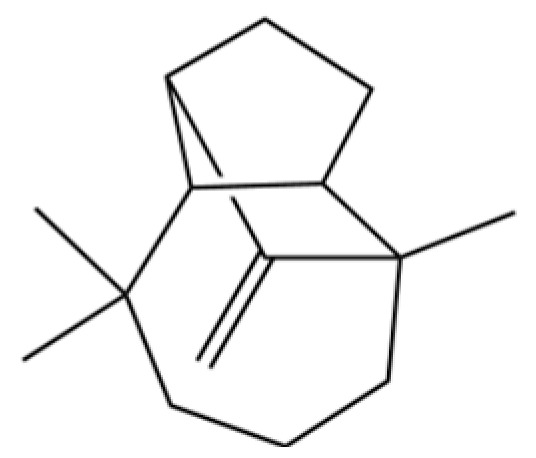	*	*	Sesquiterpenoid	[25]
104	Cryptomeridiol	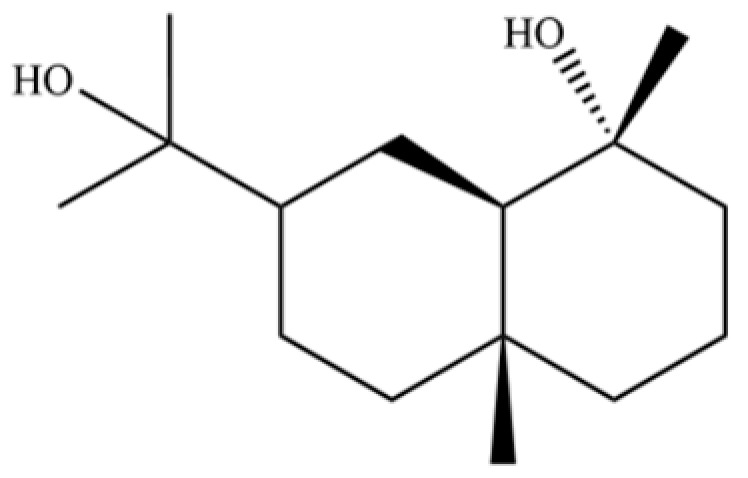	*	*	Sesquiterpenoid	[20]
105	Spathulenol	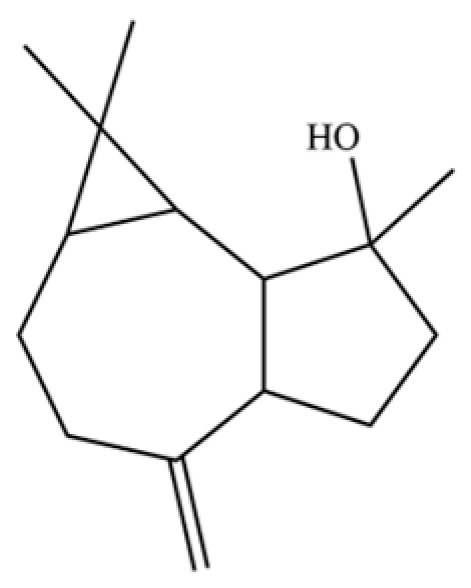	*	*	Sesquiterpenoid	[25]
106	Bicyclo[4.4.0]dec-5-ene, 1,5-dimethyl-3-hy-droxy-8-(1-methylene-2-hydroxyethyl-1)-	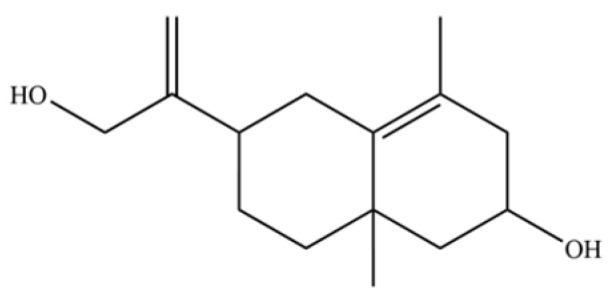	*	*	Sesquiterpenoid	[20]
107	6-Isopropenyl-4,8a-dimethyl-1,2,3,5,6,7,8,8a-octahydronaphthalene-2,3-diol	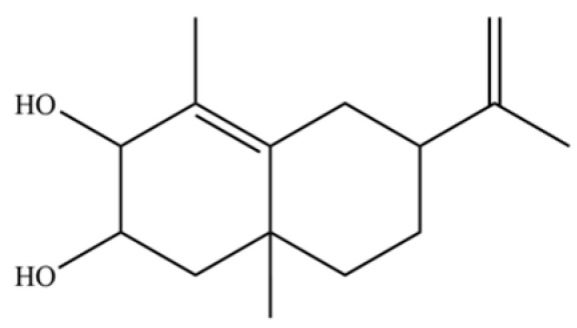	*	*	Sesquiterpenoid	[20]
108	n-Hexadecanoic acid	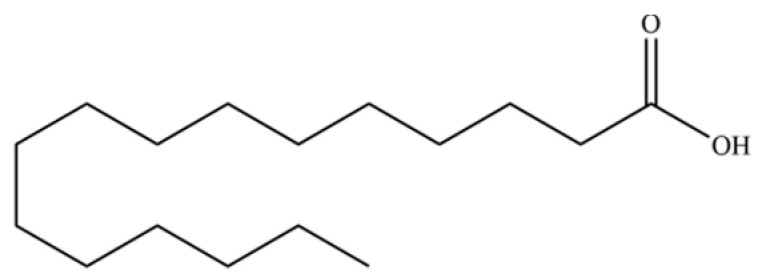	*	*	Fatty acid	[20]
109	Atractylolide	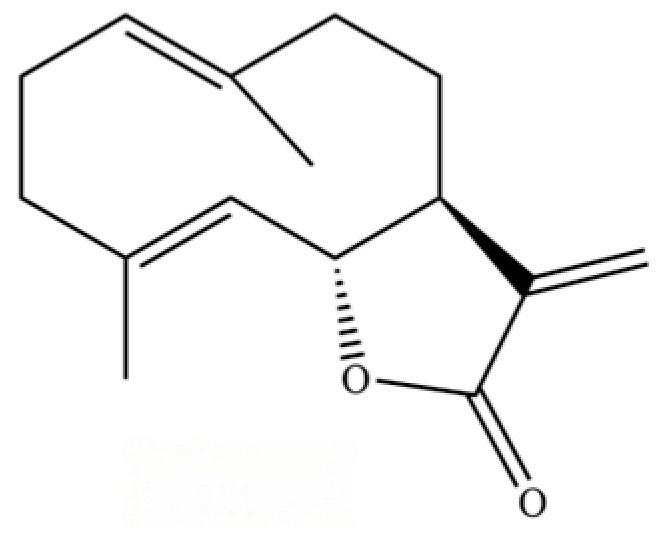	*	*	Sesquiterpenoid lactone	[34]
110	Tetratetracontane	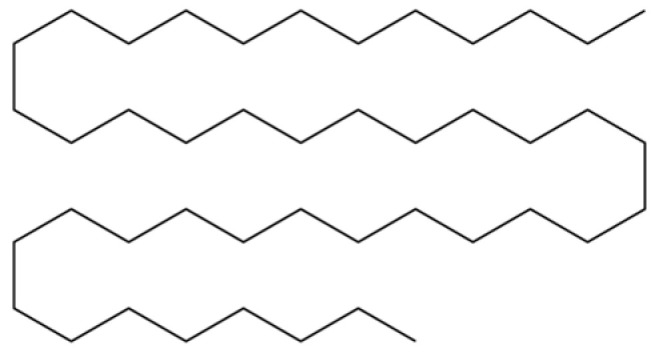	*	*	Alkane	[20]
111	α-Cyperone	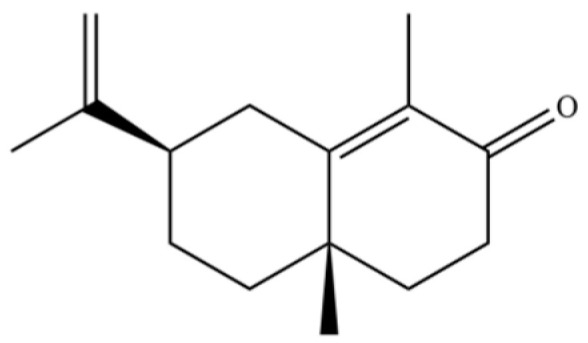	*	*	Sesquiterpenoid	[20]
112	Squalene	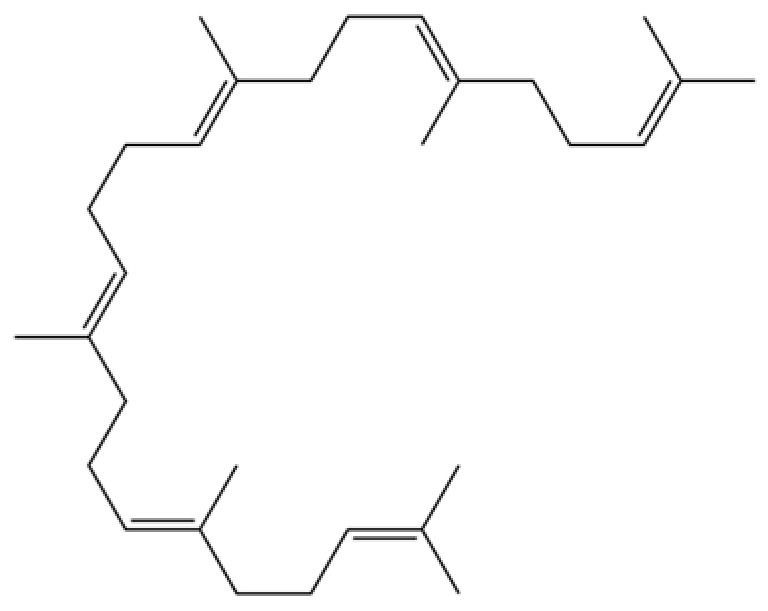	*	*	Triterpenoid	[35]
113	6-Octadecenoic acid	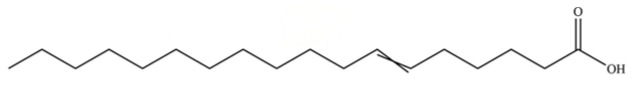	*	*	Fatty acid	[20]
114	Bicyclo[3.1.0]hexane, 4-methylene-1-(1-methylethyl)-	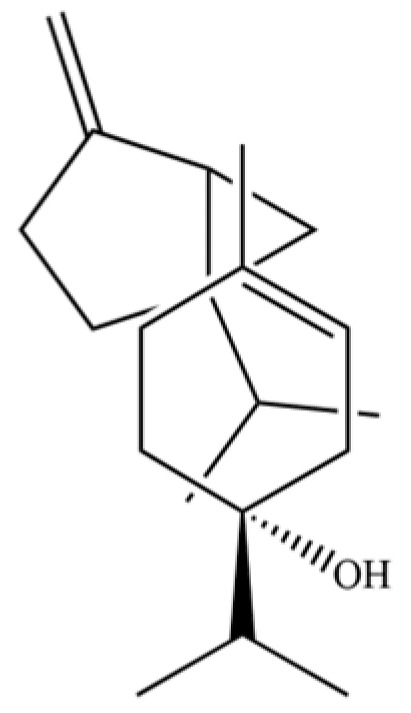	*	*	Monoterpenoid	[36]
115	Eucalyptol	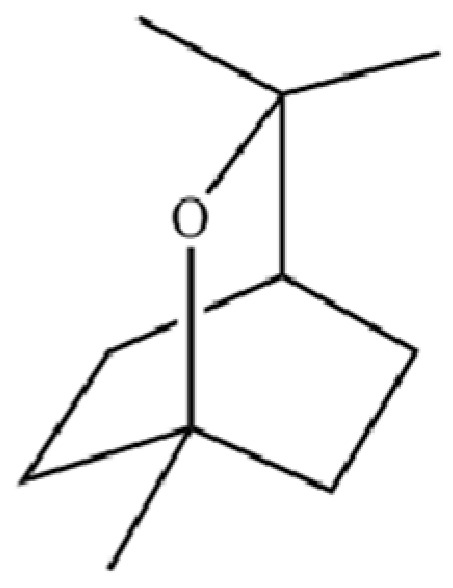	*	*	Monoterpenoid	[37]
116	γ-Terpinene	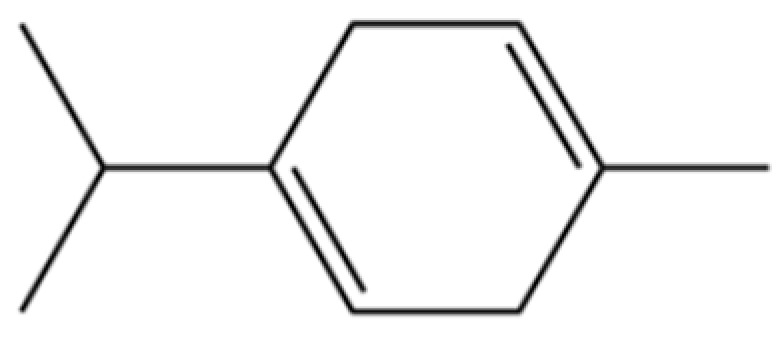	*	*	Monoterpenoid	[26]
117	Linalool	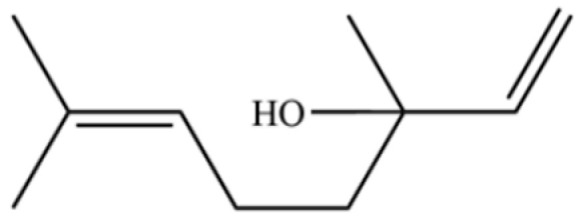	*	*	Monoterpenoid	[38]
118	1,5-Cyclodecadiene, 1,5-dimethyl-8-(1-methylethylidene)-, (E,E)-	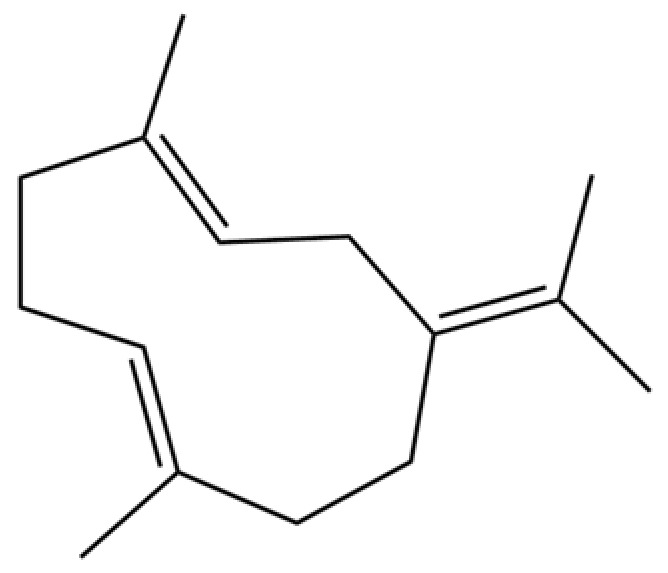	*	*	Sesquiterpenoid	[36]
119	β-Pinene	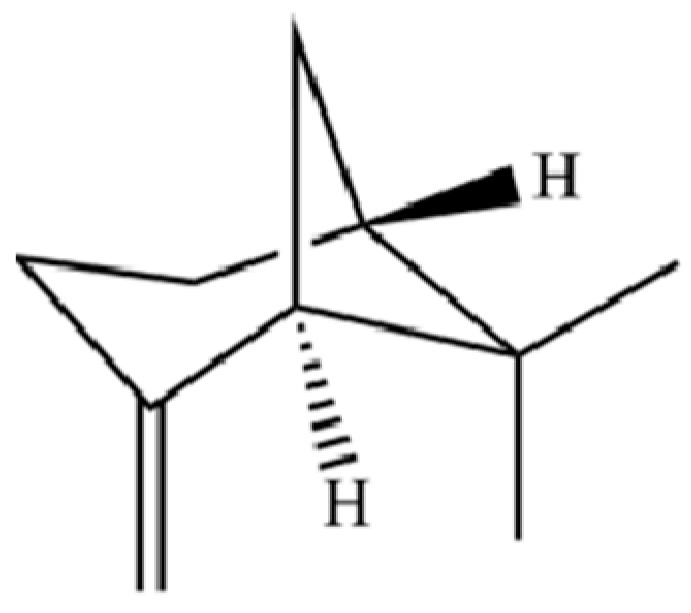	*	*	Monoterpenoid	[21]
120	β-Phellandrene	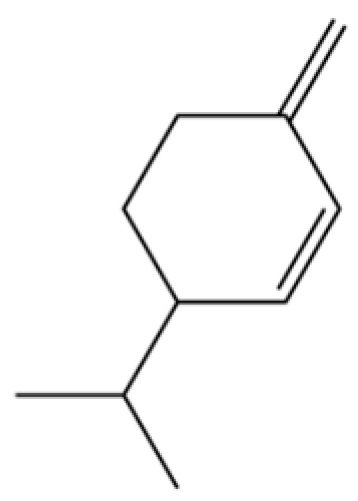	*	*	Monoterpenoid	[21]
121	Camphene	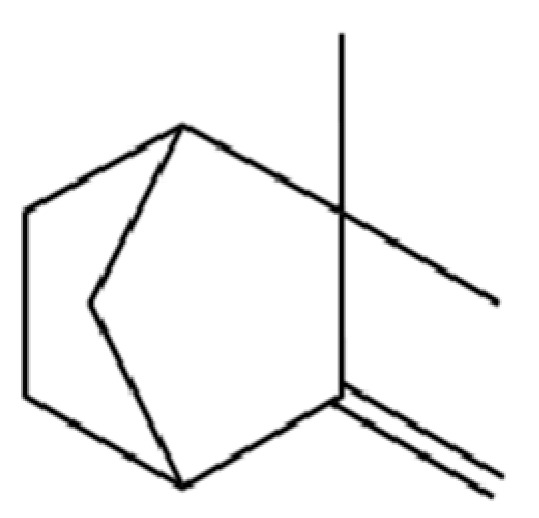	*	*	Monoterpenoid	[36]
122	β-Bisabolene	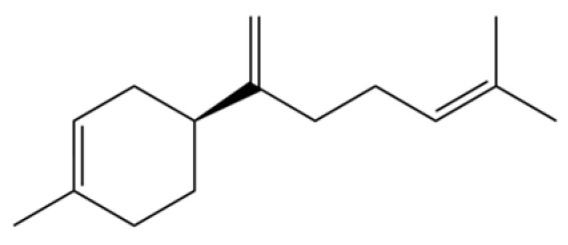	*	*	Sesquiterpenoid	[18]
123	Benzaldehyde	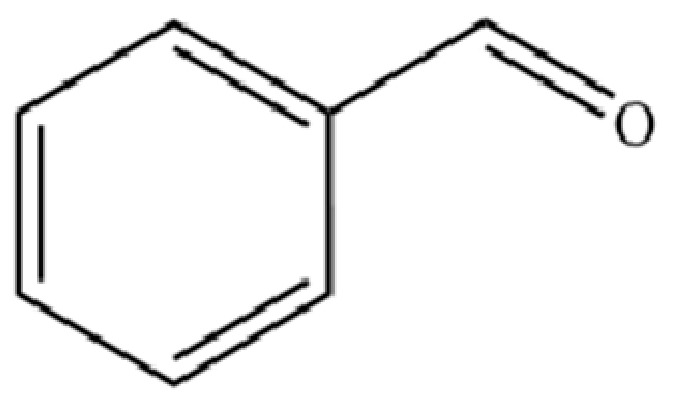	*	*	Aromatic aldehyde	[39]
124	(S)-(+)-alpha-Phellandrene	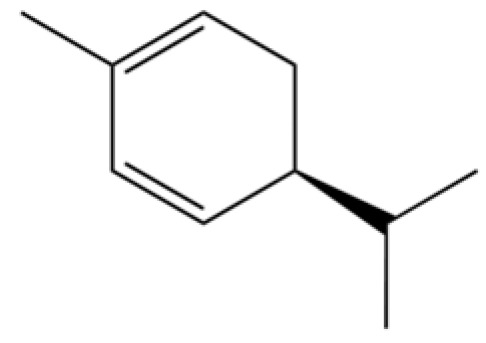	*	*	Monoterpenoid	[36]
125	Citronellyl acetate	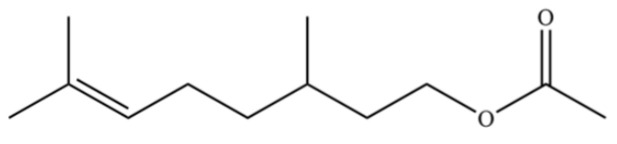	*	*	Monoterpenoid ester	[40]
126	(-)-Bornyl acetate	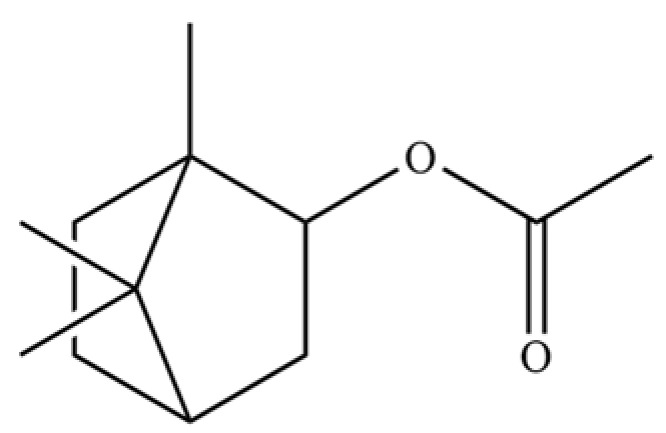	*	*	Monoterpenoid ester	[21]
127	Bicyclo[3.1.1]heptane, 6,6-dimethyl-2-methylene-, (1S)-	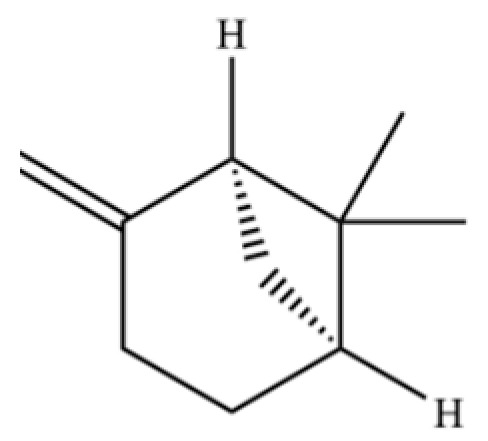	*	*	Monoterpenoid	[25]
128	Tricyclo[2.2.1.0(2,6)]heptane, 1,7,7-trimethyl-	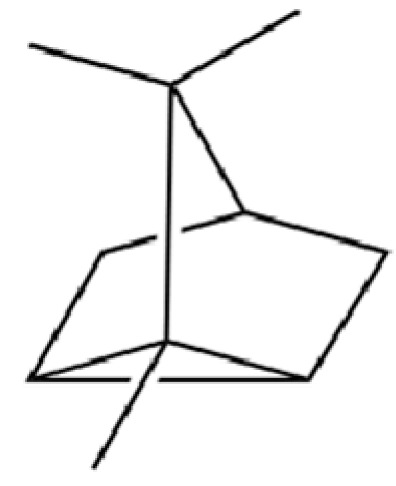	*	*	Monoterpenoid	[36]
129	Acetophenone, 4′-hydroxy-	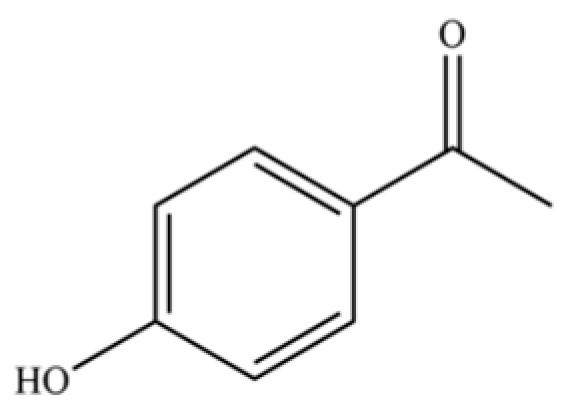	*	*	Phenolic ketone	[36]
130	(+)-2-Bornanone	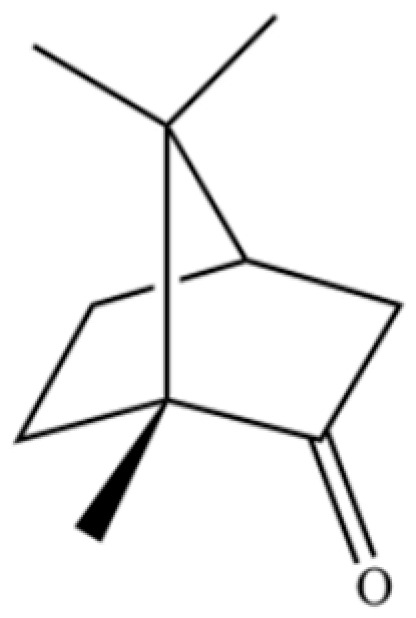	*	*	Monoterpenoid ketone	[36]
131	3-Cyclohexen-1-ol, 4-methyl-1-(1-methylethyl)-, (R)-	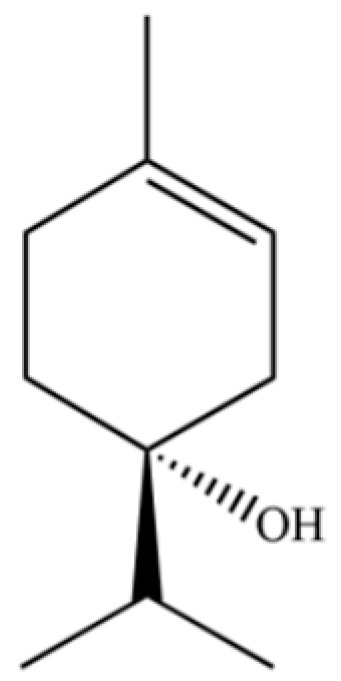	*	*	Monoterpenoid	[36]
132	2(1H)-Pyridinone	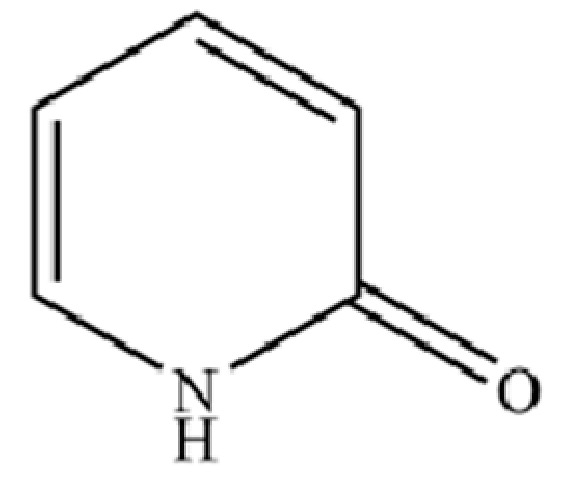	*	*	PHeterocyclic compound	[36]
133	Acetic acid, 4-methylphenyl ester	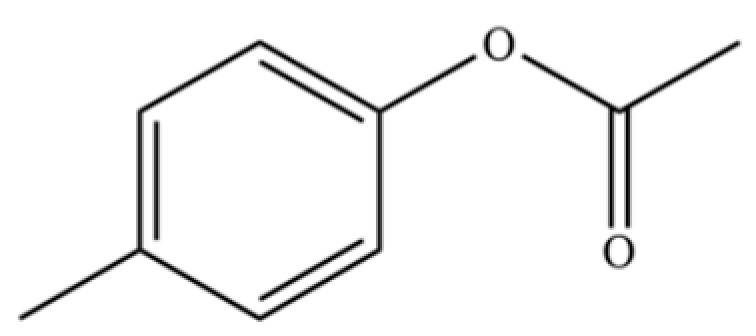	*	*	Phenolic ester	[36]
134	L-α-Terpineol	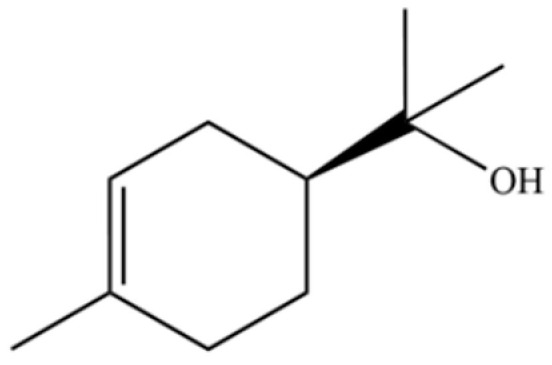	*	*	Monoterpenoid	[36]
135	3-Methyl-4-isopropylphenol	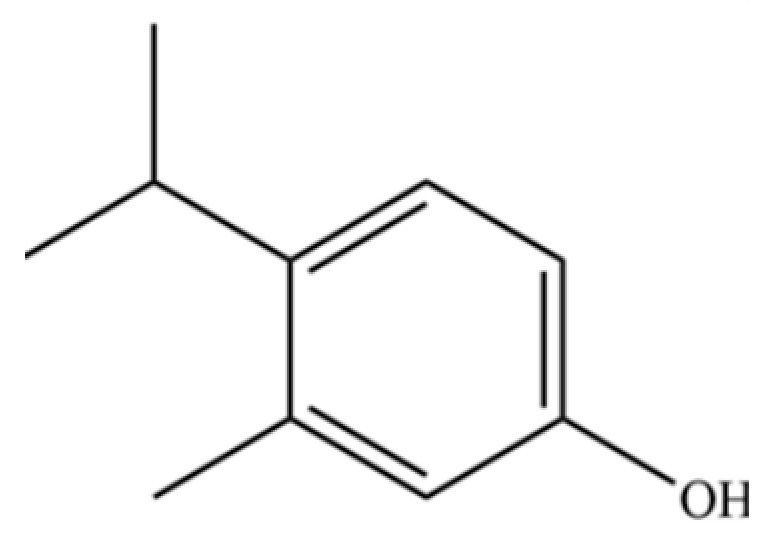	*	*	Phenolic	[41]
136	Isoelemicin	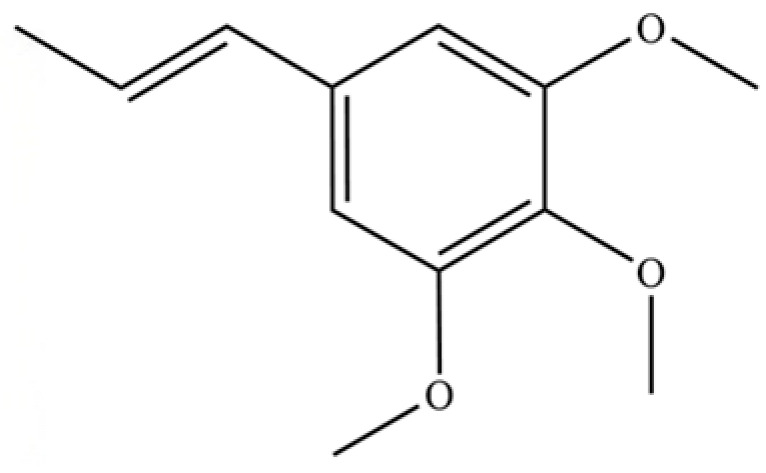	*	*	Phenylpropanoid	[36]
137	Furanodienone	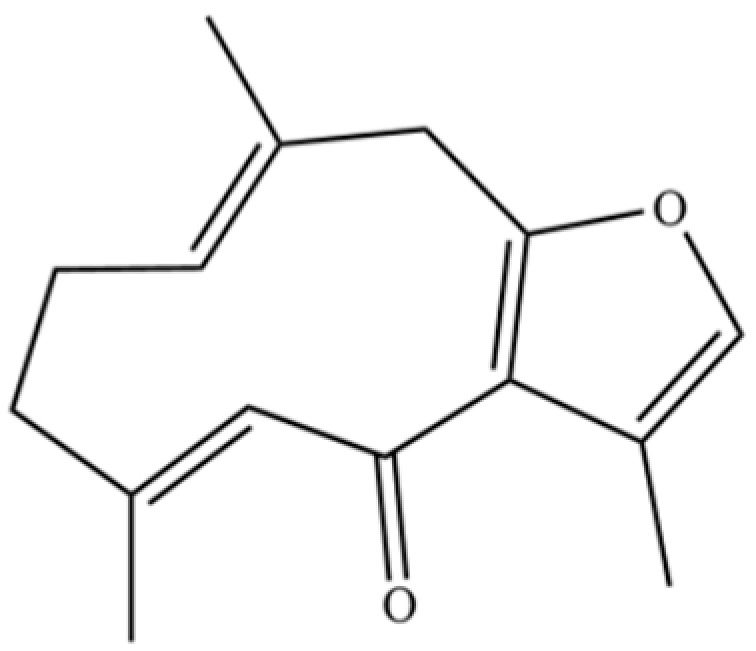	*	*	Sesquiterpenoid	[36]
138	(1S)-2,6,6-Trimethylbicyclo[3.1.1]hept-2-ene	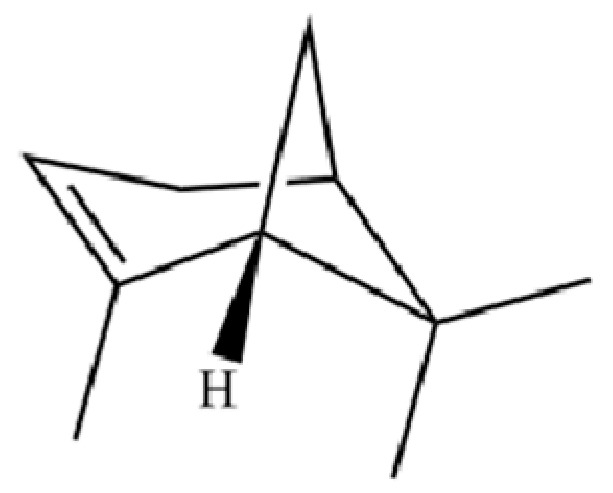	*	*	Monoterpenoid	[36]
139	Ethanone, 1-(2,4,6-trihydroxyphenyl)-	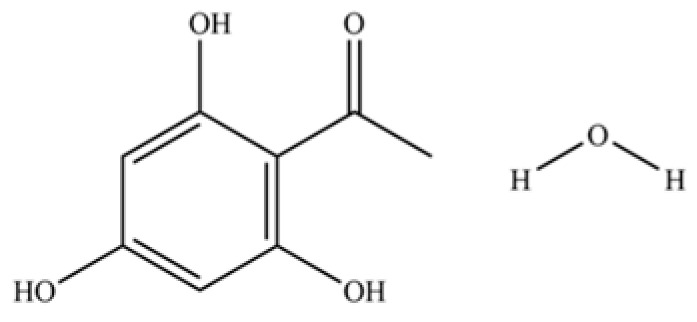	*	*	Phenolic ketone	[36]
140	Phenol, 2,3,6-trimethyl-	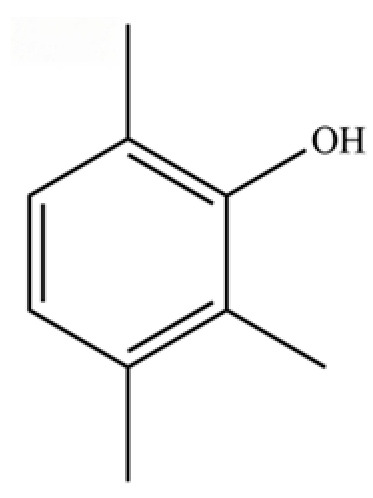	*	*	Phenolic	[36]
141	Atractylodin	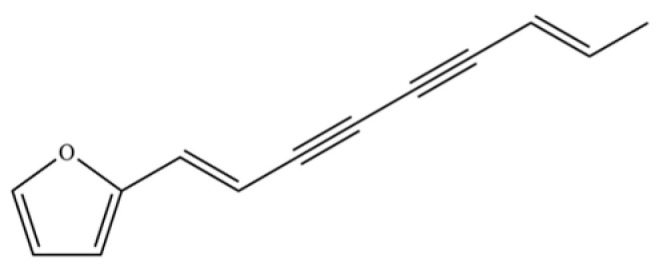	**	*	Polyacetylene	[21]
142	3-Methyl-3-buten-1-ol, acetate	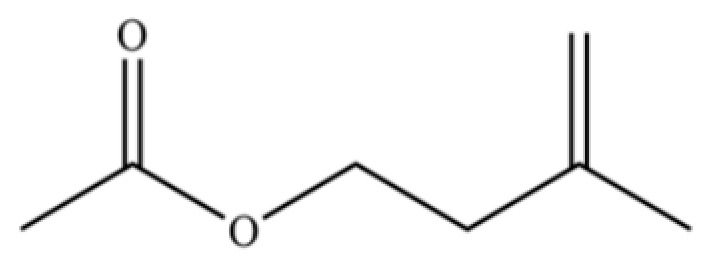	*	*	Terpene alcohol ester	[36]
143	α-Thujene	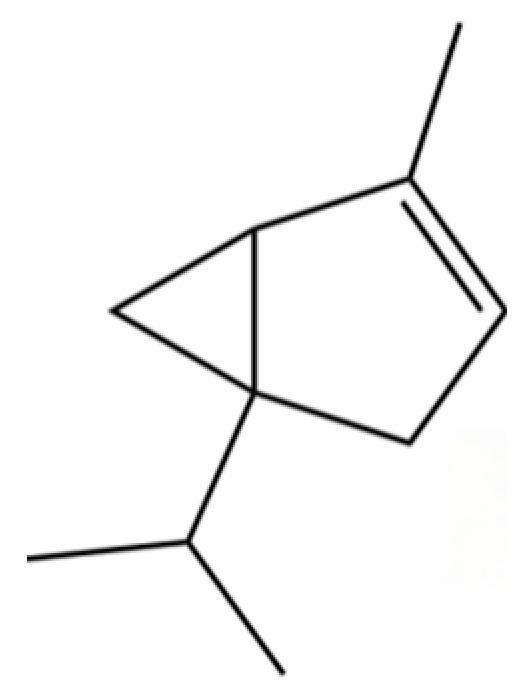	*	*	Monoterpenoid	[42]
144	3-Carene	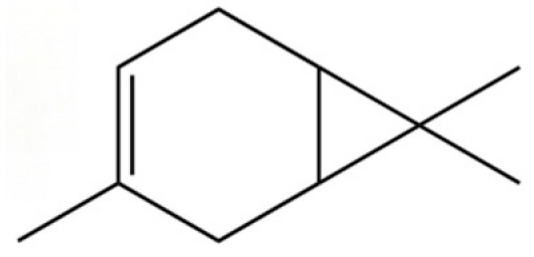	*	*	Monoterpenoid	[21]
145	1,3,8-p-Menthatriene	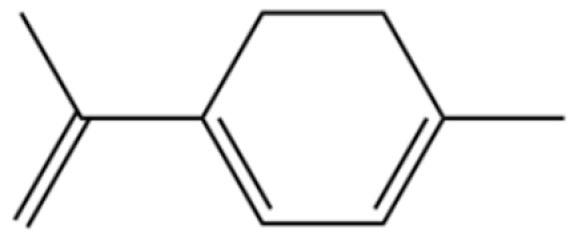	*	*	Monoterpenoid	[36]
146	Carveol	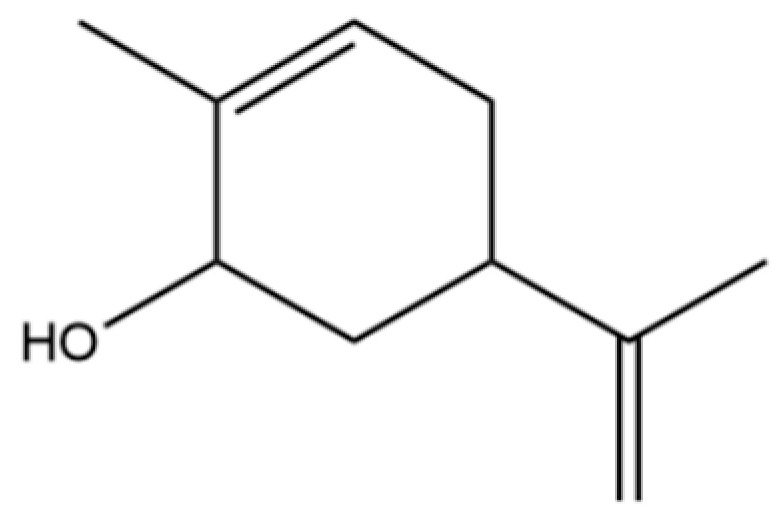	*	*	Monoterpenoid	[36]
147	Dill ether	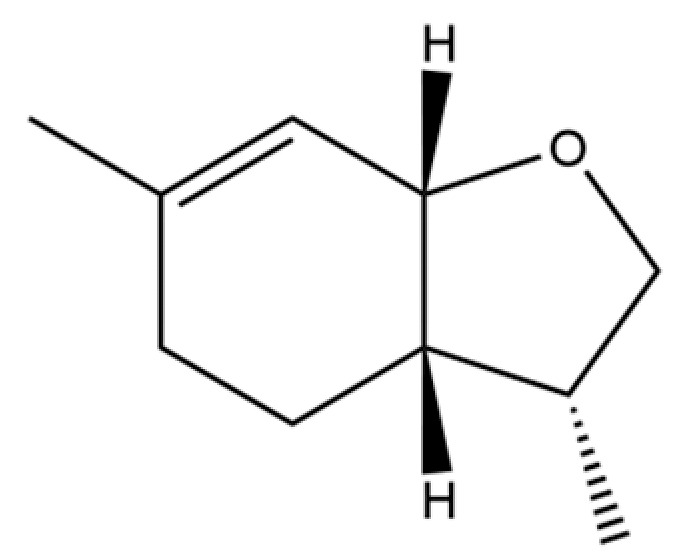	*	*	Monoterpenoid ether	[36]
148	trans-Geranic acid methyl ester	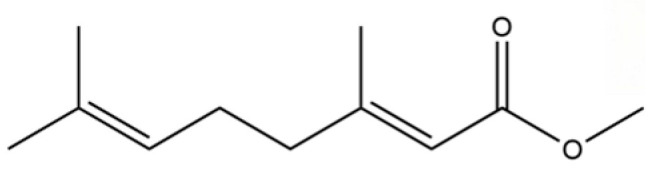	*	*	Monoterpenoid ester	[36]
149	3a,7-Methano-3aH-cyclopentacyclooctene, 1,4,5,6,7,8,9,9a-octahydro-1,1,7-trimethyl-, [3aR-(3a.α,7.α,9a.β)]-	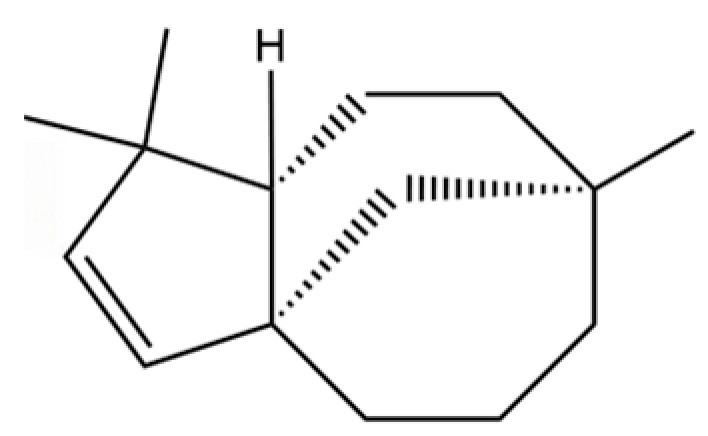	*	*	Sesquiterpenoid	[36]
150	2,6-Octadien-1-ol, 3,7-dimethyl-, acetate, (Z)-	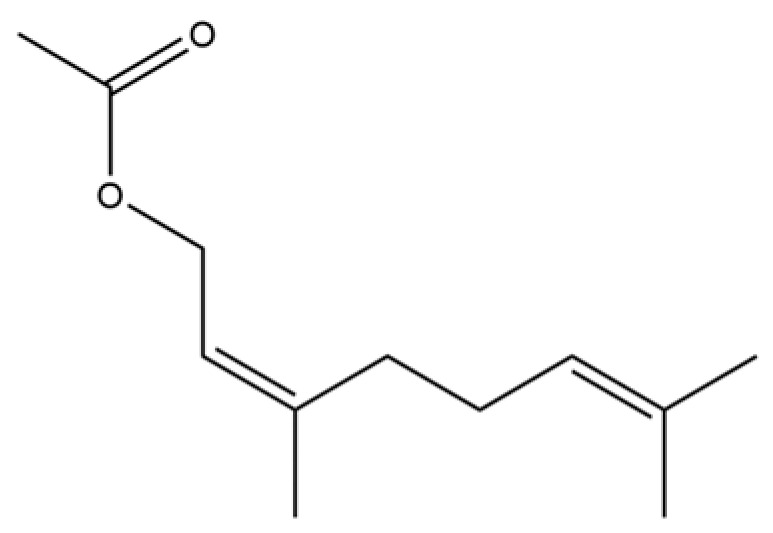	*	*	Monoterpenoid ester	[36]
151	Acetic acid, decyl ester	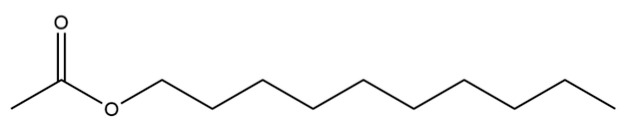	*	*	Fatty ester	[36]
152	(+)-4-Carene	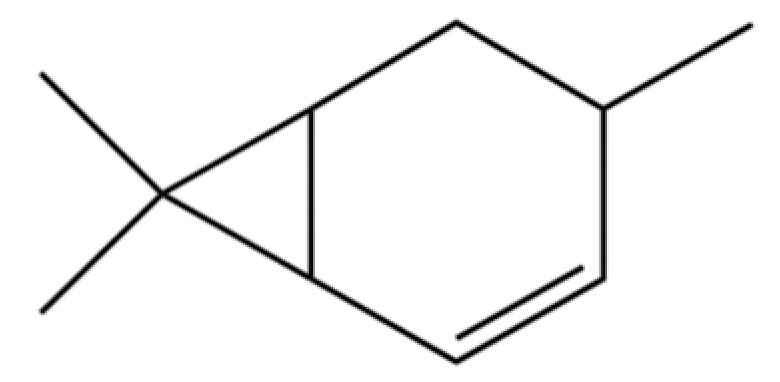	*	*	Monoterpenoid	[25]
153	γ-Muurolene	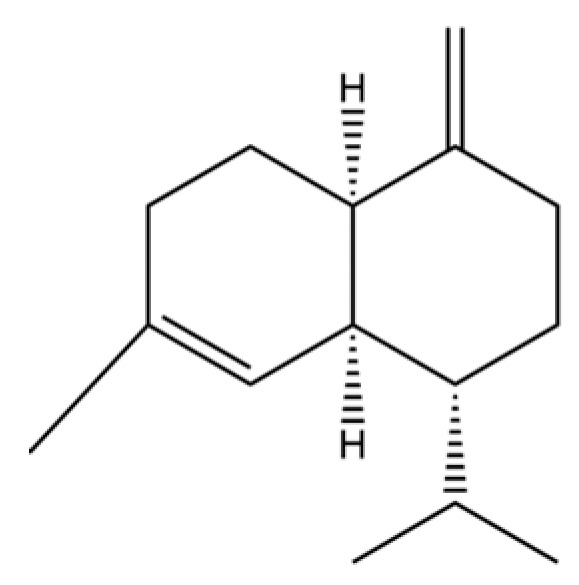	*	*	Sesquiterpenoid	[43]
154	(3aR,4R,7R)-1,4,9,9-Tetramethyl-3,4,5,6,7,8-hexahydro-2H-3a,7-methanoazulen-2-one	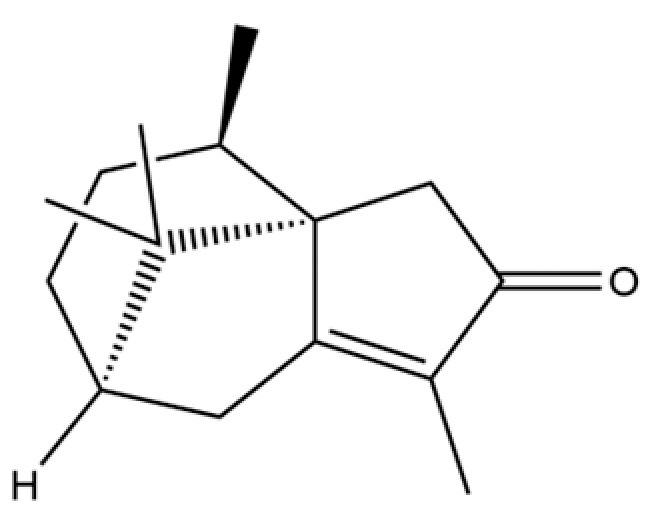	*	*	Sesquiterpenoid	[36]
155	Benzimidazo[2,1-a]isoquinoline	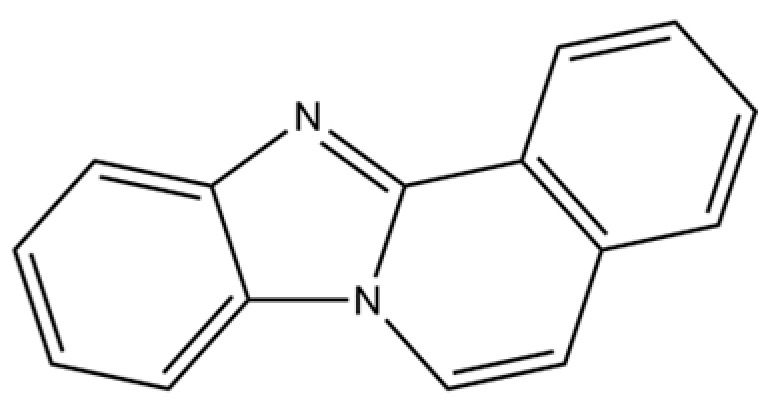	*	*	Heterocyclic compound	[36]
156	(4aR,8aR)-5,8a-Dimethyl-3-propan-2-ylidene-1,2,4,4a,7,8-hexahydronaphthalene	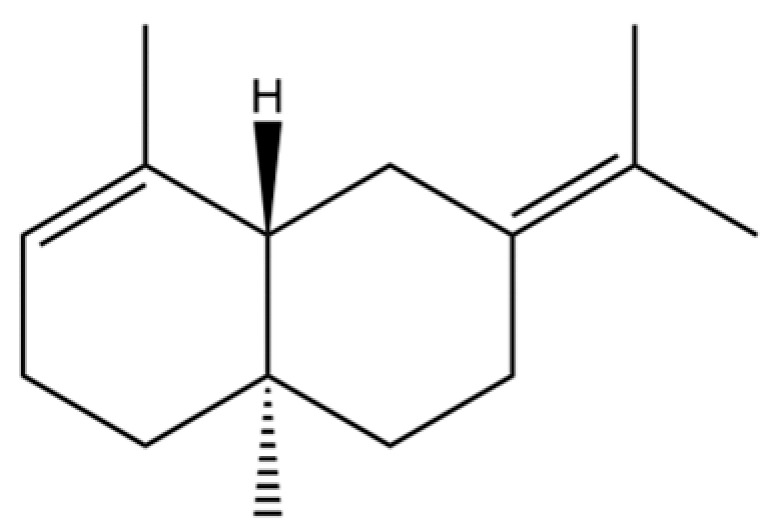	*	*	Sesquiterpenoid	[36]
157	(1R,3aS,8aS)-7-Isopropyl-1,4-dimethyl-1,2,3,3a,6,8a-hexahydroazulene	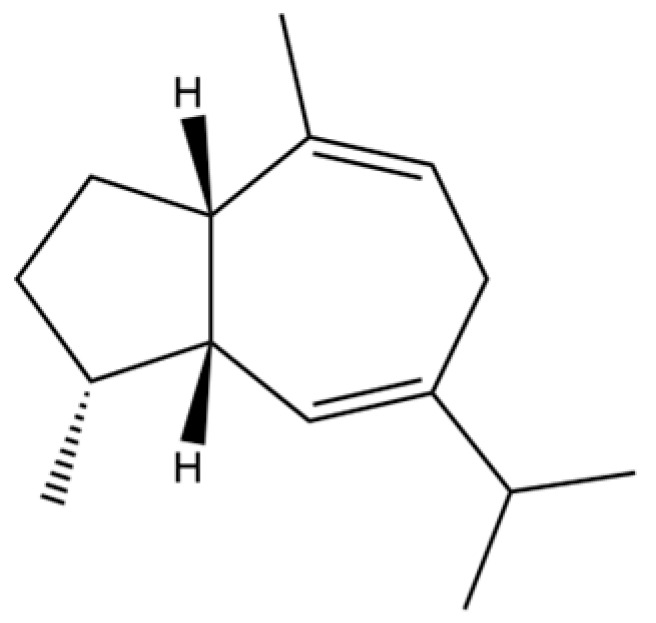	*	*	Sesquiterpenoid	[36]
158	Thujopsene	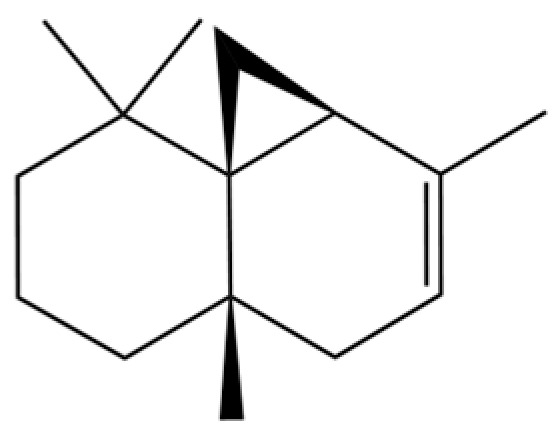	*	*	Sesquiterpenoid	[25]
159	p-Mentha-1,5,8-triene	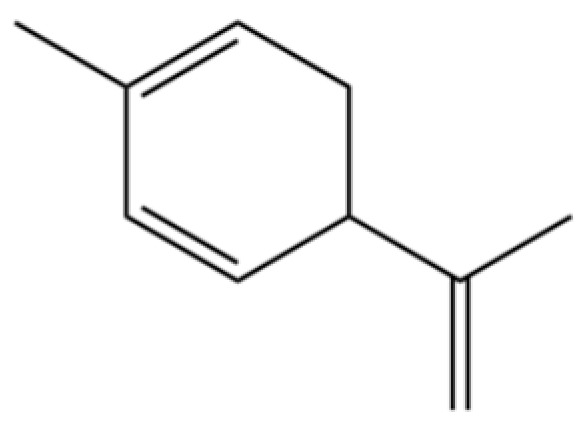	*	*	Monoterpenoid	[36]
160	1-Hexen, 2-(p-anisyl)-5-methyl-	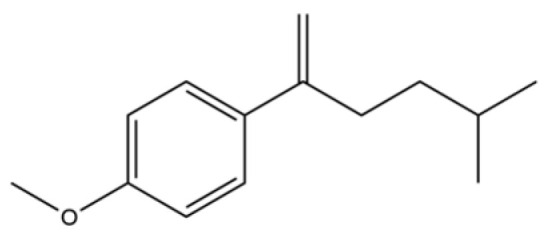	*	*	Aromatic hydrocarbon	[36]
161	6,7-Dimethyl-1,2,3,5,8,8a-hexahydronaphthalene	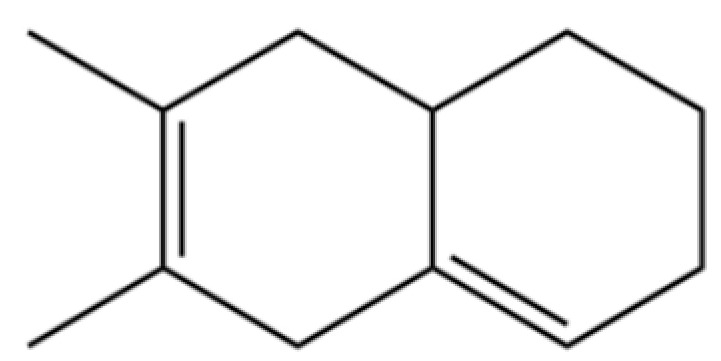	*	*	Sesquiterpenoid	[36]
162	2,3-Dimethoxy-5-aminocinnamonitrile	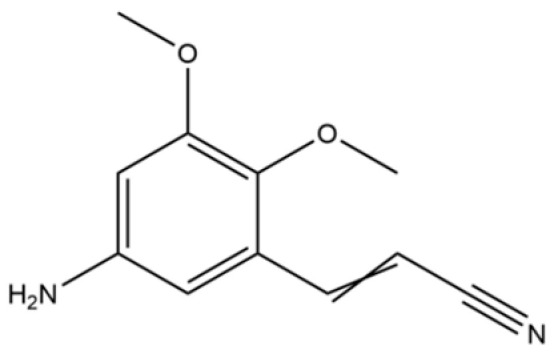	*	*	Aromatic nitrile	[36]
163	1-Penten-3-one, 1-(4-methoxyphenyl)-4-methyl-	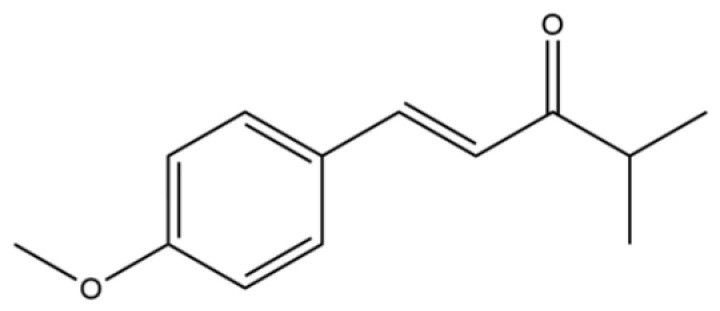	*	*	Aromatic ketone	[36]
164	4a(2H)-Naphthalenol, 1,3,4,5,6,8a-Hexahydro-4,7-dimethyl-1-(1-methylethyl)-, (1S,4S,4aS,8aR)-	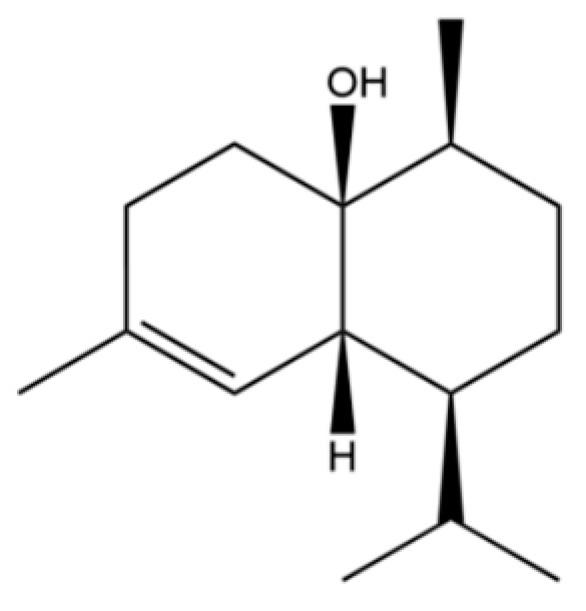	*	*	Sesquiterpenoid	[36]
165	Neoisolongifolene, 8,9-dehydro-	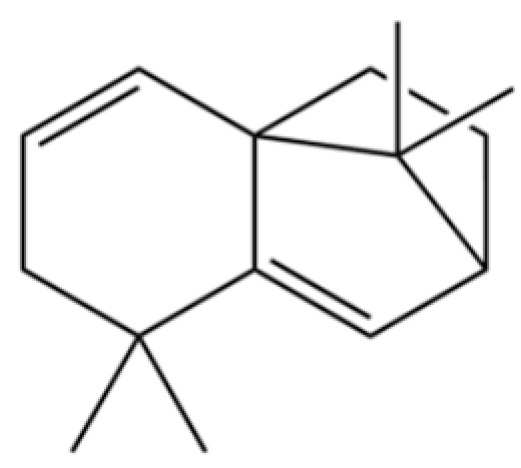	*	*	Sesquiterpenoid	[36]
166	6-Isopropenyl-4,8a-dimethyl-1,2,3,5,6,7,8,8a-octahydro-2-naphthalenyl acetate	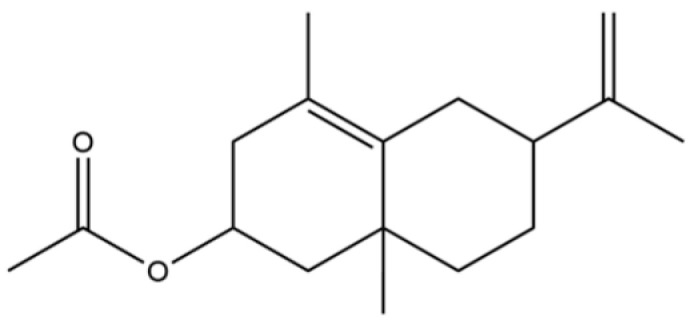	*	*	Sesquiterpenoid ester	[36]
167	Bicyclo[3.1.0]hex-2-ene, 4-methylene-1-(1-methylethyl)-	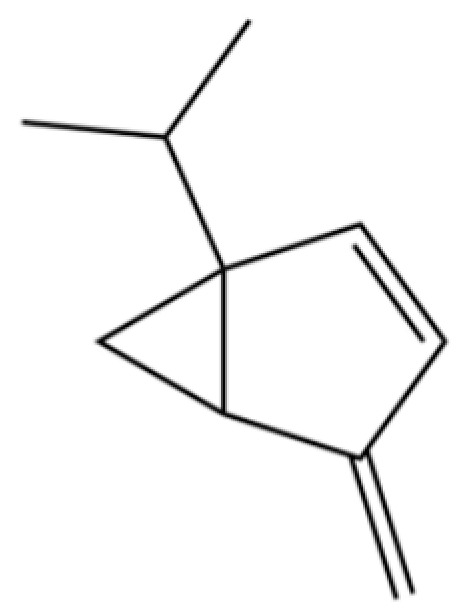	*	*	Monoterpenoid	[36]
168	Benzene, tert-butyl-	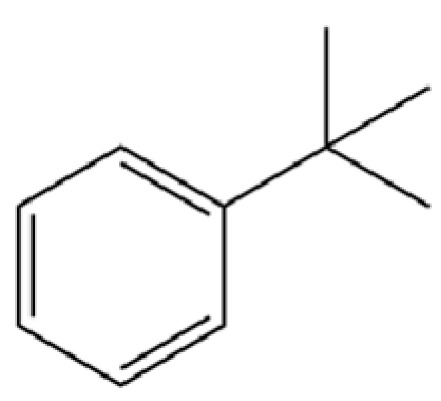	*	*	Aromatic hydrocarbon	[36]
169	1-Decen-3-one	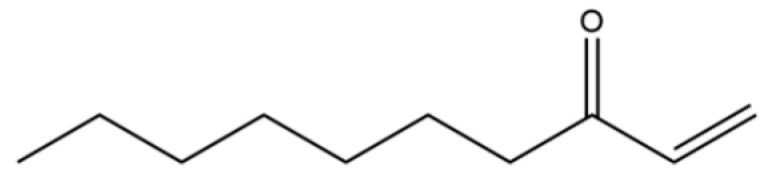	*	*	Ketone	[36]
170	Silphiperfol-5-ene	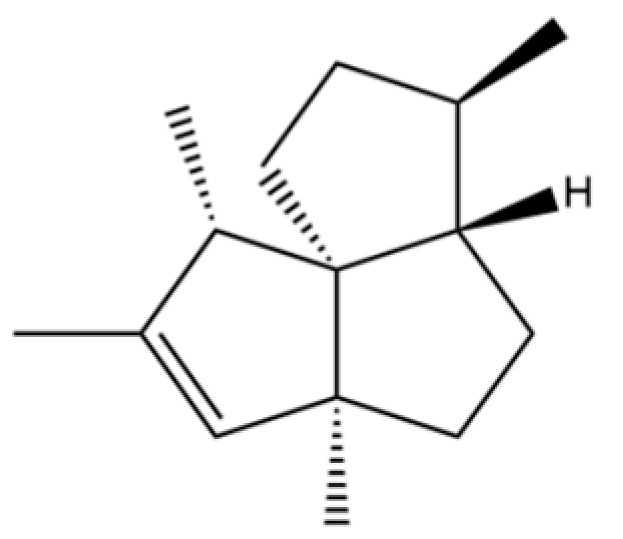	*	*	Sesquiterpenoid	[22]
171	1,1′-Biphenyl, 2,4,6-trimethyl-	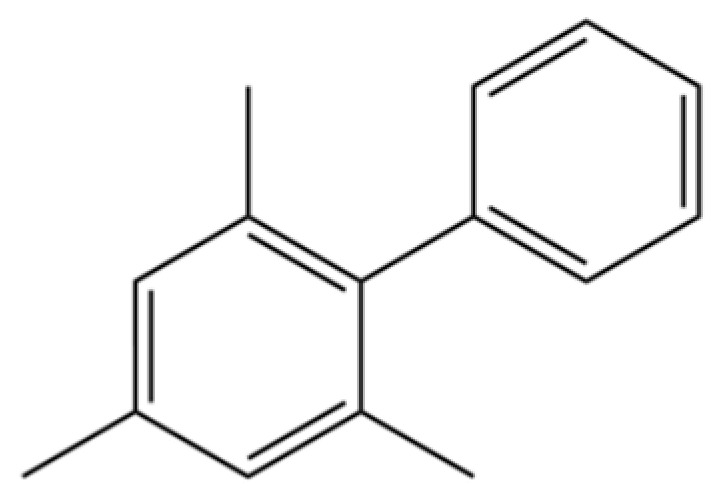	*	*	Aromatic hydrocarbon	[36]
172	4-Methylurazole	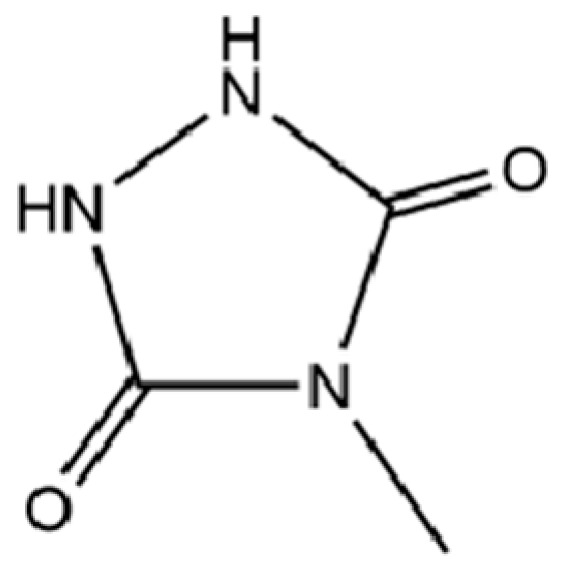	*	*	Heterocycle	[36]
173	(1R,3aS,8aS)-1,4,4,6-Tetramethyl-1,2,3,3a,4,5,7,8-octahydrocyclopenta[c]pentalene	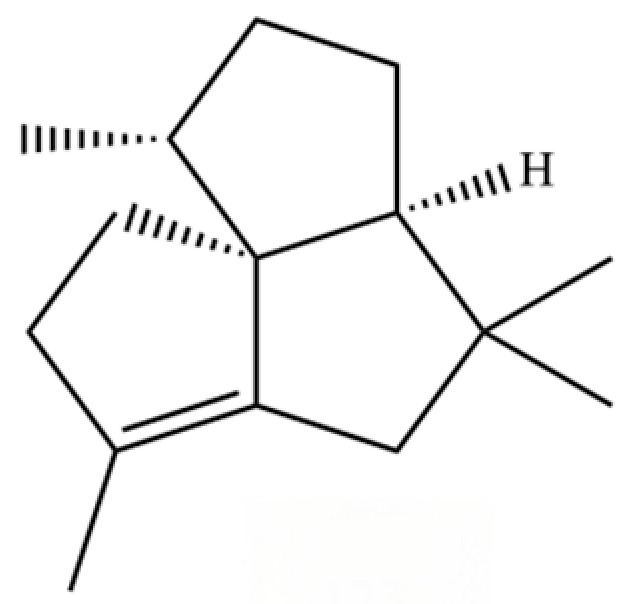	*	*	Sesquiterpenoid	[36]
174	1,3a,4,5a-Tetramethyl-1,2,3,3a,5a,6,7,8-octahydrocyclopenta[c]pentalene	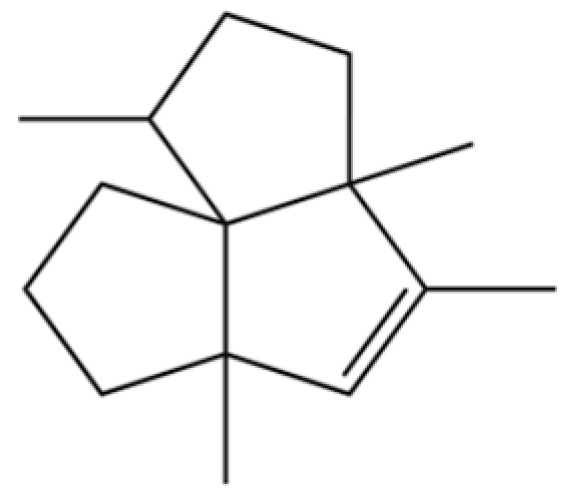	*	*	Sesquiterpenoid	[36]
175	9H-Fluorene, 1,9-dimethyl-	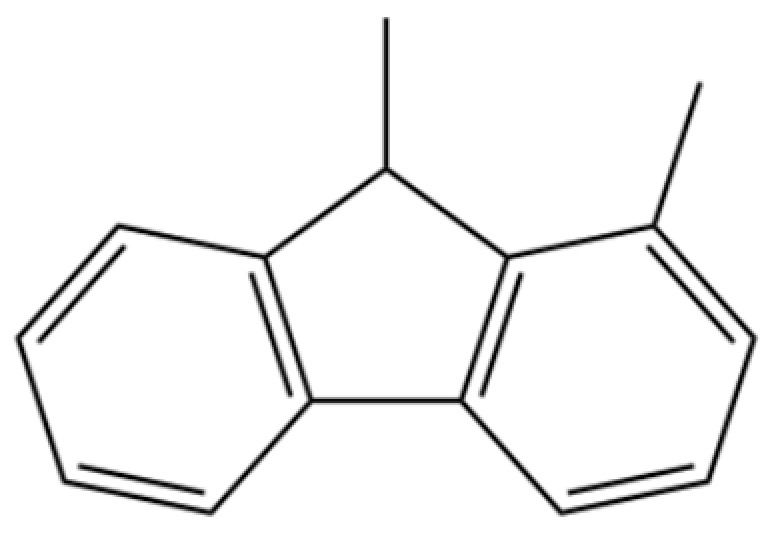	*	*	Aromatic hydrocarbon	[36]
176	N-Benzyloxy-2-carbomethoxyaziridine	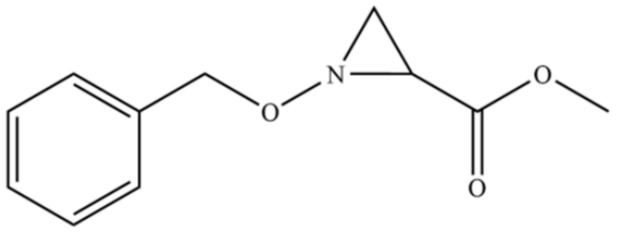	*	*	Heterocycle	[36]
177	Naphthalene, 2,3,6-trimethyl-	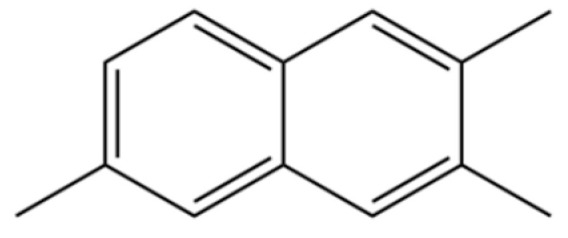	*	*	Aromatic hydrocarbon	[36]
178	2′-Ethoxyacetophenone	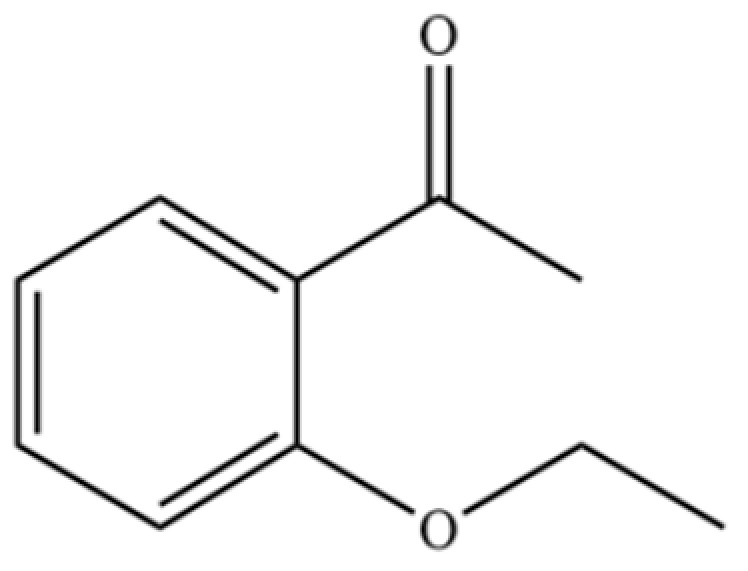	*	*	Aromatic ketone	[36]
179	Isobutyric acid, 2-pinen-10-yl ester	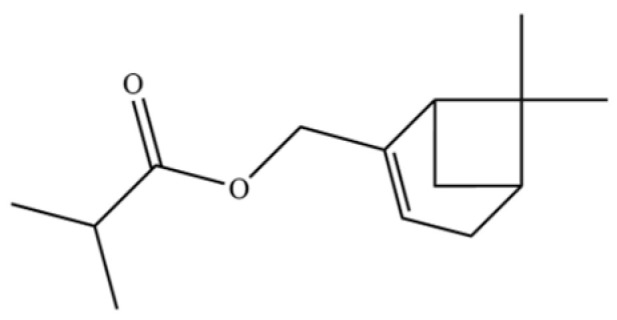	*	*	Monoterpenoid ester	[36]
180	4-Methylphenol, isopropyl ether	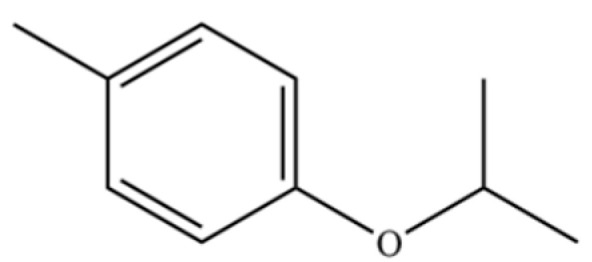	*	*	Phenolic ether	[36]
181	Benzene, (butoxymethyl)-	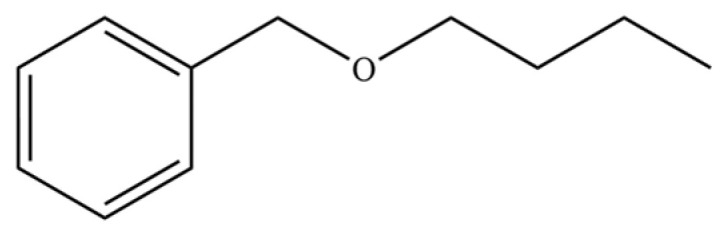	*	*	Aromatic ether	[36]
182	9-Dodecen-1-ol, acetate, (E)-	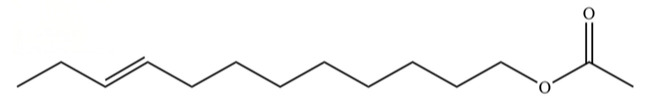	*	*	Fatty ester	[36]
183	5-Decen-1-ol, acetate, (E)-	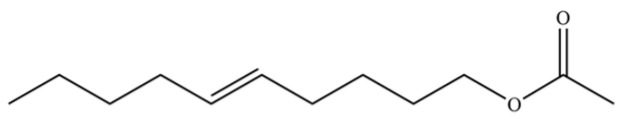	*	*	Fatty ester	[36]
184	Hexanal	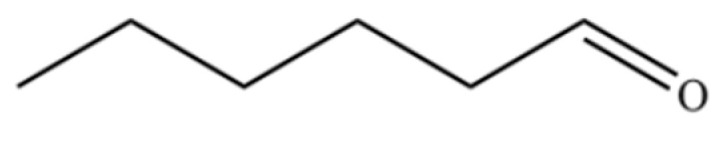	*	*	Aldehyde	[44]
185	p-Cymene	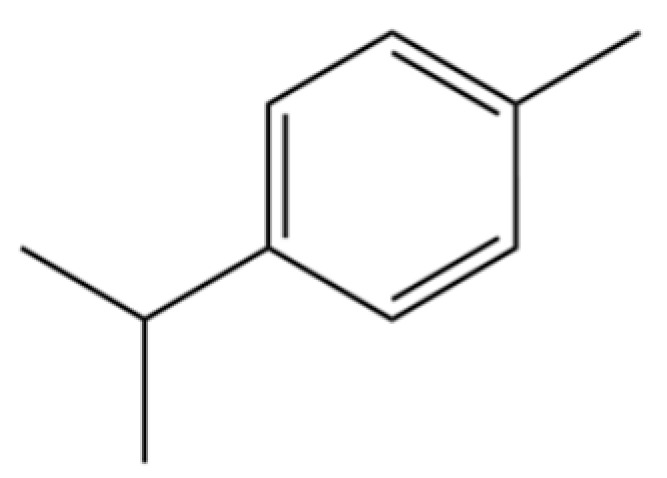	*	*	Monoterpenoid aromatic	[21]
186	Furan, 3-(4-methyl-3-pentenyl)-	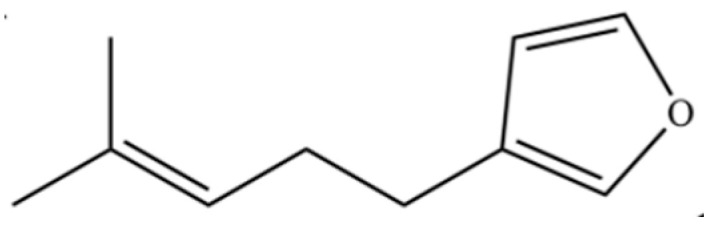	*	*	Furan derivative	[36]
187	Geraniol	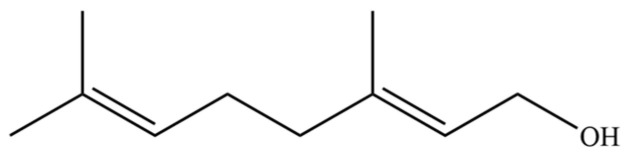	*	*	Monoterpenoid	[45]
188	Octanoic acid, ethyl ester	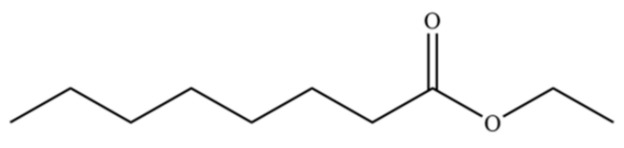	*	*	Fatty ester	[36]
189	2,6-Octadienal, 3,7-dimethyl-, (E)-	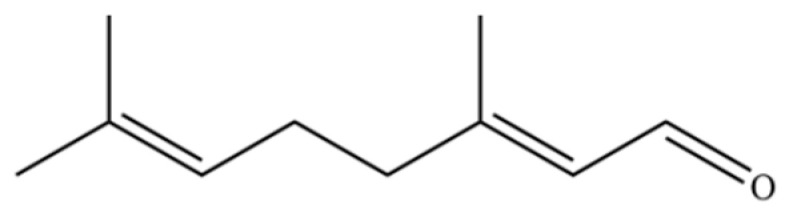	*	*	Monoterpenoid aldehyde	[36]
190	Naphthalene, 1,2,3,4-tetrahydro-1,6-dimethyl-4-(1-methylethyl)-, (1S-cis)-	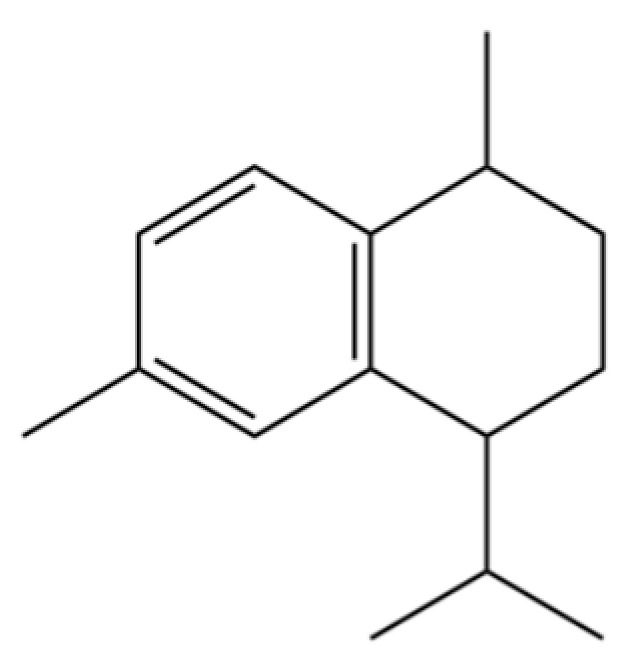	*	*	Sesquiterpenoid	[36]
191	Guaiol	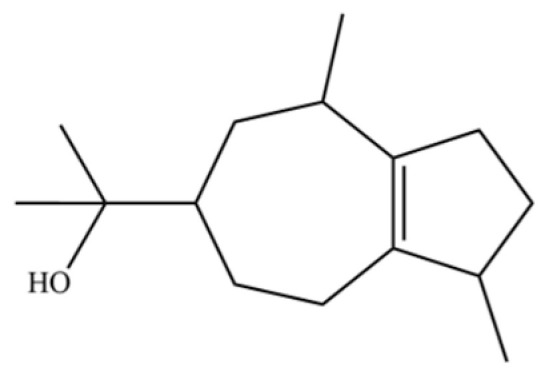	*	*	Sesquiterpenoid	[45]
192	5-Azulenemethanol, 1,2,3,3a,4,5,6,7-octahydro-α,α,3,8-tetramethyl-, [3S-(3.α,3a.β,5.α)]-	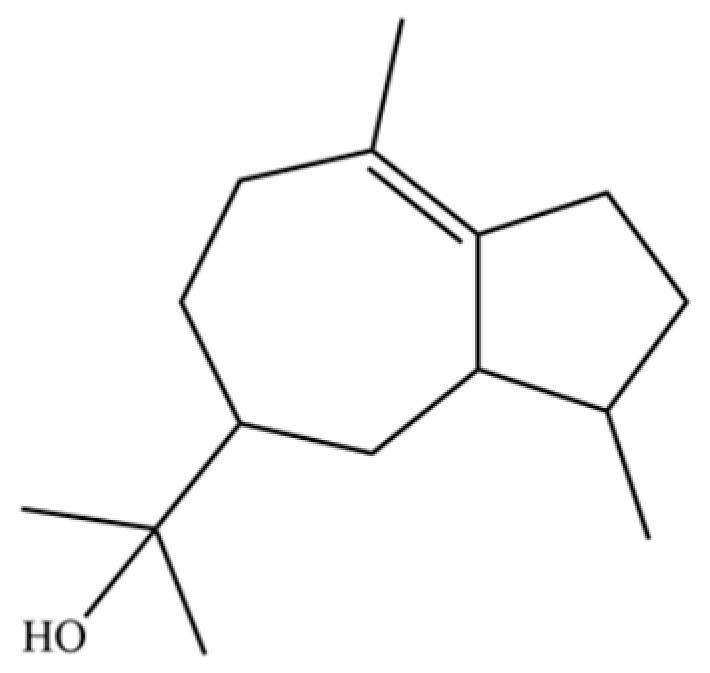	*	*	Sesquiterpenoid	[36]
193	α-Calacorene	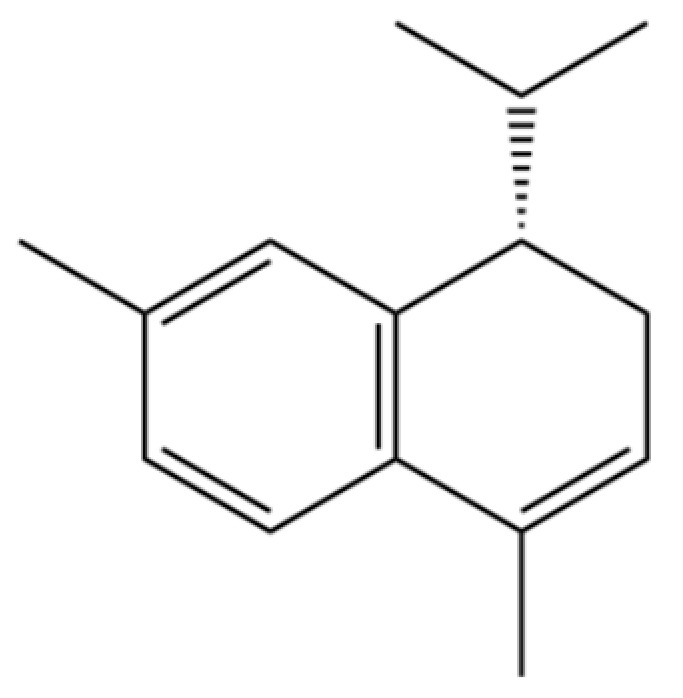	*	*	Sesquiterpenoid	[36]
194	1H-3a,7-Methanoazulene, octahydro-3,8,8-trimethyl-6-methylene-, [3R-(3.α,3a.β,7.β,8a.α)]-	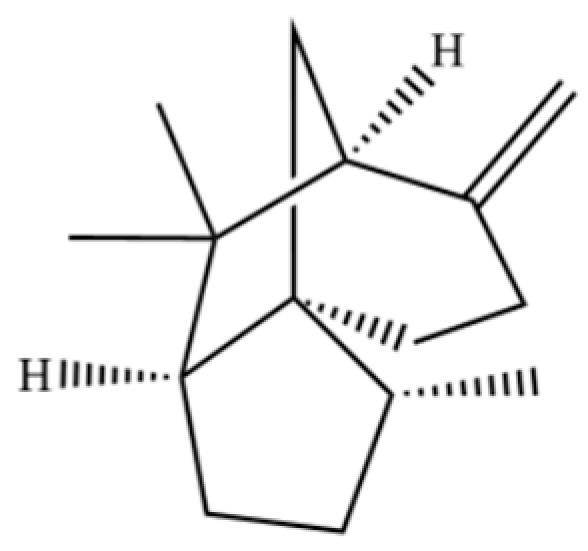	*	*	Sesquiterpenoid	[36]
195	Bicyclosesquiphellandrene	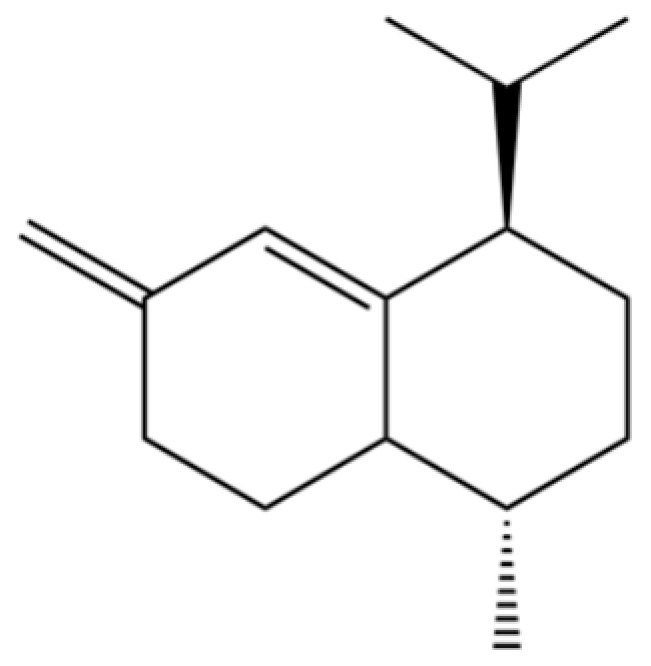	*	*	Sesquiterpenoid	[36]
196	Podocarpa-6,13-diene, 13-isopropyl-	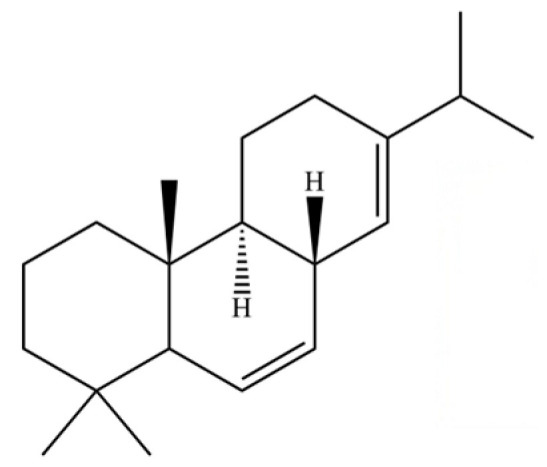	*	*	Diterpenoid	[36]
197	Bicyclo[3.1.0]hexan-3-ol, 4-methylene-1-(1-methylethyl)-, acetate	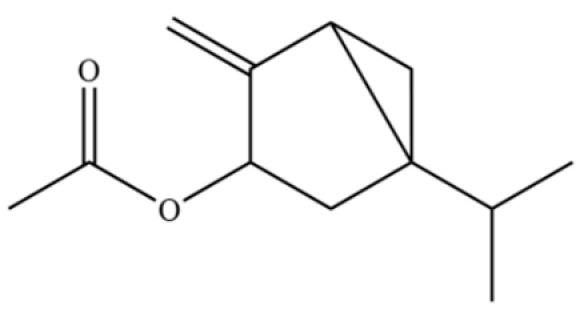	*	*	Monoterpenoid ester	[36]
198	Ethanone, 1,1′,1″-(1,3,5-benzenetriyl)tris-	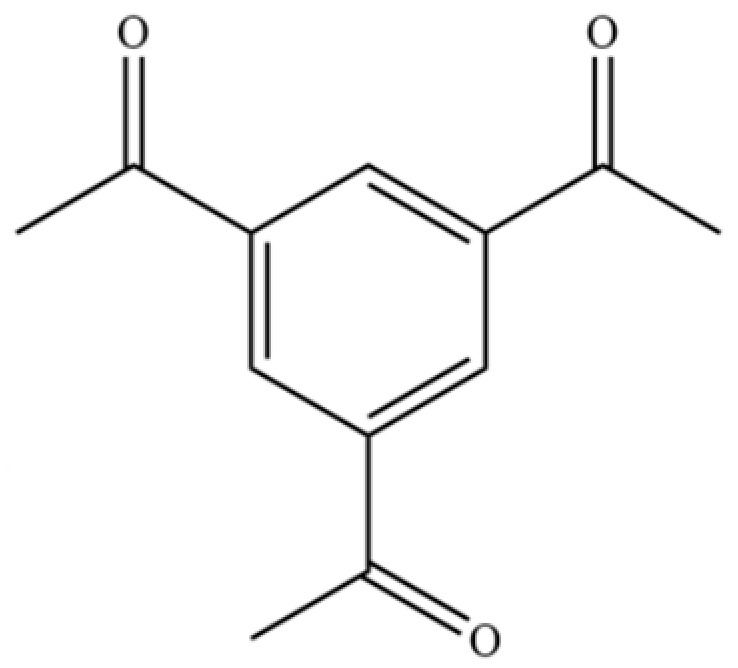	*	*	Aromatic ketone	[36]
199	Ethanone, 1-(5-methyl-1-phenyl-1H-pyrazol-4-yl)-	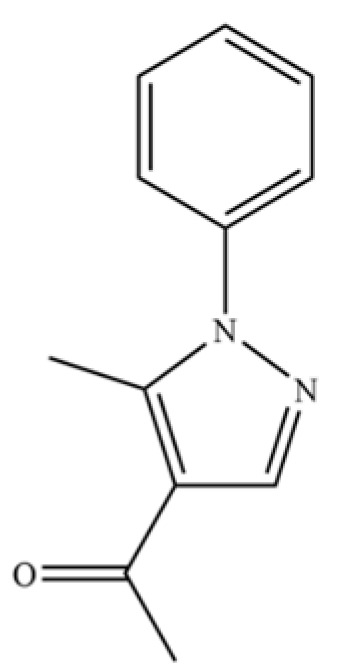	*	*	Heterocyclic ketone	[36]
200	Naphthalene, 1,2,4a,5,8,8a-hexahydro-4,7-dimethyl-1-(1-methylethyl)-, (1.α,4a.β,8a.α)-(.+/-.)-	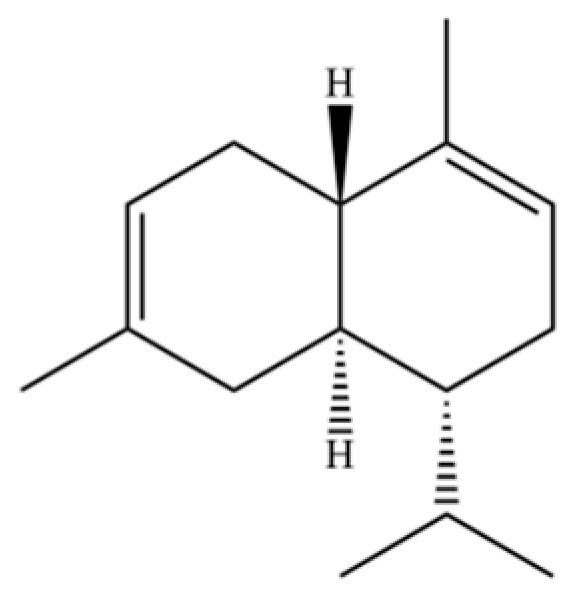	*	*	Sesquiterpenoid	[36]
201	Benzene, 1-methyl-3-(1-methylethyl)-	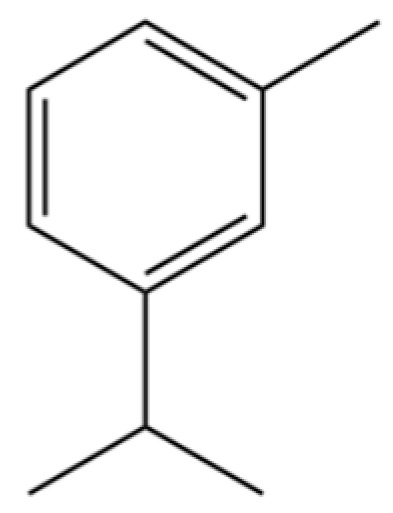	*	*	Aromatic hydrocarbon	[36]
202	6-Isopropyl-1,4-dimethylnaphthalene	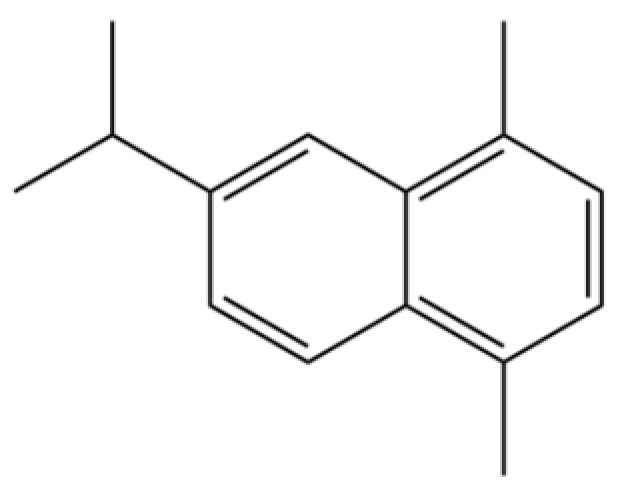	*	*	Aromatic hydrocarbon	[36]
203	Furan, 2-hexyl-	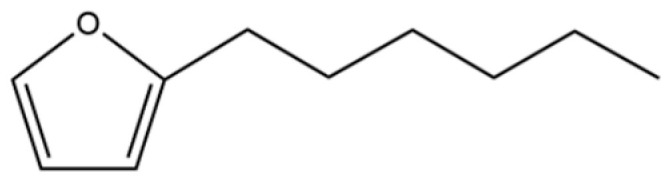	*	*	Furan derivative	[36]
204	(1S,4S,4aS)-1-Isopropyl-4,7-dimethyl-1,2,3,4,4a,5-hexahydronaphthalene	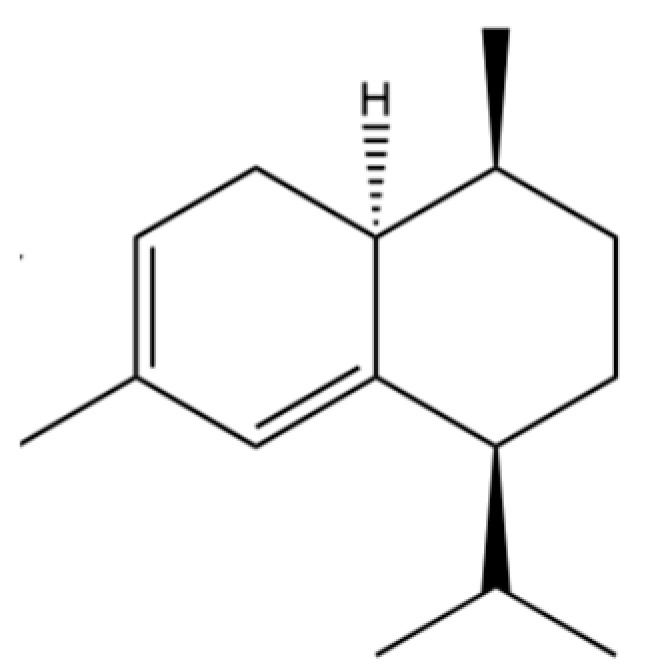	*	*	Sesquiterpenoid	[36]
205	Benzene, 1-methyl-4-(1,2,2-trimethylcyclopentyl)-, (R)-	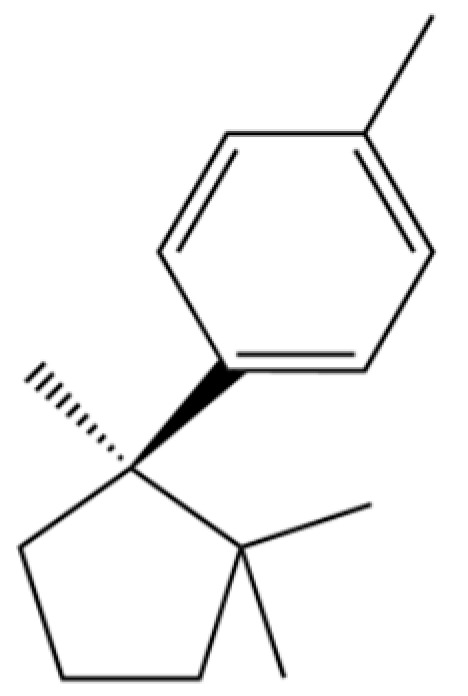	*	*	Aromatic hydrocarbon	[36]
206	Cadina-1(10),6,8-triene	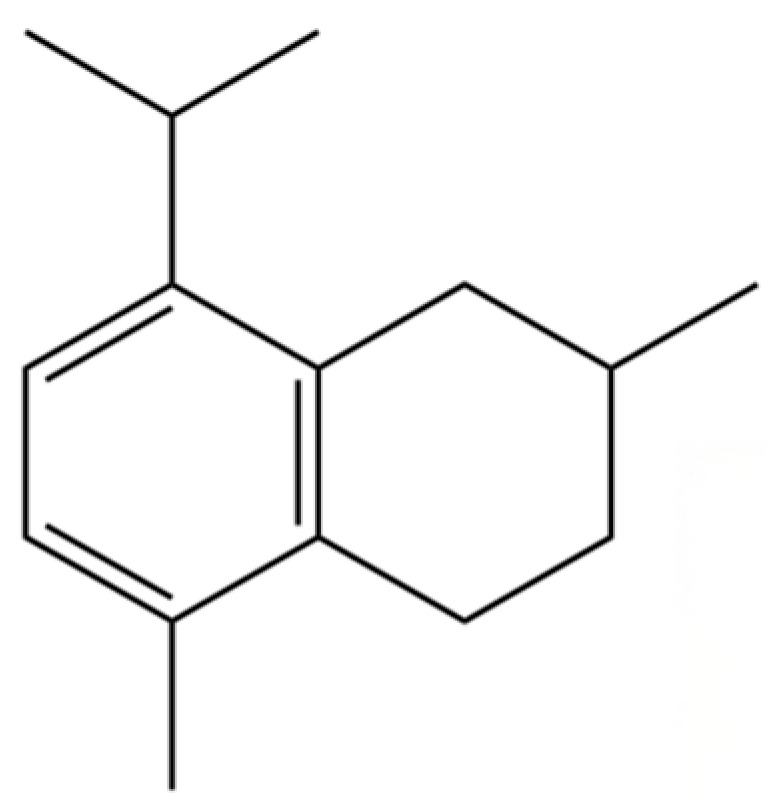	*	*	Sesquiterpenoid	[36]
207	Bicyclo[5.2.0]nonane, 2-methylene-4,8,8-trimethyl-4-vinyl-	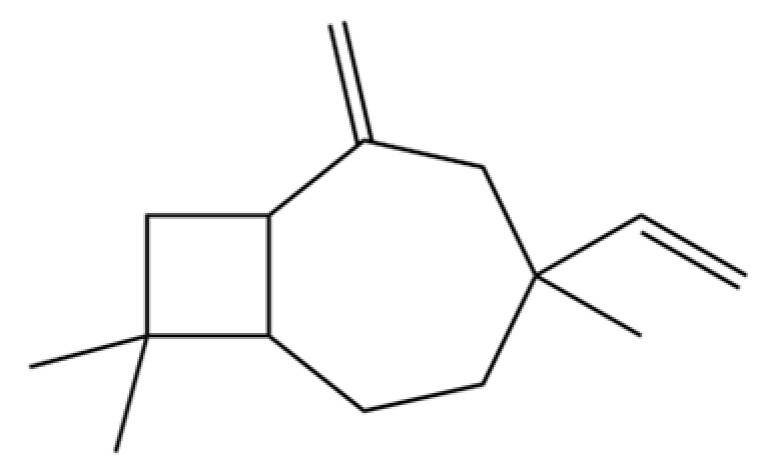	*	*	Sesquiterpenoid	[36]
208	Isoledene	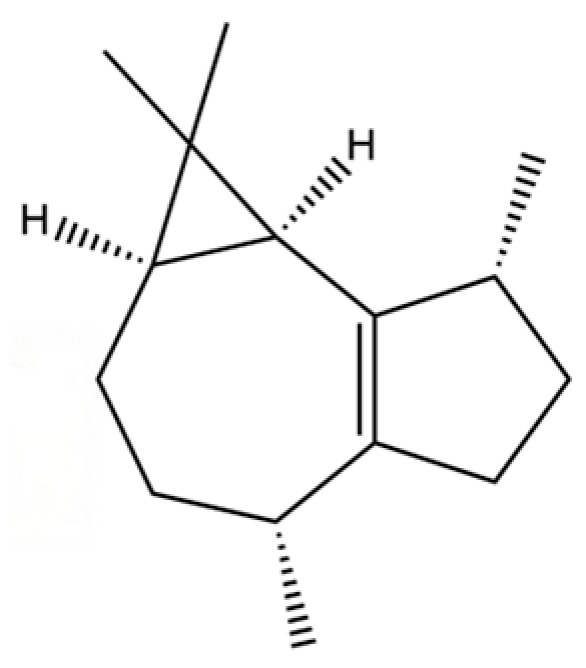	*	*	Sesquiterpenoid	[25]
209	1,4,7,-Cycloundecatriene, 1,5,9,9-tetramethyl-, Z,Z,Z-	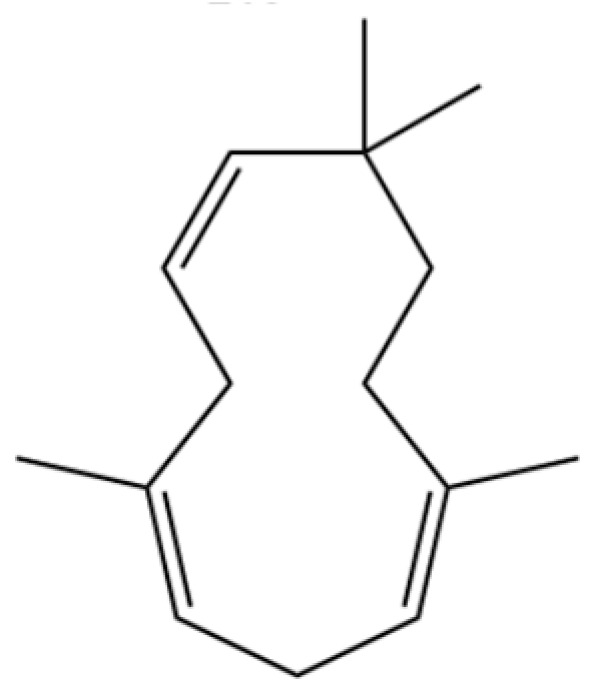	*	*	Sesquiterpenoid	[36]
210	α-Corocalene	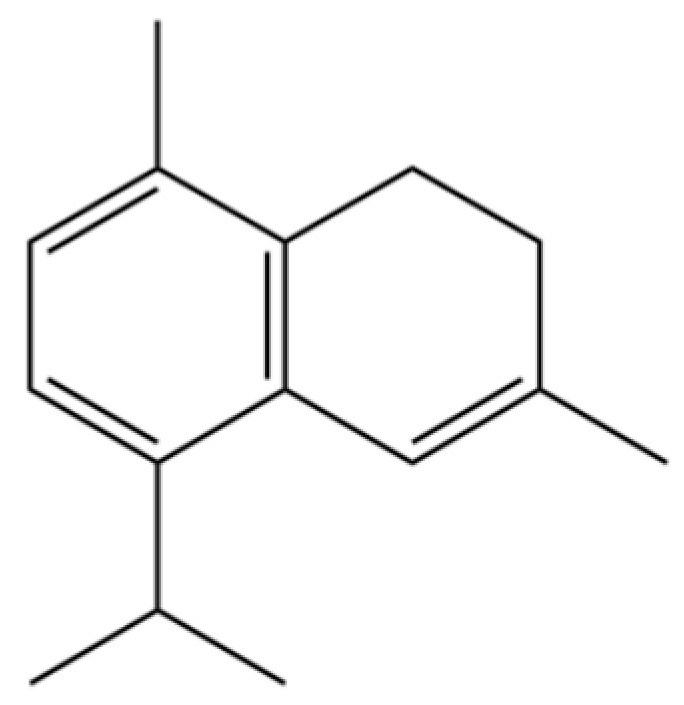	*	*	Sesquiterpenoid	[36]
211	Benzene, 1-ethenyl-3,5-dimethyl-	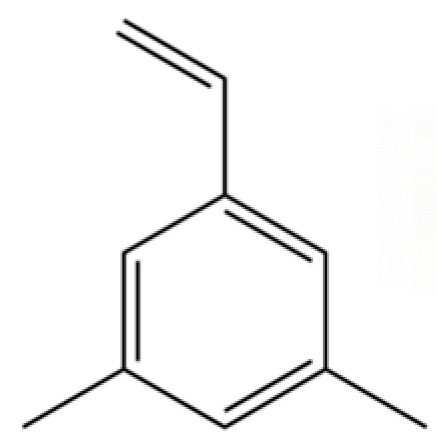	*	*	Aromatic hydrocarbon	[36]
212	Benzene, 1-methoxy-4-methyl-2-(1-methylethyl)-	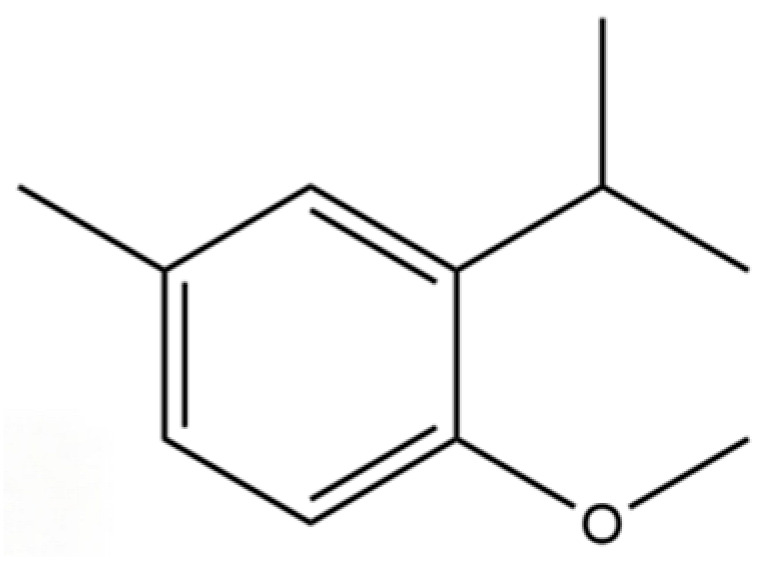	*	*	Aromatic ether	[36]
213	3-Cyclohexen-1-one, 2-isopropyl-5-methyl-	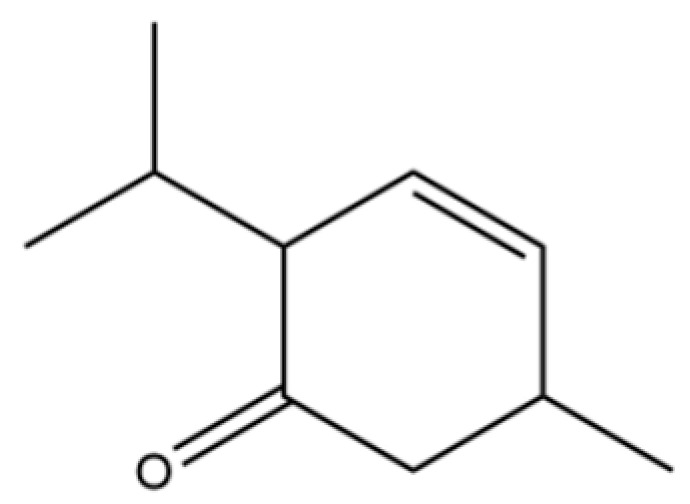	*	*	Monoterpenoid ketone	[36]
214	2,2′-Isopropylidenebis(5-methylfuran)	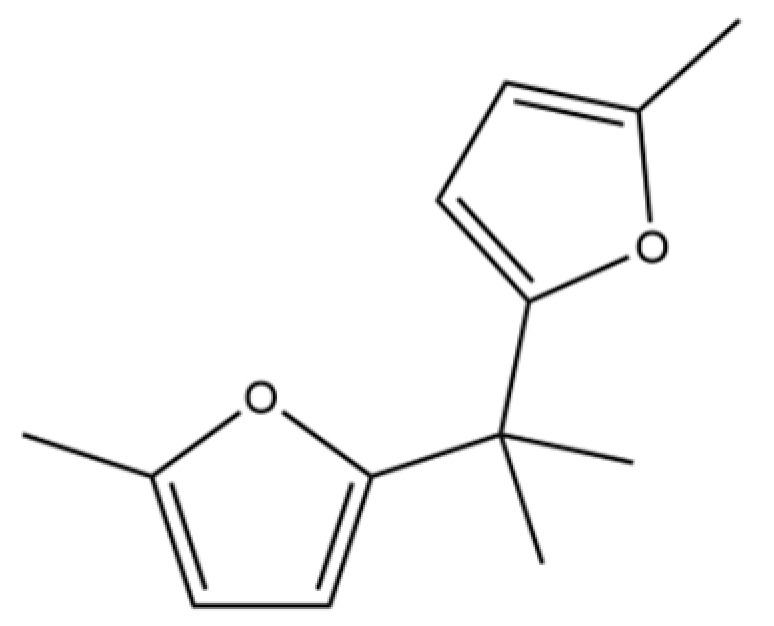	*	*	Furan derivative	[36]
215	3-Acetyl-2,5-dimethylbenzo(b)thiophene	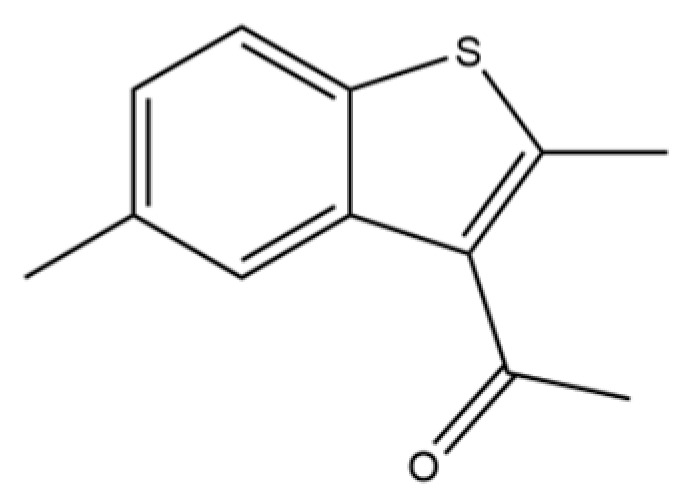	*	*	Heterocycle	[36]
216	Benzaldehyde, 2,4-dihydroxy-3,6-dimethyl-	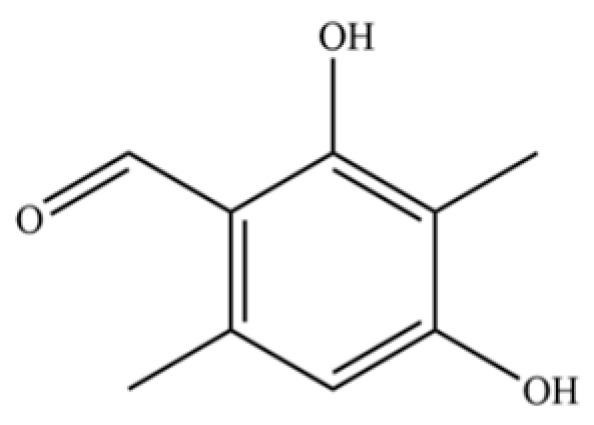	*	*	Phenolic aldehyde	[36]
217	β-Panasinsene	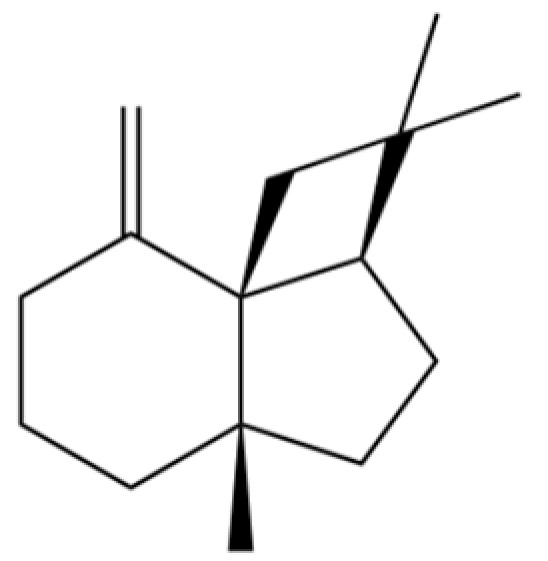	*	*	Sesquiterpenoid	[46]
218	2,3-Dimethyl-5-[(methylthio)propyl]pyrazine	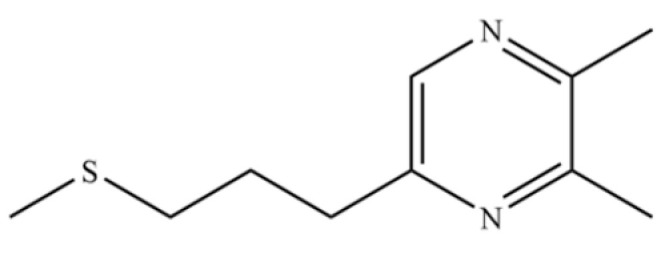	*	*	Pyrazine derivative	[36]
219	Tricyclo[4.4.0.02,7]decane, 1-methyl-3-methylene-8-(1-methylethyl)-, stereoisomer	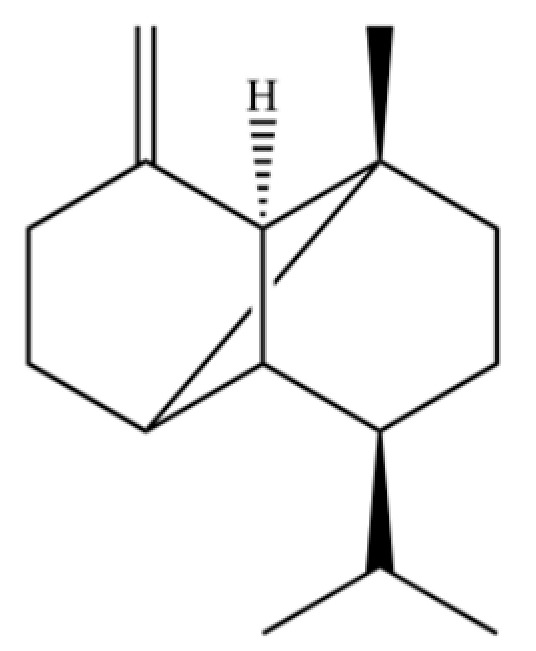	*	*	Sesquiterpenoid	[36]
220	Coumarin, 3,4-dihydro-4,4,6,8-tetramethyl-	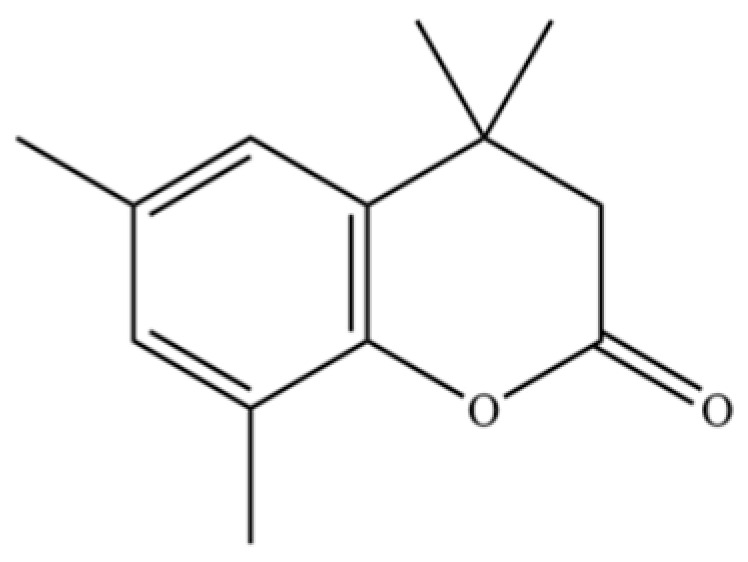	*	*	Coumarin	[36]
221	1,4-Methanocycloocta[d]pyridazine, 1,4,4a,5,6,9,10,10a-octahydro-11,11-dimethyl-, (1.α,4.α,4a.α,10a.α)-	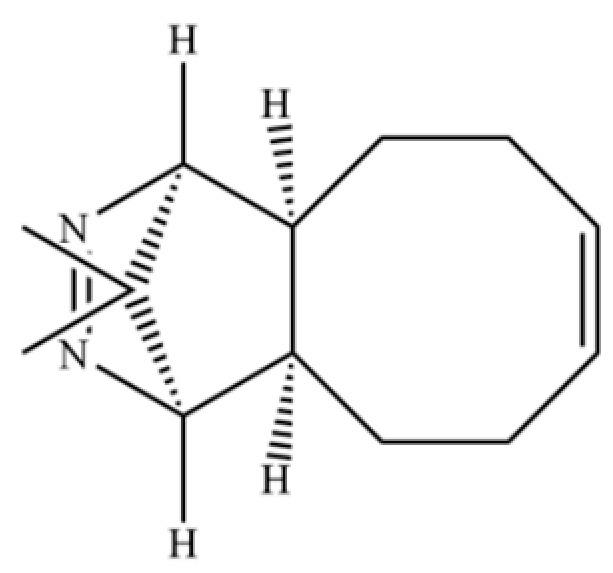	*	*	Heterocycle	[36]
222	Dibenzofuran, 2-methoxy-	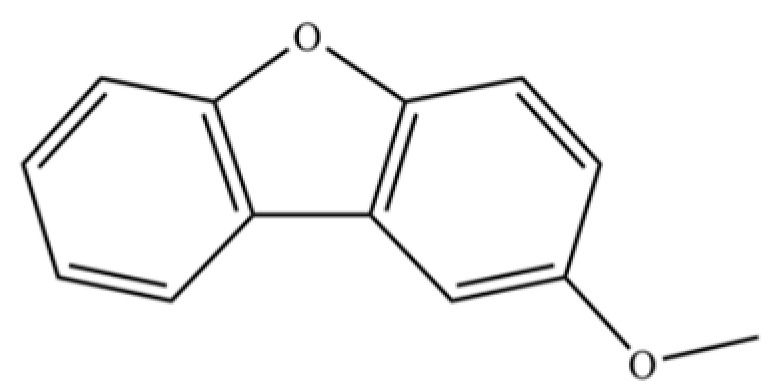	*	*	Furan derivative	[36]
223	1H-Imidazole, 1-acetyl-	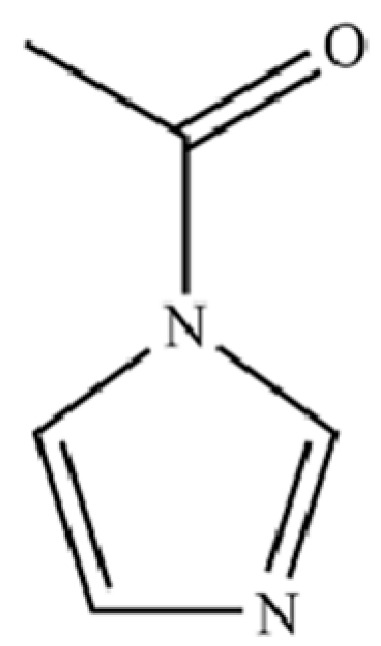	*	*	Heterocycle	[36]
224	4-Formyl-3,5-dimethyl-1H-pyrrole-2-carbonitrile	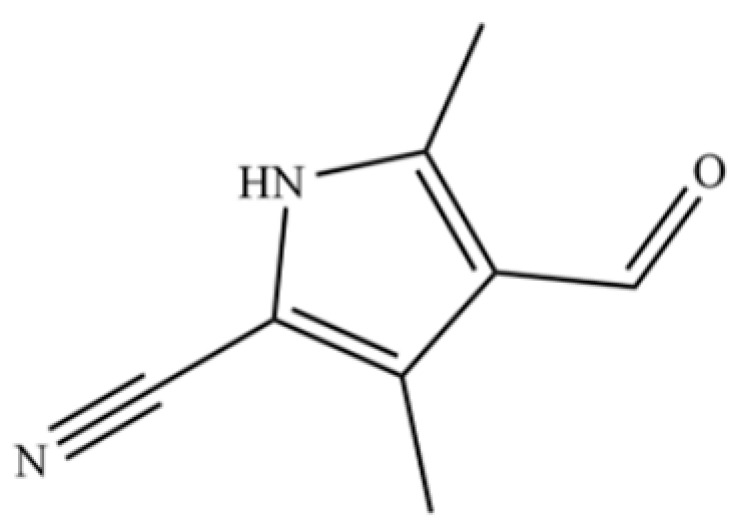	*	*	Heterocycle	[36]
225	(4R,4aS,6S)-4,4a-Dimethyl-6-(prop-1-en-2-yl)-1,2,3,4,4a,5,6,7-octahydronaphthalene	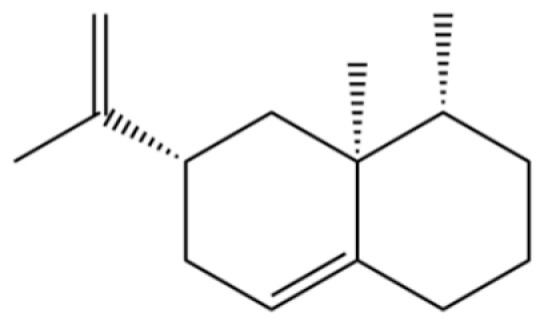	*	*	Sesquiterpenoid	[36]
226	3-Isobutyl-4,5-dimethyl-3H-isobenzofuran-1-one	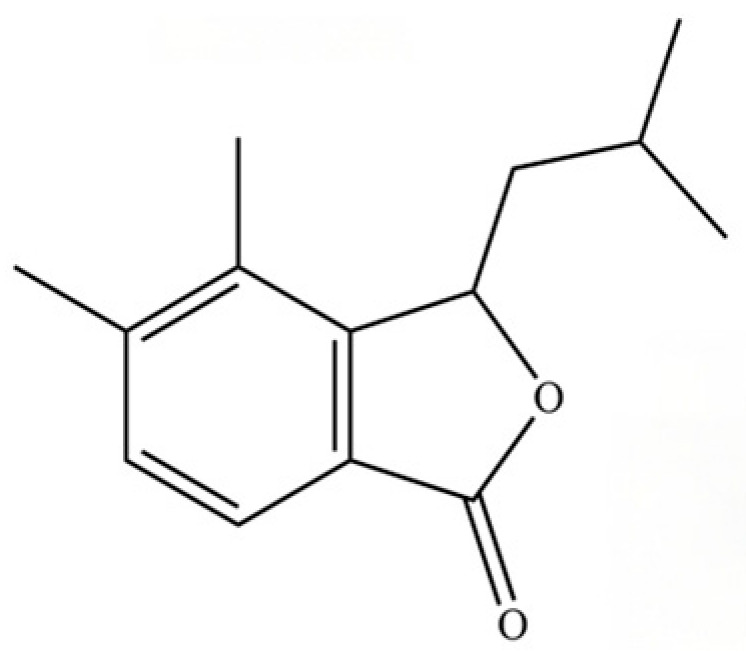	*	*	Phthalide derivative	[36]
227	4-Acetoxy-3-methoxystyrene	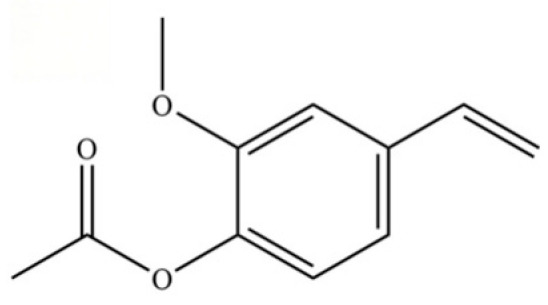	*	*	Phenylpropanoid	[36]
228	2-Acetyl-3,5-dimethylbenzo(b)thiophene	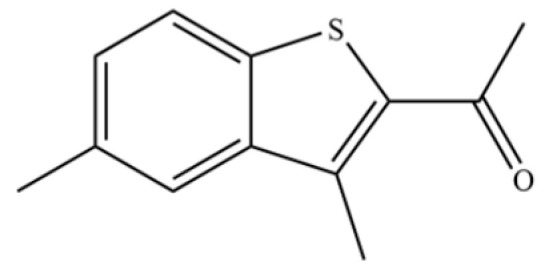	*	*	Heterocycle	[36]
229	1,3,5-Cycloheptatriene, 2,3,4,5,7,7-hexamethyl-	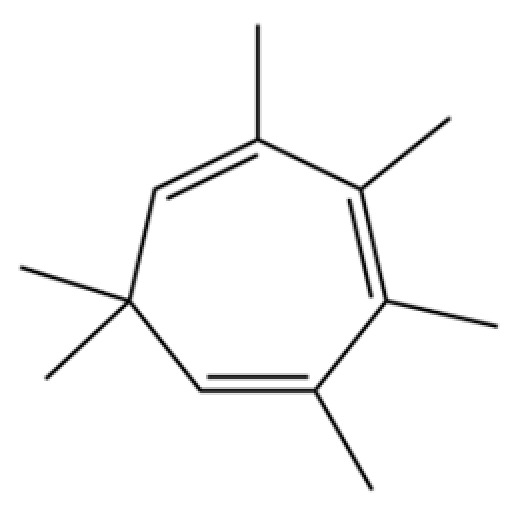	*	*	Troponoid	[36]
230	1H-1,2,3-Triazole	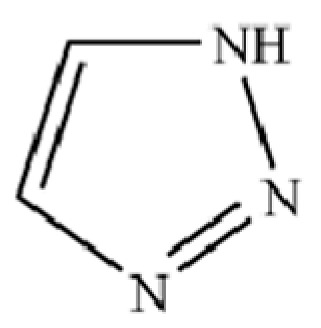	*	*	Heterocycle	[36]
231	Carvenone	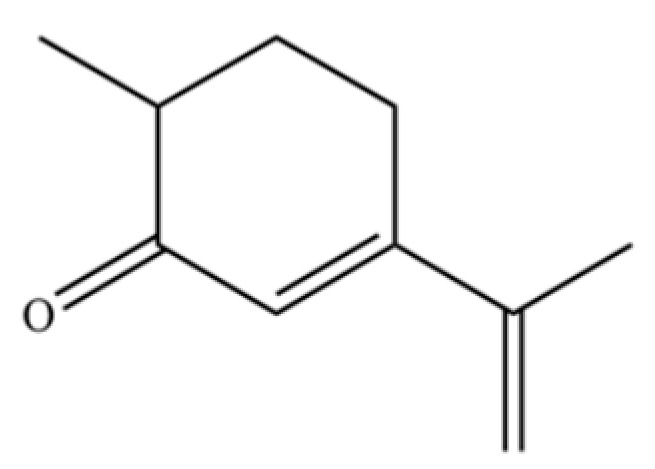	*	*	Monoterpenoid ketone	[47]
232	5-Azulenemethanol, 1,2,3,4,5,6,7,8-octahydro-α,α,3,8-tetramethyl-, acetate, [3S-(3.α,5.α,8.α)]-	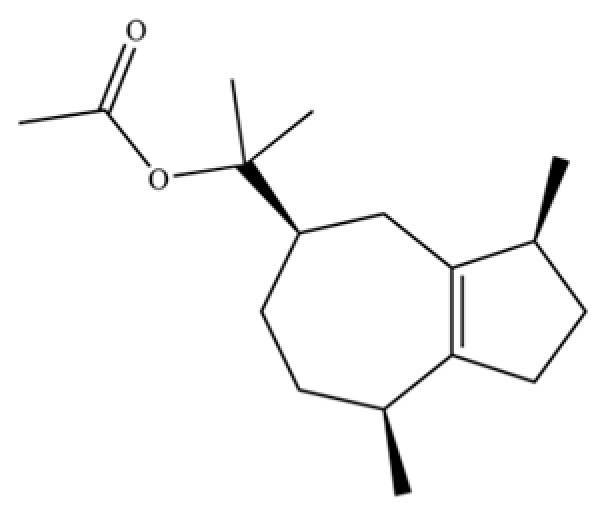	*	*	Sesquiterpenoid ester	[36]
233	vanillic acid	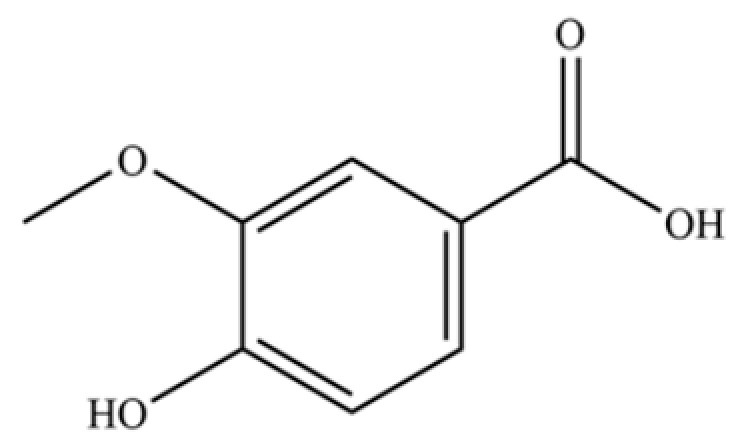	*	*	Phenolic acid	[48]
234	L-phenylalanine	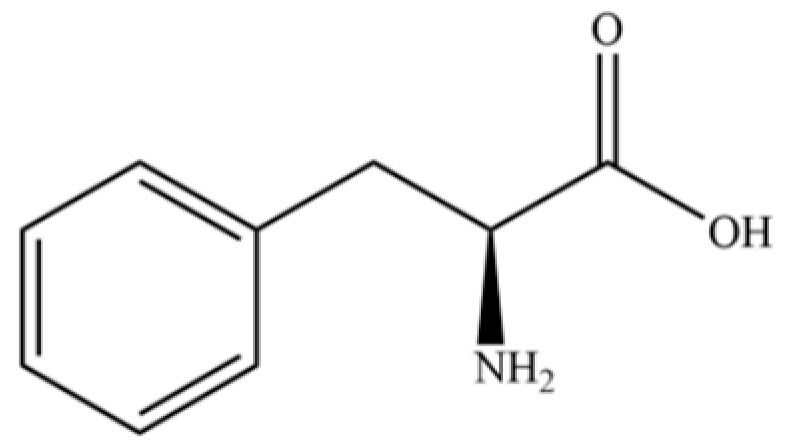	*	*	Amino acid	[49]
235	quinic acid	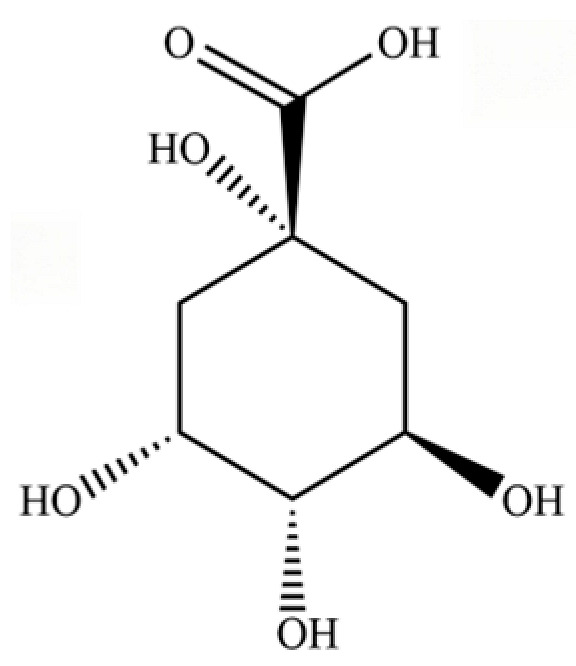	*	*	Cyclitol carboxylic acid	[3]
236	D-tryptophan	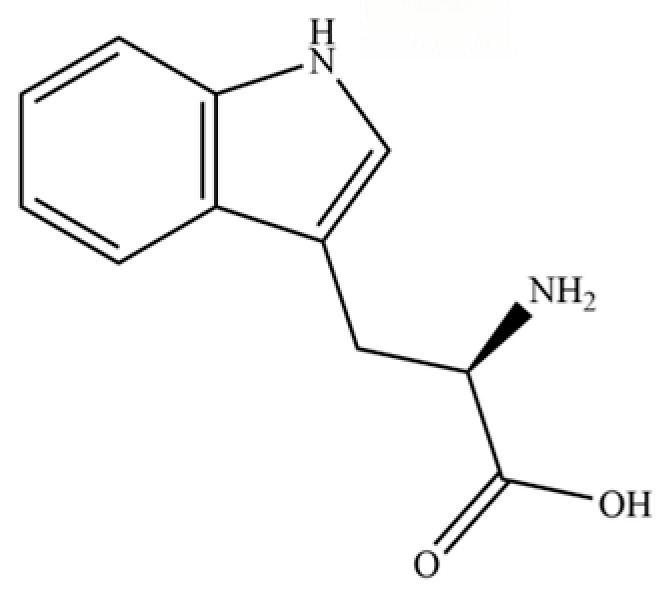	*	*	Amino acid	[50]
237	(1R, 4S, 6R)-1, 3, 3-trimethyl-2- oxabicyclo[2.2.2]oct-6-yl-6-O-β-D-glucopyranosyl-β-D-glucopyranoside	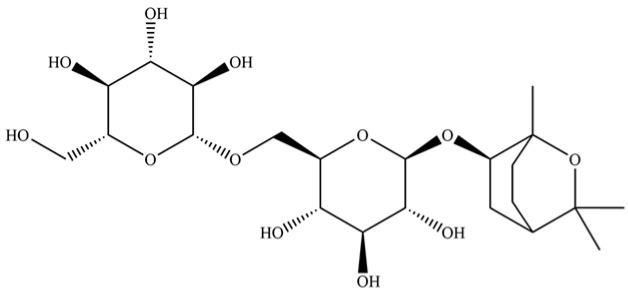	*	*	Monoterpenoid glycoside	[51]
238	2-(3-isopropyl-4-methyl-pent-3- en-1-ynyl)-2-methyl-cyclobutanone	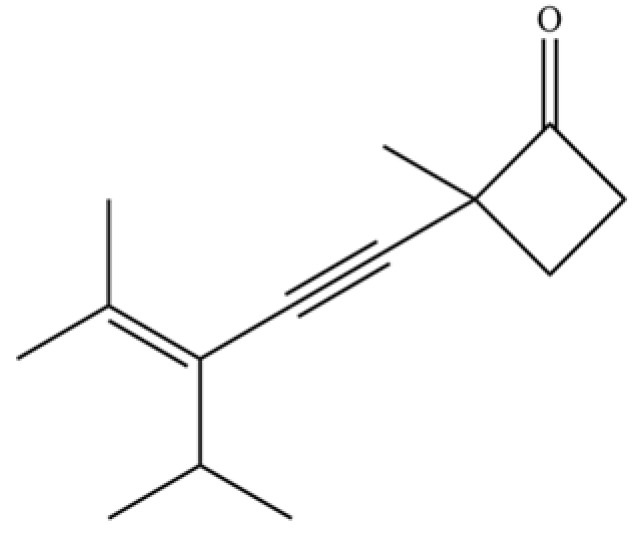	*	*	Polyacetylene	[51]
239	citric acid	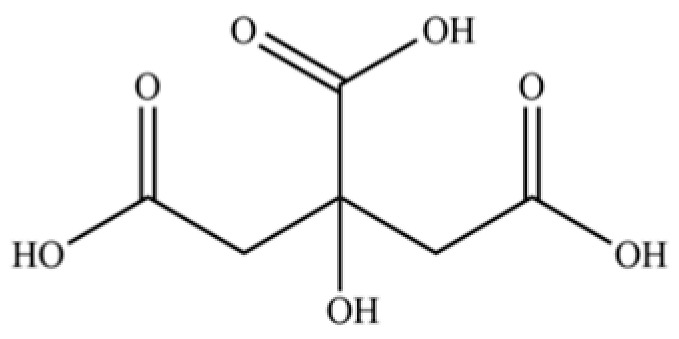	*	*	Organic acid	[52]
240	neochlorogenic acid	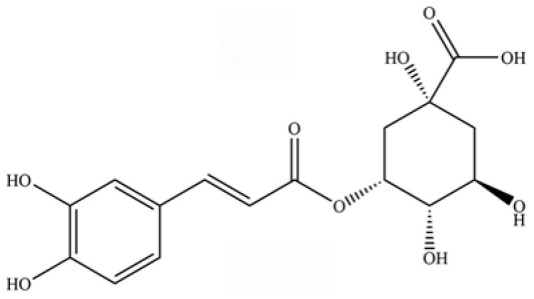	*	*	Phenolic acid (chlorogenic acid derivative)	[53]
241	scopoletin	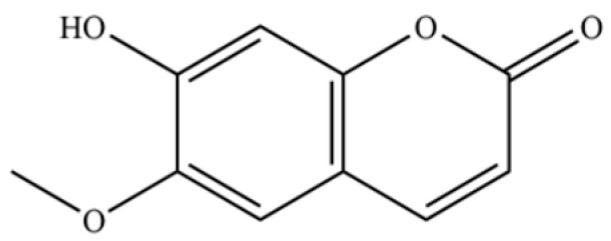	*	*	Coumarin	[54]
242	chlorogenic acid	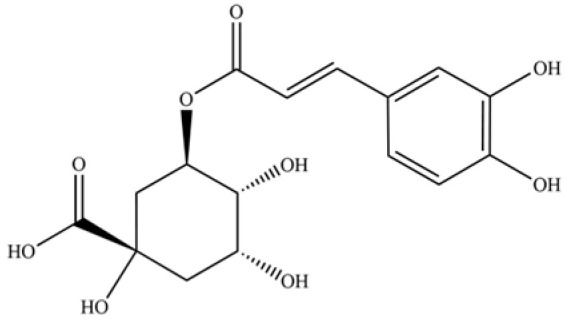	*	*	Phenolic acid	[55]
243	atractyloside A	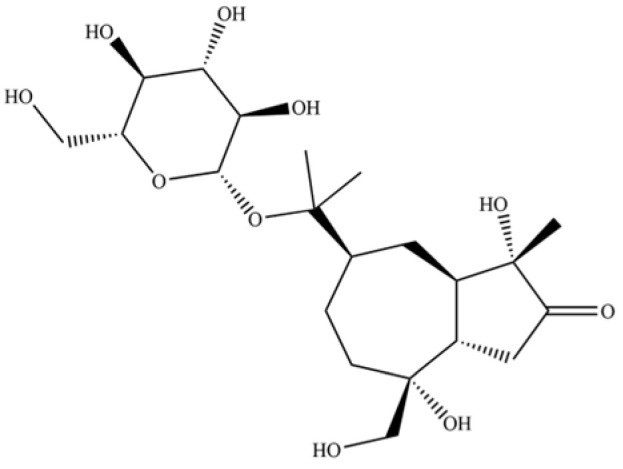	*	*	Sesquiterpenoid glycoside	[56]
244	hymecromone	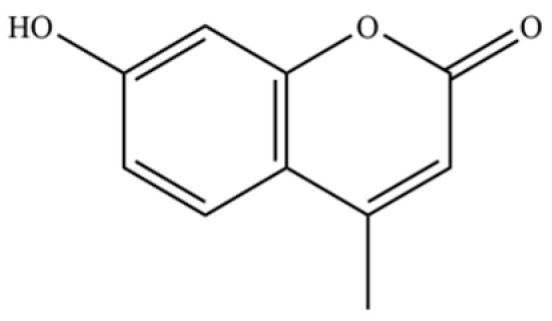	*	*	Coumarin	[57]
245	cryptochlorogenin acid	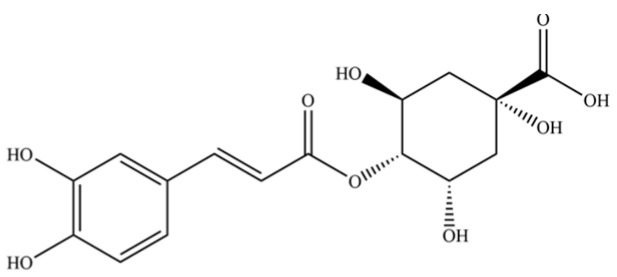	*	*	Phenolic acid	[58]
246	coumaroylquinic acid	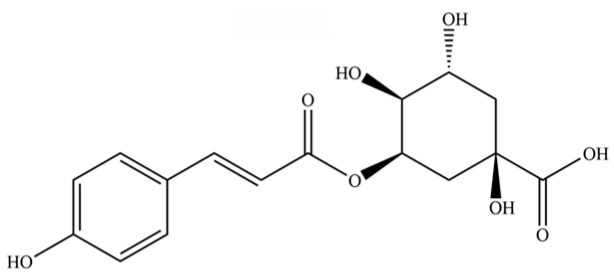	*	*	Phenolic acid	[58]
247	5-O-feruloylquinic acid	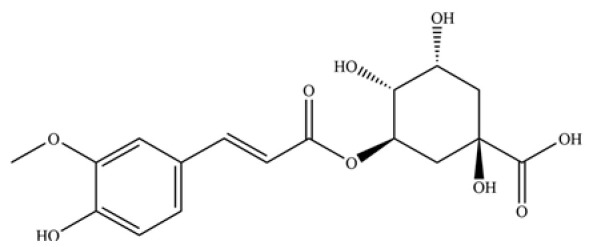	*	*	Phenolic acid	[58]
248	dihydrosyrindine	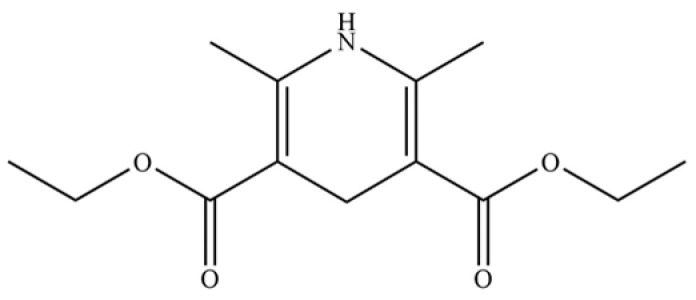	*	*	Glycosides	[58]
249	naphthol(1,2)furan-2-one	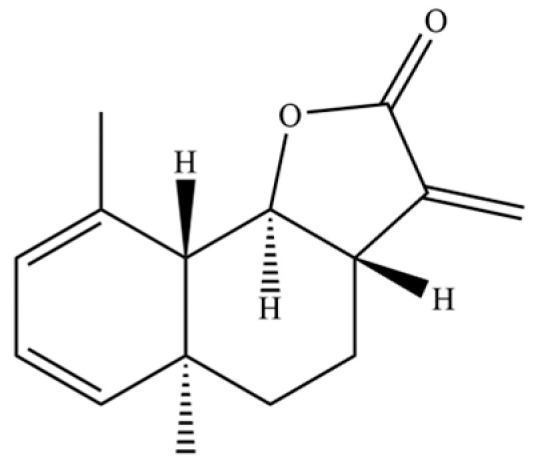	*	*	Naphthofuran derivative	[51]
250	rutin	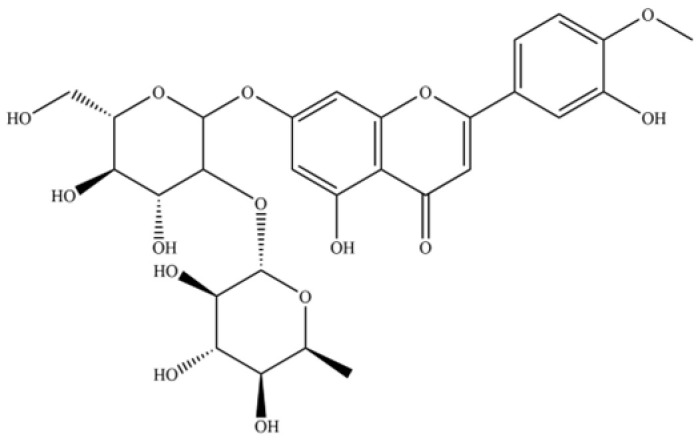	*	*	Flavonoid glycoside	[59]
251	atractylenolide III	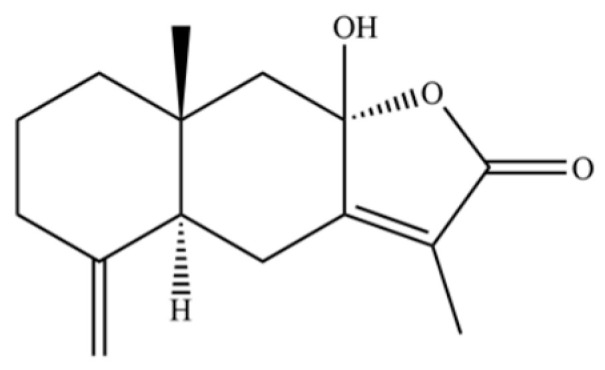	*	*	Sesquiterpenoid lactone	[60]
252	vitexin	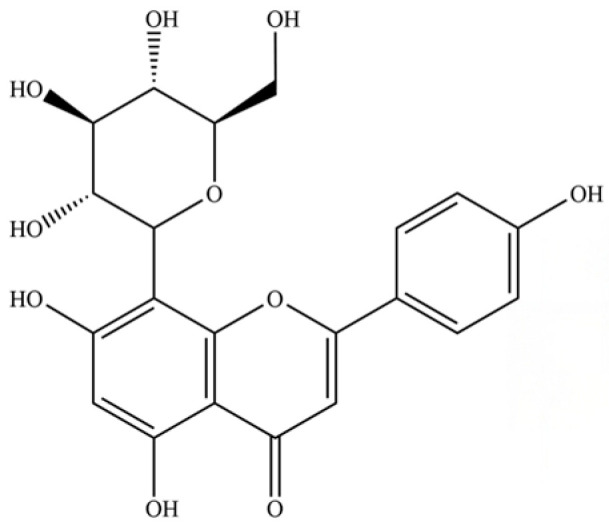	*	*	Flavonoid glycoside	[61]
253	naringenin chalcone	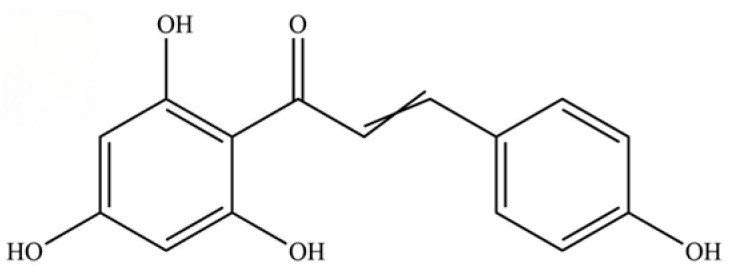	*	*	Flavonoid (chalcone)	[62]
254	icariside D1	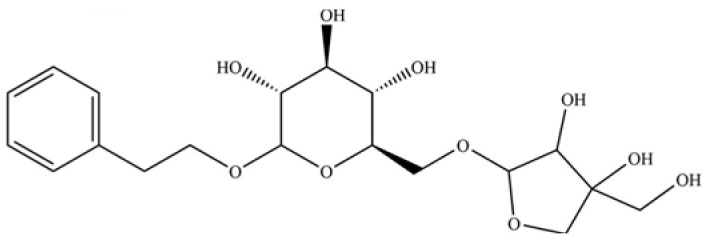	*	*	Phenolic glycoside	[58]
255	atractyloside I	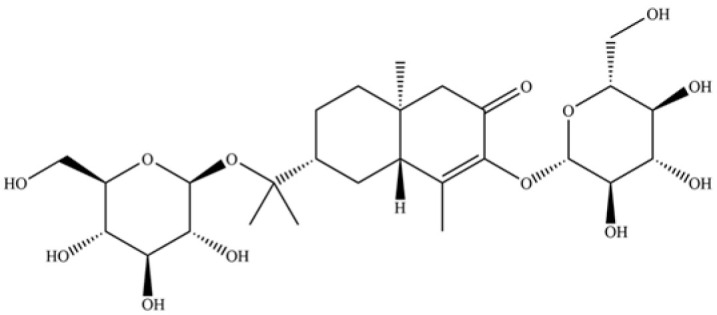	*	*	Sesquiterpenoid glycoside	[63]
256	dehydrocostus lactone	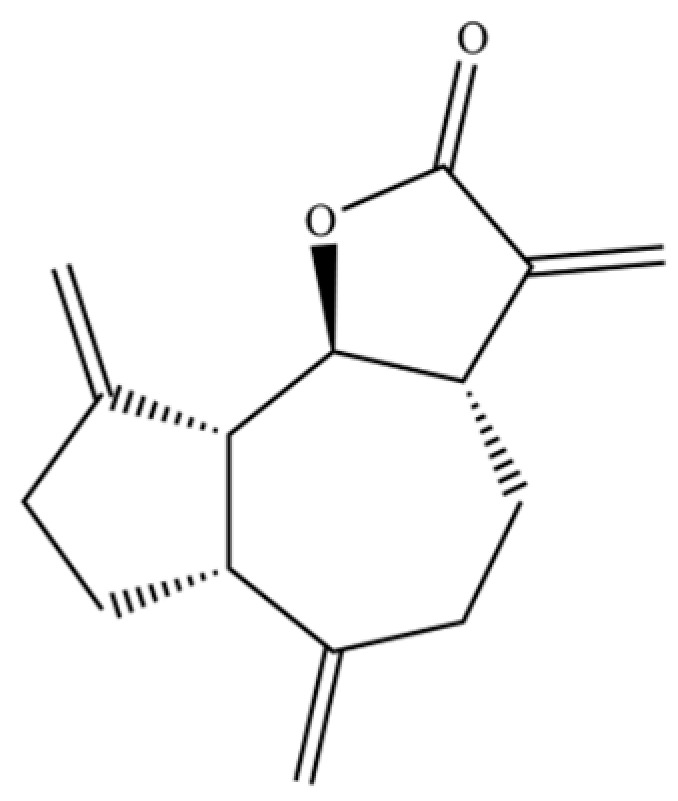	*	*	Sesquiterpenoid lactone	[51]
257	wogonoside	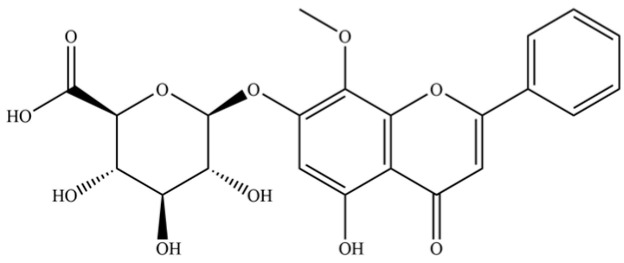	*	*	Flavonoid glycoside	[64]
258	atractylenolactam	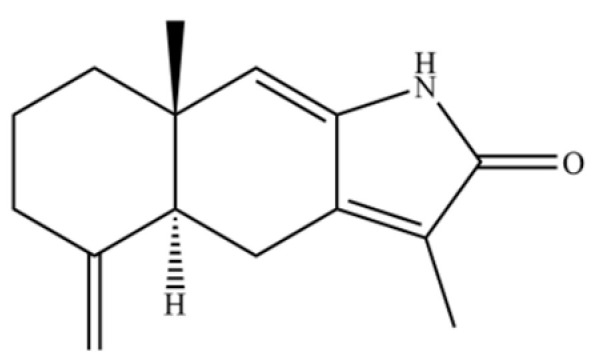	*	*	Sesquiterpenoid lactam	[65]
259	atractylodinol	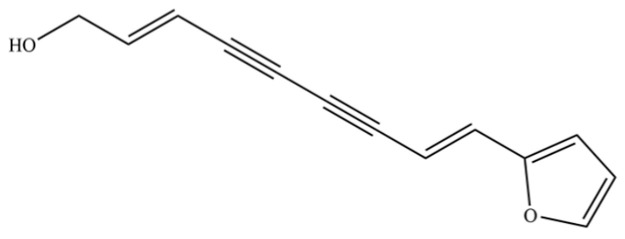	**	*	Polyacetylene	[66]
260	Atrachinenins E	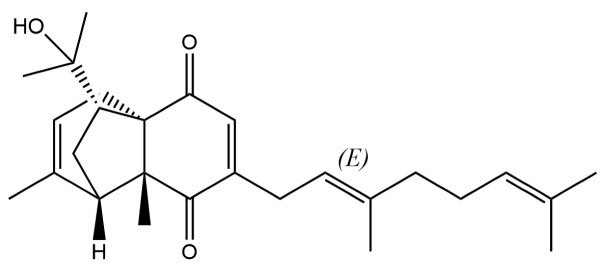	/	**	Sesquiterpenoid	[23]
261	(4β,5β,6β,7β)-Aristol-9-en-8-one	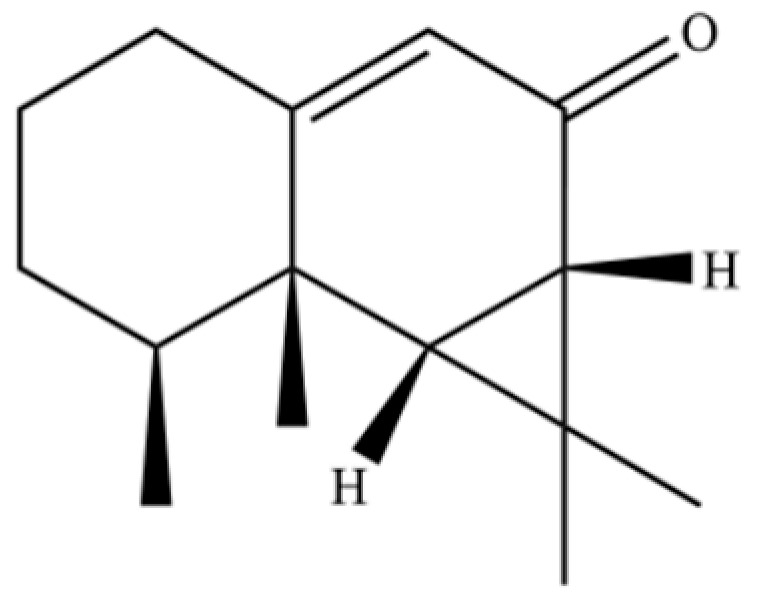	*	*	Sesquiterpenoid	[51]
262	diacetyl-atractylodiol	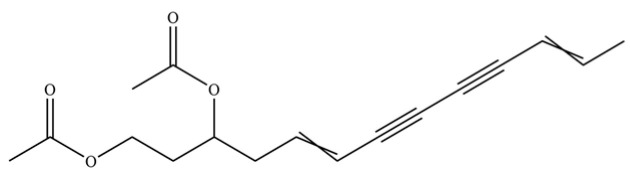	*	*	Polyacetylene derivative	[67]
263	(4E,6E,12E)-tetradeca-4,6,12- trien-8,10-diyne-1,3-diyl diacetate	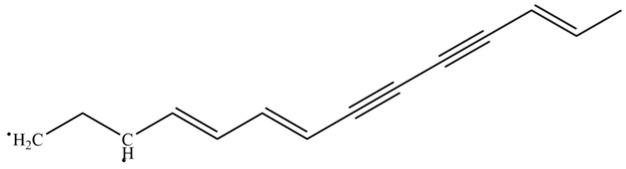	*	*	Polyacetylene ester	[51]
264	Acetyltetralone	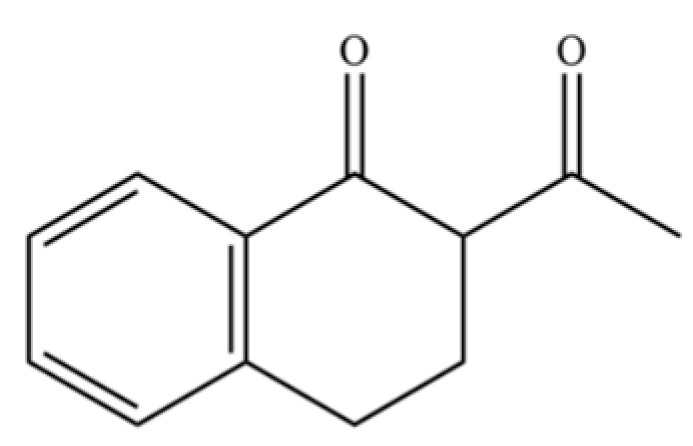	*	*	Aromatic ketone	[51]
265	9a-hydroxy-3,8a-dimethyl-5-methylene-2-oxo-2, 4, 4a, 5, 6, 7, 8, 8a, 9,9a-decahydronaphtho [2,3-b]furan-6-yl acetate	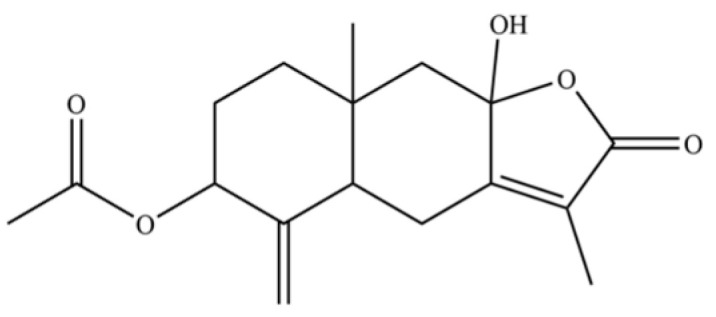	*	*	Sesquiterpenoid lactone	[51]
266	2-(biphenyl-4-yl)acetaldehyde	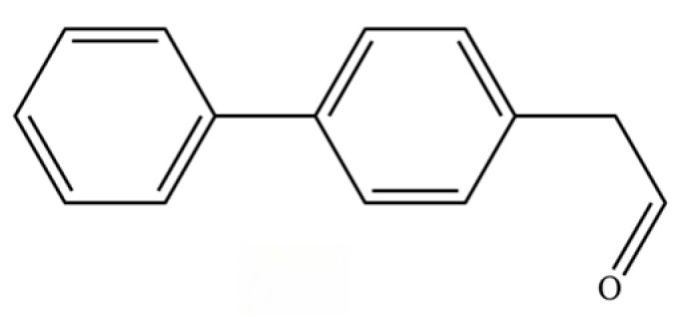	*	*	Aromatic aldehyde	[42]
267	3β-acetoxyatractylon	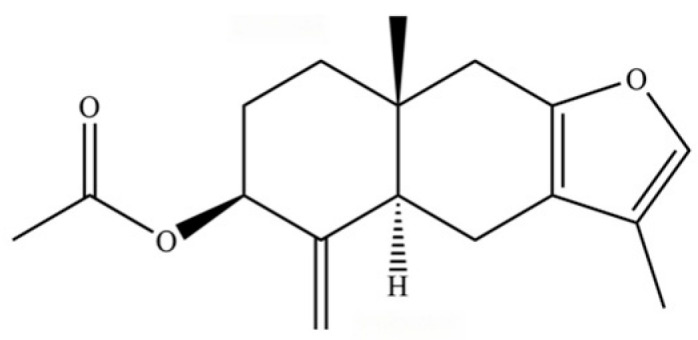	*	*	Sesquiterpenoid	[51]
268	atractylenolide II	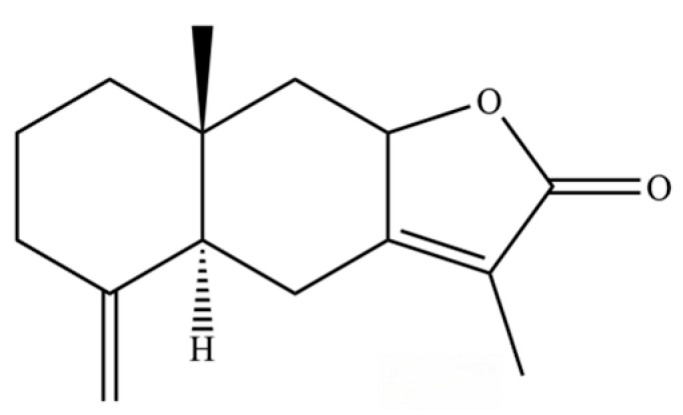	*	*	Sesquiterpenoid lactone	[60]
269	atractyloside H	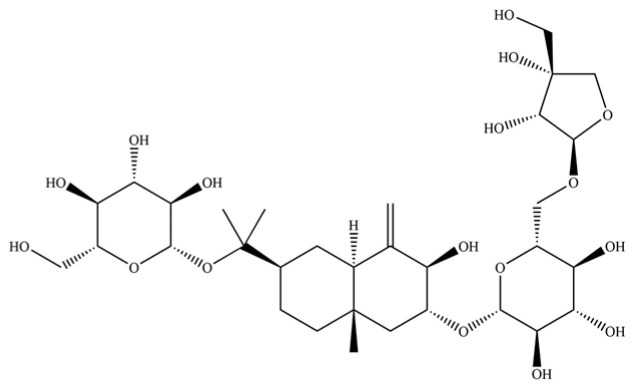	*	*	Sesquiterpenoid glycoside	[58]
270	acetylatractylodinol	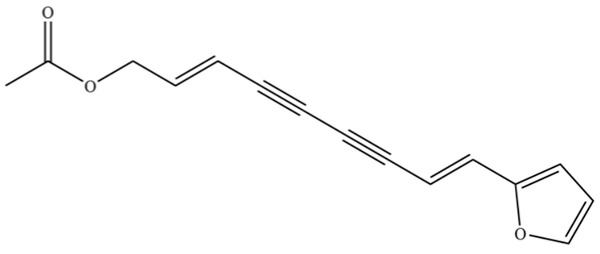	**	*	Polyacetylene ester	[68]
271	atractylenolide I	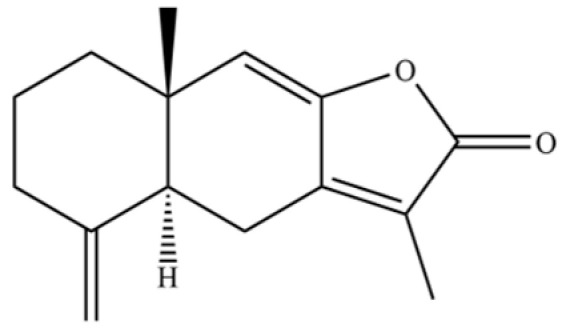	*	*	Sesquiterpenoid lactone	[21]
272	3β-hydroxyatractylon	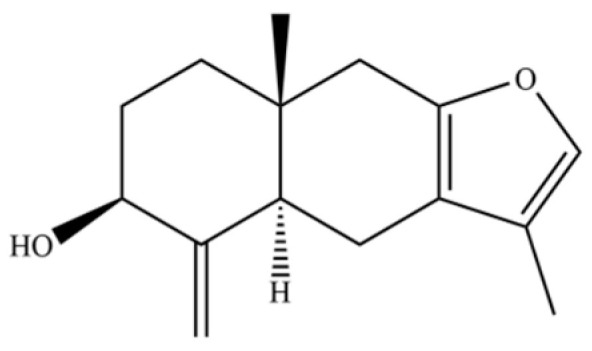	*	*	Sesquiterpenoid	[58]
273	α-cyperone	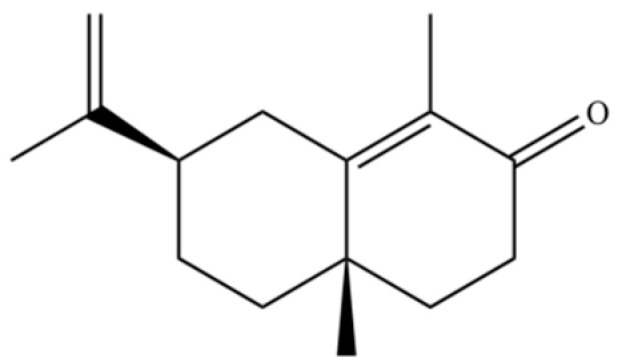	*	*	Sesquiterpenoid	[69]
274	9,10-epoxy-12(Z)-octadecenoic acid	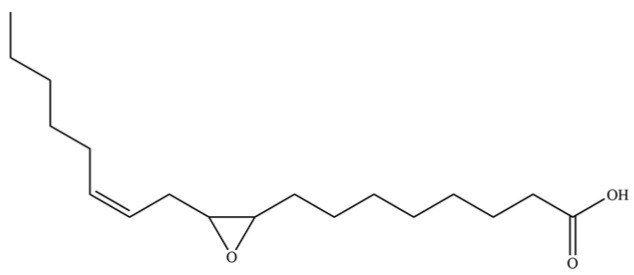	*	*	Fatty acid epoxide	[51]
275	(4E,6E,12E)-tetradecadiene-8,10-diyne-1, 3-diol-diacetate	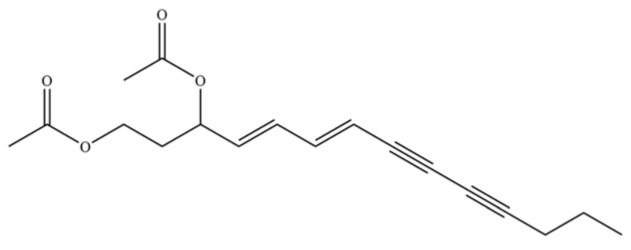	*	*	Polyacetylene ester	[51]
276	methyllinolenate	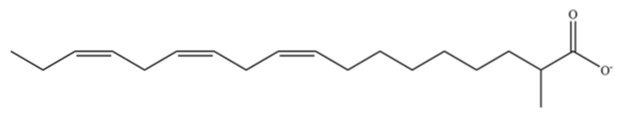	*	*	Fatty acid methyl ester	[51]
277	muscone	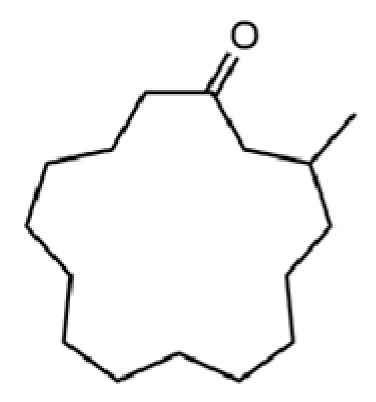	*	*	Macrocyclic ketone	[51]
278	7-methoxy-2-methyl-2-(4- methylpent-3-enyl)-2H- chromene	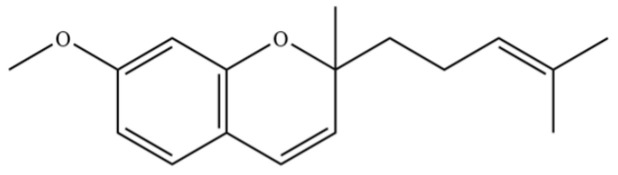	*	*	Chromene derivative	[51]
279	9, 12-octadecadienoic acid	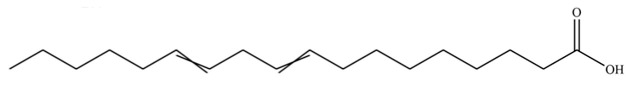	*	*	Fatty acid	[70]
280	palmitic acid	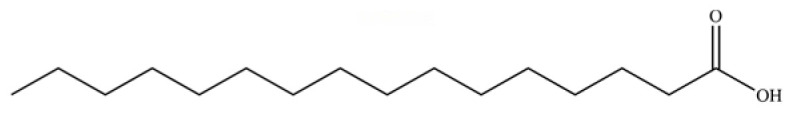	*	*	Fatty acid	[71]
281	α-Muurolene	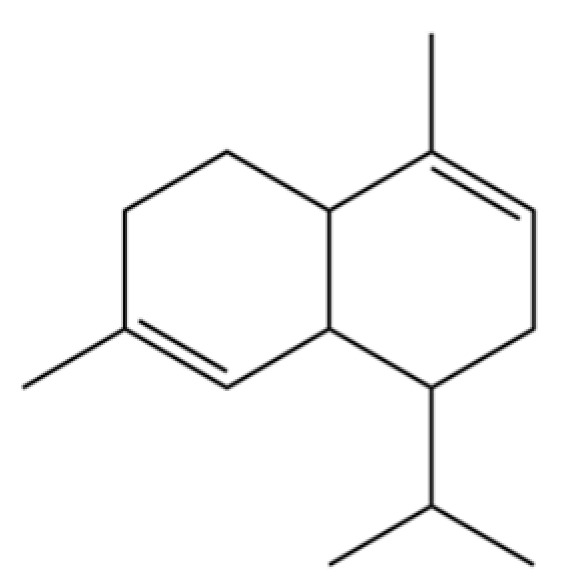	*	*	Sesquiterpenoid	[72]
282	δ-Cadinene	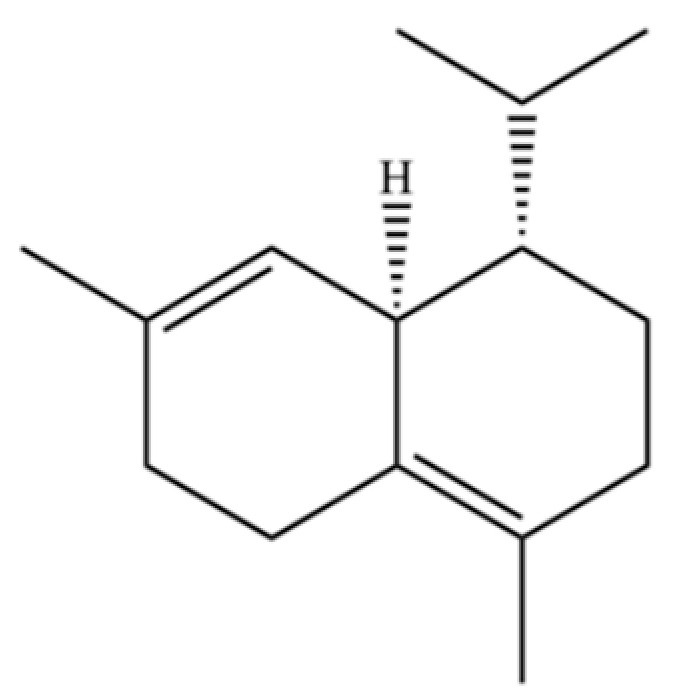	*	*	Sesquiterpenoid	[21]
283	Naphthalene	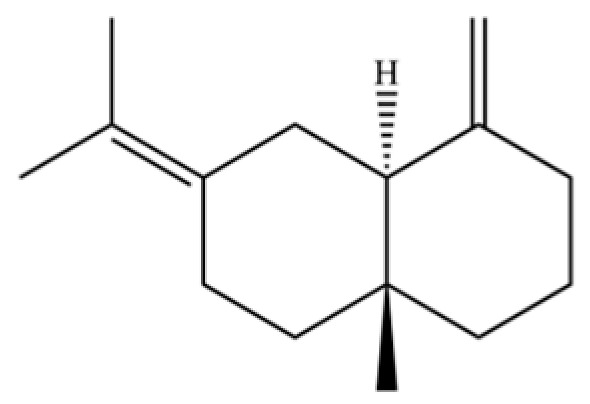	*	*	Aromatic hydrocarbon	[72]
284	Aristolone	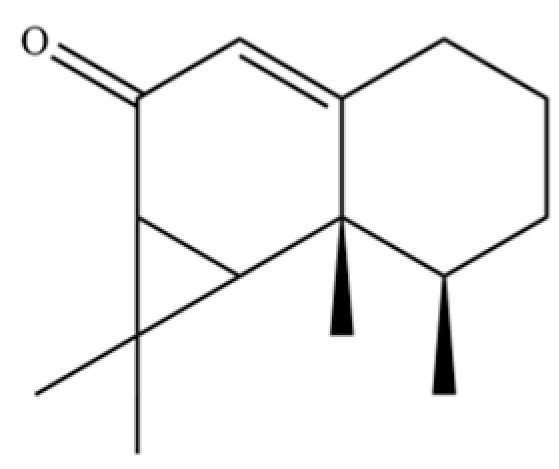	*	*	Sesquiterpenoid	[72]
285	Syringin	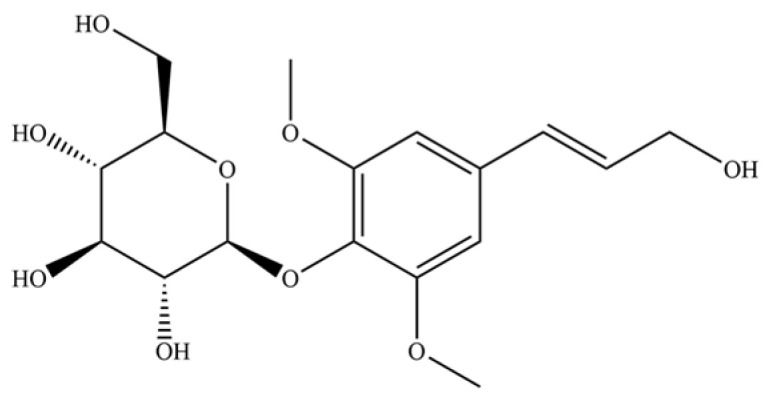	*	*	Phenolic glycoside	[73]
286	Undecanedioic acid	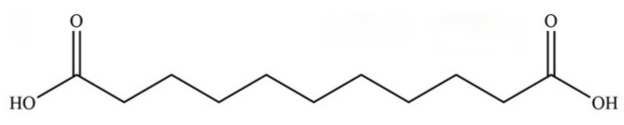	*	*	Dicarboxylic acid	[74]
287	Parthenolide	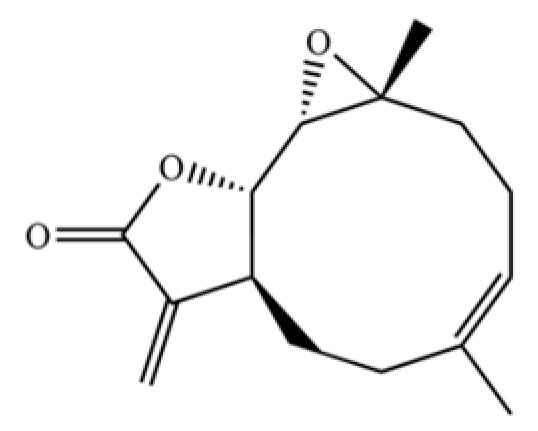	*	*	Sesquiterpenoid lactone	[74]
288	Valerenic acid	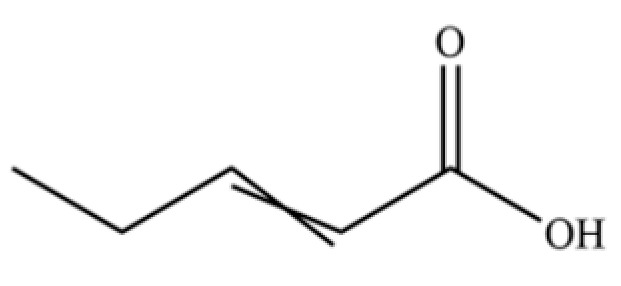	*	*	Sesquiterpenoid acid	[75]
289	3-methyl-1-phenylpent-1-yn-3-ol	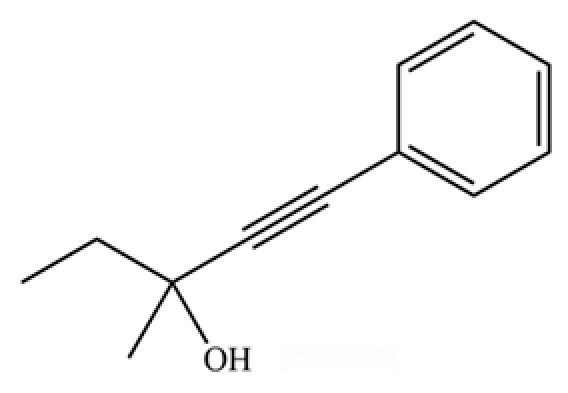	*	*	Aromatic alcohol	[74]
290	α-Cyclohexylmandelic acid	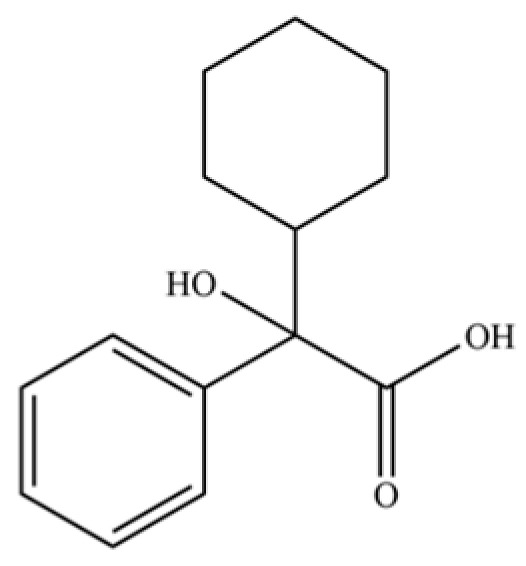	*	*	Aromatic acid	[74]
291	11-propan-2-ylidenetricyclo[4.3.1.12,5]undec-3-en-10-one	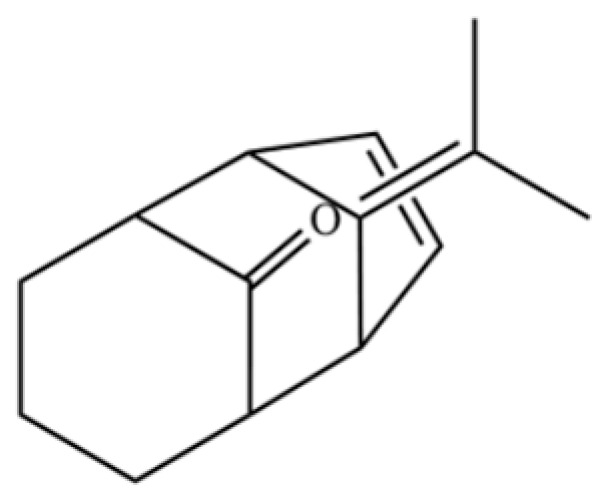	*	*	Sesquiterpenoid	[74]
292	Hydroxyvalerenic acid	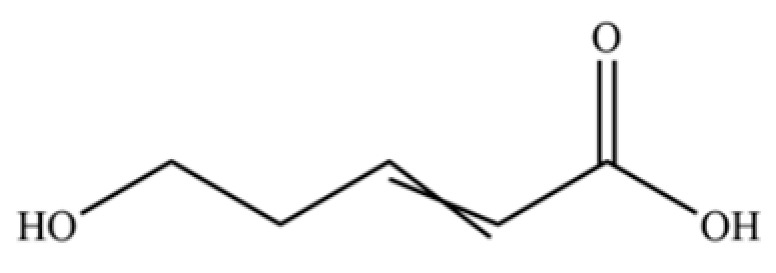	*	*	Sesquiterpenoid acid	[74]
293	3,5-ditert-butylbenzene-1,2-diol	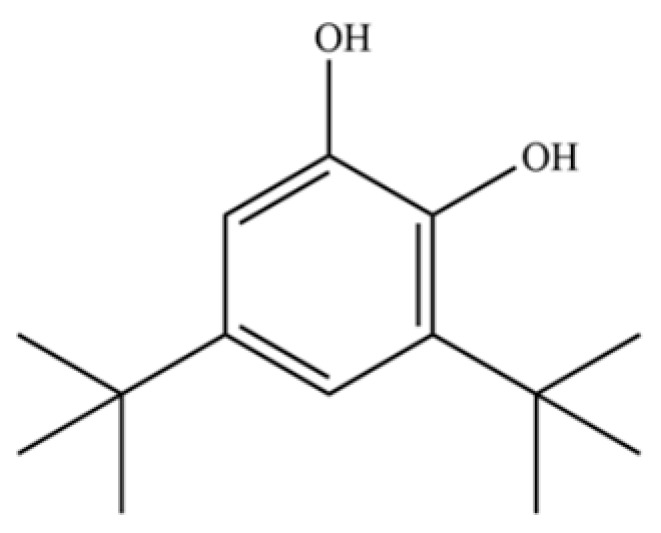	*	*	Phenolic	[74]
294	Senkyunolide A	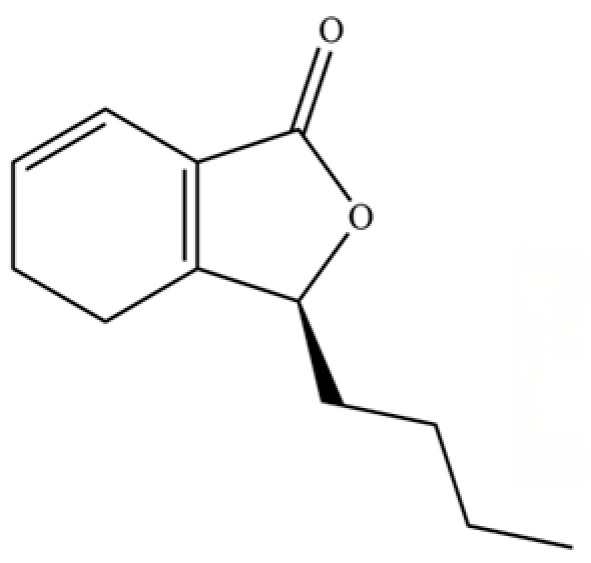	*	*	Phthalide	[74]
295	Nootkatone	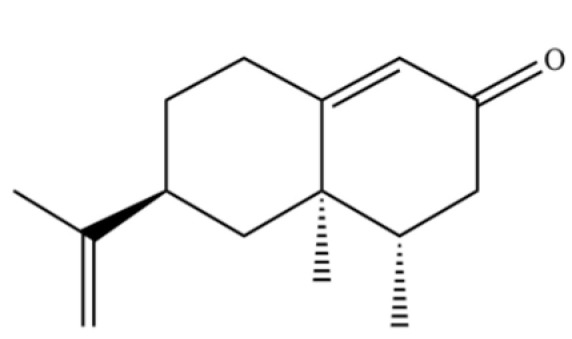	*	*	Sesquiterpenoid ketone	[74]
296	12,13-dihydroxy-9Z-octadecenoic acid	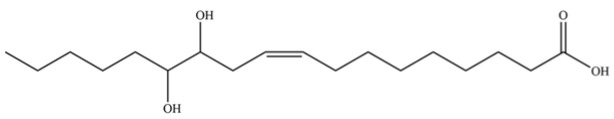	*	*	Fatty acid	[74]
297	2-hydroxyfluorene	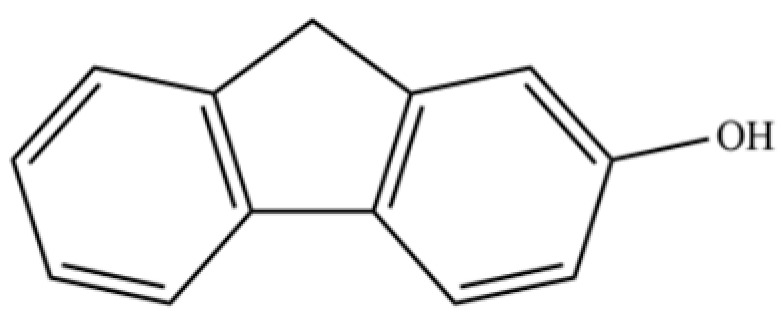	*	*	Phenolic	[26]
298	Isoalantolactone	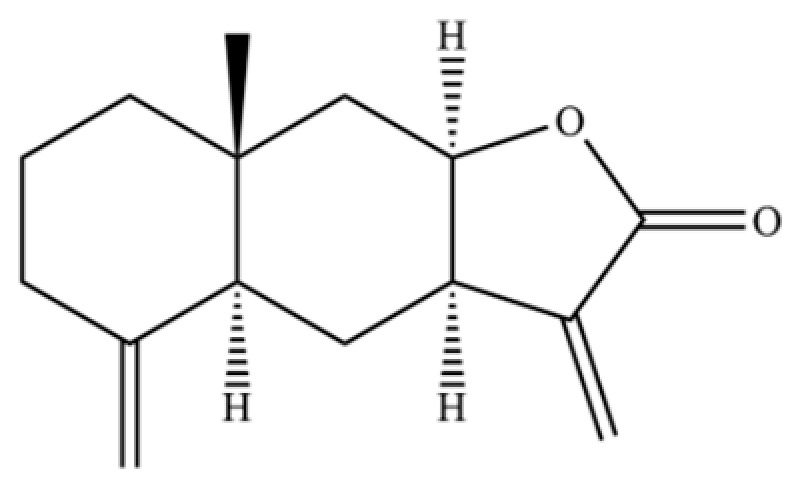	*	*	Sesquiterpenoid lactone	[74]
299	Xanthydrol	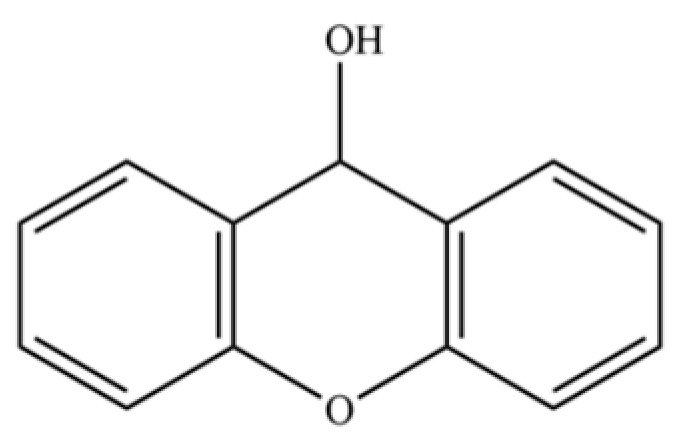	*	*	Xanthene derivative	[74]
300	Bis (4-methoxyphenyl) methanol	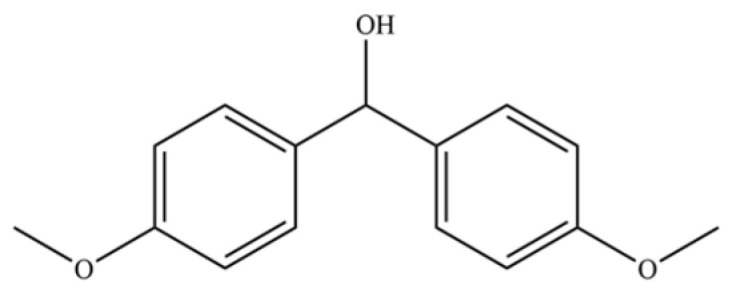	*	*	Aromatic alcohol	[74]
301	1-naphthalenemethanol	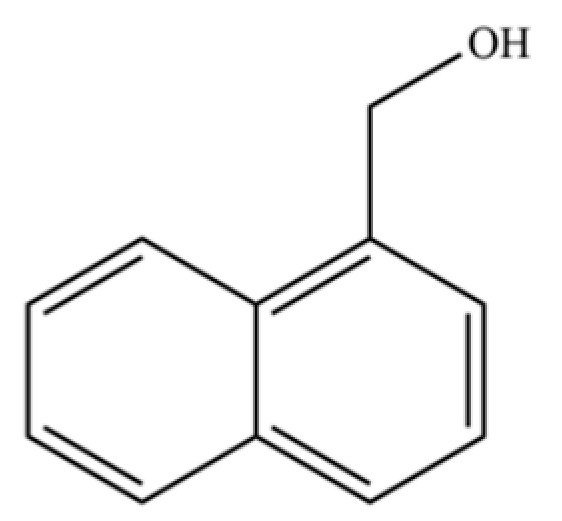	*	*	Aromatic alcohol	[74]
302	2-methylbenzhydrol	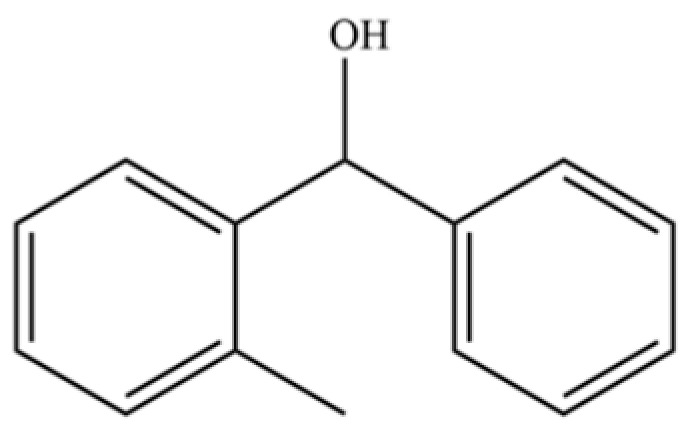	*	*	Aromatic alcohol	[74]
303	3,3′,5,5′-tetramethyldiphenoquinone	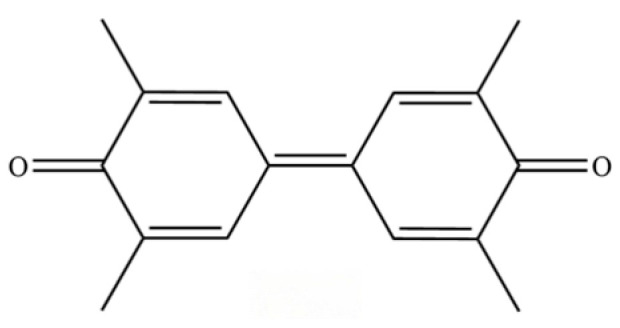	*	*	Quinone	[74]
304	(cis+trans)-nerodilol	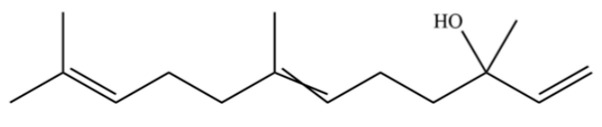	*	*	Sesquiterpenoid	[74]
305	Germacrone	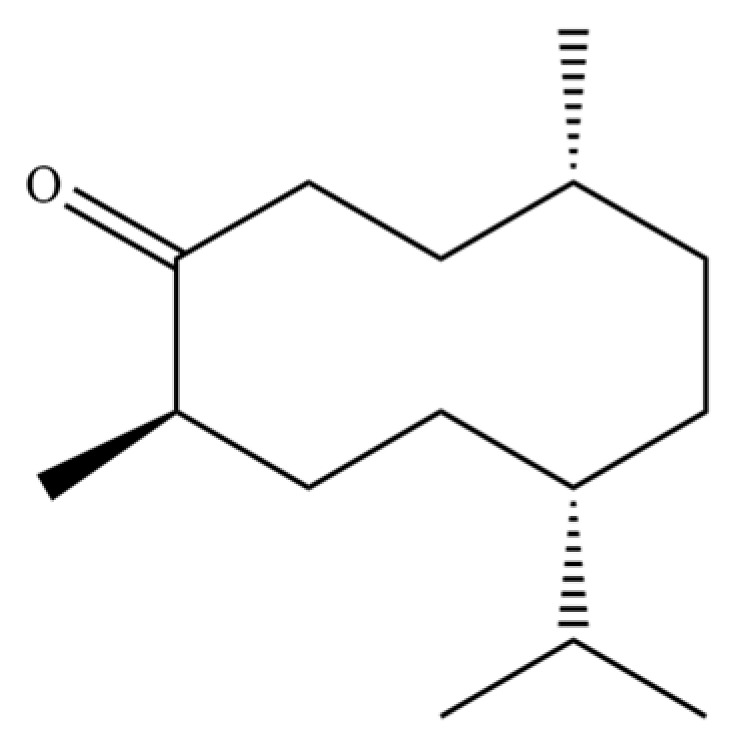	*	*	Sesquiterpenoid	[74]
306	1-palmitoyl-2-hydroxy-sn-glycero-3-phosphoethanolamine	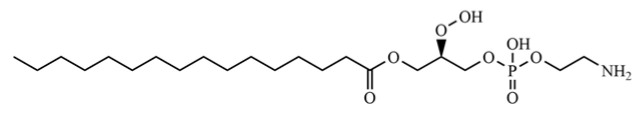	*	*	Phospholipid	[74]
307	1,3-benzenediol, 5-methyl-2-[(1R,6R)-3-methyl-6- (1-methylethenyl)-2-cyclohexen-1-yl]	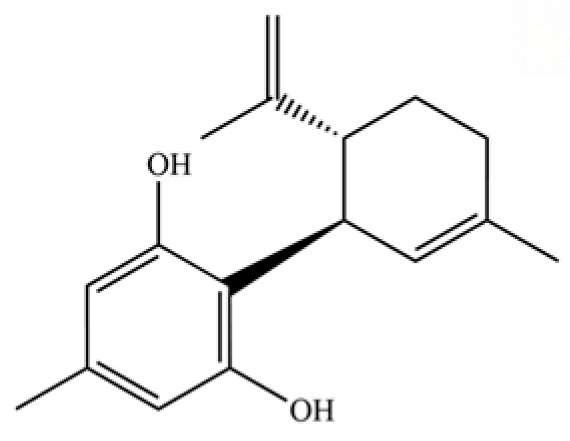	*	*	Phenolic sesquiterpenoid	[74]
308	1-oleoyl-L-α-lysophosphatidic acid	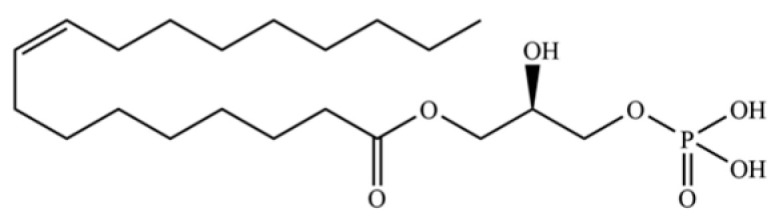	*	*	Phospholipid	[74]
309	2-hydroxypalmitic acid	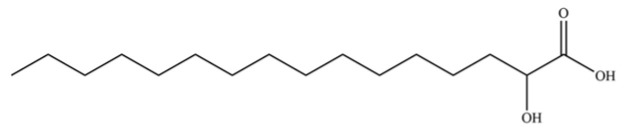	*	*	Fatty acid	[74]
310	Oleamide	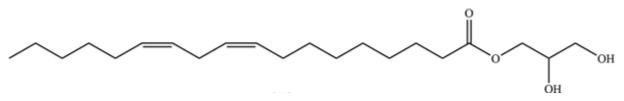	*	*	Fatty amide	[74]
311	9E,11E-octadecadienoic acid	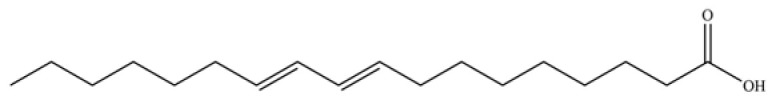	*	*	Fatty acid	[74]
312	1,2-benzenedicarboxylic acid	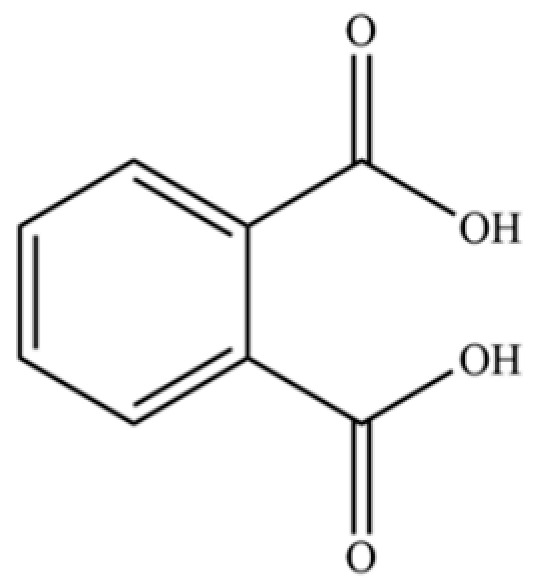	*	*	Phthalic acid (artifact)	[74]
313	Bis (2-ethylhexyl) phthalate	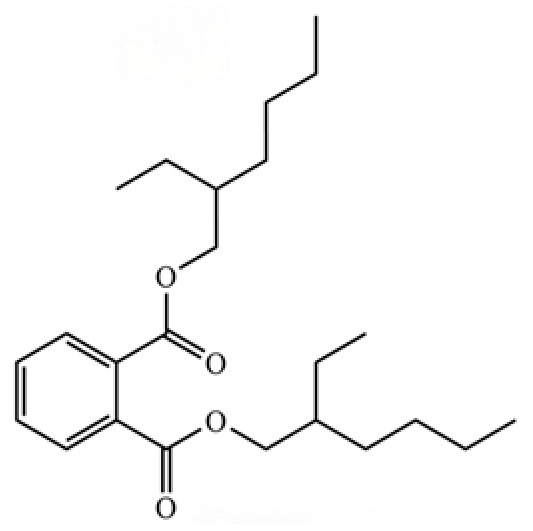	*	*	Phthalate ester (artifact)	[74]
314	Terpinolene	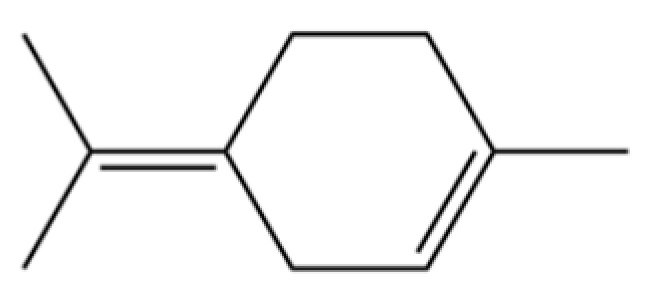	*	*	Monoterpenoid	[21]
315	1-methyl-4-(1-methylethyl)-2-cyclohexen-1-ol	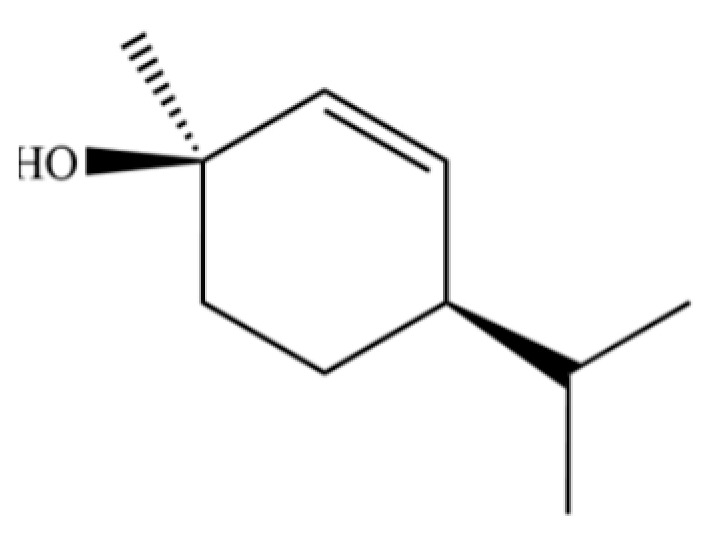	*	*	Monoterpenoid	[76]
316	(1R,3R,5R)-1-Isopropyl-4-methylenebicyclo[3.1.0]hexan-3-ol	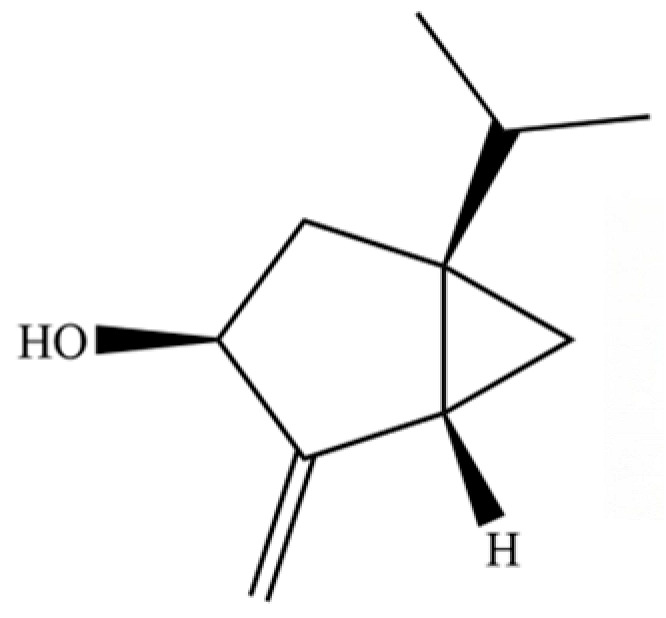	*	*	Monoterpenoid	[76]
317	Methyl geraniate	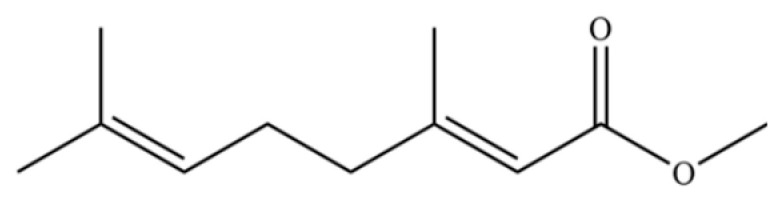	*	*	Monoterpenoid ester	[76]
318	Silphiperfol-5-ene	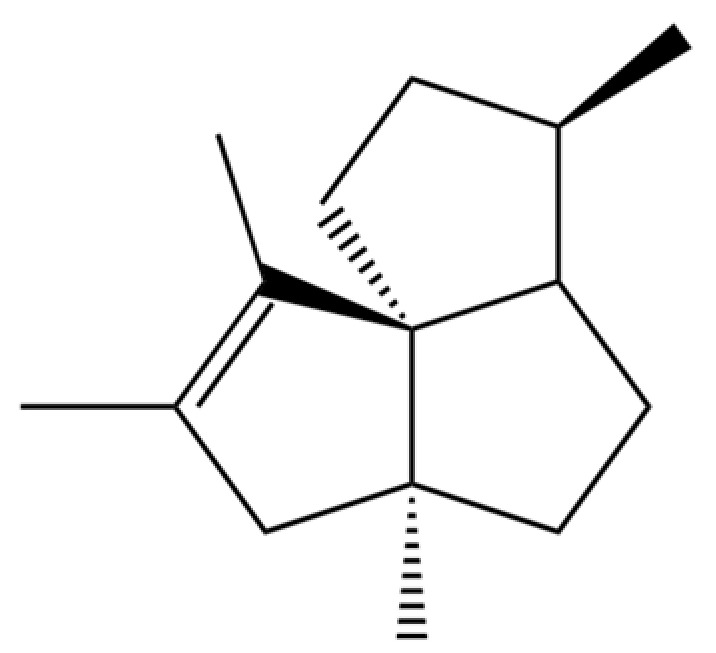	*	*	Sesquiterpenoid	[76]
319	δ-Elemene	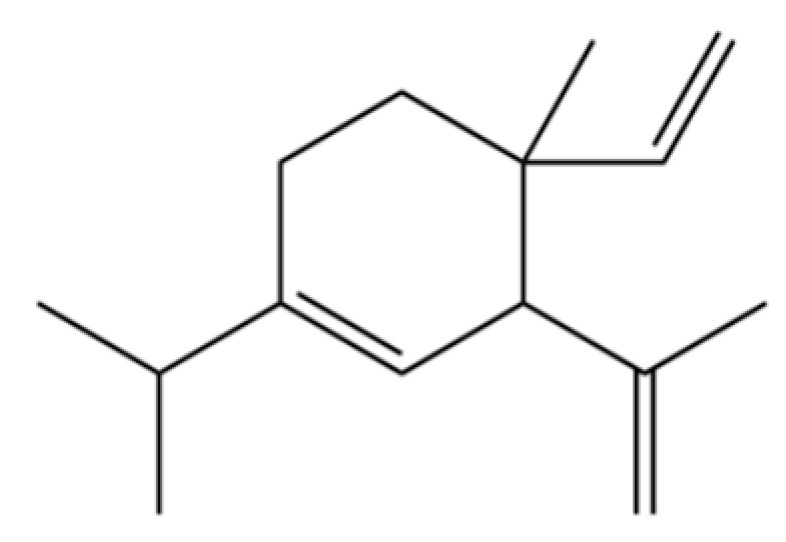	*	*	Sesquiterpenoid	[21]
320	7-epi-Silphiperfol-5-ene	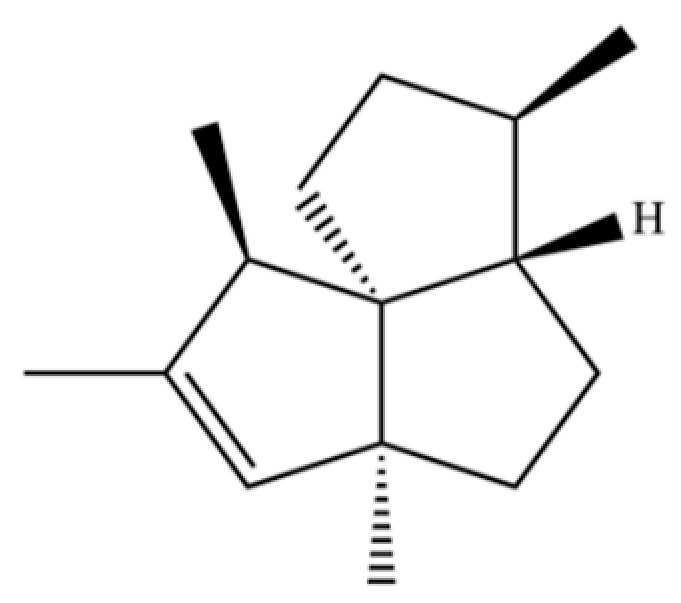	*	*	Sesquiterpenoid	[76]
321	Silphinene	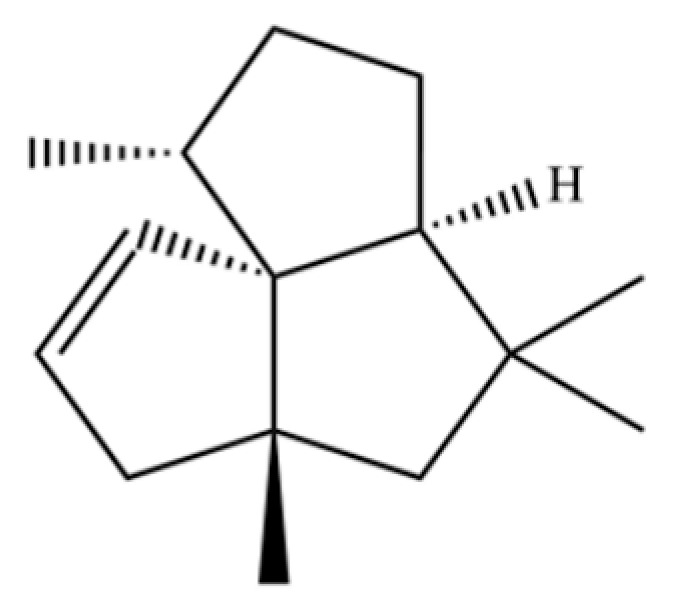	*	*	Sesquiterpenoid	[76]
322	γ-selinene	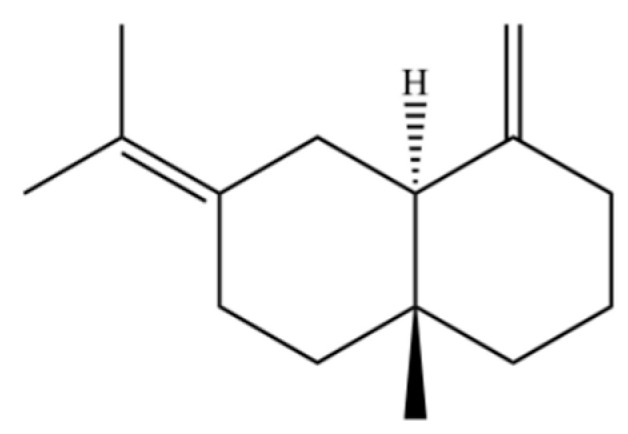	*	*	Sesquiterpenoid	[25]
323	1-Naphthalenol, 1,2,3,4,4a,5,6,7-octahydro-4a,5-dimethyl-3-(1-methylethenyl)-	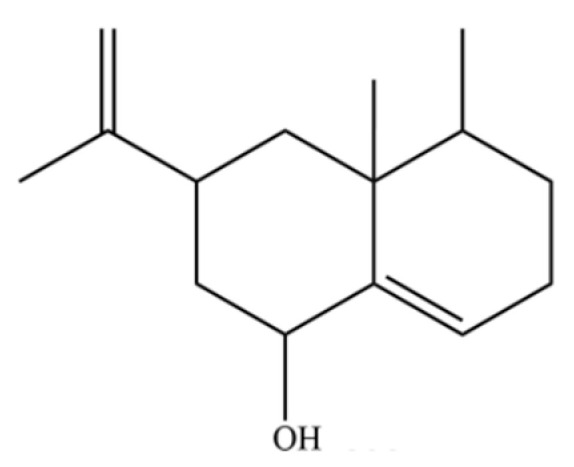	*	*	Sesquiterpenoid	[76]
324	7-epi-alpha-eudesmol	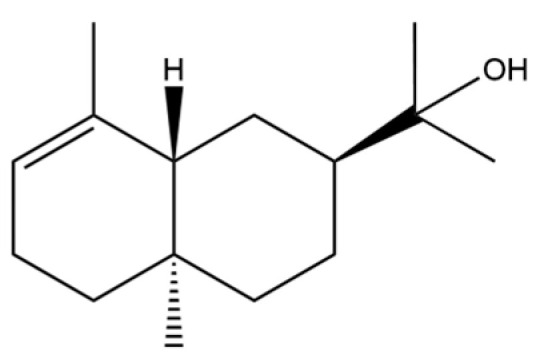	*	*	Sesquiterpenoid	[76]
325	β-guaiene	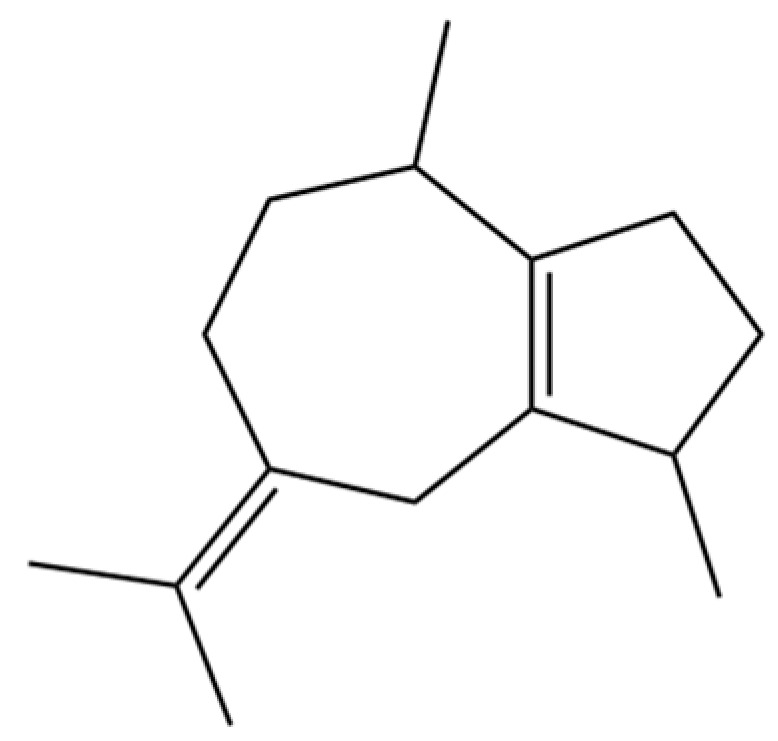	*	*	Sesquiterpenoid	[3]
326	valencene	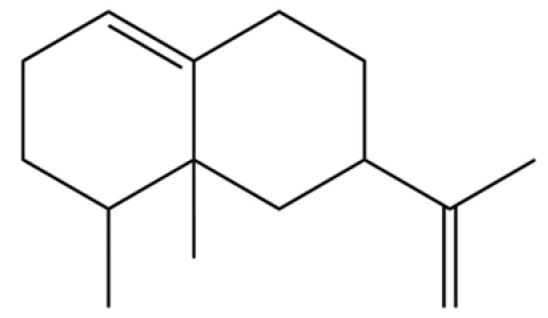	*	*	Sesquiterpenoid	[77]
327	Atrachinenins D	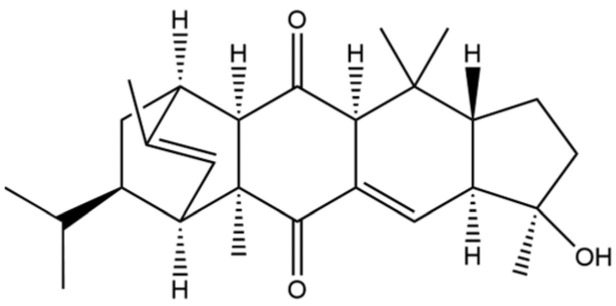	/	**	Sesquiterpenoid	[23]

Note: (1) **: High relative content in the species. (2) *: Present but with lower or unspecified relative content. (3) /: Not reported or considered absent in the species. (4) AL: *Atractylodes lancea*; AC: *Atractylodes chinensis*.

## 5. Pharmacology

### 5.1. Antimicrobial Effect

**Figure 4:**
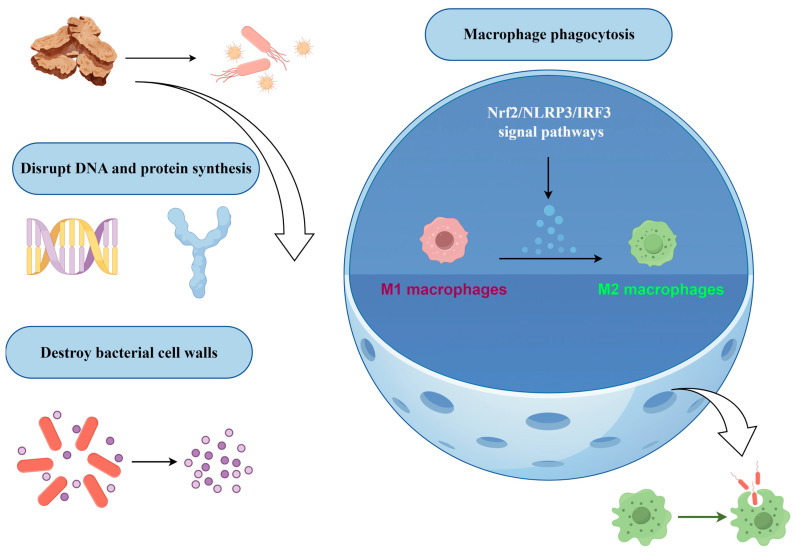
Schematic Diagram of the Antimicrobial Mechanism of Atractylodis Rhizoma. Note: The arrows in the figure indicate interactions; the red areas represent M1 macrophages, and the green areas represent M2 macrophages. In addition, the first row in the upper left corner represents the effects of AR on microorganisms; the second row shows bacterial DNA and proteins; and the third row depicts the disruption of bacterial internal structures. In the right-hand diagram, the small blue spheres in the center represent chemokines, and the bottom row illustrates phagocytosis by macrophages.

AR exhibits notable antimicrobial properties, particularly against bacteria, parasites, and viruses, as illustrated in Table S3 and Figure 4. Studies have demonstrated that the chloroform extract of AR significantly impedes the adhesion and invasion capabilities of Salmonella typhimurium in vitro [78]. A primary factor contributing to the pronounced antibacterial activity of AR is its high concentration of sesquiterpene compounds, such as cyperene, caryophyllene, aciphyllene, and humulene, among others. The antibacterial efficacy of AR is characterized by a multi-mechanism synergistic pattern at both molecular and cellular levels, with its material basis primarily attributed to a diverse array of sesquiterpene compounds. These components interfere with the survival and pathogenic processes of pathogens through distinct molecular pathways, thereby establishing a broad-spectrum antimicrobial system with potential synergistic effects. The core mechanism of its action involves two principal aspects. Firstly, it directly disrupts microbial cell membranes by increasing membrane permeability through the insertion of hydrophobic compounds [79,80]. This disruption results in intracellular electrolyte leakage, the collapse of the transmembrane proton gradient, the efflux of cellular contents, and ultimately, cell death. Additionally, it targets critical enzymes and the synthesis of biomacromolecules. Research has demonstrated that germacrene D (35) effectively inhibits *Staphylococcus aureus* by strongly binding to tyrosyl-tRNA synthetase and topoisomerase II, thereby obstructing protein synthesis and DNA replication [81]. Similarly, molecular docking studies predict that humulene (30) acts on DNA gyrase, disrupting bacterial DNA supercoiling [82]. Furthermore, it interferes with quorum sensing and the expression of virulence factors. For example, α-thujene (143) exhibits high affinity for the virulence regulatory proteins LasR and PqsR in *Pseudomonas aeruginosa*, suggesting its potential to impede bacterial quorum sensing, weaken biofilm formation, and reduce virulence as an anti-virulence agent [83]. Notably, certain components of AR, such as cyperene (21), have been identified for their potential to enhance overall antibacterial efficacy by promoting synergistic interactions among various constituents [84]. Although the aforementioned approach yielded favourable results, the mechanisms described were predominantly derived through virtual screening and computer-aided simulations, necessitating further validation. This finding presents a fresh perspective on tackling the issue of antibiotic resistance frequently linked with single-target antibiotics.

The structure-activity relationships of the active ingredients and their in vivo transformations remain inadequately understood. The majority of research has focused on crude extracts or component mixtures, which complicates the identification of individual contributions. Furthermore, the extrapolation of in vitro antimicrobial activity to in vivo efficacy is constrained by the pharmacokinetic properties and chemical stability of the components, resulting in a substantial deficiency of pertinent data. Consequently, future research should progress beyond mere characterization screening to include experimental validation at functional targets, efficacy evaluation in drug-resistant bacterial models, and rational design based on pharmacophores. This comprehensive approach will facilitate the conversion of the antibacterial potential of AR into novel anti-infective drugs with well-defined mechanisms and clinical translational value.

Beyond its antibacterial properties, AR exhibits diverse biological activities in both antiviral and antiparasitic contexts. Its mechanisms of action encompass various stages of the viral life cycle and critical physiological processes in parasites. Regarding antiviral activity, the active components demonstrate stage-specific intervention capabilities: β-pinene (119) directly influences the adsorption and replication processes of adenovirus type 3 [85]; furanodienone (137) may provide protective effects during the initial phases of viral infection by modulating cell membrane integrity or preserving intracellular reducing conditions [86]; quinic acid (235) derivatives selectively inhibit the intracellular replication of dengue virus without interfering with viral entry [87]; and atractyloside A (243) induces an antiviral immune response by activating the host’s type I interferon signaling pathway (Table S3) [88]. These findings indicate that the antiviral properties of AR exhibit dual characteristics: direct viral suppression and modulation of the host immune response. In terms of antiparasitic activity, component 1-eicosanol (101) demonstrates acaricidal properties that may be associated with its interference in the mite nervous system or its regulation of body wall osmotic pressure [89]. This discovery presents a promising candidate molecule for the development of plant-derived acaricides. However, research in this area is still in the active screening phase, with mechanisms of action primarily based on speculation. Systematic evaluations concerning target confirmation, in vivo efficacy, and environmental safety are currently insufficient. Future investigations should prioritize cross-species and multi-model studies to clarify the specific molecular targets that underlie its antiviral and antiparasitic effects. Furthermore, the potential synergistic effects of its antibacterial components in addressing complex infections or vector-borne diseases warrant exploration. This comprehensive approach will elucidate the scientific value of AR as a multi-target anti-infective medicinal resource.

### 5.2. Anti-Inflammatory Effect

Inflammation is a multifaceted biological response of the body to external stimuli or injury, involving the orchestrated activity of immune cells [90], blood vessels [91], and molecular mediators [92]. It is intricately linked to cell death and significantly influences the development and advancement of prevalent diseases such as cancer [93,94]. Consequently, the development of safe and potent anti-inflammatory medications is crucial. Presently, extensive research is concentrating on the anti-inflammatory properties of individual natural compounds. These compounds exhibit anti-inflammatory actions by inhibiting LOX, suppressing NF-κB or MAPK signaling pathways, and decreasing cytokines like TNF-α and IL-6. Nonetheless, their efficacy is frequently confined to specific targets or models, lacking a systematic, comprehensive regulation of the inflammatory network. To overcome this limitation, Traditional Chinese Medicine, known for its multi-component synergistic effects, presents a distinctive opportunity. As a case in point, AR emerges as a focal point of research, where its diverse array of compounds collectively establish a highly efficient anti-inflammatory network.

**Figure 5:**
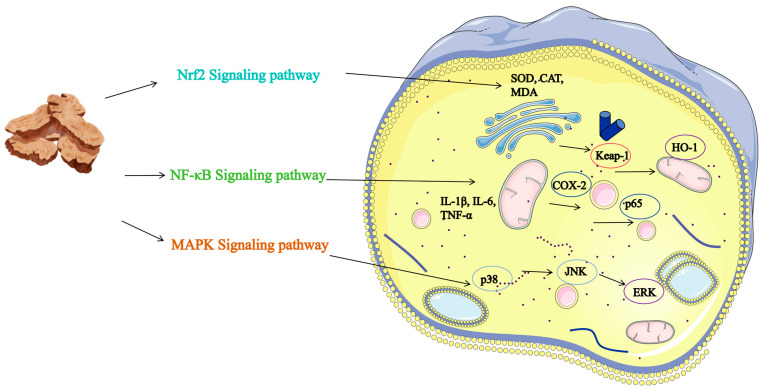
Schematic Diagram of the Anti-inflammatory Mechanism of Atractylodis Rhizoma. Note: The figure illustrates the specific mechanisms of the Nrf2, NF-κB, and MAPK signaling pathways, distinguished by color. The various targets are labeled in the figure, and arrows indicate direct interactions with those targets or pathways.

The anti-inflammatory effects of AR arise from the synergistic integration of its diverse active constituents (refer to Table S4 and Figure 5). For example, the organic acid component, cryptochlorogenic acid (compound 245), has been shown to effectively inhibit the expression of COX-2 and iNOS, while also blocking the activation of the MAPK signaling pathway [95]. Additionally, the terpenoid component, Terpinen-4-ol (compound 131), significantly suppresses the production of pro-inflammatory cytokines, such as TNF-α and IL-6, in macrophages, through the regulation of the MAPK pathway [96]. These principal compounds, in conjunction with polysaccharides and other constituents, concurrently target multiple critical nodes within the inflammatory response. They achieve this by reducing the release of pro-inflammatory factors via inhibition of the TLR4/NF-κB pathway, mitigating oxidative stress through activation of the Nrf2/HO-1 pathway, and modulating interactions between gut microbiota and immune cells. This results in multidimensional regulation of both mucosal immunity and systemic inflammation. This multi-targeted, multi-pathway action model, supported by multiple specific compounds, provides greater regulatory flexibility and systemic stability compared to single-component approaches when addressing complex, chronic inflammatory states.

### 5.3. Antioxidant Effect

**Figure 6:**
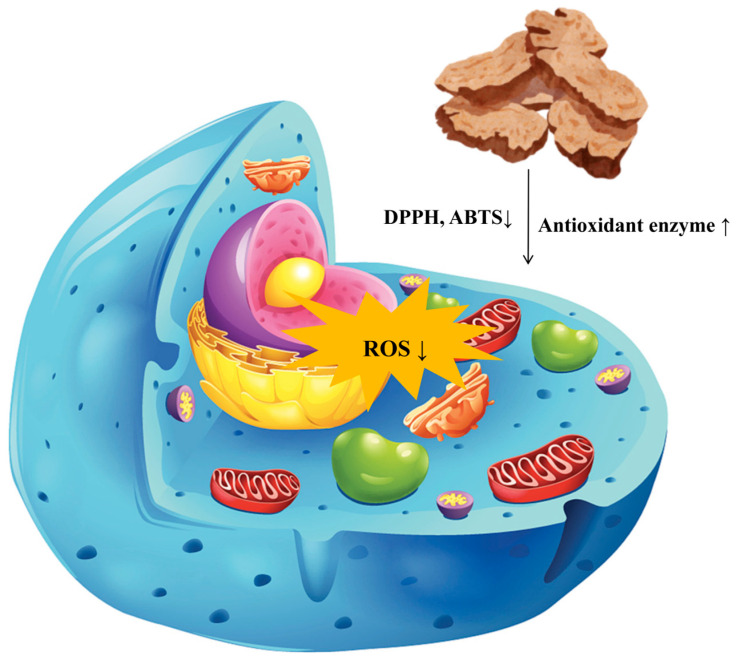
Schematic Diagram of the Antioxidant Mechanism of Atractylodis Rhizoma. Note: This figure illustrates the antioxidant effects of AR. The large arrows indicate where AR acts on cells; ↑ represents an increase, and ↓ represents a decrease. Additionally, the differently colored elements represent organelles affected by oxidative stress, such as mitochondria and the endoplasmic reticulum.

Oxidative stress refers to a condition in living organisms characterized by an imbalance between the production and elimination of reactive oxygen species (ROS), leading to cellular damage [97]. This phenomenon is closely associated with the onset and progression of various diseases, including cardiovascular diseases [98], neurodegenerative disorders [99], and cancer. Therefore, the identification of effective antioxidants is of critical importance. Current research on natural product antioxidants primarily focuses on their ability to directly scavenge free radicals, such as DPPH and ABTS, or to provide reducing equivalents, as evaluated by FRAP and CUPRAC assays [100]. As demonstrated in Table S5 and Figure 6, certain terpenoid and phenolic derivatives exhibit significant activity in chemical models, with effects comparable to those of ascorbic acid. However, their mechanisms of action are predominantly confined to the downstream stage of direct neutralization. Furthermore, their efficacy and stability within complex biological systems are often compromised by low concentrations and rapid metabolism. In contrast, the sesquiterpene lactone components characteristic of AR, which constitute the primary focus of this study, demonstrate a more profound and physiologically relevant mechanism of action. Experimental evidence substantiates that atractylenolactam (258) significantly activates the intracellular Nrf2 antioxidant signaling pathway. Nrf2 serves as a pivotal transcription factor governing cellular defenses against oxidative stress; its activation systematically induces the expression of various phase II detoxification enzymes, such as heme oxygenase-1 (HO-1) and nicotinamide adenine dinucleotide phosphate quinone dehydrogenase 1 (NQO1), alongside endogenous antioxidant enzymes [101]. This finding suggests that the antioxidant effects of AR transcend mere passive radical scavenging, actively augmenting the cells’ intrinsic defense mechanisms. Consequently, this provides a more robust molecular foundation for achieving sustained, broad-spectrum protection against oxidative damage, thereby demonstrating significant advantages, particularly in addressing persistent oxidative stress associated with chronic diseases.

Furthermore, the antioxidant properties of AR are significantly augmented by the synergistic interactions among its diverse constituents. The data presented in the Table S5 suggest that while the activity of individual compounds may be limited, natural essential oils or extracts frequently demonstrate enhanced overall activity due to the synergistic interactions among their components. AR is abundant in various sesquiterpene lactones, such as atractylenolide I (271), II (268), and III (251), in addition to polyacetylene compounds, such as atractylodin (141). These constituents may engage in network pharmacologic effects through distinct molecular targets and mechanisms of action, resulting in synergistic or additive antioxidant effects. This complex natural defense system, comprising multiple components, poses a greater challenge for the body to compensate for or circumvent compared to a single compound, thereby potentially providing more stable and comprehensive protection. Consequently, AR functions not only as a source of highly active lead compounds but also as a standardized extract. As a natural preparation capable of multi-targeted, systemic regulation of endogenous antioxidant pathways, it exhibits unique potential and broad application prospects in the development of herbal medicines and functional foods aimed at the prevention or adjunctive treatment of oxidative stress-related diseases.

### 5.4. Hepatoprotective Effect

**Figure 7:**
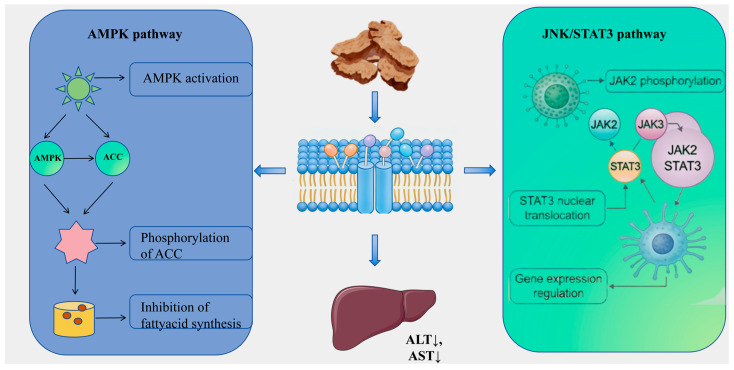
Schematic Diagram of the Hepatoprotective Mechanism of Atractylodis Rhizoma. Note: ↑ represents an increase, and ↓ represents a decrease.

Liver injury, characterized as a complex pathological process arising from various etiological factors, has historically posed significant challenges in pharmacological research, particularly in the formulation of effective prevention and treatment strategies. Conventional single-target interventions frequently prove inadequate in addressing the extensive array of pathways implicated in the initiation and progression of liver injury, which include oxidative stress, lipid metabolism disorders, inflammatory responses, and fibrosis [102]. Within this framework, AR and its diverse bioactive constituents demonstrate considerable hepatoprotective potential and systemic intervention benefits, attributable to their multi-component and multi-target action properties. Emerging research [103] suggests that the hepatoprotective effects of AR are achieved through synergistic regulation across various stages and pathological dimensions of liver injury (refer to Table S6 and Figure 7). In the initial phase of injury, bioactive compounds such as atractylodin (141) and neochlorogenic acid (240) effectively inhibit fatty acid synthesis and promote its oxidation through activation of the AMPK pathway and upregulation of PPARα/CPT-1 expression [104,105]. This modulation of lipid metabolism ameliorates metabolic disorders and addresses the underlying pathology of non-alcoholic fatty liver disease. Simultaneously, compounds such as eucalyptol (115) attenuate drug- or toxin-induced oxidative stress-related liver damage by significantly enhancing the activity of endogenous antioxidant enzymes, including glutathione (GSH) and superoxide dismutase (SOD), while reducing oxidative damage markers such as malondialdehyde (MDA) and 8-hydroxy-2′-deoxyguanosine (8-OHDG) [106]. Moreover, derivatives of AR demonstrate significant potential in mitigating the malignant progression associated with liver damage. Notably, Senkyunolide A (294) alleviates cholestatic liver fibrosis through the modulation of endoplasmic reticulum autophagy, while Germacrone impedes the JAK2/STAT3 signaling pathway, thereby inhibiting hepatic stellate cell activation and collagen deposition [107]. Furthermore, the biomolecules such as Atractylodin (141) and *Atractylodes* polysaccharide present in AR have been shown to exhibit substantial hepatoprotective properties [108]. This broad spectrum of actions, which includes metabolic regulation, antioxidant defense, and anti-fibrotic effects, provides comprehensive protection against the progressive pathological trajectory of liver injury.

### 5.5. Anti-Cancer Effect

**Figure 8:**
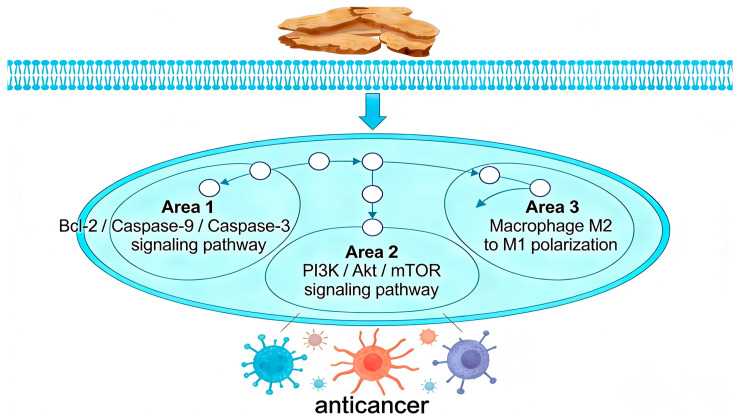
Schematic Diagram of the Anti-cancer Mechanism of Atractylodis Rhizoma. Note: The arrows indicate how AR exerts its anticancer effects through apoptosis, cell proliferation, and macrophage polarization, respectively. The tumor cells of different colors represent the outcomes resulting from these three mechanisms, including apoptosis, growth inhibition, and phagocytosis.

This study provides a comprehensive review of the diverse bioactive compounds found in AR, with a specific focus on their anticancer properties as detailed in Table S7 and Figure 8. Among these compounds, sesquiterpene lactones, including atractylenolide II (268), atractylenolide I (271), and guaiol (191), are identified as the primary active constituents. These compounds demonstrate significant efficacy in modulating key signaling pathways, such as PI3K/Akt/mTOR, STAT3, and ERK. Furthermore, volatile oils and terpenoid components, such as β-eudesmol (71) and δ-elemene (319), play a crucial role in directly inducing tumor cell apoptosis and cell cycle arrest. Phenolic acid derivatives such as Chlorogenic acid (242) further augment these effects by modulating oxidative stress and immune responses. The diversity of these components does not operate in isolation; rather, they collectively contribute to the comprehensive capacity of AR to intervene in tumor biology through multiple targets and at various levels.

The notable anti-cancer properties of AR are attributed to its extensive regulation of both the tumor microenvironment and systemic physiology, extending beyond mere direct cytotoxic effects. The primary component, atractylenolide II, exemplifies this characteristic by modulating the immune microenvironment across various cancer types, specifically through the inhibition of M2 macrophage polarization and the downregulation of PD-L1. Additionally, it participates in emerging biological processes such as glycolytic metabolism and the induction of ferroptosis [109,110]. This complex mechanism of a single molecule suggests that its target may be located at an upstream node in the bidirectional regulation between tumor cells and the microenvironment. Furthermore, the therapeutic potential of AR also addresses the systemic manifestations of tumor diseases. For instance, atractylenolide I alleviates cancer cachexia-related muscle wasting by inhibiting the STAT3 pathway [111]. This aligns with the principles of Traditional Chinese Medicine, which focus on fortifying the spleen, enhancing qi, and bolstering the body’s vital energy. It embodies a unique holistic therapeutic approach that concurrently eliminates pathogens and strengthens the body’s defenses.

### 5.6. Anti-Diabetic Effect

The anti-diabetic potential of AR is notably significant due to its direct hypoglycemic effects, which influence essential pathways of glucose metabolism through synergistic multi-component mechanisms (Table 3). Recent studies indicate that its active constituents can modulate blood glucose regulation through multiple targets. For example, molecular docking analyses have confirmed that γ-cadinene (42), a sesquiterpene component abundant in its essential oil, exhibits a strong binding affinity with the insulin receptor (INSR). This observation suggests that γ-cadinene (42) may mimic or enhance insulin signaling, thereby directly mitigating insulin resistance, which is a critical mechanism for lowering blood glucose levels [112]. Simultaneously, the sesquiterpene constituents of AR essential oil, such as eucalyptol (115) and compounds structurally similar to valeranone (60), may enhance endogenous insulin secretion and utilization by protecting pancreatic beta cells and inhibiting dipeptidyl peptidase-IV (DPP-IV) enzyme activity, respectively [113,114]. Collectively, these mechanisms fortify the insulin axis. Additionally, the extract’s potential α-glucosidase inhibitory activity may directly impede carbohydrate absorption in the intestine, thereby enabling rapid management of postprandial blood glucose surges. This simultaneous intervention across three pivotal pathways—insulin sensitivity, insulin secretion, and postprandial blood glucose regulation—constitutes the primary advantage of AR’s direct hypoglycemic effect.

In a more comprehensive context, the hypoglycemic properties of AR are intricately linked to both the prevention and treatment of diabetic complications, thereby demonstrating dual benefits in glucose metabolism regulation and tissue protection. Specific constituents, such as eucalyptol (115), play a crucial role in mitigating retinal pigment epithelial barrier dysfunction in diabetic models by downregulating matrix metalloproteinases (MMPs) and reducing apoptosis and oxidative stress, as evidenced by decreased reactive oxygen species (ROS). This underscores its unique value in preventing diabetic retinopathy [114]. Additionally, phenolic acid components, such as cryptochlorogenic acid (245), may inhibit ferroptosis in pancreatic β-cells by activating the Nrf2/GPX4 pathway, thereby providing sustained protection for pancreatic function [119]. This synergistic effect, which includes the concurrent reduction in blood glucose levels along with antioxidant and anti-inflammatory benefits, as well as the protection of microvasculature and vital organs, elevates AR beyond the role of a mere hypoglycemic agent.

### 5.7. Intestinal Regulatory Function

**Figure 9:**
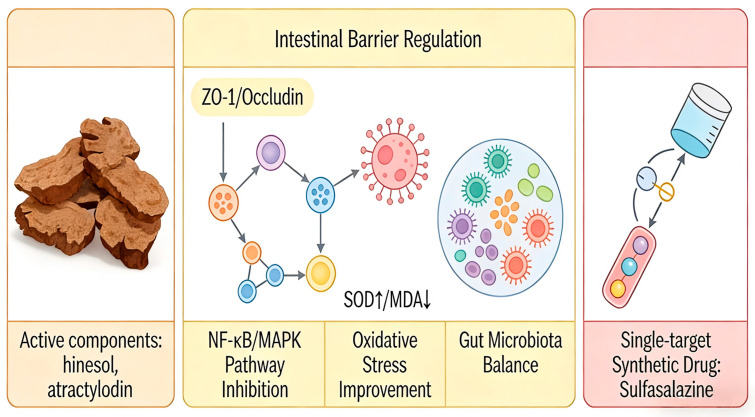
Schematic Diagram of the Intestinal regulatory Mechanism of Atractylodis Rhizoma. Note: Light orange modules represent AR; light yellow modules indicate that AR acts through four mechanisms simultaneously, with different arrows denoting multi-pathway action; light red modules indicate that synthetic drugs have only one arrow, representing a single target. Additionally, the different colors in the central region represent the interactions among the intestinal barrier, the gut microbiota, and antioxidant enzymes, illustrating AR’s multidimensional regulatory capabilities.

Recent research on ulcerative colitis (UC) and associated intestinal inflammation has increasingly concentrated on natural products, owing to their multi-target effects and favorable safety profiles [121]. Empirical evidence indicates that AR contains bioactive compounds such as sesquiterpenes, polyacetylenes, and phenolic acids, which have demonstrated efficacy in preclinical colitis models (refer to Table S8 and Figure 9). These compounds collectively reduce the disease activity index (DAI) and enhance the integrity of the intestinal barrier, as evidenced by the upregulation of tight junction proteins, including ZO-1, occludin, claudin-1, and MUC2 [122]. Furthermore, they inhibit key pro-inflammatory signaling pathways, notably NF-κB and MAPK. In addition, these compounds modulate oxidative stress markers by elevating levels of SOD, GSH-Px, and CAT, while decreasing MDA levels, and they contribute to the restoration of gut microbiota balance [123]. This multifaceted mechanism of action stands in contrast to the single-target approach of synthetic drugs such as sulfasalazine or mesalazine.

The primary benefit of AR lies in its synergistic and multi-dimensional strategy for addressing intestinal inflammation. Unlike monotherapies that typically target singular pathways, the phytochemical constituents of AR concurrently tackle multiple pathological aspects of ulcerative colitis, such as barrier dysfunction, immune dysregulation, oxidative damage, and microbial dysbiosis. For instance, atractylodin (141) not only inhibits the activation of NF-κB and MAPK pathways but also fosters the growth of beneficial gut microbiota and influences metabolic regulation through GAPDH malonylation [122]. Similarly, hinesol (66) and atractylenolide III (251) collaboratively enhance tight junction integrity while downregulating various cytokines and chemokines [123,124]. This multi-target approach may enhance therapeutic efficacy and reduce the risk of compensatory resistance, offering a treatment strategy that aligns with the principles of “network pharmacology” inherent in herbal medicine.

### 5.8. Neuroprotective Effect

Research on AR within the realm of neuroprotection is transitioning its role from a traditional medicinal herb to a comprehensive natural compound library with well-defined multi-target mechanisms. Unlike the numerous single compounds documented in the literature, the significance of AR lies in its diverse array of bioactive constituents, including polyacetylenes and sesquiterpene lactones, which collectively form a more extensive neuroprotective network (see Table 4). For instance, its primary component, atractylodin (141), demonstrates anti-inflammatory and antioxidant properties [103]; atractylenolide III (251) modulates astrocyte function [125]; and atractylenolactam (258) suppresses microglial activation [126]. Together, these elements collectively form a functionally synergistic composite system, whose combined effects exceed those of the individual compounds listed in the table. For instance, Linalool primarily addresses oxidative stress, β-Phellandrene specifically inhibits acetylcholinesterase (AChE), and Parthenolide selectively modulates certain inflammatory pathways. This inherent multi-component, multi-pathway synergistic capability confers upon AR an augmented potential for systematic intervention in complex pathologies characterized by multiple interrelated factors, such as neurodegenerative diseases.

To fully capitalize on this advantage, it is essential for research to make substantial advancements across several domains. Mechanistic investigations should transition from a focus on individual pathways to employing computational pharmacology and multi-omics approaches. This will facilitate a comprehensive understanding of how atractylodin (141), atractylenolides, and other critical components of AR interact to modulate the “neuroinflammation-oxidative stress-pyroptosis-autophagy” network. In the realm of formulation development, insights should be drawn from the successful implementation of citronellyl acetate nanoparticles and rutin-AuNPs. Given the characteristics of the active constituents, it is crucial to design innovative nanoscale systems capable of co-loading multiple components to improve brain delivery, thereby overcoming challenges related to bioavailability and targeted delivery [127,128]. Furthermore, validation models for therapeutic efficacy need to evolve beyond single-injury paradigms to establish animal models that more accurately represent the complexity of clinical diseases and the pathomechanism of spleen deficiency with dampness obstruction, as conceptualized in Traditional Chinese Medicine. These models should integrate variables such as aging, metabolic abnormalities, and neuroinflammation.

**Table 4:** Neuroprotective effect of Atractylodis Rhizoma.

Bioactivity	Compounds/Extracts	Testing Subjects (Animal/Model)	Dose	Positive Control	Results/Mechanism	References
Anti-anxiety	Dehydrofukinone (75)	Swiss mice	10, 22, 30, 100 mg/kg	Diazepam	GABAₐ receptor activity ↑, Chloride ion influx ↑, Voltage-dependent calcium channel activity ↓, Free calcium concentration ↓	[129]
Neuroprotective effect	Isoaromadendrene epoxide (76) from essential oil	In vitro: PC12 and BV2 cells; In vivo: C57BL/6 mice	In vitro: 0.1–10 µg/mL; In vivo: 10–20 mg/kg	Memantine	IL-1β ↓, IL-6 ↓, TNF-α ↓, GABA ↑, 5-HT ↑, NE ↑	[130]
Neuroprotective effect	Atractylodin (141)	C57BL/6J mice	20 mg/kg and 40 mg/kg	Not mentioned	TNF-α ↓, IL-6 ↓, IL-1β ↓, NLRP3 ↓, NF-κB ↓, BDNF ↑, Akt ↑, MDA ↓, SOD ↑, GSH-Px ↑, GSH ↑	[103]
Neuroprotective effect	Eucalyptol (115)	Wistar rats	100 mg/kg/d	Not mentioned	MDA ↓, GSH ↑, SOD ↑, GPx ↑, TNF-α ↓, IL-1β ↓, IL-6 ↓, IL-10 ↑, SIRT1 ↑, NF-κB ↓, BDNF ↑,	[131]
Neuroprotective effect	Linalool (117)	PC12 Cell	1–100 µmol/L	Not mentioned	ROS ↓, MDA ↓, DNA ↓, Bax/Bcl-2 ↓, caspase-3 ↓, caspase-9 ↓	[132]
Neuroprotective effect	β-Phellandrene (120)	Enzyme inhibition assay in vitro	300 µg/mL	Not mentioned	AChE ↓	[133]
Anti-aggregation and disaggregation effects of Aβ amyloid protein	Citronellyl acetate (125) loaded onto CaCO_3_ nanoparticles	In vitro simulation	100 µg/mL	BHT, Donepezil, Galantamine	AChE ↓, Aβ aggregation ↓	[134]
Anti-aggregation and disaggregation effects of Aβ amyloid protein	Citronellyl acetate (125) loaded onto hydroxyapatite nanocarriers	In vitro simulation	100 µg/mL	BHT, Donepezil, Galantamine	AChE ↓, Aβ aggregation ↓	[127]
Neuroprotective effect	(-)-Bornyl acetate (126)	C57BL/6 mice	100, 200, 400 mg/kg	Not mentioned	CD11b^+^/CD45^+^ Cell ↓, IL-1β ↓, IL-6 ↓, TNF-α ↓, iNOS ↓, COX-2 ↓, MCP-1 ↓, MIP-1α ↓, RANTES ↓, p38 ↓, ERK ↓, NF-κB ↓, ZO-1 ↑, Occludin ↑, PECAM-1 ↓, MMP-9 ↓, ICAM-1 ↓	[135]
Anti-neuroinflammatory effect	Carveol (146)	SD rats	5, 10, 20 mL/kg	Not mentioned	Alleviate thermal pain sensitivity and improve motor coordination, GSH ↑, GST ↑, iNOS ↓, LPO ↓, NF-κB ↓, COX-2 ↓, TNF-α ↓	[136]
Alleviating neuroinflammation and pain in diabetes	Carveol (146)	SD rats	5, 10, 20 mL/kg	Pregabalin	GSH ↑, GST ↑, LPO ↓, NO ↓, COX-2 ↓, TNF-α ↓, NF-κB ↓	[137]
Improving memory impairment and neuroinflammation	Carveol (146)	SD rats	50, 100, 200 mg/kg	Donepezil	GSH ↑, GST ↑, CAT ↑, LPO ↓, Aβ ↓, NF-κB ↓, TNF-α ↓, IL-18 ↓, PGE2 ↓	[138]
Improving Parkinson’s disease	Carveol (146)	Albino mice	50 mg/kg	Not mentioned	ROS ↓, LPO ↓, Nrf2 ↑,HO-1 ↑, NLRP3 ↓	[139]
Improving Alzheimer’s disease	Quinic acid (235)	SH-SY5Y cell	50–200 µM	Donepezil	ROS ↓, Aβ ↓, phosphorylated tau ↓, MAPK ↓	[140]
Promoting the proliferation and differentiation of neural stem cells	Quinic acid (235)	Hippocampal cell	50, 100, 200 µM	Not mentioned	Mash1 ↑, Ngn2 ↑, Notch1 ↑, Hes1 ↑	[141]
Improving Alzheimer’s disease	Quinic acid (235)	C57BL/6 mice	30 mg/kg/d	Not mentioned	IAA ↑, KYNA ↑, DR3 ↓, IKK ↓, NF-κB ↓, Aβ42 ↓, p-Tau ↓	[142]
Improving Alzheimer’s disease	Corogenic acid (242)	C57 mice	2 mg/kg	Not mentioned	Aβ ↓, BACE1 ↓, TNF-α ↓, IL-1β ↓, IL-6 ↓	[143]
Neuroprotective effect	Rutin (250)-AuNPs	In vitro: SH-SY5Y cells, In vivo: ICR mice	20–100 µg/mL	Not mentioned	Nrf2 ↑, ARE ↑, ROS ↓	[128]
Improving Alzheimer’s disease	Atractylenolide III (251)	In vitro: C8-D1A astrocytes, In vivo: C57BL/6 mice	In vitro: 100 µM; In vivo: 2.4 mg/kg	Not mentioned	AQP4 ↑	[125]
Neuroprotective effect	Atractylenolactam (258)	In vitro: Bv2 microglia, HT22 neuron cells	50 µM	Not mentioned	TNF-α ↓, IL-6 ↓, IL-1β ↓	[126]
Antidepressant effect	α-cyperone (273)	In vivo: Wistar rats	5, 10 mg/kg	Not mentioned	IL-1β ↓, TNF-α ↓, p65 phosphorylation ↓	[144]
Improving Parkinson’s disease	α-cyperone (273)	Wistar rats	10 mg/kg	Not mentioned	Nrf2 ↑, HO-1 ↑, NF-κB ↓	[145]
Alleviating brain ischaemic damage	Parthenolide (287)	In vivo: Sprague Dawley rats; In vitro: The human microglial clone 3 cell	In vivo: 0.5 mg/kg, 1 mg/kg; In vitro: 2 µM	Gination	RhoA ↓, ROCK ↓, NF-κB ↓	[146]
Improving neurological function following traumatic brain injury	Parthenolide (287)	In vivo: C57BL/6 mice; In vitro: Bv2 microglia, HT22 neuron cells	In vivo: 1 mg/kg; In vitro: 0.5–5 µM	Not mentioned	STAT3 ↓, NF-κB ↓, NLRP1 ↑, NLRP3 ↑, NLRC4 ↑	[147]
Improve cognitive impairment	leamide (310) from Ethanolic Extract of *Rosa rugosa* Roots	ICR mice	10, 20, 40 mg/kg	Not mentioned	MDA ↓, ADAM10 ↑	[148]

Note: ↑ represents an increase, and ↓ represents a decrease.

### 5.9. Cardiac-Protective Effect

AR, a traditional medicinal plant, has been shown in recent studies to contain a diverse array of bioactive compounds with cardioprotective potential. These compounds exhibit synergistic effects through multiple targets and pathways, highlighting their significant value in the prevention and treatment of cardiovascular diseases (see Table 5). The compounds listed in this table, including camphene (121), cryptochlorogenic acid (245), atractylenolide II (268), valerenic acid (288), and valencene (327), can primarily be isolated directly from this plant. These constituents enhance antioxidant capacity in animal and cellular models by increasing the ratios of superoxide dismutase (SOD), catalase (CAT), and glutathione/glutathione disulfide (GSH/GSSG). They also suppress oxidative stress and inflammatory pathways by downregulating nuclear factor kappa-light-chain-enhancer of activated B cells (NF-κB), tumor necrosis factor-alpha (TNF-α), and interleukin-6 (IL-6). Furthermore, they regulate myocardial energy metabolism by activating peroxisome proliferator-activated receptor alpha (PPARα) and promoting fatty acid oxidation [149]. Additionally, these compounds decrease levels of TGF-β1 and collagen I, effectively inhibiting myocardial fibrosis. This action results in anti-hypertrophic effects, enhanced cardiac function, and reduced myocardial damage.

In contrast to existing studies that predominantly focus on individual components or pathways, the primary advantage of AR lies in its comprehensive regulation through multiple components, targets, and pathways. While previous research often aims at specific pathological pathways using synthetic drugs or isolated plant components, the diverse constituents of AR—such as terpenoids, phenylpropanoids, and volatile oils—exert synergistic or cumulative effects on various pathological processes, including myocardial hypertrophy, fibrosis, oxidative damage, and energy metabolism disorders. For example, atractylenolide II and valerenic acid work in concert to alleviate myocardial remodeling and metabolic dysfunction by inhibiting the TGF-β1/Smad pathway and activating the PPARα pathway, respectively [150,151]. Simultaneously, camphene (121) and valencene (327) synergistically enhance the endogenous antioxidant system, thereby mitigating myocardial oxidative damage resulting from ischemia–reperfusion injury or hypertension [149,152]. This multi-tiered, networked mechanism of action aligns with the principles of combination therapy and systemic regulation emphasized in modern cardiovascular disease treatment. Moreover, it endows AR with a unique potential for the prevention and management of complex cardiac conditions, such as metabolic cardiomyopathy.

**Table 5:** Cardiac-protective effect of Atractylodis Rhizoma.

Bioactivity	Compounds/Extracts	Testing Subjects (Animal/Model)	Dose	Positive Control	Results/Mechanism	References
Cardio-protective effect	Camphene (121)	Wistar rats	30 µg/g	Not mentioned	LDH ↓, GSH/GSSG ↑; CS ↑; CAT ↑, Mn-SOD ↑, GR ↑; GPx4 ↓	[152]
Improvement of myocardial hypertrophy	Cryptochlorogenin acid (245)	In vivo: SD rats, In vitro: H9c2 cell	In vivo: 200 mg/kg; In vitro: 10, 25, 50 µM	Propranolol	Akt ↓, mTOR ↓, HIF-1α ↓; ANP ↓, BNP ↓	[151]
Improvement of cardiac function	Atractylenolide II (268)	Spontaneous hypertension rats and Wistar Kyoto rats	10, 30, 60 mg/kg/day	Not mentioned	LVEF ↑, LVMI ↓; Collagen I ↓, α-SMA ↓, Fibronectin ↓; TGF-β1 ↓; SOD ↑, GSH-Px ↑; H_2_O_2_ ↓, MDA ↓, NOX ↓	[150]
Anti-hypertrophic effect on the myocardium	Valerenic acid (288)	In vivo: ICR mice; In vitro: H9C2 cardiac myocytes	In vivo: 0.5 mg/kg, 2 mg/kg; In vitro: 1, 5, 10 µM	Fenofibrate	PPARα ↑; IL-6 ↓, IL-1β ↓, TNF-α ↓	[153]
Cardiac protection	Valencene (327)	Wistar rats	12 mg/kg/day	Not mentioned	cTn-I ↓, Myoglobin ↓; SOD ↑, CAT ↑, GPx ↑; hs-CRP ↓; Homocysteine ↓, NF-κB ↓, TNF-α ↓, IL-1β ↓, IL-6 ↓, IL-10 ↑	[149]

Note: ↑ represents an increase, and ↓ represents a decrease.

### 5.10. Joint Repair Function

*Atractylodes*, a genus of medicinal plants, is extensively employed in traditional medicine to alleviate dampness obstruction in the middle jiao and rheumatic arthralgia [12]. Notably, AR stands out among its species, as contemporary pharmacological research has underscored its substantial potential in the treatment of arthritis [154]. In contrast to chemically synthesized drugs, which generally target a singular pathway, the anti-arthritic effects of AR are derived from its naturally occurring synergistic pharmacodynamic complex, comprising multiple components. As demonstrated in Table 6, its primary active constituents—such as atractylenolide, eucalyptol, borneol acetate, carvone, geraniol, and ligustilide—exhibit multidimensional intervention in the intricate pathological processes of arthritis through an interconnected network mechanism, as evidenced in both animal and cellular models.

The principal benefit of this approach lies in its systematic regulation of the pathological network encompassing “inflammation-immunity-oxidation-degradation” in arthritis. This method effectively synergizes the inhibition of central inflammatory signaling pathways, such as NF-κB and MAPK, resulting in a comprehensive downregulation of key pro-inflammatory cytokines, including TNF-α, IL-6, and IL-1β [155]. Simultaneously, it modulates macrophage polarization, thereby exerting substantial control over the immune-inflammatory network. Additionally, it provides concurrent protection to joint tissue structures. For instance, geraniol (187) mitigates oxidative damage by activating antioxidant pathways, such as Keap1/Nrf2/HO-1 [156]. It also enhances the expression of type II collagen and aggregated proteoglycans while inhibiting MMP13 and ADAMTS-5.

**Table 6:** Joint repair function of Atractylodis Rhizoma.

Bioactivity	Compounds/Extracts	Testing Subjects (Animal/Model)	Dose	Positive Control	Results/Mechanism	References
Joint repair function	Atractylodin (141)	In vitro: BM-DCs cell; In vivo: DBA/1 mice	In vitro: 12.5, 25, 50, 100 µM; In vivo: 40 mg/kg	Quercetin	CD40 ↓, CD80 ↓, CD86 ↓; TNF-α ↓, IL-6 ↓, IL-1β ↓, IL-23 ↓, IL-12 ↓, IFN-γ ↓, NO ↓; p38 ↓, ERK ↓, NF-κB ↓	[155]
Anti-gouty arthritis effect	Eucalyptol (115)	BALB/c mice	30, 100, 300, 600 mg/kg	Indomethacin	NLRP3 ↓, IL-1β ↓, IL-6 ↓, TNF-α ↓, MPO ↓; ROS ↓, SOD ↑, GSH-Px ↑, MDA ↓; TRPV1 ↑, Nrf2 ↑, HO-1 ↑	[157]
Joint repair function	(-)-Bornyl acetate (126)	DBA/1J mice	20, 40 mg/kg	Methotrexate	CYP17A1 ↓, HSD17B3 ↓; TLR4 ↓, MAPK ↓, NF-κB ↓	[158]
Joint protection function	Carveol (146)	In vitro: RAW264.7 Macrophages, In vivo: C57BL/6J mice	In vitro: 10, 25, 50 µg/mL; In vivo: 12.5 mg/kg, 25 mg/kg	Not mentioned	M1-type macrophages ↓, IL-1β ↓, IL-6 ↓, TNF-α ↓, iNOS ↓; M2-type macrophages ↑, ARG-1 ↑, CD206 ↑, MGL-1 ↑, MGL-2 ↑; HO-1 ↑, ROS ↓; COX-2 ↓, MMP13 ↓, ADAMTS-5 ↓	[159]
Joint protection function	Geraniol (187)	In vitro: Mouse chondrocytes; In vivo: C57BL/6 mice	In vitro: 1 µM; In vivo: 1 mg/mL	Not mentioned	Col2a1 ↑, Aggrecan ↑; MMP13 ↓, ADAMTS-5 ↓; Keap1 ↓, Nrf2 ↑, HO-1 ↑; p-p65 ↓	[156]
Joint repair function	Senkyunolide A (294)	In vitro: Mouse chondrocytes; In vivo: C57BL/6 mice	In vitro: 20, 40, 80 µg/mL; In vivo: 20, 40 mg/kg	Not mentioned	MMP13 ↓, ADAMTS4/5 ↓; IGF-1 ↑, Aggrecan ↑, Col2a1 ↑; TNF-α ↓, IL-6 ↓, IL-18 ↓; NLRP3 ↓, Caspase-1 ↓	[160]

Note: ↑ represents an increase, and ↓ represents a decrease.

### 5.11. Protective Effect Against Lung Injury

The protective effects of AR against pulmonary injury are attributed to its multi-component synergistic actions, which establish a complex, multi-level, and multi-target regulatory network (Table 7). The active ingredient data compiled in this study elucidate these properties: chlorogenic acid (242) primarily exerts antioxidant effects and regulates macrophage polarization; dehydrocostus lactone (256) inhibits the key glycolytic enzyme PFKFB3, thereby modulating inflammatory metabolism. Acetylatractylodinol (270), isolated from its ethanol extract, concurrently inhibits inflammation mediated by the PI3K/AKT pathway and uniquely alters the host metabolic profile [161]. This indicates that it not only mitigates pulmonary edema and inflammatory responses but also potentially aids lung tissue in fulfilling energy and repair requirements under injury-induced stress through metabolic reprogramming. This comprehensive intervention, targeting the three core pathological pathways—inflammation, oxidative stress, and metabolic disorders—enables its mechanism of action to surpass traditional strategies primarily centered on immunosuppression, such as those exemplified by dexamethasone.

The metabolic regulatory function of acetylatractylodinol (270) is particularly noteworthy, offering substantial scientific value and potential for the development of AR in the treatment of lung injury. Contemporary therapeutic strategies for acute lung injury (ALI) are transitioning from exclusively anti-inflammatory approaches to those that prioritize the maintenance of tissue homeostasis, as metabolic dysregulation has been recognized as a pivotal factor in the progression of injury [165]. The bioactive constituents of AR such as dehydrocostus lactone (256) include compounds that both regulate inflammation and modulate metabolism, suggesting that the herb or its active fractions may influence more nuanced pathological processes, such as the repair of the alveolar epithelial barrier and the preservation of mitochondrial function. This discovery introduces a promising new paradigm for the development of lung protectants derived from natural products.

### 5.12. Other Effects

In addition to its previously discussed pharmacological effects, AR exhibits several other notable properties. Extracts of AR demonstrate significant anti-inflammatory effects in murine models of asthma and particulate matter-induced lung inflammation, potentially through the inhibition of the NF-κB pathway [166]. This effect may be attributed to components such as Senkyunolide A (294), which mitigates airway injury and remodeling in asthmatic mice and modulates the progression of asthma, consistent with the findings of [167]. Furthermore, the polysaccharides present in AR exhibit anti-aging properties and may serve as potential antioxidants [168]. These findings support the potential development of AR as a novel pharmaceutical and natural health product. However, factors such as poor water solubility and low bioavailability need to be overcome.

## 6. Toxicity

An analysis of ancient texts and contemporary research indicates that AR is characterized by minimal toxicity, a finding corroborated by extensive acute and chronic toxicity evaluations. In vivo studies using animal models such as mice, rats, and zebrafish embryos have systematically assessed its safety margin. Specifically, oral acute toxicity tests of *A. lancea* crude ethanolic extracts demonstrated a median lethal dose (LD50) greater than 2000 mg/kg, with no significant liver or kidney damage or pathological changes observed in other organs [169]. Furthermore, Even after processing, AR does not exhibit any significant toxicity [170]. This further demonstrates that AR is safe within certain dosage ranges. The safety profile is closely linked to its therapeutic mechanisms; studies confirm that AR mitigates inflammation and oxidative stress by inhibiting the NF-κB signaling pathway and activating the Nrf2 pathway without obvious toxic side effects at therapeutic doses [171].

Although the crude herb exhibits a wide safety window, investigations into major active constituents reveal dose-dependent toxicological effects that require careful consideration. AR samples with concentrations ranging from 0 to 25 mg/mL have demonstrated no toxic effects on A549 cells [172]. However, evaluations using in vitro and zebrafish embryo models for constituents such as atractylenolides (I, II, and III), atractylones, and β-eudesmol have identified specific risks. Notably, atractylenolides I (271) and II (268) have been shown to induce significant developmental abnormalities and hepatotoxicity in zebrafish embryos, evidenced by liver atrophy, decreased liver-specific fluorescence intensity, elevated liver enzyme levels, apoptosis, and the inhibition of drug-metabolizing enzymes [173]. At specific concentrations, β-eudesmol (71) and atractylones are linked to decreased embryo survival rates, developmental abnormalities, and alterations in the expression of genes related to oxidative stress, suggesting potential risks of embryotoxicity and oxidative damage [174]. Collectively, while AR possesses a substantial safety margin supporting its traditional use, certain active compounds may pose risks of developmental and hepatotoxicity at elevated doses or during embryonic development. Therefore, it is recommended that clinical applications carefully regulate dosages, avoid prolonged administration of high doses, and advance toxicological research and safety monitoring of these active constituents to ensure safe therapeutic application.

## 7. Limitations

Despite the comprehensive and systematic nature of this review, which integrates historical herbal textual research with modern phytochemical and pharmacological findings to provide a holistic understanding of AR, several inherent limitations must be acknowledged. These limitations primarily pertain to methodological constraints within the reviewed literature, the predominant reliance on preclinical evidence, and the corresponding lack of robust clinical validation, which collectively warrant a critical interpretation of the findings presented. First, from a methodological perspective, significant heterogeneity exists across the included studies regarding extraction protocols, compound identification, and experimental models. Many pharmacological investigations have utilized crude extracts or essential oils without standardized quantification of their major bioactive constituents, complicating the attribution of observed effects to specific compounds and hindering cross-study comparability. Furthermore, although computational approaches such as molecular docking have been extensively employed to predict mechanisms of action, these in silico findings frequently lack experimental validation through target engagement assays or genetic knockdown models, leaving proposed molecular interactions largely speculative.

Second, the current evidence base is overwhelmingly dominated by preclinical studies, with in vitro experiments on cell lines and in vivo evaluations in rodent models constituting the vast majority of pharmacological data. While these studies provide valuable mechanistic insights and establish biological plausibility, they possess inherent limitations in recapitulating human disease pathophysiology, particularly regarding drug metabolism, immune system interactions, and chronic disease progression. The translational relevance of these findings remains uncertain without confirmation in higher animal models or human systems. Third, and perhaps most critically, clinical evidence validating the therapeutic efficacy and safety of AR in human subjects remains exceptionally limited. The few available clinical studies are characterized by small sample sizes, lack of randomization or blinding, and absence of standardized AR preparations with verified phytochemical profiles. Consequently, definitive conclusions regarding optimal dosing, long-term safety, and comparative effectiveness against standard therapies cannot be drawn. This scarcity of rigorous clinical trials represents the most significant barrier to evidence-based clinical application. Additionally, the two pharmacopoeial species, *A. lancea* and *A. chinensis*, are often conflated in both experimental and clinical literature, yet emerging evidence demonstrates substantial chemotaxonomic differences that may translate into differential pharmacological activities. This conflation introduces confounding variables that undermine the specificity and reproducibility of research findings. It must not be overlooked that one reason for AR’s favourable biological activity lies in its nature as a complex mixture of multiple compounds, presenting an unavoidable challenge when considering it for development and therapeutic applications.

By explicitly acknowledging these limitations, this review aims to enhance critical depth and transparency, emphasizing that while the foundational science of AR is robust and promising, its clinical translation remains nascent. Future research must prioritize methodological rigor, standardized phytochemical characterization, and well-designed clinical trials to transform the extensive preclinical promise of AR into evidence-based therapeutic applications.

## 8. Conclusions

Despite significant advancements in understanding the phytochemistry and pharmacology of AR, several critical challenges and knowledge gaps persist, necessitating a paradigm shift in future research endeavors. The current body of evidence is constrained by three fundamental contradictions: the discordance between complex phytochemical profiles and poorly defined molecular mechanisms, the disparity between potent in vitro bioactivities and unfavorable in vivo pharmacokinetics characterized by low bioavailability, and the clinical interchangeability of two chemically distinct pharmacopoeial species, specifically *A. lancea* and *A. chinensis*.

To bridge these gaps and facilitate the evidence-based development of AR from a traditional remedy to a modern therapeutic agent, future investigations should prioritize five interconnected research directions. First, the in vivo fate and direct protein targets of its bioactive constituents demand rigorous elucidation through integrated pharmacokinetic profiling and chemical proteomics approaches such as activity-based protein profiling, thereby establishing a definitive molecular basis for its traditional efficacy in invigorating the spleen and resolving dampness. Second, the synergistic mechanisms underlying its multi-component nature should be systematically deconvoluted by employing network pharmacology integrated with multi-omics readouts including transcriptomics, proteomics, and metabolomics to map the compound–target–pathway–phenotype network, with particular emphasis on the combinatorial effects and potential synergism among major constituents like atractylodin, β-eudesmol, and the atractylenolides. Third, overcoming the intrinsic challenge of poor bioavailability, particularly for hydrophobic sesquiterpenes, is paramount and necessitates the rational design of advanced drug delivery systems including nanocarriers such as liposomes and polymeric nanoparticles, alongside investigations into traditional combination strategies that may enhance gastrointestinal absorption. Fourth, given the pronounced chemotaxonomic variation between *A. lancea* and *A. chinensis*, rigorous comparative studies are urgently required to correlate their distinct chemical signatures with differential pharmacological potencies in core therapeutic areas such as gastroprotection and anti-inflammation, thereby providing a scientific rationale for species-specific clinical application and quality control. Finally, a comprehensive safety evaluation framework extending beyond conventional acute toxicity models is essential; this should leverage advanced platforms including zebrafish embryos and organoid technologies to systematically assess the chronic, developmental, and organ-specific toxicity profiles of major constituents, thereby establishing definitive safety margins for clinical translation. By addressing these critical areas through the application of contemporary life sciences methodologies, future research can transform the therapeutic potential of AR into clinically validated, mechanism-based interventions for gastrointestinal, metabolic, and inflammatory disorders.

## Figures and Tables

**Table 1:** Name, Characteristic traits and Origin of Atractylodis Rhizoma in ancient books.

Dynasty	Reference	Name	Characteristics	Producing Areas	Origin
the Warring States period	Er Ya	Zhu, Shan-Ji, and Yang-Bao	Elliptical leaves, spiny-toothed leaf margins, capitate inflorescences, and purplish-red or white florets	Not mentioned	*A. lancea*/*A. chinensis*
Han	Shennong’s Herbal Classic	Shan-Ji	Thistle-like	Not mentioned	*A. lancea*/*A. chinensis*
Han	Wu Pu Ben Cao	Shan-Jie, Tian-Su	Its pungent flavor resembles ginger and mustard	Not mentioned	*A. lancea*/*A. chinensis*
Han	Ming Yi Bie Lu	Shan-Lian	Not mentioned	Jiangsu Province	*A. lancea*
Northern and Southern Dynasties	Ben Cao Jing Ji Zhu	Shan-Jing, Cang-Zhu	The leaves are slender and branchless, the roots are small, bitter, and rich in sap.	Jiangsu Province	*A. lancea*
Song	Ben Cao Tu Jing	Cang-Zhu	Elliptical leaves, spiny-toothed leaf margins, capitate inflorescences, and purplish-red or white florets	Shaanxi, Henan, Jiangsu, and other Provinces	*A. lancea*/*A. chinensis*
Ming	Ben Cao Pin Hui Jing Yao	Cang-Zhu	Leaves are narrow and hairless, arranged in pairs opposite each other… The root resembles ginger but lacks branches, with black skin and yellow flesh, rich in oily sap within. Its taste is bitter-sweet and pungent, and it readily develops a white frost.	Jiangsu and Henan Provinces	*A. lancea*/*A. chinensis*
Ming	Ben Cao Yuan Shi	Cang-Zhu	Maoshan Cang-Zhu is superior, with black bark and white flesh dotted with yellow; firm and small with sweet and pungent aroma. Those from other mountains have large, yellowish roots with intense pungency. Another variety features white bark and flesh, firm texture, sweet and pungent flavor, but is of lesser quality.	Jiangsu Province	*A. lancea*
Qing	Ben Cao Chong Yuan	Cang-Zhu	The leaves near the root branch into three or five forks, while the upper leaves are narrow and elongated, green and glossy. The stem is purple. The root resembles old ginger, with a dark brown skin and yellow flesh; when mature, it develops cinnabar-like spots. It tastes sweet at first, then bitter, with a distinctly pungent flavor. Its nature is dry and fiery	Not mentioned	*A. chinensis*
Qing	Ben Cao Cong Xin	Cang-Zhu	The best Cang-Zhu from Maoshan is sweet in taste, slender in form, and covered with abundant hair; those from the mountains of Wu Prefecture are next in quality	Jiangsu, Zhejiang, Anhui and Hubei Provinces	*A. lancea*

**Table 3:** Anti-diabetic effect of Atractylodis Rhizoma.

Bioactivity	Compounds/Extracts	Testing Subjects (Animal/Model)	Dose	Positive Control	Results/Mechanism	References
Anti-diabetic effect	γ-Cadinene (42)	In silico (computational model)	Not mentioned	Metformin	The binding affinity for the insulin receptor (INSR) is −7.3 kcal/mol	[112]
α-Glucosidase inhibitory activity	Eremophilene (47, as a component of *T. papyrifer* essential oil)	In vitro enzyme assay	Not specified (present at 6.15% in EO tested at 500 µg/mL)	Acarbose	The essential oil containing eremophilene showed 17.86% α-glucosidase inhibition at 500 µg/mL.	[115]
Anti-diabetic effect	Valeranone (60, from *Amberboa ramosa* oil)	In vitro human DPP-IV enzyme assay	IC_50_ = 14.92 µg/mL	Alogliptin	Not mentioned	[113]
Inhibitory effect on retinal pigment epithelial barrier dysfunction	Eucalyptol (115)	In vitro: Human retinal pigment epithelial cells; In vivo: db/db mice	In vitro: 1–20 µM; In vivo: 10 mg/kg	Not mentioned	ZO-1 ↓, occludin-1 ↓; MMP-2 ↓, MMP-9 ↓; bax ↓, caspase-3 ↓, Bcl-2 ↑; ROS ↓; Aβ formation ↓, RAGE ↓	[114]
Hypoglycemic effect	β-Pinene (119)	Wistar rats	25, 50, 100, 200 mg/kg	Glibenclamide	ATP-dependent K^+^ channel ↓	[116]
α-Glucosidase inhibition	(-)-Bornyl acetate (126) from *Amomum villosum* essential oil	Enzyme Inhibition Assay	0.5–2.5 mg/mL	Acarbose	Binding energy to PPARα: −6.9 kcal/mol, potentially regulating glucose metabolism through the PPAR signaling pathway	[117]
Anti-diabetic effect	Geraniol (187)	Wistar rats	100 mg/kg, 200 mg/kg	Insulin	TC ↓, TG ↓, LDL-C ↓, HDL-C ↑; SOD ↑, CAT ↑, GPx ↑, GST ↑, GR ↑; Bcl-2 ↑, Caspase-3 ↓, Bax ↓; ALT ↑, AST ↑, ALP ↑, Carbamide ↑, Creatinine ↑, Uric acid ↑	[118]
Lower blood sugar and protect pancreatic effects	Cryptochlorogenic acid (245)	In vivo: SD rats; In vitro: INS-1 cell	In vivo: 15, 30, 60 mg/kg; In vitro: 10, 25, 50 µM	Rosiglitazone	XC ↑/GPX4 ↑/Nrf2 ↑, NCOA4 ↓, MDA ↓, GSH ↑, GPX4 ↑	[119]
α-Glucosidase inhibitory activity	Vitexin (252) from *Ficus deltoidea* leaves	SD rats	50 mg/kg	Not mentioned	Longer-lasting hypoglycemic effects and higher accumulation in the gut	[120]

Note: ↑ represents an increase, and ↓ represents a decrease.

**Table 7:** Protective effect against lung injury of Atractylodis Rhizoma.

Bioactivity	Compounds/Extracts	Testing Subjects (Animal/Model)	Dose	Positive Control	Results/Mechanism	References
Anti-Acute Lung Injury (ALI)	Chlorogenic acid (242)	In vivo: C57BL/6 mice; In vitro: RAW264.7 Macrophages	In vivo: 61 µg/mL; In vitro: 488 ng/mL	Dexamethasone	MDA ↓, SOD ↑, GSH ↑, M1 macrophage ↓, IL-1β ↓, IL-6 ↓, TNF-α ↓, IL-10 ↑, iNOS ↓, MPO ↓, ROS ↓, iNOS ↑, IL-10 ↑, NO ↓	[162]
Anti-lung injury	Hymecromone (244)	C57BL/6 mice	60 mg/kg	Dexamethasone	HAS2 ↓, HAS3 ↓, CRP ↓, D-dimer ↓	[163]
Lung protection	dehydrocostus lactone (256)	In vivo: C57BL/6 mice; In vitro: RAW264.7	In vivo: 10, 20 mg/kg; In vitro: 0.625–20 µM;	Dexamethasone	PFKFB3 ↓, ECAR ↓, lactate ↓, TNF-α ↓, IL-6 ↓, NO ↓, p-IκBα ↓, p-p65 ↓, lung edema ↓	[164]
Anti-pulmonary oedema	Acetylatractylodinol (270)	SD rats	314.7, 2517.4 mg/kg	Dexamethasone	TNF-α ↓, IL-6 ↓, IL-1β ↓, MCP-1 ↓; p-PI3K ↓, p-AKT ↓; Serine ↑, L-Alanine ↑, L-Proline ↑; D-Galactose ↓, D-Glucose ↓, D-Mannose ↓	[161]

Note: ↑ represents an increase, and ↓ represents a decrease.

## Data Availability

Not applicable.

## Supplementary Materials

The following supporting information can be downloaded at: https://www.mdpi.com/article/10.3390/molecules31061015/s1, Table S1. Traditional preparations of Atractylodis Rhizoma; Table S2. Modern preparations of Atractylodis Rhizoma; Table S3. Antimicrobial effect of Atractylodis Rhizoma; Table S4. Anti-inflammatory effect of Atractylodis Rhizoma; Table S5. Antioxidant effect of Atractylodis Rhizoma; Table S6. Hepatoprotective effect of Atractylodis Rhizoma; Table S7. Anti-cancer effect of Atractylodis Rhizoma; Table S8. Intestinal regulatory function of Atractylodis Rhizoma.

## Abbreviations

The following abbreviations are used in this manuscript: AR, Atractylodis Rhizoma; MIC, Minimum Inhibitory Concentration; MBC, Minimum Bactericidal Concentration; AA, arachidonic acid; TPA, tetradecanoylphorbol-13-acetate-; DPPH, 1,1-diphenyl-2-picryl-hydrazyl radical; ABTS, 2, 2′-azino-bis(3-ethylbenzothiazoline-6-sulfonic acid); FRAP, ferric reducing antioxidant power; TRP, total reducing power; CUPRAC, cupric reducing antioxidant capacity; NO, Nitric Oxide; 8-OHdG, 8-Hydroxy-2′-deoxyguanosine; Aβ, Amyloid-beta; ACC, Acetyl-CoA Carboxylase; AChE, Acetylcholinesterase; ACLY, ATP-Citrate Lyase; ADAM10, A Disintegrin And Metalloproteinase 10; ADAMTS-4, A Disintegrin And Metalloproteinase with Thrombospondin Motifs 4; ADAMTS-5, A Disintegrin And Metalloproteinase with Thrombospondin Motifs 5; Aggrecan, Aggrecan; Akt, Protein Kinase B; ALP, Alkaline Phosphatase; ALT, Alanine Aminotransferase; AMPK, AMP-activated Protein Kinase; ANP, Atrial Natriuretic Peptide; AQP4, Aquaporin-4; ARE, Antioxidant Response Element; ARG-1, Arginase-1; AST, Aspartate Aminotransferase; ATP6V0D2, V-type Proton ATPase Subunit D2; BACE1, Beta-site APP Cleaving Enzyme 1; Bax, BCL2-Associated X Protein; BCL2, B-cell Lymphoma 2; BDNF, Brain-Derived Neurotrophic Factor; Bcl-2, B-cell Lymphoma 2; Bcl-xl, B-cell Lymphoma-extra Large; BNP, Brain Natriuretic Peptide; Carbamide, Urea; CAT, Catalase; CCL2, C-C Motif Chemokine Ligand 2; CD206, Cluster of Differentiation 206; CD40, Cluster of Differentiation 40; CD80, Cluster of Differentiation 80; CD86, Cluster of Differentiation 86; CDK2, Cyclin-dependent Kinase 2; CHO, Total Cholesterol; CLCC1, Chloride Channel CLIC-like 1; Claudin-1, Claudin-1; COX-2, Cyclooxygenase-2; CPT-1, Carnitine Palmitoyltransferase I; Creatinine, Creatinine; CRP, C-reactive Protein; CRT, Calreticulin; CS, Citrate Synthase; cTn-I, Cardiac Troponin I; CXCL16, C-X-C Motif Chemokine Ligand 16; CYP17A1, Cytochrome P450 Family 17 Subfamily A Member 1; CYP2b10, Cytochrome P450 Family 2 Subfamily B Member 10; CYP3a11, Cytochrome P450 Family 3 Subfamily A Member 11; DAI Score, Disease Activity Index Score; Dgat2, Diacylglycerol O-Acyltransferase 2; DR3, Death Receptor 3; ECAR, Extracellular Acidification Rate; EGFR, Epidermal Growth Factor Receptor; EMT, Epithelial–Mesenchymal Transition; ER, Endoplasmic Reticulum; ERK, Extracellular Signal-regulated Kinase; Fasn, Fatty Acid Synthase; FAK, Focal Adhesion Kinase; FGF1, Fibroblast Growth Factor 1; FGFR, Fibroblast Growth Factor Receptor; G6PD, Glucose-6-Phosphate Dehydrogenase; GABA, Gamma-Aminobutyric Acid; GAPDH, Glyceraldehyde-3-Phosphate Dehydrogenase; GDH, Glutamate Dehydrogenase; GLS, Glutaminase; GPX4, Glutathione Peroxidase 4; GPx, Glutathione Peroxidase; GR, Glutathione Reductase; GSH, Glutathione; GSDMD, Gasdermin D; GSSG, Oxidized Glutathione; GST, Glutathione S-Transferase; H_2_O_2_, Hydrogen Peroxide; HAS2, Hyaluronan Synthase 2; HAS3, Hyaluronan Synthase 3; HDL-C, High-Density Lipoprotein Cholesterol; HER2, Human Epidermal Growth Factor Receptor 2; Hes1, Hes Family BHLH Transcription Factor 1; HIF-1α, Hypoxia-Inducible Factor 1-alpha; HMGB1, High Mobility Group Box 1; HO-1, Heme Oxygenase-1; hs-CRP, High-sensitivity C-reactive Protein; HSD17B3, Hydroxysteroid 17-Beta Dehydrogenase 3; IAA, Indole-3-acetic Acid; ICAM-1, Intercellular Adhesion Molecule-1; IFN-γ, Interferon-gamma; IGF-1, Insulin-like Growth Factor 1; IKK, Inhibitor of Nuclear Factor Kappa-B Kinase; IL-1β, Interleukin-1 beta; IL-6, Interleukin-6; IL-8, Interleukin-8; IL-10, Interleukin-10; IL-12, Interleukin-12; IL-17A, Interleukin-17A; IL-18, Interleukin-18; IL-23, Interleukin-23; iNOS, Inducible Nitric Oxide Synthase; IRF1, Interferon Regulatory Factor 1; JAK2, Janus Kinase 2; JNK, c-Jun N-terminal Kinase; Keap1, Kelch-like ECH-associated Protein 1; KYNA, Kynurenic Acid; LAMP1, Lysosomal-associated Membrane Protein 1; LAMP2, Lysosomal-associated Membrane Protein 2; LDH, Lactate Dehydrogenase; LDHA, Lactate Dehydrogenase A; LDL-C, Low-Density Lipoprotein Cholesterol; LPO, Lipid Peroxidation; LPS, Lipopolysaccharide; LVEF, Left Ventricular Ejection Fraction; LVMI, Left Ventricular Mass Index; MCP-1, Monocyte Chemoattractant Protein-1; MDA, Malondialdehyde; Mdr1a, Multidrug Resistance Protein 1a; MGL-1, Macrophage Galactose-type Lectin 1; MGL-2, Macrophage Galactose-type Lectin 2; MIP-1α, Macrophage Inflammatory Protein-1 alpha; MLCK, Myosin Light Chain Kinase; MMP, Matrix Metalloproteinase; MMP-2, Matrix Metalloproteinase 2; MMP-9, Matrix Metalloproteinase 9; MMP13, Matrix Metalloproteinase 13; MPO, Myeloperoxidase; mTOR, Mammalian Target of Rapamycin; MUC2, Mucin 2; MyD88, Myeloid Differentiation Primary Response 88; NE, Norepinephrine; NF-κB, Nuclear Factor Kappa-light-chain-enhancer of Activated B cells; Ngn2, Neurogenin 2; NLRC4, NLR Family CARD Domain Containing 4; NLRP1, NLR Family Pyrin Domain Containing 1; NLRP3, NLR Family Pyrin Domain Containing 3; NCOA4, Nuclear Receptor Coactivator 4; Nrf2, Nuclear Factor Erythroid 2-Related Factor 2; OCR, Oxygen Consumption Rate; Occludin, Occludin; p21, Cyclin-dependent Kinase Inhibitor 1A; p38, p38 Mitogen-Activated Protein Kinase; p53, Tumor Protein p53; p65, Nuclear Factor NF-kappa-B p65 subunit; p70S6K, Ribosomal Protein S6 Kinase; PARP, Poly ADP-ribose Polymerase; PBLD, Phenazine Biosynthesis-Like Protein Domain-containing Protein; PD-L1, Programmed Death-Ligand 1; PECAM-1, Platelet Endothelial Cell Adhesion Molecule-1; PFKFB3, 6-Phosphofructo-2-Kinase/Fructose-2,6-Biphosphatase 3; PI3K, Phosphatidylinositol 3-Kinase; PKM2, Pyruvate Kinase M2; p-MLC, Phosphorylated Myosin Light Chain; PMN, Polymorphonuclear Neutrophil; PPARα, Peroxisome Proliferator-Activated Receptor Alpha; PGE2, Prostaglandin E2; RAGE, Receptor for Advanced Glycation Endproducts; RANTES, Regulated on Activation, Normal T Expressed and Secreted; RhoA, Ras Homolog Family Member A; ROCK, Rho-associated Coiled-coil Containing Protein Kinase; ROS, Reactive Oxygen Species; RXRa, Retinoid X Receptor Alpha; Scd2, Stearoyl-CoA Desaturase 2; Serine, Serine; SIRT1, Sirtuin 1; SNAP23, Synaptosome Associated Protein 23; SOD, Superoxide Dismutase; Srebf1, Sterol Regulatory Element Binding Transcription Factor 1; SREBP-1, Sterol Regulatory Element-Binding Protein 1; Src, Proto-oncogene Tyrosine-Protein Kinase Src; STAT1, Signal Transducer and Activator of Transcription 1; STAT3, Signal Transducer and Activator of Transcription 3; STAT6, Signal Transducer and Activator of Transcription 6; TAC, Total Antioxidant Capacity; TC, Total Cholesterol; TEER, Transepithelial Electrical Resistance; TG, Triglycerides; TGF-β1, Transforming Growth Factor Beta 1; TLR4, Toll-like Receptor 4; TNF-α, Tumor Necrosis Factor-alpha; TRAF6, TNF Receptor Associated Factor 6; TRPM8, Transient Receptor Potential Melastatin 8; TRPV1, Transient Receptor Potential Vanilloid 1; TWIST1, Twist Family BHLH Transcription Factor 1; Uric acid, Uric Acid; VDR, Vitamin D Receptor; VEGFA, Vascular Endothelial Growth Factor A; Vimentin, Vimentin; XCL1, X-C Motif Chemokine Ligand 1; xCT, Cystine/Glutamate Antiporter; XIAP, X-linked Inhibitor of Apoptosis Protein; ZEB1, Zinc Finger E-box Binding Homeobox 1; ZO-1, Zonula Occludens-1; α-SMA, Alpha-Smooth Muscle Actin.

## References

[1] Jun X., Fu P., Lei Y., Cheng P. (2018). Pharmacological effects of medicinal components of *Atractylodes lancea* (Thunb.) DC. Chin. Med..

[2] Zhou L. (2024). Textual Research of Historical and Current Application of Atractylodis Macrocephalae Rhizoma in Classic Famous Prescriptions. Chin. J. Mod. Appl. Pharm..

[3] Ke C., Qu L., Liu Y., Xia Y., Wang C., Xu K. (2025). Rhizoma Atractylodis: A review on processing, chemical composition, pharmacological effects, and product development. Acupunct. Herb. Med..

[4] Yang L., Yu H., Hou A., Man W., Wang S., Zhang J., Wang X., Zheng S., Jiang H., Kuang H. (2021). A Review of the Ethnopharmacology, Phytochemistry, Pharmacology, Application, Quality Control, Processing, Toxicology, and Pharmacokinetics of the Dried Rhizome of *Atractylodes macrocephala*. Front. Pharmacol..

[5] Wu Y.J., Tang L. (2023). Efficacy Analysis of Wandai Decoction Combined with Traditional Chinese Medicine Fumigation and Washing in Patients with Chronic Vaginitis After Sintilimab Treatment for Small Cell Lung Cancer. Altern. Ther. Health Med..

[6] Yang Y.L., Zhao C.Z., Zhao C.C., Wen Z.Y., Ma Y.Y., Zhao X.N., Wang L., Huang J.L., Zhou P. (2024). Ling-Gui-Zhu-Gan decoction protects against doxorubicin-induced myocardial injury by downregulating ferroptosis. J. Pharm. Pharmacol..

[7] Li N., Zhu T.Y., Dong Y.X., Zhao C.Y., Chen J.H., Tian Y.X., Liu Y.L., Hong X., Xiong H. (2025). UPLC-Q-TOF-MS-Based Serum Metabolomics Explores the Mechanism of Pingwei Powder in Treating the Damp Retention in the Middle-Jiao Syndrome. Biomed. Chromatogr..

[8] Zhang Y.N., Zhang S., Fan Y.M., Huang S.J., Wang S.M., Hao Z.H., Shen J.Z. (2025). Exploring the Underlying Mechanism of Weiling Decoction Alleviates Cold-Dampness Diarrhea Based on Network Pharmacology, Transcriptomics, Molecular Docking and Experimental Validation. Pharmaceuticals.

[9] Jiang Z., Jin K., Zhong L., Zheng Y., Shao Q., Zhang A. (2023). Near-infrared spectroscopy combined with machine learning for rapid identification of Atractylodis rhizoma decoction pieces. Ind. Crops Prod..

[10] Wang M., Chen P., Yin M., Xu X., Chen Y., Feng X., Guan F., Liao P., Wang Q. (2023). Phytochemical and chemotaxonomic study on *Atractylodes lancea*. Biochem. Syst. Ecol..

[11] Ma Z., Zhao X., Xie Z., Lv M., Gao J., Sun L., Li J., Ren X. (2025). Fourier transform infrared spectroscopy, high-performance liquid chromatography with diode array detection, and gas chromatography-mass spectrometry fingerprints combined with chemometrics for comprehensive evaluation and identification of raw and bran-fried Atractylodis Rhizoma. J. Pharm. Biomed. Anal..

[12] Zhang W.J., Zhao Z.Y., Chang L.K., Cao Y., Wang S., Kang C.Z., Wang H.Y., Zhou L., Huang L.Q., Guo L.P. (2021). Atractylodis Rhizoma: A review of its traditional uses, phytochemistry, pharmacology, toxicology and quality control. J. Ethnopharmacol..

[13] Kou B., Meng L., Zhao M., Wang H., Lu C., Yan M., Li G. (2025). Unveiling the power of *Pueraria lobata*: A comprehensive exploration of its medicinal and edible potentials. Front. Pharmacol..

[14] Feng J.X., Wu Y.Z., Li S.Y., Chang A., Yu Q.X., Zhang H. (2023). Correlation between active component content and color of *Atractylodes lancea* and *A. chinensis* based on color difference principle. China J. Chin. Mater. Med..

[15] Xiao W. (2007). Kinetics and Mechanism Studies on Oxidizing Reaction of Atractylon in Essential Oil from *Aatractylodes mmacrocephala* Koidz. Chin. J. Appl. Chem..

[16] Yan M., Wei T., Zhao D., Wei X., Chen F., Wang C., Xiao C. (2023). Comprehensive Quality Evaluation of Bran-processed Cangzhu (Atractylodis Rhizoma) Based on Chemometrics and Entropy Weight TOPSIS Analysis Combined with Multi-Component Quantification. Guid. J. Tradit. Chin. Med. Pharmacol..

[17] Manayi A., Kurepaz-mahmoodabadi M., Gohari A.R., Ajani Y., Saeidnia S. (2014). Presence of phthalate derivatives in the essential oils of a medicinal plant *Achillea tenuifolia*. DARU J. Pharm. Sci..

[18] Gan Y.F., Yang T., Gu W., Guo L.P., Qiu R.L., Wang S., Zhang Y., Tang M., Yang Z.C. (2024). Using HS-GC-MS and flash GC e-nose in combination with chemometric analysis and machine learning algorithms to identify the varieties, geographical origins and production modes of *Atractylodes lancea*. Ind. Crops Prod..

[19] Gan Y., Ju R., Peng Y., Xiao S., Qiu R., Wang S., Zhang Y., Guo L., Gu W. (2026). A multi-platform analytical strategy for *Atractylodes lancea* authentication: Fusion of stable isotope, elemental, chromatographic, and spectroscopic profiles. Talanta.

[20] Lu J., Chen W.T., Zhou B.W., Chen Y., Wang X.H., An R., Yang M. (2020). Distinguishing the Rhizomes of *Atractylodes japonica*, *Atractylodes chinensis*, and *Atractylodes lancea* by Comprehensive Two-Dimensional Gas Chromatography Coupled with Mass Spectrometry Combined with Multivariate Data Analysis. Pharmacogn. Mag..

[21] Ji L., Ao P., Pan J.G., Yang J.Y., Yang J., Hu S.L. (2001). GC-MS analysis of essential oils from rhizomes of *Atractylodes lancea* (Thunb.) DC. and *A. chinensis* (DC.) Koidz. China J. Chin. Mater. Med..

[22] Xu C., Meng L.B., Lu M.Q., Huang X.Y., Wang X., Gong F.P., Gong Q.F., Yu H. (2024). Influence of different processing methods on volatile components of Atractylodis Rhizoma based on HS-GC-MS technology. China J. Chin. Mater. Med..

[23] Zhuang L.X., Liu Y., Wang S.Y., Sun Y., Pan J., Guan W., Hao Z.C., Kuang H.X., Yang B.Y. (2022). Cytotoxic Sesquiterpenoids from *Atractylodes chinensis* (DC.) Koidz. Chem. Biodivers..

[24] Song B., Wang W., Liu R., Cai J., Jiang Y., Tang X., Wu H., Ao H., Chen L. (2023). Geographic Differentiation of Essential Oil from Rhizome of Cultivated *Atractylodes lancea* by Using GC-MS and Chemical Pattern Recognition Analysis. Molecules.

[25] Guo F.-Q., Huang L.-F., Zhou S.-Y., Zhang T.-M., Liang Y.-Z. (2006). Comparison of the volatile compounds of *Atractylodes* medicinal plants by headspace solid-phase microextraction-gas chromatography–mass spectrometry. Anal. Chim. Acta.

[26] Jia C., Mao D., Zhang W., Sun X. (2004). Studies on chemical constituents in essential oil from wild *Atractylodes lancea* in dabie mountains. J. Chin. Med. Mater..

[27] Ahmed S., Zhan C.S., Yang Y.Y., Wang X.K., Yang T.W., Zhao Z.Y., Zhang Q.Y., Li X.H., Hu X.B. (2016). The Transcript Profile of a Traditional Chinese Medicine, *Atractylodes lancea*, Revealing Its Sesquiterpenoid Biosynthesis of the Major Active Components. PLoS ONE.

[28] Zhang Z.A., Xue X.Y., Wu J.Y., Zhao X., Zhang H., Sun K., Dai C.C., Chen F. (2025). CYP71P2 regulated by MYB44 modulates oxygenated sesquiterpenoids biosynthesis in *Atractylodes lancea*. Plant Physiol. Biochem..

[29] Xu X.J., Xiong X.H., He Z.Q., Lu Q., Wang L. (2026). Optimization of ultrasound pretreatment combined with solvent-free microwave for extracting volatile oil from *Atractylodes lancea* and its chemical composition and antimicrobial activity. Talanta.

[30] Kamauchi H., Kinoshita K., Takatori K., Sugita T., Takahashi K., Koyama K. (2015). New sesquiterpenoids isolated from *Atractylodes lancea* fermented by marine fungus. Tetrahedron.

[31] Chu S.S., Jiang G.H., Liu Z.L. (2011). Insecticidal compounds from the essential oil of Chinese medicinal herb *Atractylodes chinensis*. Pest Manag. Sci..

[32] Wang M., Deng J., Duan G.H., Chen L., Huang X., Wang W.J., Gong L., Zhang Y., Yu K., Guo L.P. (2023). Insights into the impacts of autotoxic allelochemicals from rhizosphere of *Atractylodes lancea* on soil microenvironments. Front. Plant Sci..

[33] Kim H.K., Yun Y.K., Ahn Y.J. (2007). Toxicity of atractylon and atractylenolide III identified in *Atractylodes ovata* rhizome to *Dermatophagoides farinae* and *Dermatophagoides pteronyssinus*. J. Agric. Food Chem..

[34] Bagal S.K., Adlington R.M., Marquez R., Cowley A.R., Baldwin J.E. (2003). Studies towards the biomimetic synthesis of bisesquiterpene lactones. Tetrahedron Lett..

[35] Zhao J.H., Sun C.Z., Shi F.Y., Ma S.S., Zheng J.S., Du X., Zhang L.P. (2021). Comparative transcriptome analysis reveals sesquiterpenoid biosynthesis among 1-, 2-and 3-year old *Atractylodes chinensis*. BMC Plant Biol..

[36] Lei H., Yue J., Yin X.Y., Fan W., Tan S.H., Qin L., Zhao Y.N., Bai J.H. (2023). HS-SPME coupled with GC-MS for elucidating differences between the volatile components in wild and cultivated *Atractylodes chinensis*. Phytochem. Anal. PCA.

[37] Li Y. (2023). Impact of Using Natural Herbal Materials for Acupoint Application Combined with an Evidence-Based Supervision Method on Improving Renal Function and Lifestyle Treatment in Hemodialysis Patients. J. Biobased Mater. Bioenergy.

[38] Chen L.N., Li Y.H., Huang X., Deng J., Qu C.L., Zhang X.Q., Huang B.S., Zhang Y., Gong L., Yu K. (2021). Cloning and functional characterization of a terpene synthase gene AlTPS1 from *Atractylodes lancea*. Biol. Plant..

[39] Zhou J.Y., Li X., Zheng J.Y., Dai C.C. (2016). Volatiles released by endophytic *Pseudomonas fluorescens* promoting the growth and volatile oil accumulation in *Atractylodes lancea*. Plant Physiol. Biochem..

[40] Zhi Z., Xuening Y., Shimin P., Tao Z., Miaoting S. (2012). Comparison of the Volatile Oil Components from *Atractylodes chinensis* (DC.) Koidz. and *Atractylodes lancea* (Thunb.) DC. Chin. J. Appl. Chem..

[41] Fang J., Weng L., Wang M., Xiao C., Yang X., Sun J., Feng Y. (2023). Analysis of chemical compositions in *Atractylodes chinensis* with rice water before and after processing and its effects on intestinal fungal flora of spleen deficiency diarrhea rats. Chin. Tradit. Herb. Drugs.

[42] Wang C., Xiang Q., Zhao W., Gong Q., Yu H. (2022). Analysis of Chemical Compositions in *Atractylodes lancea* Rhizoma Before and After Processing with Rice-washed Water by UPLC-Q-TOF-MS. Chin. J. Exp. Tradit. Med. Formulae.

[43] Lin X., Chen Z., Zou J., Shi Y., Zhang X., Guo D., Zhai B., Luan F. (2025). Light stability examination and GC-MS analysis of volatile oils of *Acori Tatarinowii* Rhizoma and Atractylodis Rhizoma under treatment of β-cyclodextrin inclusion and Pickering emulsion technology. Chin. Tradit. Herb. Drugs.

[44] Ding S., Qiu M., Cao Y., Pan L. (2025). Identification of Different Volatile Components in Fresh and Processed *Atractylodis Macrocephala* Rhizoma Based on GC-IMS. J. Instrum. Anal..

[45] Liu X., Yan X., Wei Y. (1998). Analysis of the Essential Oil Compositionsin Rhizome of *Atractylodes lancea* (Thunb) DC. J. Instrum. Anal..

[46] Wang F., Ouyang Z., Guo L.P., Zhao M., Peng H.S., Liao J.L., Liang Z.P. (2014). Comprehensive chemical pattern recognition of atractylodis rhizoma. China J. Chin. Mater. Med..

[47] Li X., Bai Y., Qu Y., Cai Q. (2024). Study on the difference of chemical component in three processed products of *Atractylodes chinensis* (DC.) Koidz. China J. Tradit. Chin. Med. Pharm..

[48] Xin Y., Yu K.C., Yu Y., Wang H.J. (2022). Filtration and qualification for target biomarkers of traditional Chinese medicine formula “fuzi lizhong decoction” acting on stomach ulcer by UPLC/Q-TOF MS. Pak. J. Pharm. Sci..

[49] Lu M.X., Yin J.Y., Xu T.S., Dai X., Liu T.Y., Zhang Y.Y., Wang S., Liu Y.G., Shi H.F., Zhang Y.F. (2024). Fuling-Zexie formula attenuates hyperuricemia-induced nephropathy and inhibits JAK2/STAT3 signaling and NLRP3 inflammasome activation in mice. J. Ethnopharmacol..

[50] Deng A.P., Li Y., Wu Z.T., Liu T., Kang L.P., Nan T.G., Zhan Z.L., Guo L.P. (2016). Advances in studies on chemical compositions of *Atractylodes lancea* and their biological activities. China J. Chin. Mater. Med..

[51] Liu X., Liang L., Cai G., Guo Y., Gong J. (2024). Multivariate approach to assess the bioactive compounds of *Atractylodes chinensis* (DC.) Koidz in different harvest periods. J. Chromatogr. B.

[52] Li X.Y., Wang B., Guan D.X., Zhou J.J., Men Z., Sun Y.T. (2025). A research method of compatibility mechanism of traditional Chinese medicine prescription-taking Yupingfeng San as an example. J. Ethnopharmacol..

[53] Yao L.Y., Liu J.N., Pang Y.L., Wang H.M. (2025). Ultrasonic-assisted deep eutectic solvent extraction and identification of phenolic compounds from *Atractylodes chinensis* adventitious root culture. J. Chromatogr. A.

[54] Yue Y.Z., Zeng L., Wang X.P., Su L.L., Sun M.M., Wu B.S., Yan S. (2019). Loading of AgNPs onto the surface of boron nitride nanosheets for determination of scopoletin in *Atractylodes macrocephala*. Sci. Rep..

[55] Xia Z.X., Li Q., Tang Z.Y. (2023). Network pharmacology, molecular docking, and experimental pharmacology explored Ermiao wan protected against periodontitis via the PI3K/AKT and NF-ΚB/MAPK signal pathways. J. Ethnopharmacol..

[56] Feng Z.M., Xu K., Wang W., Du N., Zhang J.H., Yang Y.N., Jiang J.S., Zhang P.C. (2018). Two new thiophene polyacetylene glycosides from *Atractylodes lancea*. J. Asian Nat. Prod. Res..

[57] Cao H., Liu C., Chen G., Yao D., Xiao Y., Liu Y. (2024). Metabolic regularity of intestinal flora on alcohol extracts of Atractylodis Rhizoma before and after stir-frying and incremental component atractyloside A by in vitro co-incubation method. Chin. Tradit. Herb. Drugs.

[58] Zhou J., Tang W., Chen J. (2020). A UPLC-QTOF-MS/MS Method for the Characterization of Chemical Constituents of *Atractylodes lancea* (Thunb.) DC. and *Atractylodes chinensis* (DC.) Koidz. Pharm. Clin. Res..

[59] Zhang A., Zhu J., Gu W., Huang M., Wang S. (2025). The optimization of total flavonoid extraction process from the aboveground parts of *Atractylodes lancea* by orthogonal test and response surface methodology. China Feed.

[60] Cho H.D., Kim U., Suh J.H., Eom H.Y., Kim J., Lee S.G., Choi Y.S., Han S.B. (2016). Classification of the medicinal plants of the genus *Atractylodes* using high-performance liquid chromatography with diode array and tandem mass spectrometry detection combined with multivariate statistical analysis. J. Sep. Sci..

[61] Kim Y.C., Jun M., Jeong W.S., Chung S.K. (2005). Antioxidant properties of flavone C-glycosides from *Atradylodes japonica* leaves in human low-density lipoprotein oxidation. J. Food Sci..

[62] Wang X., Han Y., Wang X., Zhang Y. (2026). Studies on Chemical Composition, Pharmacological Effect, Clinical Application of Pingwei Powder and Its Quality Marker Prediction Analysis. J. Liaoning Univ. Tradit. Chin. Med..

[63] Xu S.Z., Qi X.J., Liu Y.Q., Liu Y.H., Lv X., Sun J.Z., Cai Q. (2018). UPLC-MS/MS of Atractylenolide I, Atractylenolide II, Atractylenolide III, and Atractyloside A in Rat Plasma after Oral Administration of Raw and Wheat Bran-Processed Atractylodis Rhizoma. Molecules.

[64] Zhuang D., Qin J., Wang H., Miao W., Lv G. (2021). Medicinal compositions of Atractylodis rhizoma: A review. Chin. J. Bioprocess Eng..

[65] Ye Y., Chou G.X., Wang H., Chu J.H., Fong W.F., Yu Z.L. (2011). Effects of Sesquiterpenes Isolated from Largehead Atractylodes Rhizome on Growth, Migration, and Differentiation of B16 Melanoma Cells. Integr. Cancer Ther..

[66] Chen H.P., Yang K., You C.X., Zheng L.S., Cai Q., Wang C.F., Du S.S. (2015). Repellency and Toxicity of Essential Oil from *Atractylodes chinensis* Rhizomes against *Liposcelis bostrychophila*. J. Food Process. Preserv..

[67] Choi S.W., Lee K.S., Lee J.H., Kang H.J., Lee M.J., Kim H.Y., Park K.I., Kim S.L., Shin H.K., Seo W.D. (2016). Suppression of Akt-HIF-1α signaling axis by diacetyl atractylodiol inhibits hypoxia-induced angiogenesis. Biomol. Biomed. Rep..

[68] Nakai Y., Kido T., Hashimoto K., Kase Y., Sakakibara I., Higuchi M., Sasaki H. (2003). Effect of the rhizomes of *Atractylodes lancea* and its constituents on the delay of gastric emptying. J. Ethnopharmacol..

[69] Zhang C., Wang S., Li Q., Zhang Y., He Y.Q., Yan B., Zhou L., Guo L. (2025). Metabolomic profiling and chemical marker identification in medicinal plants of Atractylodes. Sci. Tradit. Chin. Med..

[70] Yao D., Ma C.Y., Ke C., Wang D.P., Xu K., Liu Y.J., Qu L.H. (2025). Integrating transcriptomics, metabolomics, and microbiomics to explore the mechanism of action of bran-fried *Atractylodes lancea* rhizome polysaccharide in ameliorating the enhanced pharmacological effects of dextran sodium sulfate-induced colitis. J. Ethnopharmacol..

[71] Ye Q.Y., Jiang Y., Wu D., Cai J.W., Jiang Z.T., Zhou Z., Liu L.Y., Ling Q.H., Wang Q., Zhao G. (2023). Atractylodin alleviates nonalcoholic fatty liver disease by regulating Nrf2-mediated ferroptosis. Heliyon.

[72] Zhang Z., Tian Y., Qiao X., Li H., Ouyang L., Li X., Geng X., Xiao L., Ma Y., Li Y. (2025). Integrated Analysis of Terpenoid Profiles and Full-Length Transcriptome Reveals the Central Pathways of Sesquiterpene Biosynthesis in *Atractylodes chinensis* (DC.) Koidz. Int. J. Mol. Sci..

[73] Kitajima J., Kamoshita A., Ishikawa T., Takano A., Fukuda T., Isoda S., Ida Y. (2003). Glycosides of *Atractylodes lancea*. Chem. Pharm. Bull..

[74] Zhang H.L., Wang Y.M., Wang D.M., Sun J.K., Zhang Z.G., Wang J.H., Liu J., Liu Q. (2022). Phytochemical Profiling of Different Processed Products from Atractyloidis Rhizome using UHPLC/Q-TOF-MS. Pharmacogn. Mag..

[75] Singhuber J., Baburin I., Kählig H., Urban E., Kopp B., Hering S. (2012). GABA_A_ receptor modulators from Chinese herbal medicines traditionally applied against insomnia and anxiety. Phytomedicine.

[76] Ma Z., Liu G., Yang Z., Zhang G., Sun L., Wang M., Ren X. (2023). Species Differentiation and Quality Evaluation for Atractylodes Medicinal Plants by GC/MS Coupled with Chemometric Analysis. Chem. Biodivers..

[77] Wang W., Jiang Y., Song B., Tang X., Wu H., Jin Z., Chen L. (2023). Discovery of quality markers in the rhizome of *Atractylodes chinensis* using GC–MS fingerprint and network pharmacology. Arab. J. Chem..

[78] Gao Y., Chen H., Li W., Zhang Y., Luo J., Zhao L., Shi F., Ye G., He X., Xu Z. (2022). Chloroform extracts of *Atractylodes chinensis* inhibit the adhesion and invasion of *Salmonella typhimurium*. Biomed. Pharmacother..

[79] Zhang X.C., Zhu L., Li X.Y., Liu L.C., Lai P.X. (2021). Chemical Composition, and Evaluation of Antibacterial, Antibiofilm and Synergistic Effects with Conventional Antibiotics of Essential Oil from *Mallotus repandus*. Rec. Nat. Prod..

[80] Ghavam M., Bacchetta G., Castangia I., Manca M.L. (2025). *Phlomoides molucelloides* (Bunge) Salmaki essential oil: A traditional remedy revitalized for modern antimicrobial challenges. Inflammopharmacology.

[81] Ladjel-Mendil A., Amarni M., Chelghoum H., Lacheheb S., Moussa H., Aboumustapha M., Chebrouk F., Boudjelal A., Kebir M., Benguerba Y. (2025). Essential oil from aerial parts of *Salvia sclarea*: A comprehensive study of antibacterial activity through chemical profiling, molecular interactions, and predictive modeling using QSAR_KNN_PCA. J. Essent. Oil Bear. Plants.

[82] Prabaharan J., Prabakaran M., Prabhakaran M., Abinaya K., Krishnan N., Karen D.S., Veena J., Dhanbalan A.K., Devadasan V., Gopinath S.C.B. (2025). Comparison on extracted metabolites from different regions grown *Murraya koenigii* and validation by antibacterial, antioxidant, and molecular docking studies. Biomass Convers. Biorefinery.

[83] Hassan W.H.B., Ghani A.E.A., Taema E.A., Yahya G., El-Sadek M.E., Mansour B., Abdel-Halim M.S., Arafa A.M. (2025). Chemical profile, virtual screening, and virulence-inhibiting properties of *Sphagneticola trilobata* L. essential oils against *Pseudomonas aeruginosa*. Sci. Rep..

[84] Ben Hassine D., El Euch S.K., Rahmani R., Ghazouani N., Kane R., Abderrabba M., Bouajila J. (2021). Clove Buds Essential Oil: The Impact of Grinding on the Chemical Composition and Its Biological Activities Involved in Consumer’s Health Security. BioMed Res. Int..

[85] Qiu B., Wei F., Su J., Hao W., Zhou J., Zhao J., Wang Y., Qu Z. (2022). The Effects of β-Pinene, a Pine Needle Oil Monoterpene, on Adenovirus Type 3. Bull. Exp. Biol. Med..

[86] Madia V.N., De Angelis M., De Vita D., Messore A., De Leo A., Ialongo D., Tudino V., Saccoliti F., De Chiara G., Garzoli S. (2021). Investigation of *Commiphora myrrha* (Nees) Engl. Oil and Its Main Components for Antiviral Activity. Pharmaceuticals.

[87] Zanello P.R., Koishi A.C., Rezende C.D., Oliveira L.A., Pereira A.A., de Almeida M.V., dos Santos C.N.D., Bordignon J. (2015). Quinic acid derivatives inhibit dengue virus replication in vitro. Virol. J..

[88] Han J.C., Zhu X.Y., Gao Z.H., Xiao Y., Zhang J.X., Wang P., Fang J.B., Li Y.Q., Zhu Y.L., Li Y. (2023). Antiviral effects of Atractyloside A on the influenza B virus (*Victoria strain*) infection. Front. Microbiol..

[89] Nemoto K., Takikawa H., Ogura Y. (2023). Syntheses of (+)-costic acid and structurally related eudesmane sesquiterpenoids and their biological evaluations as acaricidal agents against *Varroa destructor*. J. Pestic. Sci..

[90] Rodríguez-Morales P., Franklin R.A. (2023). Macrophage phenotypes and functions: Resolving inflammation and restoring homeostasis. Trends Immunol..

[91] Citrin K.M., Chaube B., Fernández-Hernando C., Suárez Y. (2025). Intracellular endothelial cell metabolism in vascular function and dysfunction. Trends Endocrinol. Metab. TEM.

[92] Silveira Rossi J.L., Barbalho S.M., Reverete de Araujo R., Bechara M.D., Sloan K.P., Sloan L.A. (2022). Metabolic syndrome and cardiovascular diseases: Going beyond traditional risk factors. Diabetes/Metab. Res. Rev..

[93] Oda H., Annibaldi A., Kastner D.L., Aksentijevich I. (2025). Genetic Regulation of Cell Death: Insights from Autoinflammatory Diseases. Annu. Rev. Immunol..

[94] Mayerhofer C., Freedman R.A., Parsons H.A., Partridge A.H., Miller P.G. (2025). Clonal Hematopoiesis in Women with Breast Cancer. J. Clin. Oncol..

[95] Zhao X.L., Yu L., Zhang S.D., Ping K., Ni H.Y., Qin X.Y., Zhao C.J., Wang W., Efferth T., Fu Y.J. (2020). Cryptochlorogenic acid attenuates LPS-induced inflammatory response and oxidative stress via upregulation of the Nrf2/HO-1 signaling pathway in RAW 264.7 macrophages. Int. Immunopharmacol..

[96] Liu Y.H., Tang X., Zhang H.Z., Zheng L.Y., Lai P., Guo C., Ma J.F., Chen H.B., Qiu L.X. (2024). Terpinen-4-ol Improves Lipopolysaccharide-Induced Macrophage Inflammation by Regulating Glutamine Metabolism. Foods.

[97] An X., Yu W., Liu J., Tang D., Yang L., Chen X. (2024). Oxidative cell death in cancer: Mechanisms and therapeutic opportunities. Cell Death Dis..

[98] Huang X., Xie M., Wang Y., Lu X., Mei F., Zhang K., Yang X., Chen G., Yin Y., Feng G. (2025). *Porphyromonas gingivalis* aggravates atherosclerotic plaque instability by promoting lipid-laden macrophage necroptosis. Signal Transduct. Target. Ther..

[99] Dhapola R., Beura S.K., Sharma P., Singh S.K., HariKrishnaReddy D. (2024). Oxidative stress in Alzheimer’s disease: Current knowledge of signaling pathways and therapeutics. Mol. Biol. Rep..

[100] Takatsuka M., Goto S., Kobayashi K., Otsuka Y., Shimada Y. (2022). Evaluation of pure antioxidative capacity of antioxidants: ESR spectroscopy of stable radicals by DPPH and ABTS assays with singular value decomposition. Food Biosci..

[101] Yang X., Liu Y., Cao J., Wu C., Tang L., Bian W., Chen Y., Yu L., Wu Y., Li S. (2025). Targeting epigenetic and post-translational modifications of NRF2: Key regulatory factors in disease treatment. Cell Death Discov..

[102] Tacke F., Puengel T., Loomba R., Friedman S.L. (2023). An integrated view of anti-inflammatory and antifibrotic targets for the treatment of NASH. J. Hepatol..

[103] Liu F., Wang Y., Li D., Yang T. (2024). Atractylodin ameliorates lipopolysaccharide-induced depressive-like behaviors in mice through reducing neuroinflammation and neuronal damage. J. Neuroimmunol..

[104] Song G.Y., Kim S.M., Back S., Yang S.B., Yang Y.M. (2024). Atractylodes Lancea and Its Constituent, Atractylodin, Ameliorates Metabolic Dysfunction-Associated Steatotic Liver Disease via AMPK Activation. Biomol. Ther..

[105] Tsai M.C., Wang C.C., Tsai I.N., Yu M.H., Yang M.Y., Lee Y.J., Chan K.C., Wang C.J. (2024). Improving the Effects of Mulberry Leaves and Neochlorogenic Acid on Glucotoxicity-Induced Hepatic Steatosis in High Fat Diet Treated db/db Mice. J. Agric. Food Chem..

[106] Akcakavak G., Kazak F., Deveci M.Z.Y. (2023). Eucalyptol Protects against Cisplatin-Induced Liver Injury in Rats. Biol. Bull..

[107] Li Y.J., Guo M.Y., Qin W.Q., Li J.N., Li Y.F., Zhang F.K., Xue X.Y., Li S., Qu J.R., Liu R.P. (2025). Senkyunolide A ameliorates cholestatic liver fibrosis by controlling CLCC1-mediated endoplasmic reticulum Ca^2+^ release. Acta Pharmacol. Sin..

[108] Sun J., Jiang Y., Wang B., Yang J., Chen Y., Luo H., Chen T., Xiao C., Weng L. (2024). Structural characterization of the polysaccharides from *Atractylodes chinensis* (DC.) Koidz. and the protective effection against alcohol-induced intestinal injury in rats. Int. J. Biol. Macromol..

[109] Zhang Y., Liu Y., Wang J., Jiang Z., Zhang L., Cui Y., Zhao D., Wang Y. (2022). Atractylenolide II inhibits tumor-associated macrophages (TAMs)-induced lung cancer cell metastasis. Immunopharmacol. Immunotoxicol..

[110] Wu Y., Dai S., Zhang Y., Li Z., Zhu B., Liu Q., Wo L., Yu Z., Yuan X., Dou X. (2024). Atractylenolide II combined with Interferon-γ synergistically ameliorates colorectal cancer progression in vivo and in vitro by blocking the NF-kB p65/PD-L1 pathway. J. Cancer.

[111] Fan M., Gu X., Zhang W., Shen Q., Zhang R., Fang Q., Wang Y., Guo X., Zhang X., Liu X. (2022). Atractylenolide I ameliorates cancer cachexia through inhibiting biogenesis of IL-6 and tumour-derived extracellular vesicles. J. Cachexia Sarcopenia Muscle.

[112] Bourebaba N., Kornicka-Garbowska K., Marycz K., Bourebaba L., Kowalczuk A. (2021). *Laurus nobilis* ethanolic extract attenuates hyperglycemia and hyperinsulinemia-induced insulin resistance in HepG2 cell line through the reduction of oxidative stress and improvement of mitochondrial biogenesis—Possible implication in pharmacotherapy. Mitochondrion.

[113] Paul R.K., Ahmad I., Patel H., Raza K. (2025). Antidiabetic activity of the extracted oil from an Indian indigenous plant, *Amberboa ramosa*: Evidences from in silico, in vitro and enzyme inhibition kinetic studies. Chem. Pap..

[114] Kim D.Y., Kang M.K., Lee E.J., Kim Y.H., Oh H., Kim S.I., Oh S.Y., Na W., Kang Y.H. (2020). Eucalyptol Inhibits Amyloid-β-Induced Barrier Dysfunction in Glucose-Exposed Retinal Pigment Epithelial Cells and Diabetic Eyes. Antioxidants.

[115] Xu Z., Zhu J., Gao P., Zhu X., Zhang Y., Liu X. (2025). Essential Oil from Tetrapanax papyrifer (Hook.) K. Koch: Chemical Composition, Antioxidant Activity, α-Glucosidase Inhibitory Effect Integrating Molecular Docking Analysis. Chem. Biodivers..

[116] Santos E.S., Abrantes Coelho G.L., Saraiva Fontes Loula Y.K., Saraiva Landim B.L., Fernandes Lima C.N., Tavares de Sousa Machado S., Pereira Lopes M.J., Soares Gomes A.D., Martins da Costa J.G., Alencar de Menezes I.R. (2022). Hypoglycemic, Hypolipidemic, and Anti-Inflammatory Effects of Beta-Pinene in Diabetic Rats. Evid.-Based Complement. Altern. Med. eCAM.

[117] Wu W., Liao Y., Wei L., Feng X., Dai Y., Liu Q., Feng S. (2025). Ultrasound-Optimized Extraction and Multi-Target Mechanistic Analysis of Antioxidant and Hypoglycemic Effects of *Amomum villosum* Essential Oil. Foods.

[118] El Azab E.F., Mostafa H.S. (2022). Geraniol ameliorates the progression of high fat-diet/streptozotocin-induced type 2 diabetes mellitus in rats via regulation of caspase-3, Bcl-2, and Bax expression. J. Food Biochem..

[119] Zhou Y. (2020). The Protective Effects of Cryptochlorogenic Acid on β-Cells Function in Diabetes in vivo and vitro via Inhibition of Ferroptosis. Diabetes Metab. Syndr. Obes. Targets Ther..

[120] Shaedi N., Naharudin I., Choo C.Y., Wong T.W. (2021). Design of oral intestinal-specific alginate-vitexin nanoparticulate system to modulate blood glucose level of diabetic rats. Carbohydr. Polym..

[121] Xue J.C., Yuan S., Meng H., Hou X.T., Li J., Zhang H.M., Chen L.L., Zhang C.H., Zhang Q.G. (2023). The role and mechanism of flavonoid herbal natural products in ulcerative colitis. Biomed. Pharmacother..

[122] Qu L., Lin X., Liu C., Ke C., Zhou Z., Xu K., Cao G., Liu Y. (2021). Atractylodin Attenuates Dextran Sulfate Sodium-Induced Colitis by Alleviating Gut Microbiota Dysbiosis and Inhibiting Inflammatory Response Through the MAPK Pathway. Front. Pharmacol..

[123] Li Y.X., Liu J., Li F. (2024). Hinesol attenuates DSS-induced ulcerative colitis through the suppression of Src-mediated NF-κB and chemokine signaling pathway. Cell Biochem. Biophys..

[124] Shentu C., Mao M., Zhu J., Meng Q., Qian H., Li X., Zhang S., Ding B., Dai S., Yuan X. (2025). Atractylenolide III Ameliorates Ulcerative Colitis By Targeting IL-17RA to Suppress Macrophage M1 Polarization. J. Agric. Food Chem..

[125] Hao J., Wan Q., Chen C. (2025). Atractylenolide III Promotes Astrocyte Aβ Clearance by Up-regulating AQP4 to Improve Alzheimer’s Disease. Folia Biol..

[126] Sun T., Li Z., Xiao B., Yang J., Han M., Zhang J., Liu S., Ma H., Song J., Su Y. (2026). Multi-target neuroprotection of *Atractylodes macrocephala* ethyl acetate extract against Alzheimer’s disease: From bioactivity-guided screening to mechanistic validation. J. Ethnopharmacol..

[127] Nallasamy P., Srinivasan G., Thatchanamoorthy T., Jeyaraj W., Natarajan S. (2025). Dual functionality of hydroxyapatite nanocarriers in neurodegenerative biomarker management as immunosensor and neuroprotective therapeutic system. Microchem. J..

[128] Meyer Z.A., Rambharose S. (2026). Gold nanoparticles incorporating rutin hydrate for targeting oxidative stress-driven neurodegeneration. BioMetals.

[129] Garlet Q.I., Rodrigues P., Barbosa L.B., Londero A.L., Mello C.F., Heinzmann B.M. (2019). *Nectandra grandiflora* essential oil and its isolated sesquiterpenoids minimize anxiety-related behaviors in mice through GABAergic mechanisms. Toxicol. Appl. Pharmacol..

[130] Nguyen L.T.H., Nguyen N.P.K., Tran K.N., Choi H.J., Moon I.S., Shin H.M., Yang I.J. (2024). Essential oil of *Pterocarpus santalinus* L. alleviates behavioral impairments in social defeat stress-exposed mice by regulating neurotransmission and neuroinflammation. Biomed. Pharmacother..

[131] Asle-Rousta M., Abdollahi M., Aghajari H.M., Peirovy Y. (2025). Eucalyptol Attenuates Lead-Induced Anxiety-like Behaviors by Suppressing Oxidative Stress and Neuroinflammation, Modulating SIRT1/NF-κB Signaling, and Upregulating BDNF Expression. Biol. Trace Elem. Res..

[132] Hosseini A., Pourheidar E., Rajabian A., Asadpour E., Hosseinzadeh H., Sadeghnia H.R. (2023). Linalool attenuated ischemic injury in PC12 cells through inhibition of caspase-3 and caspase-9 during apoptosis. Food Sci. Nutr..

[133] Wee A.S., Thew H.Y., Liew S.Y., Tan W.N., Khaw K.Y. (2024). Anti-cholinesterase profile of *Murraya koenigii* (L.) Spreng essential oil and its chemical constituents. J. Essent. Oil Bear. Plants.

[134] Nallasamy P., Rajamohamed B.S., Jeyaraman J., Kathirvel B., Natarajan S. (2023). Regenerative marine waste towards CaCO_3_ nanoformulation for Alzheimer’s therapy. Environ. Res..

[135] Lee J.I., Choi J.H., Kwon T.W., Jo H.S., Kim D.G., Ko S.G., Song G.J., Cho I.H. (2023). Neuroprotective effects of bornyl acetate on experimental autoimmune encephalomyelitis via anti-inflammatory effects and maintaining blood-brain-barrier integrity. Phytomedicine.

[136] Faheem M., Khan A.U., Saleem M.W., Shah F.A., Ali F., Khan A.W., Li S. (2022). Neuroprotective Effect of Natural Compounds in Paclitaxel-Induced Chronic Inflammatory Pain. Molecules.

[137] Faheem M., Khan A.U., Shah F.A., Li S. (2022). Investigation of Natural Compounds for Therapeutic Potential in Streptozotocin-induced Diabetic Neuroinflammation and Neuropathic Pain. Front. Pharmacol..

[138] Latif K., Saneela S., Khan A.U. (2022). Ameliorative effect of carveol on scopolamine-induced memory impairment in rats. Iran. J. Basic Med. Sci..

[139] Muhammad A.J., Al-Baqami F.F., Alanazi F.E., Alattar A., Alshaman R., Rehman N.U., Riadi Y., Shah F.A. (2024). The Interplay of Carveol and All-Trans Retinoic Acid (ATRA) in Experimental Parkinson’s Disease: Role of Inflammasome-Mediated Pyroptosis and Nrf2. Neurochem. Res..

[140] Rafi K., Faizi S., Hussain S.S., Shamshad S., Versiani M.A., Simjee S.U. (2025). Quinic acid and its derivatives protect against phytohaemagglutinin-induced Alzheimer’s-like neurotoxicity in SH-SY5Y cells by down-regulating p38 MAPK signaling pathway. Toxicol. Appl. Pharmacol..

[141] Niaz M., Iftikhar K., Shahid M., Faizi S., Usman Simjee S. (2025). Quinic acid contributes to neurogenesis: Targeting Notch pathway a key player in hippocampus. Brain Res..

[142] Li S., Cai Y., Guan T., Zhang Y., Huang K., Zhang Z., Cao W., Guan X. (2024). Quinic acid alleviates high-fat diet-induced neuroinflammation by inhibiting DR3/IKK/NF-κB signaling via gut microbial tryptophan metabolites. Gut Microbes.

[143] Wang C., Song X., Zhang X., Li P., Wei W., Sun S., Chen Y. (2025). Multifunctional natural chlorogenic acid based nanocarrier for Alzheimer’s disease treatment. Mater. Today Bio.

[144] Qiao N., Wang Q., Tao Y., Wu J., Fang Y., Ni Y., Ding X. (2023). α-Cyperone ameliorates depression in mammary gland hyperplasia and chronic unpredictable mild stress rat by regulating hormone, inflammation, and oxidative stress. Immunopharmacol. Immunotoxicol..

[145] Huang B., Hu G., Zong X., Yang S., He D., Gao X., Liu D. (2023). α-Cyperone protects dopaminergic neurons and inhibits neuroinflammation in LPS-induced Parkinson’s disease rat model via activating Nrf2/HO-1 and suppressing NF-κB signaling pathway. Int. Immunopharmacol..

[146] Zhang Y., Miao L., Peng Q., Fan X., Song W., Yang B., Zhang P., Liu G., Liu J. (2022). Parthenolide modulates cerebral ischemia-induced microglial polarization and alleviates neuroinflammatory injury via the RhoA/ROCK pathway. Phytomedicine.

[147] Ding W., Cai C., Zhu X., Wang J., Jiang Q. (2022). Parthenolide ameliorates neurological deficits and neuroinflammation in mice with traumatic brain injury by suppressing STAT3/NF-κB and inflammasome activation. Int. Immunopharmacol..

[148] Park C.K., Choi S.J., Kim C.R., Shin H.R., Shin E.C., Kim Y.J., Cho T.J., Shin D.H., Kim J.K. (2025). Ethanolic Extract of *Rosa rugosa* Roots and Its Bioactive Compound, Oleamide, Prevented Amyloid β-Induced Oxidative Stress and Improved Behavioral Tests in Mice. Int. J. Mol. Sci..

[149] Shervin Prince S., Stanely Mainzen Prince P., Berlin Grace V.M. (2022). Valencene post-treatment exhibits cardioprotection via inhibiting cardiac hypertrophy, oxidative stress, nuclear factor- κB inflammatory pathway, and myocardial infarct size in isoproterenol-induced myocardial infarcted rats; A molecular study. Eur. J. Pharmacol..

[150] Song X., Wang L., Liu M., Pan R., Song J., Kong J. (2024). Atractylenolide II ameliorates myocardial fibrosis and oxidative stress in spontaneous hypertension rats. Technol. Health Care.

[151] Li J., Chen X., Li X., Tang J., Li Y., Liu B., Guo S. (2022). Cryptochlorogenic acid and its metabolites ameliorate myocardial hypertrophy through a HIF1α-related pathway. Food Funct..

[152] Stamatiou R., Anagnostopoulou M., Ioannidou-Kabouri K., Rapti C., Lazou A. (2024). Camphene as a Protective Agent in Myocardial Ischemia/Reperfusion Injury. Antioxidants.

[153] Liu T., Chen X., Sun Q., Li J., Wang Q., Wei P., Wang W., Li C., Wang Y. (2025). Valerenic acid attenuates pathological myocardial hypertrophy by promoting the utilization of multiple substrates in the mitochondrial energy metabolism. J. Adv. Res..

[154] Liu Y., Zhang B., Cai Q. (2020). Study on the pharmacodynamics and metabolomics of five medicinal species in *Atractylodes* DC. on rats with rheumatoid arthritis. Biomed. Pharmacother..

[155] Chuang C.H., Cheng Y.C., Lin S.C., Lehman C.W., Wang S.P., Chen D.Y., Tsai S.W., Lin C.C. (2019). Atractylodin Suppresses Dendritic Cell Maturation and Ameliorates Collagen-Induced Arthritis in a Mouse Model. J. Agric. Food Chem..

[156] Pan J., Cai Y., Zhang C., Xu S. (2023). Intra-articular delivery of geraniol encapsulated by pH/redox-responsive nanogel ameliorates osteoarthritis by regulating oxidative stress and inflammation. J. Mol. Histol..

[157] Yin C., Liu B., Wang P., Li X., Li Y., Zheng X., Tai Y., Wang C., Liu B. (2020). Eucalyptol alleviates inflammation and pain responses in a mouse model of gout arthritis. Br. J. Pharmacol..

[158] Liu D., Fu Q., Liu L.G., Li W., Qi F., Liu J., Shang L., Wang X., Yang F., Li J. (2024). Screening of potentially active compounds against rheumatoid arthritis in the Juan-Bi decoction using systems pharmacology and animal experiments. Front. Cell Dev. Biol..

[159] Chen S., Xu H., He Y., Meng C., Fan Y., Qu Y., Wang Y., Zhou W., Huang X., You H. (2024). Carveol alleviates osteoarthritis progression by acting on synovial macrophage polarization transformation: An in vitro and in vivo study. Chem.-Biol. Interact..

[160] Shao M., Lv D., Zhou K., Sun H., Wang Z. (2022). Senkyunolide A inhibits the progression of osteoarthritis by inhibiting the NLRP3 signalling pathway. Pharm. Biol..

[161] Shi K., Wang Y., Xiao Y., Tu J., Zhou Z., Cao G., Liu Y. (2023). Therapeutic effects and mechanism of Atractylodis rhizoma in acute lung injury: Investigation based on an Integrated approach. Front. Pharmacol..

[162] Xing H., Bai X., Pei X., Zhang Y., Zhang X., Chen S., Li D., Lv B., Wang X., Wu X. (2025). Synergistic anti-oxidative/anti-inflammatory treatment for acute lung injury with selenium based chlorogenic acid nanoparticles through modulating Mapk8ip1/MAPK and Itga2b/PI3k-AKT axis. J. Nanobiotechnol..

[163] Yang S., Ling Y., Zhao F., Li W., Song Z., Wang L., Li Q., Liu M., Tong Y., Chen L. (2022). Hymecromone: A clinical prescription hyaluronan inhibitor for efficiently blocking COVID-19 progression. Signal Transduct. Target. Ther..

[164] Li Y., Wang X., Zhao L., Pan B., Xu X., Zhu D. (2024). Dehydrocostus Lactone Ameliorates LPS-Induced Acute Lung Injury by Inhibiting PFKFB3-Mediated Glycolysis. J. Cell. Biochem..

[165] Liu H., Dong J., Xu C., Ni Y., Ye Z., Sun Z., Fan H., Chen Y. (2025). Acute lung injury: Pathogenesis and treatment. J. Transl. Med..

[166] Kim H., Choi J., Seo J., Lim H., Kang S.K. (2024). CKD-497 inhibits NF-kB signaling and ameliorates inflammation and pulmonary fibrosis in ovalbumin-induced asthma and particulate matter-induced airway inflammatory diseases. Front. Pharmacol..

[167] Zhang M., Wang C., Zhao R., Yuan J. (2025). Senkyunolide A alleviates asthma by inhibiting TGF-β/Smad2/3 signaling pathway and NLRP3 inflammasome activation. Biochem. Biophys. Res. Commun..

[168] Wang Z., Zhang S., Li Y., Zhou Z., Zhang X., Chen Y., Zhao Y., Ye C., Li J., Zhang N. (2025). Structural characterization and effect of activating autophagy and regulating oxidative stress of polysaccharide from fibrous roots of *Atractylodes chinensis*. Bioorganic Chem..

[169] Plirat W., Chaniad P., Phuwajaroanpong A., Septama A.W., Punsawad C. (2022). Phytochemical, Antimalarial, and Acute Oral Toxicity Properties of Selected Crude Extracts of Prabchompoothaweep Remedy in Plasmodium berghei-Infected Mice. Trop. Med. Infect. Dis..

[170] Wang K.-T., Chen L.-G., Yang L.-L., Ke W.-M., Chang H.-C., Wang C.-C. (2007). Analysis of the sesquiterpenoids in processed atractylodis rhizoma. Chem. Pharm. Bull..

[171] Shi K., Xiao Y., Dong Y., Wang D., Xie Y., Tu J., Xu K., Zhou Z., Cao G., Liu Y. (2022). Protective Effects of Atractylodis lancea Rhizoma on Lipopolysaccharide-Induced Acute Lung Injury via TLR4/NF-κB and Keap1/Nrf2 Signaling Pathways In Vitro and In Vivo. Int. J. Mol. Sci..

[172] Gao X., Ma D., Li K., Xing T., Liu X., Peng L., Chen D., Hao Z. (2023). Non-Targeted Metabolomics Combined with Chemometrics by UHPLC-Orbitrap-HRMS and Antioxidant Activity of *Atractylodes chinensis* (DC.) Koidez. from Eight Origins. Metabolites.

[173] Li Y., Jiang Z., Zhou Z., Zhang N., Cui X., Yu X., Zhao Y., Wang Z., Li J., Wu D. (2026). Differential toxic phenotypes and liver injury induced by Atractylenolides (I, II, and III): Insights from zebrafish (*Danio rerio*) models and network toxicology. Comp. Biochem. Physiol. Part C Toxicol. Pharmacol..

[174] Tshering G., Plengsuriyakarn T., Na-Bangchang K., Pimtong W. (2021). Embryotoxicity evaluation of atractylodin and β-eudesmol using the zebrafish model. Comp. Biochem. Physiol. Part C Toxicol. Pharmacol..

