# Supplementary material for: Herbal Textual Research, Phytochemistry, Pharmacology and Toxicity of Atractylodis Rhizoma: A Comprehensive Review
Source: Molecules. 2026 Mar 18;31(6):1015. doi: 10.3390/molecules31061015 (PMC13029298; doi:10.3390/molecules31061015)
Supplement: Supplementary file 1 [file molecules-31-01015-s001.zip › molecules-4185077-supplementary.pdf]

Supporting Information

Herbal Textual Research, Phytochemistry, Pharmacology and Toxicity of Atractylodis Rhizoma: A Comprehensive Review

Jin Sun [a,1], Juhui Qiao [a,1*], Jian Tang [a], Nuo Cheng [a], Miaomiao Gao [a], Jingrong Yang [b**], Baixin Kou [c***]

a School of Chinese Medicine, Bozhou University, Bozhou 236800, China
b School of Medicine, Changchun Sci-Tech University, Changchun 130600, China
c School of Pharmaceutical Sciences, Changchun University of Chinese Medicine, Changchun 130117, China
[1]These authors contributed equally to this work.

This file contains:

Table S1. Traditional preparations of Atractylodis Rhizoma
Table S2. Modern preparations of Atractylodis Rhizoma
Table S3. Antimicrobial effect of Atractylodis Rhizoma
Table S4. Anti-inflammatory effect of Atractylodis Rhizoma
Table S5. Antioxidant effect of Atractylodis Rhizoma
Table S6. Hepatoprotective effect of Atractylodis Rhizoma
Table S7. Anti-cancer effect of Atractylodis Rhizoma
Table S8. Intestinal regulatory function of Atractylodis Rhizoma

[*] Corresponding authors. No. 2266, Tangwang Avenue, Qiaocheng District, Bozhou, Anhui, 236800, China.
[**]Corresponding author. No. 616, Xueyuan Street, Shuangyang District, Changchun, Jilin, 130600, China.
[***]Corresponding author. No. 1035, Boshuo Road, Nanguan District, Changchun, Jilin, 130117, China.
E-mail address: Jin Sun (sunjin0509@163.com); Juhui Qiao (sunqiao150509@163.com); Jian Tang (jt.u@hotmail.com); Nuo Cheng (19565882214@163.com); Miaomiao Gao (18226068027@163.com); Jingrong Yang (13894298316@163.com); Baixin Kou (24104800112@stu.ccucm.edu.cn)

Table S1. Traditional preparations of Atractylodis Rhizoma

Date of Publication	References	Preparation name	Compositiona crude drug names	Traditional uses	Efficacy
Han	Hua Tuo Shen Fang	Hua Tuo Zhi Huang Shui Shen Fang	Proia, Atractylodis Rhizoma, Schizonepetae Herba, Taraxaci Herba, Saposhnikoviae Radix, Scutellariae Radix, Pinelliae Rhizoma, Angelicae Sinensis Radix	Oral use	Treating sores
		Hua Tuo Zhi Yang Hu Chuang Shen Fang	Proia Trichosanthis Radix, Glycyrrhizae Radix Et Rhizoma Praeparata Cum Melle, Atractylodis Macrocephalae Rhizoma, Atractylodis Rhizoma, Taraxaci Herba, Alismatis Rhizoma, Polyporus, Angelicae Dahuricae Radix, Notopterygii Rhizoma Et Radix Citri Reticulatae Pericarpium, Processed Pinelliae Rhizoma	Oral use	Treating lower lip and submandibular sores
		Hua Tuo Zhi Bai Zhuo Shen Fang	Proia, Salt processed Alpiniae Oxyphyllae Fructus, Atractylodis Macrocephalae Rhizoma, Atractylodis Rhizoma, Cimicifugae Rhizoma, Bupleuri Radix, Glycyrrhizae Radix Et Rhizoma Praeparata Cum Melle, Zingiberis Rhizoma Recens	Oral use	Treating nocturnal emissions
Tang	Xian Shou Li Shang Xu Duan Mi Fang	Ru Xiang San	Cinnamomi Cortex, Zingiberis Rhizoma, Achyranthis Bidentatae Radix, Notopterygii Rhizoma Et Radix, Angelicae Dahuricae Radix, Chuanxiong Rhizome, Asari Radix Et Rhizoma, Curcumae Longae Rhizoma, Drynariae Rhizoma, Angelicae Sinensis Radix, Paeoniae Radix Alba, Aconiti kusnezoffii radix, Aconiti Radix, Atractylodis Rhizoma, Platycodonis Radix, Vignae Semen, Olibanum, Myrrha, Polygoni Multiflori Radix, Momordicae Semen	Take warm, any time	Treating bruises and contusions
		Bie Jia San I	Cinnamomi Cortex, Chuanxiong Rhizoma, Angelicae Dahuricae Radix, Gentianae Macrophyllae Radix,	Drink while hot	Treating the five wasting diseases and seven injuries

Date of Publication	References	Preparation name	Compositiona crude drug names	Traditional uses	Efficacy
		Yun Qi San	Vinegar-Processed Pelodisci Carapax, Asteris Radix Et Rhizoma, Ephedrae Herba, Notopterygii Rhizoma Et Radix Angelicae Sinensis Radix, Zingiberis Rhizoma, Citri Reticulatae Pericarpium, Atractylodis Rhizoma, Linderae Radix, Perillae Fructus, Platycodonis Radix, Bupleuri Radix, Aconiti Radix Anisi Stellati Fructus, Citri Reticulatae Pericarpium Viride, Processed Magnoliae Officinalis Cortex, Angelicae Dahuricae Radix, Linderae Radix, Armeniacae Semen Amarum, Citri Reticulatae Pericarpium, Hordei Fructus Germinatus, Peucedani Radix, Platycodonis Radix, Atractylodis Rhizoma, Glycyrrhizae Radix Et Rhizoma	Take on an empty stomach	Regulating Qi
		Wu Ji San	Atractylodis Rhizoma, Platycodonis Radix, Aurantii Fructus, Citri Reticulatae Pericarpium, Paeoniae Radix Alba, Angelicae Dahuricae Radix, Chuanxiong Rhizoma, Angelicae Sinensis Radix, Glycyrrhizae Radix Et Rhizoma, Cinnamomi Cortex, Proia, Pinelliae Rhizoma, Magnoliae Officinalis Cortex, Zingiberis Rhizoma, Ephedrae Herba	Drink while hot	Treating the Five Consumption Diseases and Seven Injuries
		Huo Xue Dan	Schizonepetae Herba, Liquidambaris Resina, Santali Albi Lignum, Dalbergiae Odoriferae Lignum, Aconiti Kusnezoffii Radix, Cinnamomi Cortex, Angelicae Sinensis Radix, Atractylodis Rhizoma, Notopterygii Rhizoma Et Radix, Bletillae Rhizoma, Sojae Semen Nigrum, Pheretima, Celosiae Semen, Moschus,	Oral use	Treating Bruises and Contusions

Date of Publication	References	Preparation name	Compositiona crude drug names	Traditional uses	Efficacy
			Chuanxiong Rhizoma, Trogopterori Faeces, Olibanum Myrrha, Aconiti Radix, Drynariae Rhizoma, Achyranthis Bidentatae Radix, Asari Radix Et Rhizoma, Mori Ramulus, Angelicae Dahuricae Radix, Paeoniae Radix Rubra, Pharbitidis Semen, Arisaematis Rhizoma, Pyritum, Castanea mollissima Blume, Trionychidae		
		Xian Zheng San	Cinnamomi Cortex, Angelicae Sinensis Radix, Corydalis Rhizoma, Angelicae Dahuricae Radix Atractylodis Rhizoma Paeoniae Radix Rubra Saposhnikoviae Radix Schizonepetae Herba	Topical application	Treatment of fractures
		Mu Xiang San	Aucklandiae Radix, Myristicae Semen, Ginseng Radix Et Rhizoma, Aconiti Radix Lateralis Praeparata, Angelicae Sinensis Radix, Zingiberis Rhizoma, Glycyrrhizae Radix Et Rhizoma, Citri Reticulatae Pericarpium, Atractylodis Rhizoma, Euodiae Fructus, Magnoliae Officinalis Cortex	Take warm before meals	Treating spleen deficiency with cold
Song	Tai Ping Sheng Hui Fang	Hou Po	Magnoliae Officinalis Cortex, Atractylodis Rhizoma, Chebulae Fructus, Angelicae Sinensis Radix, Zingiberis Rhizoma, Aucklandiae Radix, Amomi Fructus, Halloysitum Rubrum, Aconiti Radix Lateralis Praeparata	Oral use	Treating spleen deficiency with cold
		Fa Han Ji Xiao Fang	Atractylodis Rhizoma, Glycyrrhizae Radix Et Rhizoma, Gleditsiae Fructus Abnormalis, Ephedrae Herba	Oral use, regardless of the time	Treating typhoid fever
		Gan Cao San	Glycyrrhizae Radix Et Rhizoma, Tuckahoe with pine, Polygalae Radix, Atractylodis Rhizoma, Aurantii Fructus Immaturus	Take warm, regardless of time	Treatment for typhoid fever with intermittent pulse

Date of Publication	References	Preparation name	Compositiona crude drug names	Traditional uses	Efficacy
		Bie Jia San II	Pelodiscus Carapax, Atractylodis Rhizoma, Aconiti Radix Lateralis Praeparata, Glycyrrhizae Radix Et Rhizoma, Ginseng Radix Et Rhizoma, Astragali Radix, Cistanches Herba, Persicae Semen, Rehmanniae Radix Praeparata, Achyranthis Bidentatae, Radix Bupleuri, Radix Aurantii Fructus, Schisandrae Chinensis Fructus, Crassostreae Concha, Eucommiae Cortex	Take warm, regardless of time	Treatment for Debility Following Typhoid Fever
		Fang Feng San	Saposhnikoviae Radix, Alismatis Rhizoma, Crassostreae Concha, Atractylodis Rhizoma, Ramulus Cinnamomi	Take at any time	Treating wind-deficiency with profuse sweating
		Shi Gao San	Gypsum Fibrosum, Glycyrrhizae Radix Et Rhizoma, Atractylodis Rhizoma, Ephedrae Radix Et Rhizoma	Take at any time	Treating wind-deficiency with profuse sweating
		Qin Jiao San	Gentianae Macrophyllae Radix, Aconiti Radix Lateralis Praeparata, Gypsum Fibrosum, Acori Tatarinowii Rhizoma, Ephedrae Radix Et Rhizoma, Atractylodis Rhizoma, Cinnamomi Cortex, Saposhnikoviae Radix	Take at any time	Treating wind-deficiency with profuse sweating
		Wu She Ointment	Ptyas, Gastrodiae Rhizoma, Aconiti Radix Lateralis Praeparata, Bombyx Batryticatus, Aconiti Radix Lateralis Praeparata, Arisaematis Rhizoma, Cinnamomi Cortex, Asari Radix Et Rhizoma, Euodiae Fructus, Notopterygii Rhizoma Et Radix, Angelicae Sinensis Radix, Atractylodis Rhizoma, Saposhnikoviae Radix, Achyranthis Bidentatae Radix, Zanthoxyli Pericarpium, Scorpio, Momordicae Semen,	Topical application	Treating wind-induced urticaria and swelling

Date of Publication	References	Preparation name	Compositiona crude drug names	Traditional uses	Efficacy
		Hou Po Pill	Aurantii Fructus, Rhei Radix Et Rhizoma Angelicae Dahuricae Radix Magnoliae Officinalis Cortex, Poria, Ginseng Radix Et Rhizoma, Vinegar-Processed Pelodisci Carapax, Chebulae Fructus, Aucklandiae Radix, Citri Reticulatae Pericarpium, Aconiti Radix Lateralis Praeparata, Euodiae Fructus, Atractylodis Rhizoma, Zingiberis Rhizoma, Hordei Fructus Germinatus, Sparganii Rhizoma, Alpiniae oxyphyllae Fructus, Angelicae Sinensis Radix, Astragali Radix, Arecae Semen	Take before dinner	Treating spleen fatigue
		Fu Shen San	Indian Bread with Pine, Ginseng Radix Et Rhizoma, Rehmanniae Radix Preparata, Crassostreae Concha, Ophiopogonis Radix, Astragali Radix, Ziziphi Spinosae Semen, Os Draconis, Schisandrae Chinensis Fructus, Atractylodis Rhizoma, Glycyrrhizae Radix Et Rhizoma	Take warm, regardless of time	Treating consumptive diseases
		Zhi Gan San I	Atractylodis Rhizoma, Bupleuri Radix, Platycodonis Radix, Paeoniae Radix Rubra, Citri Reticulatae Pericarpium, Amomi Fructus, Chebulae Fructus, Alpiniae Officinarum Rhizoma	Take before meals	Treatment for cold-induced cough
		Mu Li San	Crassostreae Concha, Os Draconis, Cinnamomi Cortex, Spine of Common Jujube, Paeoniae Radix Alba, Atractylodis Rhizoma, Glycyrrhizae Radix Et Rhizoma	Take before meals	Treating consumptive disease with nocturnal emissions
		Jie Geng San	Platycodonis Radix, Angelicae Sinensis Radix, Atractylodis Rhizoma, Chebulae Fructus, Chuanxiong Rhizoma, Bupleuri Radix, Pelodiscus Carapax, Rhei Radix Et	Take warm before meals	Treating bone steaming

Date of Publication	References	Preparation name	Compositiona crude drug names	Traditional uses	Efficacy
			Rhizoma, Paeoniae Radix Rubra		
		Mu Xiang Pill	Aucklandiae Radix, Pelodiscus Carapax, Sparganii Rhizoma, Paeoniae Radix Rubra, Rhei Radix Et Rhizoma, Citri Reticulatae Pericarpium, Atractylodis Rhizoma, Platycodonis Radix, Arecae Semen, Pruni Semen, Bupleuri Radix	Take before meals	Treating bone steaming
		Hou Po San I	Magnoliae Officinalis Cortex, Chebulae Fructus, Aucklandiae Radix, Aurantii Fructus, Atractylodis Rhizoma, Angelicae Sinensis Radix, Platycodonis Radix, Citri Reticulatae Pericarpium	Take warm, regardless of time	Treating distension and pain
		Bai Hua She Jian	Bungarus Parvus, Erythrina variegata, Angelicae Dahuricae Radix, Saposhnikoviae Radix, Notopterygii Rhizoma Et Radix, Atractylodis Macrocephalae Rhizoma, Aconiti Radix Lateralis Praeparata, Arisaematis Rhizoma, Pinelliae Rhizoma, Peucedani Radix, Asari Radix Et Rhizoma, Scorpio, Cinnamomi Cortex, Zanthoxyli Pericarpium Momordicae Semen, Angelicae Sinensis Radix, Euodiae Fructus, Atractylodis Rhizoma	Oral use	Treating skin sores
		Shu Gan Di Huang San	Rehmanniae Radix Preparata, Paeoniae Radix Alba Bupleuri Radix, Vinegar-Processed Pelodisci Carapax, Angelicae Sinensis Radix, Atractylodis Rhizoma, Curcumae Longae Rhizoma, Amber, Notopterygii Rhizoma Et Radix, Chuanxiong Rhizoma, Aucklandiae Radix, Magnoliae Officinalis Cortex, Cinnamomi Cortex, Citri Reticulatae Pericarpium, Achyranthis Bidentatae Radix,	Drink while hot, regardless of the time	Treating Heart and Abdominal Pain

Date of Publication	References	Preparation name	Compositiona crude drug names	Traditional uses	Efficacy
		Shun Qi San	Magnoliae Officinalis Cortex, Anisi Stellati Fructus, Citri Reticulatae Pericarpium, Atractylodis Rhizoma, Aurantii Fructus, Chuanxiong Rhizoma, Platycodonis Radix, Armeniacae Semen Amarum, Processed Angelicae Dahuricae Radix, Glycyrrhizae Radix Et Rhizoma Praeparata Cum Melle, Ephedrae Herba	Oral use	Treating typhoid fever
		Cao Huan Dan	Curculiginis Rhizoma, Notopterygii Rhizoma Et Radix, Saposhnikoviae Radix, Cibotii Rhizoma, Atractylodis Macrocephalae Rhizoma, Proia, Zingiberis Rhizoma, Acori Tatarinowii Rhizoma, Pharbitidis Semen, Clematidis Radix Et Rhizoma, Atractylodis Rhizoma	Oral use	Regulates the triple energiser, sharpens the senses, and nourishes the vital essence
Song	Bo Ji Fang	Ping Wei SanI	Magnoliae Officinalis Cortex, Glycyrrhizae Radix Et Rhizoma Praeparata Cum Melle, Rice water-processed Atractylodis Rhizoma, Citri Reticulatae Pericarpium, Ginseng Radix Et Rhizoma, Proia	Take on an empty stomach	Treatment for disharmony of the spleen and stomach
		Ding Xiang San	Myristicae Semen, Ginseng Radix Et Rhizoma, Proia, Atractylodis Rhizoma, Aristolochia debilis, Euodiae Fructus, Caryophylli Flos, Magnoliae Officinalis Cortex, Sparganii Rhizoma, Zingiberis Rhizoma, Paeoniae Radix Alba Glycyrrhizae Radix Et Rhizoma	Take on an empty stomach	Warm the spleen and aid digestion, alleviate heart and abdominal pain
		Ta Qi San	Anisi Stellati Fructus, Aurantii Fructus, Proia, Ginseng Radix Et Rhizoma, Citri Reticulatae Pericarpium, Citri Reticulatae Pericarpium Viride, Glycyrrhizae Radix Et Rhizoma, Atractylodis Rhizoma, Caryophylli Flos, Zingiberis Rhizoma, Alpiniae Officinarum Rhizoma	Drink while hot	Treating deficiency-related qi rising and surging, causing chest fullness and oppression

Date of Publication	References	Preparation name	Compositiona crude drug names	Traditional uses	Efficacy
		Qiong Qiang San	Processed Schizonepetae Spica, Processed Arctii Fructus, Equiseti Hiemalis Herba, Atractylodis Rhizoma	Take on an empty stomach	Treating itchy, swollen and painful eyes
		Chan Tui San	Cicadae Periostracum, Lycii Cortex, Coptidis Rhizoma, Chrysanthemi Flos, Atractylodis Macrocephalae Rhizoma, Atractylodis Rhizoma, Moutan Cortex, Gentianae Radix Et Rhizoma, Melo Semen	Oral use	Treating redness, pain, dryness and swelling of the eyes
		Xian Zhu San	Atractylodis Rhizoma, Equiseti Hiemalis Herba, Cicadae Periostracum, Eriocauli Flos, Glycyrrhizae Radix Et Rhizoma, Scutellariae Radix, Serpentis Periostracum	Oral use	Treating corneal opacities
		Wei Gan San	Atractylodis Rhizoma, Amomi Fructus, Bupleuri Radix, Magnoliae Officinalis Cortex, Platycodonis Radix, Bigfruit Elm Fruit, Cinnamomi Cortex, Citri Reticulatae Pericarpium, Polygalae Radix, Asteris Radix Et Rhizoma, Piperis Fructus	Take on an empty stomach	Treating spleen deficiency with cold, with persistent diarrhoea
		E Jiao San	Allii Sativi Bulbus, Notopterygii Rhizoma Et Radix, Angelicae Pubescentis Radix, Rice water-Processed Atractylodis Rhizoma, Asteris Radix Et Rhizoma, Atractylodis Macrocephalae Rhizoma, Ginseng Radix Et Rhizoma, Aconiti Radix Lateralis Praeparata, Asini Corll Colla, Glycyrrhizae Radix Et Rhizoma	Take while warm	Treatment of Typhoid Fever in Pregnancy
		Da Sheng Tong Zhen Pill	Silkworm slough, Ginseng Radix Et Rhizoma, Glycyrrhizae Radix Et Rhizoma, Saposhnikoviae Radix, Angelicae Sinensis Radix, Paeoniae Radix Alba Platycodonis Radix, Gypsum Fibrosum, Angelicae Dahuricae Radix, Zingiberis Rhizoma, Aconiti Radix Lateralis	Take on an empty stomach	Promoting blood circulation and removing blood stasis

Date of Publication	References	Preparation name	Compositiona crude drug names	Traditional uses	Efficacy
			Praeparata, Chuanxiong Rhizoma, Ligustici Rhizoma Et Radix, Lycopi Herba, Bigfruit Elm Fruit, Zanthoxyli Pericarpium, Platycladi Semen, Cicadae Periostracum, Atractylodis Rhizoma, Cynanchi Atrati Radix Et Rhizoma, Atractylodis Macrocephalae Rhizoma, Magnoliae Officinalis Cortex Aucklandiae Radix Astragali Radix Achyranthis Bidentatae Radix		
Song	Su Shen Liang Fang	Hou Po San II	Magnoliae Officinalis Cortex Atractylodis Rhizoma Citri Reticulatae Pericarpium Zingiberis Rhizoma Glycyrrhizae Radix Et Rhizoma	Drink while hot	For treating wind-cold exogenous pathogens in children, relieving muscle tension
		Zhu Gan San	Asteris Radix Et Rhizoma, Platycodonis Radix, Atractylodis Rhizoma, Paeoniae Radix Alba	Take on an empty stomach before meals	Treating liver atrophy and weak legs
		Huo Xue Ying Tong Wan	Cibotii Rhizoma, Rice-water Processed Atractylodis, Rhizoma cyperi rhizoma, Citri Reticulatae Pericarpium, Myrrha, Clematidis Radix Et Rhizoma, Aconiti Kusnezoffii Radix	Take warm, regardless of time	For treating blood stasis and stagnation, and heavy, aching pain in the lower back and legs
Song	Tai Ping Hui Min He Ji Ju Fang	He Jie San	Magnoliae Officinalis Cortex, Citri Reticulatae Pericarpium, Ligustici Rhizoma Et Radix, Platycodonis Radix, Glycyrrhizae Radix Et Rhizoma	Take warm, regardless of time	Treatment for Headaches in the Four Seasons of Cold Damage
		Shi Hua San	Acanthopanacis Cortex, Citri Reticulatae Pericarpium, Zingiberis Rhizoma, Glycyrrhizae Radix Et Rhizoma, Platycodonis Radix, Notopterygii Rhizoma Et Radix, Astragali Radix, Cinnamomi Cortex, Processed Atractylodis Rhizoma, Aconiti Radix Lateralis Praeparata, Aconiti Radix	Take while warm	For the treatment of chronic fatigue and general debility, as well as widespread bodily pain

Date of Publication	References	Preparation name	Compositiona crude drug names	Traditional uses	Efficacy
		Bao Zhen Decoction	Ligustici Rhizoma Et Radix, Chuanxiong Rhizoma, Glycyrrhizae Radix Et Rhizoma, Bran-Processed Atractylodis Rhizoma	Take hot, regardless of time	Treatment of Cold Damage in All Four Seasons
		Xiao Feng Bai Jie San	Schizonepetae Herba, Angelicae Dahuricae Radix, Citri Reticulatae Pericarpium, Atractylodis Rhizoma, Ephedrae Herba, Glycyrrhizae Radix Et Rhizoma Praeparata Cum Melle	Take warm, regardless of time	For seasonal cold damage, headache, and stiff neck
		Shen Zhu San	Rice Water-Processed Atractylodis Rhizoma, Ligustici Rhizoma Et Radix, Angelicae Dahuricae Radix, Asari Radix Et Rhizoma, Notopterygii Rhizoma Et Radix, Chuanxiong Rhizoma, Glycyrrhizae Radix Et Rhizoma Praeparata Cum Melle	Take warm, regardless of time	For treating seasonal epidemics, headaches, and stiff neck
		Bu Huan Jin Zheng Qi San	Magnoliae Officinalis Cortex, Pogostemonis Herba, Glycyrrhizae Radix Et Rhizoma Praeparata Cum Melle, Pinelliae Rhizoma, Rice Water-Processed Atractylodis Rhizoma, Citri Reticulatae Pericarpium	Take warm before meals	For Treating Cold Damage in All Four Seasons
		Shen Shi Decoction	Atractylodis Rhizoma, Atractylodis Macrocephalae Rhizoma, Glycyrrhizae Radix Et Rhizoma Praeparata Cum Melle, Proia, Zingiberis Rhizoma, Citri Exocarpium Rubrum, Caryophylli Flos	Take warm before meals	For treating cold and dampness injuries, with heaviness in the body and coldness in the lower back
		Ping Wei San II	Rice Water-Processed Atractylodis Rhizoma, Magnoliae Officinalis Cortex, Citri Reticulatae Pericarpium, Processed Glycyrrhizae Radix Et Rhizoma,	Take warm before meals	Treating disharmony of the spleen and stomach
		He Qi San	Cyperi Rhizoma, Citri Reticulatae Pericarpium, Cinnamomi Cortex, Alpiniae Officinarum Rhizoma, Citri Reticulatae Pericarpium Viride,	Take orally at any time	For treating disharmony of the spleen and stomach, and qi

Date of Publication	References	Preparation name	Compositiona crude drug names	Traditional uses	Efficacy
			Glycyrrhizae Radix Et Rhizoma, Anisi Stellati Fructus, Rice Water-Processed Atractylodis Rhizoma, Platycodonis Radix		stagnation in the epigastrium
		Shou Zhong Jin Wan	Zingiberis Rhizoma, Glycyrrhizae Radix Et Rhizoma, Rice Water-Processed Atractylodis Rhizoma, Platycodonis Radix	Take warm before meals	Imbalance in the middle burner, accumulation of cold in the spleen and stomach
		Zao Rou Ping Wei San	Citri Reticulatae Pericarpium, Magnoliae Officinalis Cortex, Glycyrrhizae Radix Et Rhizoma, Zingiberis Rhizoma Recens, Jujubae Fructus, Rice Water-Processed Atractylodis Rhizoma	Take warm before meals	Treating disharmony of the spleen and stomach
		Yang Shen San	Scorpio, Gastrodiae Rhizoma, Atractylodis Rhizoma, Processed Aconiti Radix Lateralis Praeparata, Aconiti Kusnezoffii Radix	Take warm on an empty stomach	Treating pain in the lower back and leg joints
		Rou Dou Kou San	Rice Water-Processed Atractylodis Rhizoma, Anisi Stellati Fructus, Cinnamomi Cortex, Aconiti Radix, Chebulae Fructus, Zingiberis Rhizoma, Magnoliae Officinalis Cortex, Citri Reticulatae Pericarpium, Myristicae Semen, Glycyrrhizae Radix Et Rhizoma	Take while warm	For treating spleen and stomach qi deficiency with abdominal and flank distension and fullness
		Qu Zhu Pill	Medicated Leaven, Rice Water-Processed Atractylodis Rhizoma	Take at any time	For heat-induced sudden diarrhea
		Ding Xiang Dou Kou San	Sparganii Rhizoma, Aucklandiae Radix, Magnoliae Officinalis Cortex, Paeoniae Radix Alba, Myristicae Semen, Ginseng Radix Et Rhizoma, Zingiberis Rhizoma, Proia, Euodiae Fructus, Glycyrrhizae Radix Et Rhizoma Praeparata Cum Melle, Caryophylli Flos, Atractylodis Rhizoma	Take warm on an empty stomach	For treating spleen and stomach deficiency with chronic cold stagnation

Date of Publication	References	Preparation name	Compositiona crude drug names	Traditional uses	Efficacy
		Ru Shen Zhi Xie Pill	Pinelliae Rhizoma, Rice Water-Processed Atractylodis Rhizoma, Aconiti Radix	Take warm on an empty stomach	Treating visceral deficiency-cold and spleen-stomach dampness
		Sheng Ma He Qi Yin	Zingiberis Rhizoma, Aurantii Fructus, Puerariae Lobatae Radix, Atractylodis Rhizoma, Platycodonis Radix, Cimicifugae Rhizoma, Angelicae Sinensis Radix, Processed Pinelliae Rhizoma, Proia, Angelicae Dahuricae Radix, Citri Reticulatae Pericarpium, Glycyrrhizae Radix Et Rhizoma, Paeoniae Radix Alba, Rhei Radix Et Rhizoma	Take warm before meals	Treating scabies and sores that appear on the limbs
		Hou Po San III	Rice Water-Processed Atractylodis Rhizoma, Ginger-Processed Magnoliae Officinalis Cortex, Citri Reticulatae Pericarpium, Zingiberis Rhizoma, Glycyrrhizae Radix Et Rhizoma Praeparata Cum Melle	Drink while hot	Treatment for Wind-Cold Exogenous Pathogens in Children
		Xian Zhu Decoction	Atractylodis Rhizoma, Jujubae Fructus, Zingiberis Rhizoma, Armeniacae Semen Amarum, Glycyrrhizae Radix Et Rhizoma, Salt	Take before meals	Dispel cold and dampness, warm the spleen and stomach
		Xiao Li Zhong Decoction	Rice Water-Processed Atractylodis Rhizoma, Zingiberis Rhizoma Recens, Glycyrrhizae Radix Et Rhizoma, Salt	Take on an empty stomach	Treatment for chest and flank distension and fullness
Song	Sheng Ji Zong Lu	Qiang Huo Decoction	Notopterygii Rhizoma Et Radix, Cinnamomi Cortex, Rehmanniae Radix Preparata, Paeoniae Radix Alba, Puerariae Lobatae Radix Ephedrae Herba, Glycyrrhizae Radix Et Rhizoma Praeparata Cum Melle, Zingiberis Rhizoma Recens	Take while warm at any time	Treating stroke-related joint pain
		Shen Zhu Decoction	Atractylodis Rhizoma, Glycyrrhizae Radix Et	Take while warm at any time	For treating external wind-cold

Date of Publication	References	Preparation name	Compositiona crude drug names	Traditional uses	Efficacy
			Rhizoma, Ephedrae Herba, Gleditsiae Fructus Abnormalis		pathogens and joint pain
		Mo Yao Pill	Myrrha, Aconiti Kusnezoffii Radix, Schizonepetae Spica, Rice Water-Processed Atractylodis Rhizoma, Tigris Bone, Olibanum, Draconis Sanguis	Take on an empty stomach	Treating Weakness in the Lower Back and Legs
		Xian Mao Pill	Curculiginis Rhizoma, Clematidis Radix Et Rhizoma, Notopterygii Rhizoma Et Radix, Citri Reticulatae Pericarpium Viride, Pharbitidis Semen, Proia, Curcumae Longae Rhizoma, Atractylodis Macrocephalae Rhizoma, Rice Water-Processed Atractylodis Rhizoma	Oral use	Treating bodily pain and stiffness in the limbs
		Qi Sheng Decoction	Ephedrae Herba, Processed Atractylodis Rhizoma, Citri Reticulatae Pericarpium, Akebiae Caulis, Platycodonis Radix, Artemisiae Scopariae Herba, Glycyrrhizae Radix Et Rhizoma Praeparata Cum Melle	Take while warm	Treatment for headache and high fever, accompanied by body aches and discomfort
		Cang Zhu Pill I	Rice Water-Processed Atractylodis Rhizoma, Chuanxiong Rhizoma, Saposhnikoviae Radix, Schisandrae Chinensis Fructus, Astragali Radix, Angelicae Sinensis Radix, Sulfur	Take on an empty stomach	For treating weak and fine pulse, with blood loss in stools
		Gu Jing San	Eriocauli Flos, Haliotidis Concha, Equiseti Hiemalis Herba, Schizonepetae Spica, Glycyrrhizae Radix Et Rhizoma Praeparata Cum Melle, Notopterygii Rhizoma Et Radix, Inulae Flos, Chrysanthemi Flos, Lycii Fructus, Mori Folium, Serpentis Periostracum, Rice Water-Processed Atractylodis Rhizoma	Take at any time	Treating joint pain

Date of Publication	References	Preparation name	Compositiona crude drug names	Traditional uses	Efficacy
		Mu Xiang Decoction	Aucklandiae Radix, Myristicae Semen, Angelicae Sinensis Radix, Ginseng Radix Et Rhizoma, Processed Aconiti Radix Lateralis Praeparata, Zingiberis Rhizoma, Glycyrrhizae Radix Et Rhizoma Praeparata Cum Melle, Atractylodis Rhizoma, **Citri Reticulatae Pericarpium, Euodiae Fructus, Magnoliae Officinalis Cortex**	Take hot before meals	Treating spleen deficiency with cold
		Bai Zhu Pill	Atractylodis Macrocephalae Rhizoma, Magnoliae Officinalis Cortex, Atractylodis Rhizoma, Bigfruit Elm Fruit, Citri Reticulatae Pericarpium Viride, Processed Aconiti Radix Lateralis Praeparata, Glycyrrhizae Radix Et Rhizoma Praeparata Cum Melle, Zingiberis Rhizoma	Take on an empty stomach	For treating disharmony of the spleen and stomach, resulting in inability to eat or drink
		Wu Zhu Pill	Aconiti Kusnezoffii Radix, Atractylodis Rhizoma, Citri Reticulatae Pericarpium, Glycyrrhizae Radix Et Rhizoma, Sojae Semen Nigrum	Take on an empty stomach	Treating spleen and stomach deficiency
		Bu He Decoction	Ginseng Radix Et Rhizoma, Astragali Radix, Atractylodis Macrocephalae Rhizoma, Glycyrrhizae Radix Et Rhizoma Praeparata Cum Melle, Zingiberis Rhizoma, Amomi Fructus Rotundus, Rice Water-Processed Atractylodis Rhizoma, Citri Reticulatae Pericarpium	Drink while hot	Regulating Qi
		Sang Bai Pi Decoction	Mori Cortex, Atractylodis Rhizoma, Akebiae Caulis, Cinnamomi Cortex, Angelicae Sinensis Radix, Coptidis Rhizoma, Alpiniae Katsumadai Semen, Aconiti Radix, Dianthi Herba, Arecae Pericarpium, Belamcandae Rhizoma, Pharbitidis Semen, Persicae Semen, Pruni Semen	Oral use	For the treatment of a puffy face and inability to lie down comfortably

Date of Publication	References	Preparation name	Compositiona crude drug names	Traditional uses	Efficacy
		Wu Tou Decoction	Aconiti Radix, Atractylodis Rhizoma	Drink while hot	Treating Abdominal Distension and Fullness
		Cang Zhu Pill II	Atractylodis Rhizoma, Magnoliae Officinalis Cortex, Coptidis Rhizoma, Angelicae Sinensis Radix, Chebulae Fructus, Zingiberis Rhizoma, Euodiae Fructus, Artemisiae Argyi Folium, Os Draconis, Aconiti Radix Lateralis Praeparata	Oral use	Treating Diarrhea and Weakness
		Yun Qi Decoction	Rice Water-Processed Atractylodis Rhizoma, Magnoliae Officinalis Cortex, Glycyrrhizae Radix Et Rhizoma, Zingiberis Rhizoma	Take warm before meals	Treating tenesmus
		Cang Zhu Yin	Rice Water-Processed Atractylodis Rhizoma, Armeniacae Semen Amarum, Proia, Mori Cortex, Phytolacca Americana, Arecae Pericarpium, Broussonetiae Fructus	Take warm before meals	For headache and facial swelling
		Fang Ji Yin	Stephaniae Tetrandrae Radix, Proia, Mori Cortex, Notopterygii Rhizoma Et Radix, Rice Water-Processed Atractylodis Rhizoma, Pruni Semen	Take warm, regardless of time	Treating facial swelling and bone pain
		Qi Shang San	Foeniculi Fructus, Atractylodis Macrocephalae Rhizoma, Ginseng Radix Et Rhizoma, Proia, Citri Reticulatae Pericarpium, Paeoniae Radix Alba, Platycodonis Radix, Asteris Radix Et Rhizoma, Rice Water-Processed Atractylodis Rhizoma, Angelicae Dahuricae Radix, Bupleuri Radix, Zingiberis Rhizoma	Oral use	For treating spleen fatigue with abdominal distension and loose stools
		Bai Zhu San	Atractylodis Macrocephalae Rhizoma, Angelicae Dahuricae Radix, Vinegar-Processed Pelodisci Carapax Rice Water-Processed Atractylodis	Take on an empty stomach	Treating diarrhea

Date of Publication	References	Preparation name	Compositiona crude drug names	Traditional uses	Efficacy
			Rhizoma, Saposhnikoviae Radix, Magnoliae Officinalis Cortex, Cinnamomi Cortex, Ginseng Radix Et Rhizoma, Citri Reticulatae Pericarpium, Zingiberis Rhizoma, Alpiniae Officinarum Rhizoma, Euodiae Fructus, Bupleuri Radix, Zanthoxyli Pericarpium, Chuanxiong Rhizoma, Proia, Bigfruit Elm Fruit, Amomi Fructus, Aconiti-Processed Radix Lateralis Praeparata, Aquilariae Lignum Resinatum, Caryophylli Flos, Angelicae Sinensis Radix, Aucklandiae Radix		
		Zi Wan Decoction	Asteris Radix Et Rhizoma Bupleuri Radix Processed Aconiti Radix Lateralis Praeparata Rice Water-Processed Atractylodis Rhizoma Paeoniae Radix Rubra Myristicae Semen Ginseng Radix Et Rhizoma	Take warm, regardless of time	Treating consumptive cough
		Shi Hu Decoction	Dendrobii Caulis, Rice Water-Processed Atractylodis Rhizom, Platycodonis Radix, Citri Reticulatae Pericarpium, Glycyrrhizae Radix Et Rhizoma Praeparata Cum Melle, Ephedrae Herba, Drynariae Rhizoma, Cinnamomi Cortex	Drink while hot	For treating physical pain caused by deficiency-syndrome
		Zhi Gan San II	Atractylodis Rhizoma, Aucklandiae Radix, Cinnamomi Cortex, Processed Aconiti Radix Lateralis Praeparata, Proia, Ginseng Radix Et Rhizoma, Magnoliae Officinalis Cortex, Achyranthis Bidentatae Radix, Paeoniae Radix Alba, Vinegar-Processed Pelodisci Carapax, Angelicae Sinensis Radix, Citri Reticulatae Pericarpium Viride	Take on an empty stomach	For acute exhaustion and wasting disease
		Bu Zhen Pill	Magnoliae Officinalis Cortex, Rice Water-Processed	Oral use	Treating emaciation, night

Date of Publication	References	Preparation name	Compositiona crude drug names	Traditional uses	Efficacy
			Atractylodis Rhizoma, Citri Reticulatae Pericarpium, Dendrobii Caulis, Processed Aconiti Radix Lateralis Praeparata, Bupleuri Radix, Ginseng Radix Et Rhizoma, Proia, Aquilariae Lignum Resinatum, Caryophylli Flos, Vinegar-Processed Pelodisci Carapax, Cistanches Herba, Aucklandiae Radix, Morinda Officinalis Radix, Angelicae Sinensis Radix, Alpiniae Katsumadai Semen, Chebulae Fructus, Cinnamomi Cortex, Schisandrae Chinensis Fructus, Arecae Semen, Corni Fructus, Eucommiae Cortex, Psoraleae Fructus, Astragali Radix, Euodiae Fructus		sweats, and loss of appetite
		Cang Zhu Pill III	Atractylodis Rhizoma Chebulae Fructus皮 Citri Reticulatae Pericarpium Aucklandiae Radix Paeoniae Radix Alba Citri Reticulatae Pericarpium Viride, Os Draconis, Zingiberis Rhizoma Recens	Oral use	Treating bone steaming
		Jiang Zhu Pill	Rice Water-Processed Atractylodis Rhizoma, Zingiberis Rhizoma, Iris lactea, Genkwa Flos, Trogopterori Faeces, Processed Aconiti Radix	Take warm	Treating sudden hernias
		Wu Zhu Pill	Atractylodis Rhizoma, Aconiti Radix, Zanthoxyli Pericarpium, Citri Reticulatae Pericarpium Viride, Halitum	Take on an empty stomach	Nourish the organs, strengthen the sinews and bones
		Zheng Qi Pill	Toosendan Fructus, Rice Water-Processed Atractylodis Rhizoma, Foeniculi Fructus, Zanthoxyli Pericarpium, Acori Tatarinowii Rhizoma, Anemarrhenae Rhizoma, Processed Aconiti Radix Lateralis Praeparata	Take on an empty stomach	Treating deficiency-cold in the lower energiser

Date of Publication	References	Preparation name	Compositiona crude drug names	Traditional uses	Efficacy
		Ping Wei Zhu San Jia Ju Pi Fang	Magnoliae Officinalis Cortex, Rice Water-Processed Atractylodis Rhizoma, Citri Reticulatae Pericarpium, Glycyrrhizae Radix Et Rhizoma Praeparata Cum Melle	Take warm	Treating constipation
		Sheng Bing Zi	Chuanxiong Rhizoma, Cyperi Rhizoma, Ligustici Rhizoma Et Radix, Glycyrrhizae Radix Et Rhizoma Praeparata Cum Melle, Zanthoxyli Pericarpium, Rice Water-Processed Atractylodis Rhizoma, Menthae haplocalycis herba, Menthae haplocalycis herba,	Oral use	Treating chronic deficiency of the liver and kidneys
		Qing Yan San	Serpentis Periostracum Halitum, Rice Water-Processed Atractylodis Rhizoma, Equiseti Hiemalis Herba	Take warm on an empty stomach	Treatment for corneal opacities in both eyes
		Sheng Ming San	Notopterygii Rhizoma Et Radix, Halitum, Zanthoxyli Pericarpium, Arctii Fructus, Rice Water-Processed Atractylodis Rhizoma, Viticis Fructus, Equiseti Hiemalis Herba	Oral use	For treating deficiency of the liver and kidneys, resulting in dim vision
		Cang Zhu San	Atractylodis Rhizoma, Cicadae Periostracum, Equiseti Hiemalis Herba, Scutellariae Radix	Take before meals	For redness and pain in the eyes
		Xiang Xiong Pill	Chuanxiong Rhizoma, Rice Water-Processed Atractylodis Rhizoma, Lycii Fructus, Schizonepetae Spica, Cyperi Rhizoma, Asari Radix Et Rhizoma, Cicadae Periostracum, Chrysanthemi Flos, Cassiae Semen, Inulae Flos, Gypsum Fibrosum, Glycyrrhizae Radix Et Rhizoma Praeparata Cum Melle	Take warm, regardless of time	For treating wind-toxin affecting the eyes, causing redness, dryness, itching and pain
		Gan Ju San	Chrysanthemi Flos, Inulae Flos, Buddlejae Flos, Celosiae Semen, Haliotidis Concha Notopterygii Rhizoma Et Radix Equiseti Hiemalis Herba Cassiae Semen Rice Water-	Take after breakfast and dinner	Treatment for eye swelling, dryness, and pain

Date of Publication	References	Preparation name	Compositiona crude drug names	Traditional uses	Efficacy
			Processed Atractylodis Rhizoma Cicadae Periostracum Schizonepetae Spica Glycyrrhizae Radix Et Rhizoma Praeparata Cum Melle Saposhnikoviae Radix Chuanxiong Rhizoma Ginseng Radix Et Rhizoma Scutellariae Radix		
		Yin Chen Hao San	Artemisiae Scopariae Herba, Schizonepetae Spica, Notopterygii Rhizoma Et Radix, Equiseti Hiemalis Herba, Inulae Flos, Viticis Fructus, Glycyrrhizae Radix Et Rhizoma Praeparata Cum Melle, Chuanxiong Rhizoma, Rice Water-Processed Atractylodis Rhizoma, Tribuli Fructus, Haliotidis Concha, Cassiae Semen	Oral use	For treating eye wind swelling and pain
		Chan Ke San	Cicadae Periostracum Lycii Cortex, Coptidis Rhizoma, Moutan Cortex, Atractylodis Macrocephalae Rhizoma, Atractylodis Rhizoma, Chrysanthemi Flos, Gentianae Radix Et Rhizoma, Melo Semen	Take after breakfast and dinner	For treating eye wind swelling and pain
		Yuan Ling Pill	Atractylodis Rhizoma, Glycyrrhizae Radix Et Rhizoma, Schizonepetae Spica, Pharbitidis Semen, Phellodendri Chinensis Cortex	Oral use	For treating eye wind swelling and pain
		Da Xiao Guang Ming San	Rice Water-Processed Atractylodis Rhizoma, Gecko, Equiseti Hiemalis Herba	Oral use	Treatment for eye swelling, dryness, and pain
		Ju Hua San	Chrysanthemi Flos, Atractylodis Rhizoma Schizonepetae Spica Cassiae Semen Equiseti Hiemalis Herba Inulae Flos, Glycyrrhizae Radix Et Rhizoma Praeparata Cum Melle Cicadae Periostracum Serpentis Periostracum	Take before bedtime	Treating strabismus
		Cang Zhu Pill IV	Atractylodis Rhizoma, Tribuli Fructus, Equiseti Hiemalis Herba, Inulae Flos, Aurantii	Oral use	Brighten the eyes

Date of Publication	References	Preparation name	Compositiona crude drug names	Traditional uses	Efficacy
			Fructus Immaturus, Viticis Fructus, Soybean		
		Xiong Xin Pill	Atractylodis Rhizoma, Chuanxiong Rhizoma, Asari Radix Et Rhizoma, Cicadae Periostracum, Schizonepetae Spica, Chrysanthemi Flos, Prinsepiae Nux	Oral use	For treating dizziness and headaches
		Yuan Ling Dan	Atractylodis Rhizoma, Glycyrrhizae Radix Et Rhizoma, Schizonepetae Spica, Phellodendri Chinensis Cortex, Pharbitidis Semen	Take warm	Brighten the eyes
		Jue Ming Pill	Cassiae Semen, Thlaspi Herba, Glycyrrhizae Radix Et Rhizoma Praeparata Cum Melle, Asari Radix Et Rhizoma, Chuanxiong Rhizoma, Chrysanthemi Flos, Schizonepetae Spica, Equiseti Hiemalis Herba, Inulae Flos, Atractylodis Rhizoma	Oral use	Brighten the eyes
		Ling Zhu Pill	Rice Water-Processed Atractylodis Rhizoma, Proia, Zanthoxyli Pericarpium	Take warm	Brighten the eyes
		Shi Hu San	Dendrobii Caulis, Epimedii Folium, Rice Water-Processed Atractylodis Rhizoma	Take on an empty stomach	Treatment for night blindness
		Tui Yi Xian Zhu San	Atractylodis Rhizoma, Equiseti Hiemalis Herba, Glycyrrhizae Radix Et Rhizoma Praeparata Cum Melle, Cicadae Periostracum, Eriocauli Flos, Serpentis Periostracum, Scutellariae Radix	Oral use	Treatment for corneal opacity
		E Jiao Decoction	Asini Corll Colla, Allii Sativi Bulbus, Notopterygii Rhizoma Et Radix, Angelicae Pubescentis Radix, Rice Water-Processed Atractylodis Rhizoma, Asteris Radix Et Rhizoma, Atractylodis Macrocephalae Rhizoma, Ginseng Radix Et Rhizoma, Processed Aconiti Radix Lateralis Praeparata, Glycyrrhizae Radix Et Rhizoma Praeparata Cum Melle	Take warm	Pregnancy preservation

Date of Publication	References	Preparation name	Compositiona crude drug names	Traditional uses	Efficacy
		Ma Huang Decoction	Ephedrae Herba, Atractylodis Rhizoma, Atractylodis Macrocephalae Rhizoma, Citri Reticulatae Pericarpium, Glycyrrhizae Radix Et Rhizoma	Take warm, regardless of time	Treatment of pregnancy typhoid
		Yi Gong Decoction	Ephedrae Herba, Atractylodis Rhizoma, Atractylodis Macrocephalae Rhizoma, Chuanxiong Rhizoma, Glycyrrhizae Radix Et Rhizoma Praeparata Cum Melle	Take warm	Treatment of pregnancy typhoid
		Jian Pi Decoction	Magnoliae Officinalis Cortex, Atractylodis Rhizoma, Jujubae Fructus, Citri Reticulatae Pericarpium, Proia, Ginseng Radix Et Rhizoma, Glycyrrhizae Radix Et Rhizoma	Take warm	For treating diarrhea during pregnancy
		He Li Le Decoction	Chebulae Fructus, Atractylodis Rhizoma, Myristicae Semen, Halloysitum Rubrum, Zingiberis Rhizoma, Asini Corll Colla, Artemisiae Argyi Folium, Atractylodis Macrocephalae Rhizoma, Os Draconis, Citri Reticulatae Pericarpium, Glycyrrhizae Radix Et Rhizoma Praeparata Cum Melle	Oral use, regardless of the time	For treating diarrhea during pregnancy
		Yi Sheng Decoction	Atractylodis Rhizoma, Platycodonis Radix, Aurantii Fructus, Ephedrae Herba, Processed Aconiti Radix Lateralis Praeparata, Citri Reticulatae, Pericarpium Paeoniae Radix Alba Angelicae Dahuricae Radix, Chuanxiong Rhizoma, Angelicae Sinensis Radix, Glycyrrhizae Radix Et Rhizoma, Cinnamomi Cortex, Pinelliae Rhizoma, Proia, Magnoliae Officinalis Cortex, Zingiberis Rhizoma	Drink while hot, regardless of the time	Treating women with blood and qi imbalance
		Hou Po Decoction	Magnoliae Officinalis Cortex, Rehmanniae Radix, Atractylodis Rhizoma, Angelicae Sinensis Radix, Granati Pericarpium	Take warm before meals	Treating persistent postpartum diarrhea

Date of Publication	References	Preparation name	Compositiona crude drug names	Traditional uses	Efficacy
		Xiang Wu Yao Pill	Foeniculi Fructus, Linderae Radix, Dendrobii Caulis, Trigonellae Semen, Morinda Officinalis Radix, Processed Aconiti Radix, Notopterygii Rhizoma Et Radix, Toosendan Fructus, Atractylodis Rhizoma, Pheretima, Halitum, Zanthoxyli Pericarpium, Iris lactea, Vignae Semen	Take warm before meals	For treating qi deficiency and fatigue, with a sensation of fullness and distension in the flanks
		Bu Zhong Pill	Processed Aconiti Radix, Clematidis Radix Et Rhizoma, Morinda Officinalis Radix, Rice Water-Processed Atractylodis Rhizoma, Paeoniae Radix Rubra	Take warm before meals	Replenish deficiency and boost qi
		Shao Yang Dan	Atractylodis Rhizoma, Foeniculi Fructus, Aconiti Radix, Lateralis Praeparata, Cinnabar	Take warm on an empty stomach before bedtime	Replenish vitality and preserve youthful appearance
		Shi Hu Huang Qi Pill	Aquilariae Lignum Resinatum, Cistanches Herba, Schisandrae Chinensis Fructus, Astragali Radix, Aurantii Fructus, Rehmanniae Radix, Preparata Chebulae Fructus, Aucklandiae Radix, Sweet Potato, Atractylodis Rhizoma, Alismatis Rhizoma	Before meals, on an empty stomach	For the treatment of loose stools, reduce food intake
		He Jie San	Chuanxiong Rhizoma, Notopterygii Rhizoma Et Radix, Angelicae Pubescentis Radix, Magnoliae Officinalis Cortex, Atractylodis Rhizoma, Asari Radix Et Rhizoma, Glycyrrhizae Radix Et Rhizoma	Take warm, regardless of time	Dispel cold pathogens
Song	Ji Feng Pu Ji Fang	Xiang Po Bu Xu Decoction	Magnoliae Officinalis Cortex, Atractylodis Rhizoma, Anisi Stellati Fructus, Aconiti Radix, Lateralis Praeparata, Zingiberis Rhizoma, Citri Reticulatae Pericarpium, Glycyrrhizae Radix Et Rhizoma	Take warm, regardless of time	Treatment for indigestion and summer-autumn malaria
		Bu Wo San	Atractylodis Rhizoma, Chuanxiong Rhizoma, Glycyrrhizae Radix Et	Take warm, regardless of time	Treating typhoid fever

Date of Publication	References	Preparation name	Compositiona crude drug names	Traditional uses	Efficacy
			Rhizoma, Ligustici Rhizoma Et Radix		
		Xiong Xin San	Chuanxiong Rhizoma, Atractylodis Rhizoma, Glycyrrhizae Radix Et Rhizoma, Asari Radix Et Rhizoma	Oral use	For the treatment of headaches and dizziness
		Bu Zhen Pill	Magnoliae Officinalis Cortex, Atractylodis Rhizoma, Citri Reticulatae Pericarpium, Vinegar-Processed Pelodisci Carapax, Aquilariae Lignum Resinatum, Caryophylli Flos, Cistanches Herba, Aucklandiae Radix, Morinda Officinalis Radix, Angelicae Sinensis Radix, Alpiniae Katsumadai Semen, Chebulae Fructus, Cinnamomi Cortex, Schisandrae Chinensis Fructus, Arecae Semen, Corni Fructus, Eucommiae Cortex, Psoraleae Fructus, Ginseng Radix Et Rhizoma, Aconiti Radix Lateralis, Praeparata Bupleuri Radix, Proia, Aquilariae Lignum Resinatum, Astragali Radix	Oral use	Treating weakness in the limbs
		Zhi Gan San III	Atractylodis Rhizoma, Platycodonis Radix, Citri Reticulatae Pericarpium, Bupleuri Radix, Paeoniae Radix, Rubra Asteris Radix Et Rhizoma, Amomi Fructus, Chebulae Fructus, Alpiniae Officinarum Rhizoma	Taking before meals	Treatment for cough and weakness in the limbs
		Jiang Mian Pill	Atractylodis Rhizoma, Flour, Angelicae Sinensis Radix, Zingiberis Rhizoma	Taking on an empty stomach	Treating abdominal coldness due to deficiency
		Ping Wei San III	Magnoliae Officinalis Cortex, Citri Reticulatae Pericarpium, Atractylodis Rhizoma, Glycyrrhizae Radix Et Rhizoma	Taking before meals	Enhancing nutrition
		Zao Rou Pill	Atractylodis Rhizoma, Zingiberis Rhizoma Recens,	Taking on an empty stomach	Treating disharmony of the

Date of Publication	References	Preparation name	Compositiona crude drug names	Traditional uses	Efficacy
			Jujubae Fructus, Chuanxiong Rhizoma, Cinnamomi Cortex		spleen and stomach
		Zi Gui Dan	Atractylodis Rhizoma, Medicated Leaven, Alpiniae Officinarum Rhizoma, Zingiberis Rhizoma, Cinnamomi Cortex, Hordei Fructus Germinatus, Glycyrrhizae Radix Et Rhizoma	Taking on an empty stomach	Treating umbilical and abdominal pain
		Xin Zhu San	Atractylodis Rhizoma, Citri Reticulatae Pericarpium, Asari Radix Et Rhizoma, Magnoliae Officinalis Cortex, Amomi Fructus, Aconiti Radix, Lateralis Praeparata Cinnamomi Cortex, Myristicae Semen, Zingiberis Rhizoma, Caryophylli Flos, Glycyrrhizae Radix Et Rhizoma	Take while warm	Treatment of rheumatic cold-dampness and spontaneous sweating
		Shen Shi Decoction	Atractylodis Rhizoma, Citri Reticulatae, Pericarpium Asari Radix Et Rhizoma, Magnoliae Officinalis Cortex, Amomi Fructu, Aconiti Radix, Lateralis Praeparata Cinnamomi Cortex, Myristicae Semen Zingiberis Rhizoma, Caryophylli Flos, Glycyrrhizae Radix Et Rhizoma Praeparata Cum Melle	Take while warm	Treating diarrhea and dysentery
		Zhong He Pill	Alpiniae Officinarum Rhizoma, Mume Fructus, Anisi Stellati Fructus, Zingiberis Rhizoma, Medicated Leaven, Hordei Fructus Germinatus, Proia, Glycyrrhizae Radix Et Rhizoma, Atractylodis Rhizoma	Oral use	Treating disharmony of the spleen and stomach
		Jue Ming Pill	Cassiae Semen, Celosiae Semen, Atractylodis Rhizoma, Equiseti Hiemalis Herba, Chuanxiong Rhizoma, Notopterygii Rhizoma Et Radix, Saposhnikoviae Radix, Glycyrrhizae Radix Et Rhizoma, Aurantii Fructus Immaturus, Chrysanthemi Flos, Cicadae Periostracum,	Taking after meals	Treating dim vision

Date of Publication	References	Preparation name	Compositiona crude drug names	Traditional uses	Efficacy
		Xiao Shi Jun Zi Decoction	Gypsum Fibrosum, Serpentis Periostracum, Epimedii Folium, Eriocauli Flos Quisqualis Fructus, Atractylodis Rhizoma, Paeoniae Radix Alba Ginseng Radix Et Rhizoma, Proia, Citri Reticulatae Pericarpium, Bigfruit Elm Fruit	Take on an empty stomach	Treatment of dental caries
		Wei Feng Decoction	Angelicae Dahuricae Radix, Puerariae Lobatae Radix, Atractylodis Rhizoma, Angelicae Sinensis Radix, Cimicifugae Rhizoma, Glycyrrhizae Radix Et Rhizoma Praeparata Cum Melle, Bupleuri Radix, Ligustici Rhizoma Et Radix, Notopterygii Rhizoma Et Radix, Phellodendri Chinensis Cortex, Alpiniae Katsumadai Semen, Ephedrae Herba, Viticis Fructus	Take while warm	Treatment for facial swelling and pain
Song	Ren Zhai Zhi Zhi Fang Lun	Ru Sheng San	Atractylodis Rhizoma, Processed Aconiti Radix, Saposhnikoviae Radix, Processed Aconiti Kusnezoffii Radix, Asari Radix Et Rhizoma, Anemones Raddeanae Rhizoma, Gastrodiae Rhizoma, Chuanxiong Rhizoma, Angelicae Dahuricae Radix, Scorpio, Realgar, Olibanum	Take warm, regardless of time	Stopping bleeding and relieving pain
		Bu Huan Jin Zheng Qi San	Atractylodis Rhizoma, Citri Exocarpium Rubrum, Pinelliae Rhizoma Fermentata, Processed Magnoliae Officinalis Cortex, Pogostemonis Herba, Glycyrrhizae Radix Et Rhizoma	Oral use	Expelling cold pathogens
		Yang Wei Decoction	Atractylodis Rhizoma, Processed Magnoliae Officinalis Cortex, Pinelliae Rhizoma Fermentata, Ginseng Radix Et Rhizoma, Proia, Tsaoko Fructus, Pogostemonis Herba, Citri Exocarpium	Oral use	Treating wind pathogens affecting the exterior

Date of Publication	References	Preparation name	Compositiona crude drug names	Traditional uses	Efficacy
			Rubrum, Glycyrrhizae Radix Et Rhizoma		
		Sheng Liao Wu Ji San	Rice Water-Processed Atractylodis Rhizoma, Platycodonis Radix, Citri Reticulatae Pericarpium, Ephedrae Herba, Aurantii Fructus, Magnoliae Officinalis Cortex, Zingiberis Rhizoma, Angelicae Dahuricae Radix, Chuanxiong Rhizoma, Glycyrrhizae Radix Et Rhizoma Praeparata Cum Melle, Proia, Cinnamomi Cortex, Paeoniae Radix Alba, Angelicae Sinensis Radix, Processed Pinelliae Rhizoma	Drink while hot	Treating the common cold
		Qing Shu Yi Qi Decoction	Astragali Radix, Rice Water-Processed Atractylodis Rhizoma, Cimicifugae Rhizoma, Ginseng Radix Et Rhizoma, Atractylodis Macrocephalae Rhizoma, Medicated Leaven, Citri Reticulatae Pericarpium, Alismatis Rhizoma, Glycyrrhizae Radix Et Rhizoma Praeparata Cum Melle, Ophiopogonis Radix, Angelicae Sinensis Radix, Puerariae Lobatae Radix, Schisandrae Chinensis Fructus, Citri Reticulatae Pericarpium Viride	Take while warm	Treating physical weakness with spontaneous sweating
		Jia Wei Chu Shi Decoction	Atractylodis Rhizoma, Atractylodis Macrocephalae Rhizoma, Glycyrrhizae Radix Et Rhizoma Praeparata Cum Melle, Zingiberis Rhizoma, Proia, Citri Exocarpium Rubrum, Cinnamomi Cortex, Magnoliae Officinalis Cortex	Oral use	Treating slight coldness in the limbs
		Fu Ling Bai Zhu Decoction	Proia, Zingiberis Rhizoma, Glycyrrhizae Radix Et Rhizoma Praeparata Cum Melle, Atractylodis Macrocephalae	Oral use	Treating body pain caused by dampness

Date of Publication	References	Preparation name	Compositiona crude drug names	Traditional uses	Efficacy
		Sheng Fu Chu Shi Decoction	Rhizoma, Cinnamomi Cortex, Atractylodis Rhizoma Aconiti Radix Lateralis Praeparata, Atractylodis Rhizoma, Atractylodis Macrocephalae Rhizoma, Magnoliae Officinalis Cortex, Chaenomelis fructus, Glycyrrhizae Radix Et Rhizoma Praeparata Cum Melle	Take while warm	Treating bodily chills and pains
		Wu Ji San	Atractylodis Rhizoma, Atractylodis Macrocephalae Rhizoma, Glycyrrhizae Radix Et Rhizoma, Zingiberis Rhizoma, Proia, Citri Reticulatae Pericarpium, Caryophylli Flos	Oral use	Treating wind-cold exogenous pathogens
		Xie Fu San	Aconiti Radix Lateralis Praeparata, Processed Aconiti Kusnezoffii Radix, Atractylodis Rhizoma, Achyranthis Bidentatae Radix, Chuanxiong Rhizoma, Angelicae Sinensis Radix, Gastrodiae Rhizoma, Stephaniae Tetrandrae Radix,	Take before meals	Treating liver and kidney deficiency
		Si Miao San	Angelicae Dahuricae Radix, Astragali Radix, Scprpio Clematidis Radix Et Rhizoma, Saigae Tataricae Cornu, Sinapis Semen, Xanthii Fructus	Oral use	Treating gout
		Xi Yao Fang	Schizonepetae Herba, Potentillae Discoloris Herba, Saposhnikoviae Radix, Xanthii Fructus, Sophorae Flavescentis Radix, Sanguisorbae Radix, Sinomenii Caulis, Clematidis Radix Et Rhizoma, Ephedrae Herba, Atractylodis Rhizoma, Scallion, Salt	Topical application	For treating swelling and pain in the lower legs
		Jia Wei Bai Du San	Notopterygii Rhizoma Et Radix, Angelicae Pubescentis Radix, Peucedani Radix, Bupleuri Radix, Aurantii Fructus, Platycodonis Radix, Glycyrrhizae Radix Et Rhizoma, Ginseng Radix Et Rhizoma, Proia, Chuanxiong	Oral use	Treating swollen and painful ankles

Date of Publication	References	Preparation name	Compositiona crude drug names	Traditional uses	Efficacy
			Rhizoma, Rhei Radix Et Rhizoma, Atractylodis Rhizoma		
		Jian Bu Pill	Rehmanniae Radix, Angelicae Sinensis Radix, Paeoniae Radix Alba, Citri Reticulatae Pericarpium, Atractylodis Rhizoma, Achyranthis Bidentatae Radix, Euodiae Fructus, Scutellariae Radix, Cinnamomi Ramulus, Arecae Pericarpium	Oral use	Treating leg pain
		Shun Qi Mu Xiang San	Alpiniae Officinarum Rhizoma, Zingiberis Rhizoma, Anisi Stellati Fructus, Amomi Fructus, Cinnamomi Cortex, Citri Exocarpium Rubrum, Magnoliae Officinalis Cortex, Glycyrrhizae Radix Et Rhizoma, Atractylodis Rhizoma, Caryophylli Flos, Platycodonis Radix	Take before meals	For bloating and diarrhea
		Luo Si Ke Pill	Spiral Shell, Talcum, Atractylodis Rhizoma, Gardeniae Fructus, Cyperi Rhizoma, Arisaematis Rhizoma, Aurantii Fructus, Citri Reticulatae Pericarpium Viride, Aucklandiae Radix, Pinelliae Rhizoma, Amomi Fructus	Oral use	Treat phlegm accumulation
		An Tong San	Trogopterori Faeces, Corydalis Rhizoma, Atractylodis Rhizoma, Alpiniae Officinarum, Rhizoma Angelicae, Sinensis Radix	Take hot, regardless of time	Treating stomachaches
		Yan Jian San	Amomi Fructus, Glycyrrhizae Radix Et Rhizoma Praeparata Cum Melle, Proia, Tsaoko Fructus, Myristicae Semen, Chuanxiong Rhizoma, Anisi Stellati Fructus, Litseae Fructus, Hordei Fructus Germinatus, Arecae Semen, Alpiniae Officinarum Rhizoma, Aurantii Fructus, Atractylodis Rhizoma, Citri Reticulatae	Taking it warm on an empty stomach	Treating sharp pain in the chest and sides

Date of Publication	References	Preparation name	Compositiona crude drug names	Traditional uses	Efficacy
			Pericarpium, Notopterygii Rhizoma Et Radix, Magnoliae Officinalis Cortex		
		Tiao Zhong Yi Qi Decoction	Cimicifugae Rhizoma, Bupleuri Radix, Citri Reticulatae Pericarpium, Aucklandiae Radix, Glycyrrhizae Radix Et Rhizoma Praeparata Cum Melle, Ginseng Radix Et Rhizoma, Atractylodis Rhizoma, Astragali Radix, Angelicae Sinensis Radix, Paeoniae Radix Alba, Schisandrae Chinensis Fructus	Oral use	Toning the spleen and stomach
		Bu Pi Pill	Atractylodis Macrocephalae Rhizoma, Atractylodis Rhizoma, Proia, Citri Reticulatae Pericarpium, Paeoniae Radix Alba	Oral use	Toning the Spleen
		Shen Ling Ping Wei San	Magnoliae Officinalis Cortex, Citri Reticulatae Pericarpium, Atractylodis Rhizoma, Glycyrrhizae Radix Et Rhizoma, Proia, Ginseng Radix Et Rhizoma	Take warm, regardless of time	Treating disharmony of the spleen and stomach
		Shen Zhu Pill	Rice Water-Processed Atractylodis Rhizoma, Semen Sesami Nigrum, Jujubae Fructus	Take warm	Treating phlegm and fluid retention
		Zhong He Pill	Atractylodis Rhizoma, Scutellariae Radix, Pinelliae Rhizoma, Cyperi Rhizoma	Oral use	For treating damp phlegm and heat in the qi
		Qing Ge Hua Tan Pill	Coptidis Rhizoma, Scutellariae Radix, Phellodendri Chinensis Cortex, Gardeniae Fructus, Cyperi Rhizoma, Atractylodis Rhizoma	Oral use	For treating damp phlegm and heat in the qi
		Qing Tan Pill	Mume Fructus, Alumen, Scutellariae Radix, Atractylodis Rhizoma, Citri Reticulatae Pericarpium, Talcum, Citri Reticulatae Pericarpium Viride, Aurantii Fructus Immaturus, Arisaematis Rhizoma, Pinelliae Rhizoma, Medicated Leaven, Crataegi Fructus, Zingiberis Rhizoma, Cyperi Rhizoma	Oral use	Clearing phlegm-heat accumulation in the middle burner

Date of Publication	References	Preparation name	Compositiona crude drug names	Traditional uses	Efficacy
Song	Shi Zhai Bai Yi Xuan Fang	Qing Qi Hua Tan Pill	Pinelliae Rhizoma, Citri Reticulatae Pericarpium, Proia, Menthae Haplocalycis Herba, Schizonepetae Spica, Scutellariae Radix, Fructus Forsythiae, Gardeniae fructus, Platycodonis Radix, Glycyrrhizae Radix Et Rhizoma Praeparata Cum Melle, Rice Water-Processed Atractylodis Rhizoma, Vinegar-Processed Cyperi Rhizoma	Take after meals	Clearing the throat and resolving phlegm
		Huo Xiang Ban Xia San	Pogostemonis Herba, Cinnamomi Cortex, Pinelliae Rhizoma Fermentata, Citri Reticulatae Pericarpium, Atractylodis Rhizoma, Zingiberis Rhizoma, Magnoliae Officinalis Cortex, Gleditsiae Spina, Glycyrrhizae Radix Et Rhizoma	Take warm, regardless of time	Treating coughs
Song	Yu Yuan Yao Fang	Ta Qi San	Anisi Stellati Fructus, Aurantii Fructus, Proia, Ginseng Radix Et Rhizoma, Zingiberis Rhizoma, Citri Reticulatae Pericarpium, Citri Reticulatae Pericarpium Viride, Glycyrrhizae Radix Et Rhizoma Praeparata Cum Melle, Atractylodis Rhizoma, Alpiniae Officinarum Rhizoma, Caryophylli Flos	Take hot, regardless of time	Promoting smooth flow of qi and easing the chest
		Huo Xiang An Wei San	Pogostemonis Herba, Pinelliae Rhizoma, Citri Reticulatae Pericarpium, Magnoliae Officinalis Cortex, Rice Water-Processed Atractylodis Rhizoma, Glycyrrhizae Radix Et Rhizoma Praeparata Cum Melle	Take warm before meals	Treating persistent vomiting
		Yong Shou Dan	Rice Water-Processed Atractylodis Rhizoma, Rehmanniae Radix Preparata, Asparagi Radix, Proia, Polygoni Multiflori Radix, Lycii Cortex	Take warm on an empty stomach	Nourish the kidneys and enrich the essence

Date of Publication	References	Preparation name	Compositiona crude drug names	Traditional uses	Efficacy
Yuan	Rui Zhu Tang Jing Yan Fang	Er Sheng Pill	Aconiti Radix, Atractylodis Rhizoma	Take warm before meals	Treating loose and painful teeth
		Tong Guang Pill	Atractylodis Rhizoma, Scutellariae Radix, Natrii Sulfas, Glycyrrhizae Radix Et Rhizoma	Take after meals	Treatment of corneal opacity
		Yi Yi Ren Decoction	Coicis Semen, Angelicae Sinensis Radix, Paeoniae Radix Alba Ephedrae Herba, Cinnamomi Cortex, Glycyrrhizae Radix Et Rhizoma, Rice Water-Processed Atractylodis Rhizoma	Take warm before meals	Treating pain in the hands and feet
		Bu Yang Pill	Cuscutae Semen, Psoraleae Fructus, Alpiniae Oxyphyllae Fructus, Eucommiae Cortex, Dioscoreae Rhizoma, Anisi Stellati Fructus, Rice Water-Processed Atractylodis Rhizoma	Take on an empty stomach	Restoring vital energy
		Si Chao Jian Pi San	Atractylodis Rhizoma, Zingiberis Rhizoma, Aconiti Lateralis Radix Praeparata, Glycyrrhizae Radix Et Rhizoma	Take before meals	Promoting dietary habits
Ming	Qi Xiao Liang Fang	Sheng Yang Chu Shi Decoction	Atractylodis Rhizoma, Pinelliae Rhizoma, Alpiniae oxyphyllae Fructus, Alismatis Rhizoma, Notopterygii Rhizoma Et Radix, Saposhnikoviae Radix, Medicated Leaven Bupleuri Radix, Cimicifugae Rhizoma, Polyporus, Citri Reticulatae Pericarpium, Hordei Fructus Germinatus, Glycyrrhizae Radix Et Rhizoma	Take on an empty stomach	Treating spleen and stomach deficiency
		Huo Xiang An Wei San	Pogostemonis Herba, Pinelliae Rhizoma, Magnoliae Officinalis Cortex, Rice Water-Processed Atractylodis Rhizoma, Citri Reticulatae Pericarpium, Glycyrrhizae Radix Et Rhizoma Praeparata Cum Melle	Take warm, regardless of time	Treating persistent vomiting
		Ban Xia Bai Zhu Tian Ma Decoction I	Pinelliae Rhizoma, Atractylodis Macrocephalae Rhizoma, Gastrodiae Rhizoma, Proia, Citri Reticulatae Pericarpium, Atractylodis Rhizoma, Ginseng	Oral use	Treating dizziness, nausea and irritability

Date of Publication	References	Preparation name	Compositiona crude drug names	Traditional uses	Efficacy
		Xian Zhu Xiong San	Radix Et Rhizoma, Medicated Leaven, Hordei Fructus Germinatus, Astragali Radix, Alismatis Rhizoma, Zingiberis Rhizoma, Tsaoko Fructus Chuanxiong Rhizoma, Forsythiae Fructus, Scutellariae Radix, Saposhnikoviae Radix, Rhei Radix Et Rhizoma, Pogostemonis Herba, Angelicae Sinensis Radix, Paeoniae Radix Alba, Platycodonis Radix, Gypsum Fibrosum Talcum, Atractylodis Rhizoma, Glycyrrhizae Radix Et Rhizoma, Menthae Haplocalycis Herba, Amomi Fructus, Schizonepetae Herba	Take after meals	For treating wind-heat obstruction
		Cang Zhu Decoction	Atractylodis Rhizoma, Bupleuri Radix, Saposhnikoviae Radix, Phellodendri Chinensis Cortex	Take on an empty stomach before meals	Treating damp-heat causing pain in the lower back and legs
		Tiao Zhong Decoction	Citri Reticulatae Pericarpium, Amomi Fructus, Pogostemonis Herba, Glycyrrhizae Radix Et Rhizoma, Paeoniae Radix Alba, Platycodonis Radix, Pinelliae Rhizoma, Angelicae Dahuricae Radix, Aurantii Fructus, Notopterygii Rhizoma Et Radix, Chuanxiong Rhizoma, Ephedrae Herba, Cinnamomi Ramulus	Oral use	Treatment of macules
Ming	Yi Fang Kao	Xiao Feng Bai Jie San	Schizonepetae Herba, Ephedrae Herba, Citri Reticulatae Pericarpium, Rice Water-Processed Atractylodis Rhizoma, Angelicae Dahuricae Radix, Glycyrrhizae Radix Et Rhizoma	Oral use	Treatment for colds and coughs
		Yue Ju Pill	Vinegar-Processed Cyperi Rhizoma, Rice Water-Processed Atractylodis Rhizoma, Gardeniae Fructus, Medicated Leaven	Oral use	Dispelling stagnant qi and relieving depression

Date of Publication	References	Preparation name	Compositiona crude drug names	Traditional uses	Efficacy
		Jiu Wei Qiang Huo Decoction	Notopterygii Rhizoma Et Radix, Saposhnikoviae Radix, Atractylodis Rhizoma, Asari Radix Et Rhizoma, Chuanxiong Rhizoma, Angelicae Dahuricae Radix, Rehmanniae Radix, Scutellariae Radix, Glycyrrhizae Radix Et Rhizoma	Oral use	Treatment of oedema
		Er Miao San	Phellodendri Chinensis Cortex, Atractylodis Rhizoma	Oral use	Treatment for damp-heat causing pain
		Ban Xia Bai Zhu Tian Ma Decoction II	Pinelliae Rhizoma, Citri Reticulatae Pericarpium, Hordei Fructus Germinatus, Ginseng Radix Et Rhizoma, Ginseng Radix Et Rhizoma, Astragali Radix, Rice Water-Processed Atractylodis Rhizoma, Gastrodiae Rhizoma, Proia, Medicated Leaven, Alismatis Rhizoma, Phellodendri Chinensis Cortex, Zingiberis Rhizoma	Oral use	Treatment for headaches and dizziness
Ming	Ren Shu Bian Lan	Dang Gui Zhi Tong Decoction	Notopterygii Rhizoma Et Radix, Glycyrrhizae Radix Et Rhizoma Praeparata Cum Melle, Wine Processed Scutellariae Radix, Artemisiae Scopariae Herba, Ginseng Radix Et Rhizoma, Saposhnikoviae Radix, Cimicifugae Rhizoma, Sophorae Flavescentis Radix, Puerariae Lobatae Radix, Rice Water-Processed Atractylodis Rhizoma, Angelicae Sinensis Radix, Anemarrhenae Rhizoma, Proia, Alismatis Rhizoma, Polyporus, Atractylodis Macrocephalae Rhizoma	Oral use	Treating rheumatism
		Tong Qi Fang Feng Decoction	Notopterygii Rhizoma Et Radix, Angelicae Pubescentis Radix, Ligustici Rhizoma Et Radix, Saposhnikoviae Radix, Glycyrrhizae Radix Et	Oral use	Treatment for shoulder and back pain

Date of Publication	References	Preparation name	Compositiona crude drug names	Traditional uses	Efficacy
		Xiao Feng Bai Jie San	Rhizoma, Chuanxiong Rhizoma, Viticis Fructus Schizonepetae Herba, Angelicae Dahuricae Radix, Citri Reticulatae Pericarpium, Ephedrae Herba, Atractylodis Rhizoma, Ephedrae Herba, Glycyrrhizae Radix Et Rhizoma Praeparata Cum Melle	Oral use	For treating headaches and fever
		Liu Shen Tong Jie San	Ephedrae Herba, Glycyrrhizae Radix Et Rhizoma, Scutellariae Radix, Gypsum Fibrosum, Atractylodis Rhizoma, Chuanxiong Rhizoma, Notopterygii Rhizoma Et Radix, Asari Radix Et Rhizoma, Sojae Semen Praeparatum	Oral use	For headaches and fever
		Tiao Zhong Yi Qi Decoction	Cimicifugae Rhizoma, Astragali Radix, Glycyrrhizae Radix Et Rhizoma, Atractylodis Rhizoma, Aucklandiae Radix, Ginseng Radix Et Rhizoma, Bupleuri Radix, Citri Reticulatae Pericarpium	Oral use	Treating abdominal pain
		Xiang Sha He Zhong Decoction	Pogostemonis Herba, Atractylodis Rhizoma, Amomi Fructus, Magnoliae Officinalis Cortex, Citri Reticulatae Pericarpium, Proia, Pinelliae Rhizoma, Cyperi Rhizoma, Citri Reticulatae Pericarpium Viride, Glycyrrhizae Radix Et Rhizoma, Aucklandiae Radix	Oral use	Relieving pain in the heart and abdomen
		Huo Xiang An Wei Decoction	Pogostemonis Herba, Pinelliae Rhizoma, Magnoliae Officinalis Cortex, Atractylodis Rhizoma, Citri Reticulatae Pericarpium, Glycyrrhizae Radix Et Rhizoma	Oral use	Treat vomiting
		Yue Ju Pill	Atractylodis Rhizoma, Cyperi Rhizoma, Chuanxiong Rhizoma, Medicated Leaven, Gardeniae Fructus	Oral use	Treatment for depression
		Kai Yu Decoction	Cyperi Rhizoma, Fritillariae Cirrhosae Bulbus, Atractylodis Rhizoma, Chuanxiong Rhizoma, Medicated Leaven, Medicated Leaven, Gardeniae	Oral use	Treating stagnation and depression of qi

Date of Publication	References	Preparation name	Compositiona crude drug names	Traditional uses	Efficacy
			Fructus, Citri Exocarpium Rubrum, Proia, Aurantii Fructus, Perillae Fructus, Glycyrrhizae Radix Et Rhizoma, Pinelliae Rhizoma, Arisaematis Rhizoma		
		Sheng Yang Chu Shi Fang Feng Decoction	Atractylodis Rhizoma, Saposhnikoviae Radix, Atractylodis Macrocephalae Rhizoma, Proia, Paeoniae Radix Alba	Take on an empty stomach	For constipation, or tenesmus
		Han Duo Wan An San	Atractylodis Rhizoma, Magnoliae Officinalis Cortex, Citri Reticulatae Pericarpium, Arecae Semen, Dichroae Radix, Tsaoko Fructus, Glycyrrhizae Radix Et Rhizoma	Oral use	Treating malaria
Ming	Zu Ji	Cang Zhu Di Yu Decoction	Atractylodis Rhizoma, Sanguisorbae Radix	Oral use	For treating abdominal distension, dampness, and bloody diarrhoea
Qing	Hui Zhi Tang Jing Yan Fang	Zhu Dan Dao Shui Fang	Bupleuri Radix, Scutellariae Radix, Paeoniae Radix Alba, Plantaginis Semen, Indian Bread with Pine, Alismatis Rhizoma, Gardeniae Fructus, Atractylodis Rhizoma	Oral use	For treating deficiency of the liver and gallbladder
Qing	Cheng Fang Qie Yong	He Wei Yin	Citri Reticulatae Pericarpium, Magnoliae Officinalis Cortex, Zingiberis Rhizoma, Glycyrrhizae Radix Et Rhizoma Praeparata Cum Melle	Oral use	Treatment for nausea, vomiting and abdominal distension
		Qi De Pill	Psoraleae Fructus, Zingiberis Rhizoma, Atractylodis Rhizoma, Linderae Radix, Euodiae Fructus, Aucklandiae Radix, Proia	Oral use	Treatment for initial stages of diarrhoea and dysentery, with abdominal pain
Qing	Yan Fang Xin Bian	Liu Yu Decoction	Wine Processed Cyperi Rhizoma, Pinelliae Rhizoma, Proia, Citri Reticulatae Pericarpium, Chuanxiong Rhizoma, Atractylodis Rhizoma, Amomi Fructus, Gardeniae Fructus, Glycyrrhizae Radix Et Rhizoma	Oral use	Treating Back-Related Sores

Date of Publication	References	Preparation name	Compositiona crude drug names	Traditional uses	Efficacy
Qing	Yi Fang Jian Yi	Xiang Lian Ba Wu Decoction	Pogostemonis Herba, Coptidis Rhizoma, Euodiae Fructus, Indian Bread with Pine, Atractylodis Rhizoma, Magnoliae Officinalis Cortex, Plantaginis Semen	Oral use	Toning the spleen and stomach
Qing	Tai Yi Yuan Mi Cang Gao Dan Wan San Fang Ji	Jia Wei Bao He Pill	Atractylodis Macrocephalae Rhizoma, Aurantii Fructus, Immaturus Atractylodis Rhizoma, Cyperi Rhizoma, Raphani Semen, Coptidis Rhizoma Scutellariae Radix, Hordei Fructus Germinatus, Sparganii Rhizoma, Curcumae Rhizoma, Magnoliae Officinalis Cortex, Forsythiae Fructus, Citri Reticulatae Pericarpium, Pinelliae Rhizoma, Proia, Medicated Leaven, Crataegi Fructus, Aucklandiae Radix	Oral use	Treating dietary imbalances
		Ping An Dan	Atractylodis Rhizoma, Rue, Santali Albi Lignum, Aquilariae Lignum Resinatum	Oral use	Preventing epidemics
		Fa Biao Pill	Glycyrrhizae Radix Et Rhizoma, Ephedrae Herba, Cimicifugae Rhizoma, Puerariae Lobatae Radix, Atractylodis Rhizoma	Oral use	For treating typhoid fever without sweating, accompanied by headache and fever

[a]All the crude drug names in column 2 were identified in line with the Chinese Pharmacopoeia (2025) and the Latin names of the original plants were identified with WFO (www.worldfloraonline.org) and MPNS (https://mpsn.kew.org).

Table S2. Modern preparations of Atractylodis Rhizoma

NO.	Preparation name	Compositionacrude drug names	Uses	Efficacy	Page
1	Er Miao Pill	Atractylodis Rhizoma, Phellodendri Chinensis Cortex	Oral use	Disperse dampness and clear heat	471
2	Jiu Wei Qiang Huo Granule	Notopterygii Rhizoma Et Radix, Saposhnikoviae Radix, Atractylodis Rhizoma, Asari Radix Et Rhizoma, Chuanxiong Rhizoma, Angelicae Dahuricae Radix, Scutellariae Radix, Glycyrrhizae Radix Et Rhizoma, Rehmanniae Radix	Oral use	Disperse wind and release the exterior, dispel cold and eliminate dampness	516
3	Er Miao Pill	Atractylodis Rhizoma, Phellodendri Chinensis Cortex, Achyranthis Bidentatae Radix	Oral use	Clear heat and dry dampness	527
4	Xiao Er Bai Shou Pill	Uncariae Ramulus Cum Uncis, Fried Bombyx Batryticatus, Arisaema Cum Bile, Bambusae Concretio Silicea, Platycodonis Radix, Aucklandiae Radix, Amomi Fructus, Citri Reticulatae Pericarpium, Bran-Fried Atractylodis Rhizoma, Proia, Fired Crataegi Fructus, Medicated Leaven, Hordei Fructus Germinatus, Menthae haplocalycis herba, Talcum, Glycyrrhizae Radix Et Rhizoma, Cinnabaris, Bovis Calculus	Oral use	Clear heat and disperse wind, aid digestion and relieve food stagnation	570
5	Xiao Er Xiang Ju Pill	Aucklandiae Radix, Citri Reticulatae Pericarpium, Atractylodis Rhizoma, Fried Atractylodis Macrocephalae Rhizoma, Proia, Glycyrrhizae Radix Et Rhizoma, Lablab Semen Album, Bran-Fried Dioscoreae Rhizoma, Nelumbinis Semen, Bran-Fried Coicis Semen, Fried Crataegi Fructus, Hordei Fructus Germinatus, Medicated Leaven, Ginger-Processed Magnoliae Officinalis Cortex, Bran-Fried Aurantii Fructus, Immaturus, Vinegar-Processed Cyperi Rhizoma, Amomi Fructus, Pinelliae Rhizoma Praeparatum, Alismatis Rhizoma	Oral use	Strengthen the spleen and harmonize the stomach, aid digestion and stop diarrhea	585

NO.	Preparation name	Compositionacrude drug names	Uses	Efficacy	Page
6	Mu Xiang Shun Qi Pill	Aucklandiae Radix, Amomi Fructus, Vinegar-Processed Cyperi Rhizoma, Arecae Semen, Glycyrrhizae Radix Et Rhizoma, Citri Reticulatae Pericarpium, Magnoliae Officinalis Cortex, Aurantii Fructus, Atractylodis Rhizoma, Citri Reticulatae Pericarpium Viride, Zingiberis Rhizoma Recens	Oral use	Promote qi circulation and dispel dampness, strengthen the spleen and harmonize the stomach	654
7	Wu Shi Cha capsule	Atractylodis Rhizoma, Bupleuri Radix, Notopterygii Rhizoma Et Radix, Saposhnikoviae Radix, Angelicae Dahuricae Radix, Chuanxiong Rhizoma, Pogostemonis Herba, Peucedani Radix, Forsythiae Fructus, Citri Reticulatae Pericarpium, Crataegi Fructus, Aurantii Fructus Immaturus, Hordei Fructus Germinatus, Glycyrrhizae Radix Et Rhizoma, Platycodonis Radix, Perillae Fructus叶, Magnoliae Officinalis Cortex, Black Tea, Medicated Leaven	Oral use	Dispel wind and release the exterior, transform dampness and harmonize the middle burner	686
8	Feng Shi Ma Qian Tablet	Semen Strychni Pulveratum, Fired Bombyx Batryticatus, Olibanum Myrrha, Scorpion, Achyranthis Bidentatae Radix, Atractylodis Rhizoma, Ephedrae Herba, Glycyrrhizae Radix Et Rhizoma	Oral use	Dispel wind and dampness, invigorate blood circulation and remove blood stasis	738
9	Wu She Zhi Yang Pill	Zaocys, Saposhnikoviae Radix, Cnidii Fructus, Phellodendri Amurensis Cortex, Atractylodis Rhizoma, Ginseng Radix Et Rhizoma Rubra, Moutan Cortex, Snake Bile, Sophorae Flavescentis Radix, Bovis Calculus Artifactus, Angelicae Sinensis Radix	Oral use	Nourish the blood and dispel wind, dry dampness and relieve itching	753
10	Zheng Qi Tablet	Patchouli Oil, Perillae Folium, Aucklandiae Radix, Atractylodis Rhizoma, Glycyrrhizae Radix Et Rhizoma, Proia, Citri Reticulatae Pericarpium, Processed Pinelliae Rhizoma, Ginger-Processed Magnoliae Officinalis Cortex, Zingiberis Rhizoma Recens	Oral use	Disperse wind-cold, transform dampness, and harmonize the middle burner.	805

NO.	Preparation name	Compositionacrude drug names	Uses	Efficacy	Page
11	Si Miao Pill	Atractylodis Rhizoma, Achyranthis Bidentatae Radix, Salt-Processed Phellodendri Chinensis, Cortex Coicis Semen	Oral use	Clear heat and promote diuresis	843
12	Bai Shi Pill	Arnebiae Radix, Ganoderma, Dalbergiae Odoriferae Lignum, Salt-Processed Psoraleae Fructus, Salviae Miltiorrhizae Radix Et Rhizoma, Processed Polygoni Multiflori Radix, Carthami Flos, Sepiae Endoconcha, Moutan Cortex, Rhizoma Dioscoreae Bulbiferae, Atractylodis Rhizoma, Glycyrrhizae Radix Et Rhizoma, Tribuli Fructus, Gentianae Radix Et Rhizoma	Oral use	Nourish the liver and kidneys, invigorate blood circulation and remove blood stasis	860
13	Feng Liao Xing Feng Shi Die Da Yao Jiu	Erycibes Caulis, Cinnamomi Ramulus, Ephedrae Herba, Notopterygii Rhizoma Et Radix, Angelicae Sinensis Radix, Chuanxiong Rhizoma, Angelicae Dahuricae Radix, Psoraleae Fructus, Olibanum, Gleditsiae Fructus Abnormalis, Citri Reticulatae Pericarpium, Atractylodis Rhizoma, Magnoliae Officinalis Cortex, Cyperi Rhizoma, Aucklandiae Radix, Aurantii Fructus, Atractylodis Macrocephalae Rhizoma, Dioscoreae Rhizoma, Polygonati Rhizoma, Cuscutae Semen, Foeniculi Fructus, Armeniacae Semen, Amarum Alismatis Rhizoma, Trogopterori Faeces, Silkworm Excrement, Moutan Cortex Myrrha	Topical application	Dispel wind and dampness, invigorate blood circulation and relieve pain	872
14	Ban Xia Tian Ma Pill	Pinelliae Rhizoma, Praeparatum Gastrodiae Rhizoma, Astragali Radix Praeparata Cum Melle, Ginseng Radix Et Rhizoma, Atractylodis Rhizoma, Fired Atractylodis Macrocephalae Rhizoma, Proia, Citri Reticulatae Pericarpium, Alismatis Rhizoma, Medicated Leaven, Hordei	Oral use	Strengthen the spleen and dispel dampness, resolve phlegm and subdue wind	875

NO.	Preparation name	Compositionacrude drug names	Uses	Efficacy	Page
15	Dang Gui Nian Tong Pill	Fructus Germinatus, Phellodendri Chinensis Cortex Angelicae Sinensis Radix, Puerariae Thomsonii Radix, Codonopsis Radix, Atractylodis Rhizoma, Cimicifugae Rhizoma, Sophorae Flavescentis Radix, Alismatis Rhizoma, Fired Atractylodis Macrocephalae Rhizoma, Anemarrhenae Rhizoma, Saposhnikoviae Radix, Notopterygii Rhizoma Et Radix, Scutellariae Radix, Polyporus, Artemisiae Scopariae Herba, Glycyrrhizae Radix Et Rhizoma	Oral use	Clear heat and drain dampness, dispel wind and relieve pain	910
16	Ru Yi Jin Huang San	Curcumae Longae Rhizoma, Rhei Radix Et Rhizoma, Phellodendri Chinensis Cortex, Atractylodis Rhizoma, Magnoliae Officinalis Cortex, Citri Reticulatae Pericarpium, Glycyrrhizae Radix Et Rhizoma, Arisaematis Rhizoma, Angelicae Dahuricae Radix, Trichosanthis Radix	Oral use	Clear heat and detoxify, reduce swelling and relieve pain	969
17	Ke Li Sha capsule	Angelicae Dahuricae Radix, Atractylodis Rhizoma, Acori Tatarinowii Rhizoma, Asari Radix Et Rhizoma, Piperis Longi Fructus, Centipeda Herba, Gleditsiae Fructus Abnormalis, Realgar, Caryophylli Flos, Natrii Sulfas, Alumen, Borneolum	Oral use	Detoxify and purify impurities, regulate qi and stop diarrhea	1021
18	Li Ge Pill	Fired Raphani Semen, Arecae Semen, Wine-Processed Rhei Radix Et Rhizoma, Ginger-Processed Magnoliae Officinalis Cortex, Crataegi Fructus, Medicated Leaven, Amomi Fructus, Platycodonis Radix, Vinegar-Processed Citri Reticulatae Pericarpium Viride, Bran-Fried Aurantii Fructus, Hordei Fructus Germinatus, Aucklandiae Radix, Citri Reticulatae Pericarpium, Bran-Fried Atractylodis Rhizoma, Pogostemonis Herba, Tsaoko	Oral use	Relieves chest congestion and diaphragmatic discomfort, eliminates food stagnation and alleviates pain	1074

NO.	Preparation name	Compositionacrude drug names	Uses	Efficacy	Page
		Fructus, Glycyrrhizae Radix Et Rhizoma			
19	Chun Yang Zheng Qi Pill	Pogostemonis Herba, Pinelliae Rhizoma Praeparatum Cum Zingibere Et Alumine, Aucklandiae Radix, Citri Reticulatae Pericarpium, Caryophylli Flos, Cinnamomi Cortex, Atractylodis Rhizoma, Atractylodis Macrocephalae Rhizoma, Proia, Cinnabaris, Natrii Sulfas, Borax, Realgar, Micae Lapis Aureus, Moschus, Borneolum	Oral use	Warm the middle and dispel cold	1125
20	Miao Ji Pill	Black Fungus, Angelicae Sinensis Radix, Wine-Processed Paeoniae Radix Alba, Chuanxiong Rhizoma, Chaenomelis Fructus, Salt-processed Eucommiae Cortex, Dipsaci Radix, Cyathulae Radix, Atractylodis Rhizoma, Salt-processed Foeniculi Fructus, Aucklandiae Radix, Caryophylli Flos, Caryophylli Fructus, Olibanum, Proia, Smilacis Glabrae Rhizoma, Testudinis Carapax Et Plastrum	Oral use	Nourish the liver and kidneys, dispel dampness and unblock meridians	1125
21	Shen Yan Xiao Zhong Tablet	Cinnamomi Ramulus, Alismatis Rhizoma, Citri Reticulatae Pericarpium, Periplocae Cortex, Atractylodis Rhizoma, Proia, Cortex Zingiberis Recens, Arecae Pericarpium, Phellodendri Amurensis Cortex, Zanthoxyli Pericarpium, Benincasae Exocarpium, Leonuri Herba	Oral use	Strengthen the spleen and drain dampness, promote yang and facilitate water metabolism	1154
22	Shen Yan Shu Tablet	Atractylodis Rhizoma, Proia, Imperatae Rhizoma, Stephaniae Tetrandrae Radix, Ginseng Radix Et Rhizoma, Polygonati Rhizoma, Cuscutae Semen, Lycii Fructus, Lonicerae Japonicae Flos, Taraxaci Herba	Oral use	Nourishes the kidneys and strengthens the spleen, promotes diuresis and reduces swelling	1155
23	Guo Gong Jiu	Angelicae Sinensis Radix, Notopterygii Rhizoma Et Radix, Achyranthis Bidentatae Radix, Saposhnikoviae Radix, Angelicae	Topical application	Disperse wind and dampness, relax tendons and activate meridians	1166

NO.	Preparation name	Compositionacrude drug names	Uses	Efficacy	Page
24	He Zhong Li Pi Pillu Yi Jin Huang San	Pubescentis Radix, Moutan Cortex, Pogostemonis Herba, Arecae Semen, Ophiopogonis Radix, Citri Reticulatae Pericarpium, Acanthopanacis Cortex, Ginger-Processed Magnoliae Officinalis Cortex, Carthami Flos, Arisaematis Rhizoma, Preparatum Lycii Fructus, Angelicae Dahuricae Radix, Paeoniae Radix Alba, Arnebiae Radix, Salt-Processed Psoraleae Fructus, Vinegar-Processed Citri Reticulatae Pericarpium Viride, Fired Atractylodis Macrocephalae Rhizoma, Chuanxiong Rhizoma, Chaenomelis Fructus, Gardeniae Fructus, Bran-Fried Atractylodis Rhizoma, Bran-Fried Aurantii Fructus, Linderae Radix, Citri Sarcodactylis Fructus, Polygonati Odorati Rhizoma, Ultivarietas Oryzae Sativae Et Monasci Codonopsis Radix, Bran-Fried Atractylodis Macrocephalae Rhizoma Atractylodis Rhizoma, Proia, Glycyrrhizae Radix Et Rhizoma, Citri Reticulatae Pericarpium, Pinelliae Rhizoma, Praeparatum Aucklandiae Radix, Amomi Fructus, Bran-Fried Aurantii Fructus, Ginger-Processed Magnoliae Officinalis Cortex, Amomi Fructus Rotundus, Vinegar-Processed Cyperi Rhizoma, Pogostemonis Herba, Crataegi Fructus, Medicated Leaven, Hordei Fructus Germinatus, Fired Raphani Semen	Oral use	Strengthen the spleen and harmonize the stomach, regulate qi and transform dampness	1180
25	Gou Pi Gao	Aconiti Radix, Aconiti Kusnezoffii Radix, Notopterygii Rhizoma Et Radix, Angelicae Pubescentis Radix, Sinomenii Caulis, Periplocae Cortex, Saposhnikoviae Radix, Clematidis Radix Et Rhizoma, Atractylodis Rhizoma,	Topical application	Dispels wind and cold, promotes blood circulation and relieves pain	1225

NO.	Preparation name	Compositionacrude drug names	Uses	Efficacy	Page
		Cnidii Fructus, Ephedrae Herba, Alpiniae Officinarum Rhizoma, Foeniculi Fructus, Cinnamomi Cortex, Angelicae Sinensis Radix, Paeoniae Radix Rubra, Chaenomelis Fructus, Sappan Lignum, Rhei Radix Et Rhizoma, Pini Lignum Nodi, Dipsaci Radix, Chuanxiong Rhizoma, Angelicae Dahuricae Radix, Olibanum Myrrha, Borneolum, Camphor, Caryophylli Flos, Cinnamomi Cortex			
26	Jing Wan Hong Ruan Gao	Sanguisorbae Radix, Rehmanniae Radix, Angelicae Sinensis Radix, Persicae Semen, Coptidis Rhizoma, Momordicae Semen, Papaveris Pericarpium, Crinis Carbonisatus, Trachycarpi Petiolus, Lobeliae Chinensis Herba, Eupolyphaga Steleophaga, Ampelopsis Radix, Phellodendri Chinensis Cortex, Arnebiae Radix, Lonicerae Japonicae Flos, Carthami Flos, Rhei Radix Et Rhizoma, Sophorae Flavescentis Radix, Galla Chinensis, Sophorae Flos, Chaenomelis Fructus, Atractylodis Rhizoma, Angelicae Dahuricae Radix, Paeoniae Radix Rubra, Scutellariae Radix, Picrorhizae Rhizoma, Chuanxiong Rhizoma, Gardeniae Fructus, Mume Fructus, Borneolum, Draconis Sanguis, Olibanum Myrrha,	Topical application	Promotes blood circulation and detoxification, reduces swelling and relieves pain	1225
27	Xie Li Xiao capsule	Wine-Processed Coptidis Rhizoma, Atractylodis Rhizoma, Wine-Processed Paeoniae Radix Alba, Aucklandiae Radix, Euodiae Fructus, Ginger-Processed Magnoliae Officinalis Cortex, Arecae Semen, Aurantii Fructus, Citri Reticulatae Pericarpium, Alismatis Rhizoma, Proia, Glycyrrhizae Radix Et Rhizoma	Oral use	Clear heat and dry dampness, promote qi circulation and relieve pain	1234

NO.	Preparation name	Compositionacrude drug names	Uses	Efficacy	Page
28	Wei Li Kang Tablet	Pogostemonis Herba, Hordei Fructus Germinatus, Proia, Medicated Leaven, Atractylodis Rhizoma, Ginger-Processed Magnoliae Officinalis Cortex, Atractylodis Macrocephalae Rhizoma, Aucklandiae Radix, Alismatis Rhizoma, Polyporus, Citri Reticulatae Pericarpium, Pinelliae Rhizoma Praeparatum Cum Alumine, Amomi Fructus Rotundus, Glycyrrhizae Radix Et Rhizoma, Ginseng Radix Et Rhizoma, Processed Euodiae Fructus	Oral use	Promotes digestion and harmonizes the middle burner; regulates qi and transforms stagnation	1291
29	Xiang Sha Ping Wei Pill	Atractylodis Rhizoma, Citri Reticulatae Pericarpium, Ginger-Processed Magnoliae Officinalis Cortex, Aucklandiae Radix, Amomi Fructus, Glycyrrhizae Radix Et Rhizoma	Oral use	Stimulates digestion, relieves gas, and alleviates pain	1325
30	Xiang Sha He Zhong Pill	Citri Reticulatae Pericarpium, Ginger-Processed Magnoliae Officinalis Cortex, Atractylodis Rhizoma, Bran-Fried Aurantii Fructus, Vinegar-Processed Citri Reticulatae Pericarpium Viride, Crataegi Fructus, Amomi Fructus, Glycyrrhizae Radix Et Rhizoma Praeparata Cum Melle, Pogostemonis Herba, Pinelliae Rhizoma Praeparatum Cum Alumine, Atractylodis Macrocephalae Rhizoma, Proia, Medicated Leaven	Oral use	Strengthen the spleen and dry dampness, harmonize the middle burner and aid digestion	1326
31	Xiang Sha He Zhong Pill	Aucklandiae Radix , Amomi Fructus, Bran-Fried Atractylodis Rhizoma, Ginger-Processed Magnoliae Officinalis Cortex, Bran-Fried Atractylodis Macrocephalae Rhizoma, Citri Reticulatae Pericarpium, Proia, Alismatis Rhizoma, Polyporus, Cinnamomi Cortex, Glycyrrhizae Radix Et Rhizoma	Oral use	Dispel dampness and regulate the spleen, promote qi circulation and harmonize the stomach	1327

NO.	Preparation name	Compositionacrude drug names	Uses	Efficacy	Page
32	Fu Fang Zhen Zhu Kou Chuang Granule	Margarita, Galla Chinensis, Atractylodis Rhizoma, Glycyrrhizae Radix Et Rhizoma	Oral use	Dries dampness, promotes tissue regeneration, and relieves pain	1362
33	Fu Fang Xia Tian Wu Tablet	Corydalis Decumbentis Rhizoma, Coryadlis ambailis migo total alkaloids, Aconiti Kusnezoffii Radix, Moschus Olibanum, Agkistrodon, Angelicae Pubescentis Radix, Siegesbeckiae Herba, Sargentodoxae Caulis, Clematidis Radix Et Rhizoma, Salviae Miltiorrhizae Radix Et Rhizoma, Sinomenii Caulis, Spatholobi Caulis, Crataegi Folium, Achyranthis Bidentatae Radix, Angelicae Sinensis Radix, Stephaniae Tetrandrae Radix, Atractylodis Rhizoma, Acanthopanacis Cortex, Chuanxiong Rhizoma, Myrrha, Gentianae Macrophyllae Radix, Notopterygii Rhizoma Et Radix, Aucklandiae Radix, Paeoniae Radix Rubra, Saposhnikoviae Radix, Drynariae Rhizoma, Processed Strychni Semen, Bombyx Batryticatus, Scorpion, Ephedrae Herba Notoginseng Radix Et Rhizoma, Borneolum	Oral use	Dispel wind and dampness, relax tendons and activate meridians	1366
34	Fu Fang Xiao Shi Cha	Atractylodis Rhizoma, Atractylodis Macrocephalae Rhizoma, Medicated Leaven, Tea, Crataegi Fructus, Coicis Semen, Desmodium Caudatum	Oral use	Strengthen the spleen and promote diuresis, stimulate appetite and relieve indigestion	1371
35	Bao Ji Kou Fu Ye	Uncariae Ramulus Cum Uncis, Chrysanthemi Flos, Tribuli Fructus, Magnoliae Officinalis Cortex, Aucklandiae Radix, Atractylodis Rhizoma, Trichosanthis Radix, Pogostemonis Herba, Puerariae Lobatae Radix, Citri Exocarpium Rubrum, Angelicae Dahuricae Radix, Coicis Semen, Oryzae	Oral use	Resolve exterior syndromes, eliminate dampness, harmonize the middle burner	1388

NO.	Preparation name	Compositionacrude drug names	Uses	Efficacy	Page
		Fructus Germinatus, Menthae haplocalycis herba, Proia, Medicated Leaven			
36	Mai Luo Shu Tong Granule	Astragali Radix, Lonicerae Japonicae Flos, Phellodendri Chinensis Cortex, Atractylodis Rhizoma, Coicis Semen, Scrophulariae Radix, Angelicae Sinensis Radix, Paeoniae Radix Alba, Glycyrrhizae Radix Et Rhizoma, Hirudo, Scolopendra, Scorpion	Oral use	Clear heat and detoxify, resolve stasis and unblock meridians	1399
37	Qian Lie Shu Pill	Rehmanniae Radix, Praeparata Coicis Semen, Chinese Waxgourd Seed, Corni Fructus, Dioscoreae Rhizoma, Moutan Cortex, Atractylodis Rhizoma, Persicae Semen, Alismatis Rhizoma, Proia, Cinnamomi Ramulus, Aconiti Radix Lateralis Praeparata, Allii Tuberosi Semen, Epimedii Folium, Glycyrrhizae Radix Et Rhizoma	Oral use	Strengthen the body's foundation, nourish the kidneys, and promote urination	1426
38	Huo Xue Zhi Tong Gao	Zingiberis Rhizoma, Kaempferia Rhizoma, Angelicae Dahuricae Radix, Nardostachyos Radix Et Rhizoma, Rhei Radix Et Rhizoma, Arisaematis Rhizoma, Pinelliae Rhizoma, Myrrha, Olibanum, Borneolum, L-Menthol, Camphor, Citri Reticulatae Pericarpium, Angelicae Sinensis Radix, Caryophylli Flos, Piperis Fructus, Periplocae Cortex, Asari Radix Et Rhizoma, Schizonepetae Herba, Cinnamomi Ramulus, Magnoliae Flos, Chuanxiong Rhizoma, Angelicae Pubescentis Radix, Moutan Cortex, Capsici Fructus, Atractylodis Rhizoma, Belladonna Liquid Extract, Methyl Salicylate	Topical application	Promotes blood circulation and relieves pain, relaxes tendons and unblocks meridians	1432
39	Jin Li Da Granule	Ginseng Radix Et Rhizoma, Polygonati Rhizoma, Bran-Fried Atractylodis Rhizoma, Sophorae Flavescentis Radix, Ophiopogonis Radix, Rehmanniae Radix, Polygoni Multiflori Radix Praeparata, Corni Fructus, Proia,	Oral use	Tonify qi and nourish yin, strengthen the spleen and promote fluid metabolism	1439

NO.	Preparation name	Compositionacrude drug names	Uses	Efficacy	Page
40	Qu Feng Shu Jin Pill	Eupatorii Herba, Coptidis Rhizoma Anemarrhenae Rhizoma, Frying Epimedii Folium, Salviae Miltiorrhizae Radix Et Rhizoma, Puerariae Thomsonii Radix, Litchi Semen, Lycii Cortex Saposhnikoviae Radix, Cinnamomi Ramulus, Ephedrae Herba, Clematidis Radix Et Rhizoma, Processed Aconiti Radix, Processed Aconiti Kusnezoffii Radix, Bran-Fried Atractylodis Rhizoma, Proia, Chaenomelis Fructus, Gentianae Macrophyllae Radix, Drynariae Rhizoma, Achyranthis Bidentatae Radix, Glycyrrhizae Radix Et Rhizoma, Piperis Kadsurae Caulis, Sinomenii Caulis, Dioscoreae Nipponicae Rhizoma, Erodii Herba, Geranii Herba, Root of Eggplant	Oral use	Dispel wind and disperse cold, eliminate dampness and activate the meridians	1458
41	Chu Shi Bai Dai Pill	Codonopsis Radix, Fired Atractylodis Macrocephalae Rhizoma, Dioscoreae Rhizoma, Paeoniae Radix Alba, Euryales Semen, Plantaginis Semen, Angelicae Sinensis Radix, Atractylodis Rhizoma, Citri Reticulatae Pericarpium, Ginkgo Semen, Schizonepetae Herba Carbonisata, Bupleuri Radix, Phellodendri Chinensis Cortex Carbonisata, Rubiae Radix Et Rhizoma, Sepiae Endoconcha, Crassostreae Concha	Oral use	Strengthen the spleen and boost qi, eliminate dampness and stop vaginal discharge	1462
42	Jian Wei Tablet	Crataegi Fructus, Medicated Leaven, Hordei Fructus Germinatus, Arecae Semen, Galli Gigerii Endothelium Corneum, Atractylodis Rhizoma, Alpiniae Katsumadai Semen, Citri Reticulatae Pericarpium, Zingiberis Rhizoma Recens, Bupleuri Radix, Paeoniae Radix Alba, Toosendan Fructus,	Oral use	Soothe the liver and harmonize the stomach, aid digestion and relieve food stagnation	1512

NO.	Preparation name	Compositionacrude drug names	Uses	Efficacy	Page
43	Tiao Wei Xiao Zhi Pill	Vinegar-Processed Corydalis Rhizoma, Licorice Extract Ginger-Processed Magnoliae Officinalis Cortex, Notopterygii Rhizoma Et Radix, Medicated Leaven, Aurantii Fructus, Cyperi Rhizoma, Pinelliae Rhizoma Praeparatum Cum Zingibere Et Alumine, Saposhnikoviae Radix, Peucedani Radix, Chuanxiong Rhizoma, Angelicae Dahuricae Radix, Menthae haplocalycis herba, Amomi Fructus, Tsaoko Fructus Aucklandiae Radix, Amomi Fructus Rotundus, Proia, Atractylodis Rhizoma, Pogostemonis Herba, Linderae Radix, Glycyrrhizae Radix Et Rhizoma, Perillae Folium, Citri Reticulatae Pericarpium	Oral use	Disperse wind and release the exterior, dispel cold and transform dampness	1602
44	Qing Shu Yi Qi Pill	Ginseng Radix Et Rhizoma, Astragali Radix, Fired Atractylodis Macrocephalae Rhizoma, Atractylodis Rhizoma, Ophiopogonis Radix, Alismatis Rhizoma, Vinegar-Processed Schisandrae Chinensis Fructus, Angelicae Sinensis Radix, Phellodendri Chinensis Cortex, Puerariae Lobatae Radix, Vinegar-Processed Citri Reticulatae Pericarpium Viride, Citri Reticulatae Pericarpium, Medicated Leaven, Cimicifugae Rhizoma, Glycyrrhizae Radix Et Rhizoma	Oral use	Dispels summer heat and promotes diuresis, Tonifies qi and generates fluids	1730
45	Jing Fu Kang Granule	Notopterygii Rhizoma Et Radix, Chuanxiong Rhizoma, Puerariae Lobatae Radix, Gentianae Macrophyllae Radix, Clematidis Radix Et Rhizoma, Bran-Fried Atractylodis Rhizoma, Salviae Miltiorrhizae Radix Et Rhizoma, Paeoniae Radix Alba, Wine-Processed Pheretima, Carthami Flos, Processed Olibanum, Astragali Radix, Codonopsis	Oral use	Promote blood circulation and unblock meridians; dispel wind and relieve pain	1736

NO.	Preparation name	Compositionacrude drug names	Uses	Efficacy	Page
		Radix, Rehmanniae Radix, Haliotidis Concha, Ophicalcitum, Phellodendri Amurensis Cortex, Fired Vaccariae Semen, Persicae Semen, Processed Myrrha, Wine-Processed Eupolyphaga Steleophaga			
46	Yue Ju Er Chen Pill	Vinegar-Processed Cyperi Rhizoma, Bran-Fried Atractylodis Rhizoma, Chuanxiong Rhizoma, Pinelliae Rhizoma, Praeparatum Cum Alumine, Hordei Fructus Germinatus, Fired Medicated Leaven Proia, Fired Gardeniae Fructus, Citri Reticulatae Pericarpium, Glycyrrhizae Radix Et Rhizoma	Oral use	Regulate qi and relieve depression, transform phlegm and harmonize the middle burner	1745
47	Yue Ju Pill	Vinegar-Processed Cyperi Rhizoma, Chuanxiong Rhizoma, Fired Gardeniae Fructus, Fired Atractylodis Rhizoma, Fired Medicated Leaven	Oral use	Regulate qi and relieve depression, ease the middle and eliminate fullness	1746
48	Yue Ju Bao He Pill	Ginger-Processed Gardeniae Fructus, Bran-Fried Medicated Leaven, Vinegar-Processed Cyperi Rhizoma, Chuanxiong Rhizoma, Atractylodis Rhizoma, Aucklandiae Radix, Arecae Semen	Oral use	Soothe the liver and relieve depression, stimulate appetite and aid digestion	1747
49	Shu Gan Ping Wei Pill	Ginger-Processed Magnoliae Officinalis Cortex, Citri Reticulatae Pericarpium, Bran-Fried Aurantii Fructus, Pinelliae Rhizoma, Praeparatum Atractylodis Rhizoma, Glycyrrhizae Radix Et Rhizoma Praeparata Cum Melle, Arecae Semen	Oral use	Soothe the liver and harmonize the stomach, transform dampness and resolve stagnation	1777
50	Sha Yao	Caryophylli Flos, Atractylodis Rhizoma, Gastrodiae Rhizoma, Ephedrae Herba, Rhei Radix Et Rhizoma, Glycyrrhizae Radix Et Rhizoma, Borneolum, Moschus, Processed Bufonis Venenum, Realgar Cinnabaris	Oral use	Dispel summer heat and detoxify, purify impurities and clear the senses	1797
51	Shi Re Bi Tablet	Atractylodis Rhizoma, Lonicerae Japonicae Caulis, Pheretima, Forsythiae Fructus, Phellodendri	Oral use	Dispel wind and dampness, clear	1805

NO.	Preparation name	Compositionacrude drug names	Uses	Efficacy	Page
		Chinensis Cortex, Coicis Semen, Saposhnikoviae Radix, Clematidis Radix Et Rhizoma, Stephaniae Tetrandrae Radix, Cyathulae Radix, Dioscoreae Hypoglaucae Rhizoma, Mori Ramulus		heat and reduce swelling	
52	Yao Tong Ning capsule	Semen Strychni Pulveratum, Eupolyphaga Steleophaga, Cyathulae Radix, Glycyrrhizae Radix Et Rhizoma, Ephedrae Herba, Olibanum, Myrrha, Scorpion, Bombyx Batryticatus, Bran-Fried Atractylodis Rhizoma	Oral use	Reduce swelling and relieve pain, disperse cold pathogens	1845
53	Tan Yin Pill	Cinnamomi Cortex, Radix Aconiti Carmichaeli Preparata, Atractylodis Rhizoma, Bran-Fried Atractylodis Macrocephalae Rhizoma, Fired Perillae Fructus, Fired Raphani Semen, Zingiberis Rhizoma, Fired Sinapis Semen, Glycyrrhizae Radix Et Rhizoma, Praeparata Cum Melle	Oral use	Warmly tonify the spleen and kidneys, assist yang to transform fluid retention	1854
54	Huo Xiang Zheng Qi Shui	Atractylodis Rhizoma, Citri Reticulatae Pericarpium, Ginger-Processed Magnoliae Officinalis Cortex, Angelicae Dahuricae Radix, Proia, Arecae Pericarpium, Pinelliae Rhizoma, Licorice Extract, Patchouli Oil, Perillae Folium Oil	Oral use	Resolve exterior pathogens and transform dampness, regulate qi and harmonize the middle burner	1924

[a]All the crude drug names in column 2 were identified in line with the Chinese Pharmacopoeia (2025) and the Latin names of the original plants were identified with WFO (www.worldfloraonline.org) and MPNS (https://mpsn.kew.org).

Table S3. Antifungal, antibacterial, and antiviral effect of Atractylodis Rhizoma

Bioactivity	Compound/Extract	Test Subject (in vitro)	Dose (MIC/MBC etc.)	Positive Control	Key Results / Proposed Mechanism	References
Antibacterial effect	Extract containing α-Pinene (1)	*Staphylococcus aureus*,*Bacillus subtilis*,*Bacillus cereus*(Gram-positive bacteria)	MIC = 32-128 μg/mL	Penicillin, Streptomycin sulfate	The essential oil exhibited broad-spectrum activity against both Gram-positive and Gram-negative bacteria	[1]
Antibacterial effect	Essential oil containing silphiperfol-5-ene (13)	*S. aureus*(ATCC 6538) (Gram-positive)	MIC = 0.5 mg/mL; MBC = 1 mg/mL	Not specified	Inhibited the growth of *S. aureus*	[2]
Antibacterial effect	Essential oil containing Cyperene (21)	*S. aureus*,*Listeria monocytogenes*,*B. cereus*(Gram-positive);*Escherichia coli*,*Salmonella enteritidis*(Gram-negative)	1, 5, 10 μL	Penicillin, Chloramphenicol, Gentamicin, Erythromycin	Increased content of Cyperene may enhance the overall antibacterial activity of the oil via synergistic effects	[3]
Antibacterial & Antifungal effects	Essential oil containing Caryophyllene (25)	*S. aureus*,*B. subtilis*(Gram-positive);*Pseudomonas aeruginosa*,*Acinetobacter baumannii*(Gram-negative);*C. albicans*	MIC: 62.5->1000 μg/mL (varies by strain)	Rifampin, Gentamicin, Nystatin	Proposed mechanisms include disruption of bacterial cell membranes, inhibition of protein/DNA synthesis, and interference with metabolic pathways	[4]
Antibacterial effect	Essential oil containing Aciphyllene (26)	*B. subtilis*,*S. aureus*,*Paenibacillus larvae*(Gram-positive);*E. coli*,*P. aeruginosa*(Gram-negative)	MIC = 0.05–0.80 mg/mL	Chloramphenicol	Activity is possibly related to the disruption of bacterial cell membrane integrity by hydrophobic components	[5]
Antibacterial effect	Humulene (30)	*Chromobacterium violaceum*,*P.*	100 μg/mL	Not specified	Molecular docking suggested potential binding to S. aureus DNA gyrase (PDB: 4PLB)	[6]

Bioactivity	Compound/Extract	Test Subject (in vitro)	Dose (MIC/MBC etc.)	Positive Control	Key Results / Proposed Mechanism	References
Antibacterial effect	Essential oil containing trans-Geranic acid methyl ester (32)	*aeruginosa*(Gram-negative) *Salmonella paratyphi-A*,*Klebsiella pneumoniae*,*Shigella dysenteriae*(Gram-negative)	300 µg/mL	Rifampin, Gentamicin	May disrupt bacterial cell membrane structure due to its hydrophobicity, leading to cell death	[7]
Antibacterial effect	Essential oil containing Germacrene D (35)	*S. aureus*(Gram-positive)	MIC: 25–100 µL/mL	Amoxicillin (10 µg/disk)	Showed strong binding affinity to tyrosyl-tRNA synthetase and topoisomerase II, suggesting inhibition of protein synthesis and DNA replication	[8]
Antibacterial & Antifungal effects	Essential oil containing Guaia-1(10),11-diene (39)	*S. aureus*(Gram-positive);*P. aeruginosa*,*E. coli*(Gram-negative);*Saccharomyces cerevisiae*,*C. albicans*	MIC: 125–2500 µg/mL; MBC/MFC: 500–5000 µg/mL	Vancomycin, Norfloxacin, Amphotericin B	Exhibited broad-spectrum activity against both Gram-positive and Gram-negative bacteria and fungi	[9]
Antimicrobial effect	Essential oil containing 2,6-Octadien-1-ol, 3,7-dimethyl-, acetate (40)	*Cronobacter sakazakii*,*P. aeruginosa*(Gram-negative);*L. monocytogenes* (Gram-positive)	MIC: 0.4%–2.23% (v/v); MBC: 3.02%–4.34% (v/v)	Erythromycin	Molecular docking indicated strong binding to antimicrobial target protein 1R4U (-5.2 kcal/mol)	[10]
Antimicrobial effect	Citronellol (41)	*E. coli*(Gram-negative);*S. aureus*,*Corynebacterium glutamicum*(Gram-positive)	MIC (v/v%): 1% (E. coli), 0.5% (S. aureus), 0.05% (C. glutamicum)	Medium blank, DMSO solvent control	Targets the bacterial cell envelope, disrupting membrane integrity, increasing permeability, and altering surface hydrophobicity	[11]
Antifungal effect	n-Hexane extract of *Curcuma zanthorrhiza* containing *α*-	*Fusarium oxysporum*f. sp.*cubense*(Foc)	Not mentioned	Solvent controls (hexane, ethanol, water)	Terpenes may inhibit fungal growth by disrupting cell membrane structure/permeability and interfering with ion transport proteins	[12]

Bioactivity	Compound/Extract	Test Subject (in vitro)	Dose (MIC/MBC etc.)	Positive Control	Key Results / Proposed Mechanism	References
Antifungal effect	Curcumene (46) Essential oil containing γ-Elemene (49)	*Aspergillus niger*(ATCC 9587)	MIC = 32 μg/mL	Nystatin (8 μg/mL)	*P. thomsonii* essential oil showed strong antifungal activity	[13]
Antimicrobial effect	Essential oil containing Cedrene epoxide (53)	*Salmonella typhimurium*, *P. aeruginosa*,*E. coli*(Gram-negative)	50 μg/mL	Gentamicin, Ampicillin	Potential mechanisms include alteration of membrane permeability and enzyme inhibition	[14]
Antibacterial effect	Aromadendrene oxide (80)	*E. coli*MG1655 (Gram-negative),*Staphylococcus epidermidis*ATCC12228 (Gram-positive)	100 μg/mL (liquid); 10 mg/mL (agar diffusion)	Gentamicin	Showed partial inhibitory activity against *E. coli*	[15]
Antibacterial effect	Agarospirol (65) (from *A. lancea* oil)	*S. aureus*(in vitro) (Gram-positive)	MIC: 0.032 mg/mL; MBC: 0.062 mg/mL	Ciprofloxacin hydrochloride	Molecular docking showed binding to S. aureus DNA gyrase (ΔG < -6 kcal/mol) via H-bonds and π-alkyl contacts, potentially inhibiting replication	[16]
Antibacterial & Anti-biofilm effects	2-methyl-5-(1-methylethyl)-Phenol (68) (from *Penicillium gladioli*)	Clinical isolates of*P. aeruginosa*(Gram-negative)	64–512 μg/mL (extract)	Ciprofloxacin	Not mentioned	[17]
Antifungal effect	2,4-Di-tert-butylphenol (78)	*Ustilaginoidea virens*(mycelium & spores)	EC_{50} = 0.087 mmol/L (17.94 mg/L)	Not set	Transcriptomics indicated effects on microtubule assembly, cell wall/membrane-related genes, and toxin synthesis pathways	[17]
Antibacterial & Anti-biofilm effects	Heneicosane (from *Jatropha oil*) (83)	*E. coli*(Gram-negative)	Tested as part of essential oil	Gentamicin (antibacterial), None (anti-biofilm)	In silico docking showed the strongest binding to *E. coli* FimH (ΔG = -30.68 kcal/mol), suggesting inhibition of bacterial adhesion and biofilm formation	[18]
Antibacterial effect	CXE oil (containing 18.48% Valerenol) (81)	*S. aureus*(Gram-positive)	MIC = 78 μg/mL	1% Gentamicin	Showed moderate antibacterial activity, potentially associated with	[19]

Bioactivity	Compound/Extract	Test Subject (in vitro)	Dose (MIC/MBC etc.)	Positive Control	Key Results / Proposed Mechanism	References
					Valerenol and other constituents	
Antibacterial & Antifungal effects	Caryophyllene oxide (88)	*E. coli*(Gram-negative),*S. aureus*(Gram-positive),*C. albicans*	Not mentioned	Gentamicin, Clotrimazole	Demonstrated good binding affinity in molecular docking (e.g., -7.00 kcal/mol for S. aureus) mainly via alkyl and hydrogen bond interactions	[20]
Antibacterial effect	Octacosane (100) (from *Cucumis spp.* oils)	*E. coli*,*P. aeruginosa*(Gram-negative);*S. aureus*,*Streptococcus pyogenes*(Gram-positive)	200, 100, 50 μg/mL	Ceftriaxone	A major constituent (up to 51.79%) in the essential oils	[21]
Antibacterial effect	*Essential oil* containing Spathulenol (105)	*Xanthomonas citri*(Gram-negative)	100 μg/mL	Copper treatment	Showed antibacterial activity against all three tested strains	[22]
Antibacterial effect	n-Hexadecanoic acid (108)	*S. aureus*,*B. subtilis*(Gram-positive)	250–500 μg/mL	Gentamycin	Essential oils from leaves and roots showed moderate to strong inhibition against Gram-positive bacteria	[23]
Antibacterial effect	6-Octadecenoic acid (113) (from *N. dassonvillei* $CHCl_3$ extract)	*L. monocytogenes* (Gram-positive), Shiga toxin-producing*E. coli*(Gram-negative)	100 mg/mL	Ciprofloxacin	In silico predictions suggested potential inhibition of key enzymes (e.g., DHFR, DNA gyrase B)	[24]
Antibacterial effect	α-Thujene (143) (from Sphagneticola trilobata oil)	*P. aeruginosa*PAO1 (Gram-negative)	Not mentioned	Ciprofloxacin	Molecular docking showed high binding affinity to virulence targets	[25]
Antibacterial effect	1,3,8-p-Menthatriene (145) (from *Eucalyptus globulus*)	Bacterial strains	Not mentioned	Erythromycin	Molecular docking showed binding to antimicrobial protein 1R4U (-5.0 kcal/mol)	[10]
Antibacterial & Antifungal effects	trans-Geranic acid methyl ester (148) (from *Dracocephalum kotschyi*)	Various bacterial strains	300 μg/mL	Rifampin, Gentamicin, Nystatin	Proposed to act by disrupting microbial cell membranes, increasing permeability, and causing electrolyte leakage	[7]

Bioactivity	Compound/Extract	Test Subject (in vitro)	Dose (MIC/MBC etc.)	Positive Control	Key Results / Proposed Mechanism	References
Antibacterial effect	2,6-Octadien-1-ol, 3,7-dimethyl-, acetate, (Z)- (150) (from *Plumeria alba*)	*E. coli*,*Salmonella* sp. (Gram-negative)	Not mentioned	Not mentioned	Showed significant inhibitory activity against the tested strains	[26]
Antibacterial & Antifungal effects	Acetic acid, decyl ester (151) (from *Pittosporum angustifolium*)	*S. aureus*,*S. epidermidis*(Gram-positive),*C. albicans*	MIC: 0.25%, 0.0625%, 0.25% (respectively)	Tetracycline	NS166 oil exhibited strong activity, with the strongest inhibition against S. epidermidis	[27]
Antibacterial effect	α-Calacorene (193) (from *Cyperus articulatus* oil)	*S. aureus*TyrRS (Gram-positive)	Not mentioned	Chloramphenicol	Molecular docking score was -6.2 kcal/mol, comparable to chloramphenicol (-6.3 kcal/mol)	[28]
Antibacterial effect	Tricyclene (128) (from *Cistus albidus*)	*E. coli*(Gram-negative),*S. aureus*(Gram-positive)	MIC: 1% - 8% (v/v)	Gentamicin	Showed moderate binding affinity to bacterial DHFR in silico, suggesting a potential contribution to overall oil activity via folate synthesis inhibition	[29]
Antibacterial effect	Thujopsene (from stem/seed ethanol extract) (158)	*B. subtilis*,*S. aureus*(Gram-positive);*P. aeruginosa*,*E. coli*(Gram-negative)	Not mentioned	Streptomycin	Mechanism may be related to differences in cell wall structure and synergistic effects among multiple compounds in the extract	[30]
Antibacterial effect	α-Citral (189)	*Vibrio parahaemolyticus*(Gram-negative)	150 μg/mL	Not mentioned	Directly inhibited bacterial growth, but exhibited poor stability, volatility, and short duration of action	[30]
Antifungal effect	(1S,4S,4aS)-1-Isopropyl-4,7-dimethyl-1,2,3,4,4a,5-hexahydronaphthalene (204)	Fungal pathogen*Escovopsis weberi*	Not mentioned	Not mentioned	Indicated that such compounds are common in plant essential oils and possess antifungal potential	[31]
Antibacterial effect	Isoledene (208) (from *Ocimum americanum* L. oil)	*S. aureus*(Gram-positive),*E. coli*(Gram-negative)	Not mentioned	Streptomycin, Cefotaxime	Activity may be related to synergistic effects among multiple components in the essential oil	[32]
Antibacterial effect	Benzaldehyde, 2,4-dihydroxy-3,6-dimethyl- (216) (from	MSSA (ATCC 25923) and 20 clinical MRSA isolates	30 mg/ml, 3 mg/ml (tested)	Vancomycin	Traric acid is suggested to be one of the main antibacterial components in the extract	[33]

Bioactivity	Compound/Extract	Test Subject (in vitro)	Dose (MIC/MBC etc.)	Positive Control	Key Results / Proposed Mechanism	References
	Parmotrema spp.)	(Gram-positive)				
Antiviral effect	Quinic acid (235) derivatives	Huh7.5 cells, human PBMCs	NTC (Non-toxic concentration)	IFN-α-2A	Inhibits dengue virus replication without affecting viral adsorption and internalisation, acting at the replication stage	[34]
Antibacterial effect	Atractylodin (141)	Mice (C57BL/6, Balb/C), J774A.1 macrophages	4 μg/mL (in vitro); 30–60 mg/kg/day (in vivo)	Not mentioned	Inhibits pore formation by *Listeria hemolysin* O, modulates the Nrf2/NLRP3 pathway, and alleviates inflammation and oxidative stress	[35]
Anti-influenza virus effect	Furanodienone (137)	A549 cells	80 μg/mL	Not mentioned	Effective during both pre-infection and post-infection stages, potentially interfering with cell membrane factors or maintaining cellular reduced states	[36]
Antiviral effect	β-Pinene (119)	HeLa cells	18.7–300 μg/mL	Ribavirin	Directly inactivates viruses, inhibiting viral adsorption and replication without compromising cellular protection	[37]
Antiviral effect	Atractyloside A (243)	A549, MDCK cells; C57BL/6 mice	20–150 μM (in vitro); 10–30 mg/kg (in vivo)	Not mentioned	Activate IRF3 phosphorylation and the type I interferon pathway, promote macrophage M2 polarisation, and inhibit viral replication	[38]

Note: ↑ represents an increase, and ↓ represents a decrease.

Table S4. Anti-inflammatory effect of Atractylodis Rhizoma

Bioactivity	Compounds/Extracts	Testing subjects (Animal/model)	Dose	Positive control	Results/Mechanism	References
Anti-inflammatory effect	2-Isopropenyl-4a, 8-dimethyl-1,2,3,4,4a, 5,6,7-octahydronaphthalene (31) from *N. acuminata* leaf essential oil	In vitro (Soybean lipoxygenase, LOX)	0.0125–0.100 mg/mL	Nordihydroguaiaretic acid	Lipoxygenase↓, Leukotrienes↓	[39]
Anti-inflammatory effect	kaur-16-ene (90) derivatives	RAW264.7 macrophages	0.77–100 μM	Dexamethasone	NO ↓, TNF-*α*↓, IL-6↓	[40]
Anti-inflammatory effect	Eucalyptol (115)	C57BL/6 mice	30, 100, 300 mg/kg	Ibuprofen	IL-1β↓, TNF-*α*↓, IL-6↓, TRPM8↑	[41]
Anti-inflammatory effect	γ-Terpinene (116)	Swiss mice	25, 50 mg/kg	Indomethacin	leukocyte migration ↓, neutrophil (PMN) migration ↓, IL-1β↓, TNF-*α* ↓	[42]
Anti-inflammatory effect	Terpinen-4-ol (131)	RAW264.7 macrophages	50–200 μmol/L	Not mentioned	IL-6 ↓, TNF-*α*↓, ECAR/OCR↓, GLS↑, GDH↑, mTOR ↓, NF-κB ↓	[43]
Anti-inflammatory effect	Isoelemicin (136)	RAW264.7 macrophages	100 μM	Kaempferol	NO↓	[44]
Anti-inflammatory effect	Guaiol (191)	RAW264.7 macrophages	100 μM	Not mentioned	NO↓, IL-6 ↓, TNF-*α*↓	[45]
Anti-inflammatory effect	Cryptochlorogenic acid (245)	RAW264.7 macrophages	20–80 μM	Not mentioned	NO↓, TNF-*α*↓, IL-6↓, iNOS↓, COX-2↓, GSH/GSSG↑, SOD↑, MDA↓, p65↓, p-JNK↓, p-ERK↓, p-p38↓, Nrf2↑, HO-1↑	[46]
Anti-inflammatory effect	Naringenin chalcone (253)	CD-1 mice	2% (AA model), 1% (TPA model)	Disodium diclofenac	Prostaglandins/Leukotrienes↓	[47]
Anti-inflammatory effect	Syringin (285)	RAW264.7 macrophages	0.5, 1.5, 4.5 μM	Not mentioned	IL-6↓	[48]

Note: ↑ represents an increase, and ↓ represents a decrease.

Table S5. Antioxidant effect of Atractylodis Rhizoma

Bioactivity	Compounds/ Extracts	Testing subjects (Animal/model)	Dose	Positive control	Results/Mechanism	References
Antioxidant effect	trans-Longipinocarveol (55) from *I. oculus-christi* oil	In vitro DPPH, ABTS, etc.	Comprehensive Essential Oil Testing	Not mentioned	DPPH scavenging rate (57.4%), ABTS scavenging rate (82.7%), FRAP value (24.42 μg Fe/mg), TRP value (2.60 μg AAE/mg), CUPRAC value (26.94 μg TE/mg)	[49]
Antioxidant effect	γ-Terpinene (116)	SH-SY5Y cell	50 nM	α-Tocopherol	Lipid peroxidation chain reaction↓, Oxidative damage to cell membranes↓	[50]
Antioxidant effect	γ-Terpinene (116)	Homogeneous solution	Not mentioned	α-Tocopherol	HOO^{+} reduces phenoxy radicals/quinones	[51]
Antioxidant effect	*Ocimum sanctum essential* oil containing β-Bisabolene (122)	Containing β-Bisabolene	100μg/mL	Ascorbic acid	Clearance rate: 96.48% (Bishoftu) / 96.17% (Debre Berhan), approaching that of ascorbic acid (98.08%)	[52]
Antioxidant effect	Ethanone, 1-(2,4,6-trihydroxyphenyl)-(139)	In vitro chemical system	6.83% (in essential oils)	Ascorbic acid	IC_{50} EO=1.29 μg/mL vs Ascorbic acid=20.0 μg/mL	[53]
Antioxidant effect	*Senecio glaucus* oil containing (+)-carene (152)	In vitro DPPH	8.39% (in essential oils)	Ascorbic acid	Ability to scavenge DPPH radicals↑	[54]
Antioxidant effect	1,4,7,-Cycloundecatriene, 1,5,9,9-tetramethyl-, Z,Z,Z-(209)	In vitro chemical and computational modelling	As an extract component	Ascorbic acid	Binding energy with nitric oxide synthase (6NGJ) = −7.0 kcal/mol, primarily via hydrophobic interactions	[55]
Antioxidan	Atractylenolactam (258)	HEK293T cell	10–30 μM	Tert-Butyl hydr	Nrf2↑	[56]

Bio acti vity	Compounds/ Extracts	Testing subjects (Animal/m odel)	Dose	Positi ve contr ol	Results/Mechanism	R ef er en ce s
t effe ct				oquin one		

Note: ↑ represents an increase, and ↓ represents a decrease.

Table S6. Hepatoprotective effect of Atractylodis Rhizoma

Bioactivity	Compounds/Extracts	Testing subjects (Animal/model)	Dose	Positive control	Results/Mechanism	References
Anti-fatty liver effect	Atractylodin (141)	C57BL/6N mice	5, 10 mg/kg	Not mentioned	Srebf1↓, Fasn↓, Scd2↓, Dgat2↓, AMPK↑	[57]
Resistance to cisplatin-induced liver injury	Eucalyptol (115)	Wistar albino rats	100 mg/kg	Not mentioned	MDA↓; GSH↑, G6PD↑, CAT↑; iNOS↓, 8-OHdG↓	[58]
Hepatoprotective effect	Neochlorogenic acid (240)	db/db Mice	177.4 μg/d	Not mentioned	AST↓, ALT↓, TG↓, CHO↓, LDL-C↓; GSH↑, GPx↑, SOD↑, Catalase↑; SREBP-1↓, FASN↓, PPARα↑, CPT-1 ↑	[59]
Improve cholestatic liver fibrosis	Senkyunolide A (294)	In vivo: C57BL/6J mice; In vitro: Human cholangiocytes	In vivo: 10, 20, 40 mg/kg; In vitro: 12.5, 25, 50 μmol/L	Not mentioned	CLCC1 ubiquitination↑, cytoplasmic Ca^{2+}↓, Endoplasmic reticulum (ER) autophagy↓	[60]
Improve liver fibrosis	Germacrone (305)	In vitro: HSCs cell, LO2 cell; In vivo: C57BL/6 mice	In vitro: 25–100 μg/mL; In vivo: 8, 16, 32 mg/kg	Not mentioned	JAK2↓, STAT3↓, Type I collagen↓	[61]

Note: ↑ represents an increase, and ↓ represents a decrease.

Table S7. Anti-cancer effect of Atractylodis Rhizoma

Bioactivity	Compounds/ Extracts	Testing subjects (Animal/model)	Dose	Positive control	Results/Mechanism	References
Anti-prostate cancer effect	Containing Nerol (50) of Cinnamomi Cortex oil	PC-3cell	Not mentioned	Not mentioned	High binding affinity for Bcl-2 (-6.8 kcal/mol)	[57]
Anti-nasopharyngeal carcinoma effect	β-Eudesmol (71)	In vitro: HK1, SUNE1, NP69 cell; In vivo: BALB/c mice	In vitro: 50–200 μM; In vivo: 2.5 mg/kg/d	AZD4547	Blocking FGF1/FGFR signalling→Akt↓, STAT3↓, ERK↓→β-catenin↓, ZEB1↓, TWIST1↓, Vimentin↓→ABCC1↓	[62]
Anti-cancer effect	Atractylodin (141)	In vitro: HCT116, HT29 cell; In vivo: C.B-17 SCID mice	In vitro: 5–80 μM; In vivo: 5–100 mg/kg/d	Not mentioned	PI3K↓, Akt↓, mTOR↓, p70S6K↓	[63]
Anti-gastric cancer effect	Cryptomeridiol (104)	HGT-1 cell	50–200 μg/mL	5-Fluorouracil	p21↑, CDK2↓; caspase-3↑, caspase-9↑, XIAP↓	[64]
Anti-melanoma effect	P-cymene (185)	BALB/c mice	60 mM	Dacarbazine	Bcl-2↓, Bcl-xl↓, Caspase-3↑	[65]
Anti-cancer effect	Guaiol (191)	A549, Calu-1 cell	60–120 μM	Z-VAD-FMK	PI3K↓, Akt↓, Bcl-2↓	[66]
Anti-cancer effect	Guaiol (191)	In vitro: LLC cell; In vivo: BALB/c mice	In vitro: 60 μM; In vivo: 8 mg/kg	Not mentioned	M2 macrophage polarisation↓, IL-10↓, STAT3↓, EMT↓	[67]
Anti-cancer effect	Chlorogenic acid (242)	In vitro: A375, SK-OV-3, MDA-MB-231 cell; In vivo: BALB/c mice	In vitro: 0–200 μM; In vivo: 50 mg/kg	Not mentioned	p-STAT1↓, IRF1↓, PD-L1↓; CD8+ Tcell↑, Granzyme↑	[68]
Anti-tumour effect	Self-assembled Cu@Chlorogenic acid nanoparticles (242)	In vitro: CT26 cell; In vivo: BALB/c mice	In vitro: 0–300 μg/mL; In vivo: 300 μg per animal	Chlorogenic acid	ROS↑, GSH↓; caspase-1↑, GSDMD↑; HMGB1↑, CRT↑; CD8+ T cell↑, M2→M1 polarisation	[69]
Anti-lung cancer effect	Cryptochlorogenin acid (245)	In vitro: LA795 cell; In vivo: Balb/c mice	In vitro: 10–200 μM; In vivo: 50/100 μM	T16Ainh-A01	p-ERK1/2↓, p-MEK1/2↓, Cyclin D1↓; N-cadherin↓, β-catenin↓, vimentin↓, E-cadherin↑; caspase-3↑, Bax/Bcl-2↑	[70]
Inhibiting colonitis-associated	Vitexin (252)	In vitro: THP-1, RAW264.7, CT26.WT,	In vitro: 5–20 μM; In vivo: 10 mg/kg	Calcitriol	PBLD↑, VDR↑; Macrophage M1	[71]

Bioactivity	Compounds/ Extracts	Testing subjects (Animal/model)	Dose	Positive control	Results/Mechanism	References
carcinogenesis		HCT116cell; In vivo: Lyz2-cre mice			polarisation↑, M2 polarisation↓	
Anti-gastric cancer effect	dehydrocostus lactone(256)	In vitro: MKN-28, AGS cell; BALB/c mice	In vitro: 5–15 μM; In vivo: 15–30 mg/kg	5-Fluorouracil	ACLY↓, ACC↓, FASN↓; LAMP1↓, LAMP2↓	[72]
Inhibiting lung cancer metastasis	Atractylenolide II (268)	In vitro: A549 cell; In vivo: BALB/c mice	In vitro: 2.5–5 μM; In vivo: 50 mg/kg/d×21day	Not mentioned	M2 polarisation↓, STAT6↓; lung metastasis↓	[73]
Anti-Colorectal Cancer	Atractylenolide II (268)	In vitro: HT29, HCT15 cell; In vivo: BALB/c mice, C57BL/6 mice	In vitro: 80 μmol/L + 250 ng/ml IFN-γ; In vivo: 50 mg/kg + 0.3 mg/kg IFN-γ	Not mentioned	PD-L1↓, p38 MAPK↓, FAK↓; CD4+/CD8+ T cell↑	[74]
Anti-endometrial cancer effect	Atractylenolide II (268)	RL95-2, AN3CA cell	100–200 μM	Not mentioned	PKM2↓, LDHA↓; Bcl-2↓, Bax↑, cleaved-PARP↑	[75]
Anti-liver cancer effect	Atractylenolide II (268)	In vitro: Hep3B, Huh7cell; In vivo: BALB/c mice	In vitro: 50–200 μM; In vivo: 50 mg/kg/d×4weeks	Not mentioned	Fe^{2+}↑, ROS↑, MDA↑, GSH↓, GPX4↓, xCT↓; CD8+ T cell↑, IFN-γ↑, IL-10↓, PD-L1↓; TRAF6↓	[76]
Anti-renal cell carcinoma and anti-angiogenic effects	Atractylenolide I (271)	In vitro: 786O, OSRC2, ACHN, HK-2 cell; In vivo: mice	In vitro: 80–160 μM; In vivo: 25–50 mg/kg	Bevacizumab, Sunitinib	ATP6V0D2↑→VEGFA↓→angiogenesis↓	[77]
Improving Cancer Cachexia effect	Atractylenolide I (271)	In vitro: C26, C2C12 and 3T3-L1 cells; In vivo: BALB/c mice	In vitro: 0.31–5 μM; In vivo: 25 mg/kg	GW4869, PDTC	STAT3↓, PKM2↓, SNAP23↓→IL-6↓, EV secretion↓	[78]
Anti-bladder cancer effect	Atractylenolide I (271)	In vitro: T-24, 253J, RT4 and 5637 cells; In vivo: BALB/c mice	In vitro: 10–30 μM; In vivo: 25–75 mg/kg	Z-DEVD-fmk, Z-LEHD-fmk	p21↑; Bax↑, Bcl-2↓, caspase-3/9↑; PI3K↓	[79]
Anti-bladder cancer effect	Isoalantolactone (298)	In vitro: PC-3, DU145 cells; In vivo: BALB/c mice	In vitro: 2.5–60 μM; In vivo: 10 mg/kg	Not mentioned	ROS↑, STAT3↓	[80]

Bioactivity	Compounds/ Extracts	Testing subjects (Animal/model)	Dose	Positive control	Results/Mechanism	References
Inhibiting the growth of lung adenocarcinoma	δ-Elemene (319)	A549, PC9 cells	Not mentioned	Not mentioned	Interference with glutathione synthesis → oxidative stress	[81]
Anti-cancer effect	Longifolene-(V4) (103)	MCF-7, A-549 cells	Not mentioned	Not mentioned	Molecular docking analysis indicates high binding affinity for p53, BCL2, EGFR, and HER2 (-5.4 to -6.6 kcal/mol)	[82]
Anti-lung cancer effect	Valencene (327)	A549 cells	5–20 μg/ml(IC_{50} 16.71 μg/ml)	5-Fluorouracil	Mitochondrial membrane potential↓	[83]

Note: ↑ represents an increase, and ↓ represents a decrease.

Table S8. Intestinal regulatory function of Atractylodis Rhizoma

Bioactivity	Compounds/Extracts	Testing subjects (Animal/model)	Dose	Positive control	Results/Mechanism	References
Anti-ulcerative colitis effect	Hinesol (66)	In vivo: C57BL/6 mice; In vitro: RAW 264.7 cell	In vivo: 5, 10, 20 mg/kg/day; In vitro: 2.5, 5, 10 μg/ml	Not mentioned	ZO-1↑, Occludin↑, Claudin-1↑; TNF-α↓, IL-1β↓, IL-18↓, IL-6↓; SOD↑, GSH-px↑, CAT↑, MDA↓; Src↓, XCL1↓, CCL2↓, CXCL16↓	[84]
Anti-colitis effect	Atractylodin (141)	BALB/c mice	10, 20 mg/kg	Sulfasalazine	DAI Score↓; ZO-1↑, occludin↑, MUC2↑; MAPK↓; TNF-α↓, GAPDH malonylation↓	[85]
Anti-colitis effect	Atractylodin (141)	C57BL/6 mice	40 mg/kg	Rosiglitazone	NF-κB↓	[86]
Relieve constipation	Atractylodin (141)	SD rats	10 mg/kg	Not mentioned	TNF-α↓, IL-1β↓, IL-6↓; iNOS↓, NF-κB↓	[87]
Anti-colitis effect	Furanodienone (137)	C57BL/6J mice	10 mg/kg	Pregnenolone 16α-carbonitrile	Cyp3a11↑, Mdr1a↑, Cyp2b10↑, GSTs↑; NF-κB↓, IL-6↓, IL-1β↓, TNF-α↓; Occludin↑, ZO-1↑;	[88]
Anti-gastrointestinal inflammation effect	Carveol (146)	SD rats	10, 30, 50 μg/kg	Omeprazole	TNF-α↓, IL-8↓, PGE2↑; GSH↑, GST↑, CAT↑, LPO↓; H^+/K^+-ATPase↓	[89]
Anti-colitis effect	Vanillic acid (233)	C57BL/6 mice	12.5, 25, 50 mg/kg/day	Mesalazine	DAI Score↓, MUFA↑, IL-1β↓, IL-6↓, IL-17A↓, TNF-α↓, IL-23↓	[90]
Anti-ulcerative colitis effect	Quinic acid (235)	Wistar rats	10, 30, 60, 100 mg/kg	Dexamethasone	MDA↓, NO↓; TAC↑, SOD↑, CAT↑; TNF-α↓, IL-1β↓, IL-6↓, TLR4↓, iNOS↓, NF-κB↓; Caspase-3↓, Caspase-8↓, Bax↓	[91]
Improving post-infection irritable bowel syndrome	Chlorogenic Acid (242)	SD rats	50 mg/kg/day	Not mentioned	IL-1β↓, IL-6↓, TNF-α↓; MLCK↓, p-MLC↓, ZO-1↑, Occludin↑; Bacteroides acidifaciens↑, Glycine↑	[92]
Anti-ulcerative colitis effect	Atractylenolide III (251)	In vivo: C57BL/6 mice; In vitro: RAW264.7 Macrophage	In vivo: 10 mg/kg, 20 mg/kg; In vitro: 100 μM, 200 μM	Rosiglitazone	DAI Score↓; GSH-Px↑, SOD↑, ↓MDA; ZO-1↑, Occludin↑, Claudin1↑, LPS↓, D-lac↓; NF-κB↓, MAPK↓; IL-1β↓, IL-6↓, TNF-α↓, MCP-1↓, iNOS↓	[93]
Anti-enteritis effect	Dehydrocostus lactone (256)	Kunming mice	7.5, 15, 30 mg/kg	Loperamide	TLR4/MD2↓; NF-κB↓, NLRP3↓	[94]
Anti-ulcerative colitis effect	Dehydrocostus lactone (256)	C57BL/6 mice	6, 12, 24 mg/kg	Rosiglitazone	TLR4↓, NF-κB p65↓, PI3K↓	[95]

Bioactivity	Compounds/Extracts	Testing subjects (Animal/model)	Dose	Positive control	Results/Mechanism	References
Improving intestinal barrier function	Acetylatractylodinol (270) from ethanol extracts	In vivo: BALB/c mice; In vitro: Caco-2 cell	In vivo: 555, 1110 mg/kg; In vitro: 12.5, 25μg/mL	Sulfasalazine	PI3K↑, AKT↑	[96]
Anti-enteritis effect	α-Cyperone (273)	In vivo: C57BL/6 mice; In vitro: Caco2 cell	In vivo: 10 mg/kg/d; In vitro: 10 μmol/L	Rosiglitazone	DAI Score↓, TEER↑, ZO-1↑, claudin-1↑; IL-6↓, TNF-α↓, IL-1β↓, IFN-γ↓, TLR4↓, MyD88↓, p-p65↓	[97]
Anti-enteritis effect	δ-Elemene(319)	BALB/c mice	45, 135 mg/kg/d	Rosiglitazone	Firmicutes↑, Proteobacteria↓, TNF-α↓, IL-6↓	[98]

Note: ↑ represents an increase, and ↓ represents a decrease.

1. He, F.; Wang, W.; Wu, M.C.; Fang, Y.P.; Wang, S.Z.; Yang, Y.; Ye, C.; Xiang, F. Antioxidant and antibacterial activities of essential oil from *Atractylodes lancea* rhizomes. *Ind. Crops Prod.* **2020**, *153*, doi:10.1016/j.indcrop.2020.112552.
2. Jirakitticharoen, S.; Wisuitiprot, W.; Jitareerat, P.; Wongs-Aree, C. Terpenoids and Bio-Functions of Essential Oils Hydrodistilled Differently from Freshly Immature and Mature *Blumea balsamifera* Leaves. *J. Trop. Med.* **2023**, *2023*, doi:10.1155/2023/5152506.
3. Ben Hassine, D.; El Euch, S.K.; Rahmani, R.; Ghazouani, N.; Kane, R.; Abderrabba, M.; Bouajila, J. *Clove Buds Essential Oil*: The Impact of Grinding on the Chemical Composition and Its Biological Activities Involved in Consumer's Health Security. *BioMed Res. Int.* **2021**, *2021*, doi:10.1155/2021/9940591.
4. Zhang, X.C.; Zhu, L.; Li, X.Y.; Liu, L.C.; Lai, P.X. Chemical Composition, and Evaluation of Antibacterial, Antibiofilm and Synergistic Effects with Conventional Antibiotics of Essential Oil from *Mallotus repandus*. *Rec. Nat. Prod.* **2021**, *15*, 324-329, doi:10.25135/rnp.217.20.10.1854.
5. Ghavam, M.; Bacchetta, G.; Castangia, I.; Manca, M.L. *Phlomoides molucelloides* (Bunge) Salmaki essential oil: a traditional remedy revitalized for modern antimicrobial challenges. *Inflammopharmacology* **2025**, *33*, 7033-7043, doi:10.1007/s10787-025-02004-5.
6. Prabaharan, J.; Prabakaran, M.; Prabhakaran, M.; Abinaya, K.; Krishnan, N.; Karen, D.S.; Veena, J.; Dhanbalan, A.K.; Devadasan, V.; Gopinath, S.C.B.; et al. Comparison on extracted metabolites from different regions grown *Murraya koenigii* and validation by antibacterial, antioxidant, and molecular docking studies. *Biomass Convers. Biorefin.* **2025**, *15*, 23835-23863, doi:10.1007/s13399-023-04105-z.
7. Ghavam, M. A GC-MC analysis of chemical compounds and identification of the antibacterial characteristics of the essential oil of two species exclusive to Iranian habitats: New chemotypes. *PloS one* **2022**, *17*, e0273987, doi:10.1371/journal.pone.0273987.
8. Ladjel-Mendil, A.; Amarni, M.; Chelghoum, H.; Lacheheb, S.; Moussa, H.; Aboumustapha, M.; Chebrouk, F.; Boudjelal, A.; Kebir, M.; Benguerba, Y.; et al. Essential oil from aerial parts of *Salvia sclarea*: A comprehensive study of antibacterial activity through chemical profiling, molecular interactions, and predictive modeling using QSAR_KNN_PCA. *J. Essent. Oil Bear. Plants* **2025**, *28*, 902-919, doi:10.1080/0972060X.2025.2559656.
9. Sitarek, P.; Rijo, P.; Garcia, C.; Skała, E.; Kalemba, D.; Białas, A.J.; Szemraj, J.; Pytel, D.; Toma, M.; Wysokińska, H.; et al. Antibacterial, Anti-Inflammatory, Antioxidant, and Antiproliferative Properties of Essential Oils from Hairy and Normal Roots of *Leonurus sibiricus* L. and Their Chemical Composition. *Oxid. Med. Cell. Longevity* **2017**, *2017*, 7384061, doi:10.1155/2017/7384061.
10. Nasir Shah, S.; Khan, I.; Tul Muntaha, S.; Hayat, A.; Ur Rehman, M.; Ali Shah, T.; Siddique, F.; Salamatullah, A.M.; Mekonnen, A.B.; Bourhia, M. Bioactive, antioxidant and antimicrobial properties of chemically fingerprinted essential

oils extracted from *Eucalyptus globulus*: in-vitro and in-silico investigations. *Front. Chem.* **2023**, *11*, 1287317, doi:10.3389/fchem.2023.1287317.
11. Wei, T.; Regeard, C.; Barroca-Aubry, N.; Roger, P.; Aymes-Chodur, C. Chemoenzymatic oxidation of citronellol and geraniol: Synthesis and antibacterial activity assessment. *Colloids Surf., B* **2025**, *253*, 114723, doi:10.1016/j.colsurfb.2025.114723.
12. Napitupulu, T.P.; Purnaningsih, I.; Kanti, A.; Sudiana, I.M. Comparative evaluation on chemical composition and in vitro anti- Fusarium activity of solvent extracts of Zingiberaceae rhizomes from Indonesia. *Kuwait J. Sci.* **2024**, *51*, doi:10.1016/j.kjs.2024.100281.
13. Huong, L.; Dai, D.N.; Hung, N.H.; Luyen, N.D.; Pham, T.V.; Linh, N.N.; Son, N.T. *Piper hainanense* Hemsl. and P. thomsonii (C.DC.) Hook.f.: Essential Oil Compositions, Antimicrobial and Mosquito Larvicidal Activities. *Natural Product Communications* **2025**, *20*, doi:10.1177/1934578x251324002.
14. Ospina, L.M.P.; Muñoz, P.B.; Matulevich, J.; Teherán, A.A.; Villamizar, L.B. Composition and Antimicrobial Activity of the Essential Oils of Three Plant Species from the Sabana of Bogota (Colombia): *Myrcianthes leucoxyla*, *Vallea stipularis* and *Phyllanthus salviifolius*. *Nat. Prod. Commun.* **2016**, *11*, 1913-1918.
15. Carev, I.; Gelemanovic, A.; Glumac, M.; Tutek, K.; Dzelalija, M.; Paiardini, A.; Prosseda, G. *Centaurea triumfetii* essential oil chemical composition, comparative analysis, and antimicrobial activity of selected compounds. *Sci. Rep.* **2023**, *13*, doi:10.1038/s41598-023-34058-2.
16. Xu, X.J.; Xiong, X.H.; He, Z.Q.; Lu, Q.; Wang, L. Optimization of ultrasound pretreatment combined with solvent-free microwave for extracting volatile oil from *Atractylodes lancea* and its chemical composition and antimicrobial activity. *Talanta* **2026**, *297*, doi:10.1016/j.talanta.2025.128724.
17. Elekhnawy, E.; Moglad, E.; Sirag, N.; Ahmed, R.; Abd El-Salam, N.; Younis, S.S.; Rashed, H.A.; Elmorsy, E.A.; Salama, L.A.; Al-Fakhrany, O.M. Multifunctional bioactivity of eco-friendly *Penicillium gladioli* extract against *Toxoplasma gondii* and *Pseudomonas aeruginosa*. *Sci. Rep.* **2025**, *15*, doi:10.1038/s41598-025-23921-z.
18. El-Din, M.I.G.; Youssef, F.S.; Altyar, A.E.; Ashour, M.L. GC/MS Analyses of the Essential Oils Obtained from Different *Jatropha* Species, Their Discrimination Using Chemometric Analysis and Assessment of Their Antibacterial and Anti-Biofilm Activities. *Plants-Basel* **2022**, *11*, doi:10.3390/plants11091268.
19. David, A.; Wang, F.; Sun, X.M.; Li, H.N.; Lin, J.R.; Li, P.L.; Deng, G. Chemical Composition, Antioxidant, and Antimicrobial Activities of *Vetiveria zizanioides* (L.) Nash Essential Oil Extracted by Carbon Dioxide Expanded Ethanol. *Molecules* **2019**, *24*, doi:10.3390/molecules24101897.
20. Fufa, M.; Deresa, D.A.; Abdissa, N. GC-MS Analysis, Antimicrobial Activity, and In Silico Docking of Essential Oil Constituents From *Piper capense* Seeds. *J. Chem.* **2025**, *2025*, doi:10.1155/joch/5572855.

21. Assefa, T.; Tesso, H.; Abdisa, E.; Guta, L.; Melaku, Y. Chemical composition and antibacterial activity of essential oils from selected species of the genus *Cucumis* in Ethiopia. *Bull. Chem. Soc. Ethiop.* **2023**, *37*, 703-715, doi:10.4314/bcse.v37i3.13.
22. da Silva, I.R.R.; Fernandes, C.C.; Gonçalves, D.S.; Martins, C.H.G.; Miranda, M.L.D. Chemical composition and anti-Xanthomonas citri activities of essential oils from *Schinus molle* L. fresh and dry leaves and of its major constituent spathulenol. *Nat. Prod. Res.* **2024**, *38*, 3476-3480, doi:10.1080/14786419.2023.2249584.
23. Kiashi, F.; Momeni Nasab, F.; Tavakoli, S.; Aghaahmadi, M.; Goodarzi, S.; Pirali Hamedani, M.; Heidari, F.; Hadjiakhoondi, A.; Tofighi, Z. *Trigonella teheranica:* a valuable source of phytochemicals with antibacterial, antioxidant and cytotoxic properties. *Nat. Prod. Res.* **2022**, *36*, 6405-6409, doi:10.1080/14786419.2022.2032694.
24. Ngema, S.S.; Khumalo, S.H.; Ojo, M.C.; Pooe, O.J.; Malilehe, T.S.; Basson, A.K.; Madoroba, E. Evaluation of Antimicrobial Activity by Marine *Nocardiopsis dassonvillei* against Foodborne *Listeria monocytogenes* and Shiga Toxin-Producing Escherichia coli. *Microorganisms* **2023**, *11*, doi:10.3390/microorganisms11102539.
25. Hassan, W.H.B.; Ghani, A.E.A.; Taema, E.A.; Yahya, G.; El-Sadek, M.E.; Mansour, B.; Abdel-Halim, M.S.; Arafa, A.M. Chemical profile, virtual screening, and virulence-inhibiting properties of *Sphagneticola trilobata* L. essential oils against *Pseudomonas aeruginosa*. *Sci. Rep.* **2025**, *15*, 11964, doi:10.1038/s41598-025-94486-0.
26. Ferdosi, M.F.H.; Naseem, M.K.; Afzal, A.; Khan, I.H.; Javaid, A. Potential antimicrobial compounds in flower extract of *Plumeria alba*. *Arabian J. Chem.* **2023**, *16*, doi:10.1016/j.arabjc.2023.104719.
27. Sadgrove, N.J.; Jones, G.L. Chemical and biological characterisation of solvent extracts and essential oils from leaves and fruit of two Australian species of *Pittosporum* (Pittosporaceae) used in aboriginal medicinal practice. *J. Ethnopharmacol.* **2013**, *145*, 813-821, doi:10.1016/j.jep.2012.12.019.
28. Swain, A.; Choudhir, G.; Prabakaran, D.; Hariprasad, P. Molecular docking, dynamics simulation and pharmacokinetic studies of *Cyperus articulatus* essential oil metabolites as inhibitors of *Staphylococcus aureus*. *J. Biomol. Struct. Dyn.* **2023**, *41*, 9245-9255, doi:10.1080/07391102.2022.2145371.
29. Elbouzidi, A.; Taibi, M.; Laaraj, S.; Loukili, E.; Haddou, M.; El Hachlafi, N.; Mrabti, H.N.; Baraich, A.; Bellaouchi, R.; Asehraou, A.; et al. Chemical profiling of volatile compounds of the essential oil of grey-leaved rockrose (*Cistus albidus* L.) and its antioxidant, anti-inflammatory, antibacterial, antifungal, and anticancer activity in vitro and in silico. *Front. chem.* **2024**, *12*, doi:10.3389/fchem.2024.1334028.
30. Sakuntala, P.; Pradyutha, A.C.; Selvaraju, R. Analysis of minerals and phytochemicals in the stems and seeds of *Ocimum Kilimandcharicum* plant using

spectroscopic methods and screening of antibacterial activity. *Chem. Pap.* **2024**, *78*, 7927-7931, doi:10.1007/s11696-024-03644-7.

31. Cuervo, L.; Alvarez-García, S.; Salas, J.A.; Méndez, C.; Olano, C.; Malmierca, M.G. The Volatile Organic Compounds of *Streptomyces* spp.: An In-Depth Analysis of Their Antifungal Properties. *Microorganisms* **2023**, *11*, doi:10.3390/microorganisms11071820.
32. Mahendran, G.; Vimolmangkang, S. Chemical compositions, antioxidant, antimicrobial, and mosquito larvicidal activity of *Ocimum americanum* L. and *Ocimum basilicum* L. leaf essential oils. *BMC Complementary Med. Ther.* **2023**, *23*, doi:10.1186/s12906-023-04214-2.
33. Shiromi, P.; Hewawasam, R.P.; Jayalal, R.G.U.; Rathnayake, H.; Wijayaratne, W.; Wanniarachchi, D. Chemical Composition and Antimicrobial Activity of Two Sri *Lankan Lichens, Parmotrema rampoddense*, and *Parmotrema tinctorum* against Methicillin-Sensitive and Methicillin-Resistant *Staphylococcus aureus*. *Evidence-Based Complement. Altern. Med.* **2021**, *2021*, doi:10.1155/2021/9985325.
34. Zanello, P.R.; Koishi, A.C.; Rezende, C.D.; Oliveira, L.A.; Pereira, A.A.; de Almeida, M.V.; dos Santos, C.N.D.; Bordignon, J. Quinic acid derivatives inhibit dengue virus replication in vitro. *Virol. J.* **2015**, *12*, doi:10.1186/s12985-015-0443-9.
35. Xu, L.; Zhou, Y.L.; Xu, J.W.; Xu, X.Z.; Lu, G.J.; Lv, Q.H.; Wei, L.J.; Deng, X.M.; Shen, X.; Feng, H.H.; et al. Anti-inflammatory, antioxidant and anti-virulence roles of atractylodin in attenuating *Listeria monocytogenes* infection.Front. Immunol. **2022**, *13*, doi:10.3389/fimmu.2022.977051.
36. Madia, V.N.; De Angelis, M.; De Vita, D.; Messore, A.; De Leo, A.; Ialongo, D.; Tudino, V.; Saccoliti, F.; De Chiara, G.; Garzoli, S.; et al. Investigation of *Commiphora myrrha* (Nees) Engl. Oil and Its Main Components for Antiviral Activity. *Pharmaceuticals* **2021**, *14*, doi:10.3390/ph14030243.
37. Qiu, B.; Wei, F.; Su, J.; Hao, W.; Zhou, J.; Zhao, J.; Wang, Y.; Qu, Z. The Effects of β-Pinene, a Pine Needle Oil Monoterpene, on Adenovirus Type 3. *Bull. Exp. Biol. Med.* **2022**, *172*, 345-351, doi:10.1007/s10517-022-05390-w.
38. Han, J.C.; Zhu, X.Y.; Gao, Z.H.; Xiao, Y.; Zhang, J.X.; Wang, P.; Fang, J.B.; Li, Y.Q.; Zhu, Y.L.; Li, Y.; et al. Antiviral effects of Atractyloside A on the influenza B virus (*Victoria strain*) infection. *Frontiers in Microbiology* **2023**, *13*, doi:10.3389/fmicb.2022.1067725.
39. Kambiré, D.A.; Kablan, A.C.L.; Yapi, T.A.; Vincenti, S.; Maury, J.; Baldovini, N.; Tomi, P.; Paoli, M.; Boti, J.B.; Tomi, F. Neuropeltis acuminata (P. Beauv.): Investigation of the Chemical Variability and In Vitro Anti-inflammatory Activity of the Leaf Essential Oil from the *Ivorian* Species. *Molecules* **2022**, *27*, doi:10.3390/molecules27123759.
40. Brito, L.S.; Batista, A.; Santos, F.A.; de Lima, R.P.; Ayala, A.P.; Canuto, K.M.; Silveira, E.R.; Pessoa, O.D.L. Anti-inflammatory kaurane diterpenoids of *Erythroxylum bezerrae*. *Fitoterapia* **2023**, *165*, doi:10.1016/j.fitote.2022.105424.

41. Bustos, A.D.; Liu, B.Y.; Jabba, S.; Morris, J.; Jordt, S.E. The anti-inflammatory effects of eucalyptol in a mouse model of LPS-induced pulmonary inflammation are mediated by TRPM8. *Eur. Respir. J.* **2016**, *48*, doi:10.1183/13993003.congress-2016.OA4541.
42. Ramalho, T.R.D.; de Oliveira, M.T.P.; Lima, A.L.D.; Bezerra-Santos, C.R.; Piuvezam, M.R. Gamma-Terpinene Modulates Acute Inflammatory Response in Mice. *Planta Med.* **2015**, *81*, 1248-1254, doi:10.1055/s-0035-1546169.
43. Liu, Y.H.; Tang, X.; Zhang, H.Z.; Zheng, L.Y.; Lai, P.; Guo, C.; Ma, J.F.; Chen, H.B.; Qiu, L.X. Terpinen-4-ol Improves Lipopolysaccharide-Induced Macrophage Inflammation by Regulating Glutamine Metabolism. *Foods* **2024**, *13*, doi:10.3390/foods13121842.
44. Ali, I.; Mu, Y.; Atif, M.; Hussain, H.; Li, J.P.; Li, D.D.; Shabbir, M.; Bankeu, J.J.K.; Cui, L.; Sajjad, S.; et al. Separation and anti-inflammatory evaluation of phytochemical constituents from *Pleurospermum candollei* (Apiaceae) by high-speed countercurrent chromatography with continuous sample load. *J. Sep. Sci.* **2021**, *44*, 2663-2673, doi:10.1002/jssc.202100155.
45. Hong, Y.H.; Kao, C.; Chang, C.C.; Chang, F.K.; Song, T.Y.; Houng, J.Y.; Wu, C.H. Anti-Inflammatory and T-Cell Immunomodulatory Effects of Banana Peel Extracts and Selected Bioactive Components in LPS-Challenged In Vitro and In Vivo Models. *Agriculture-Basel* **2023**, *13*, doi:10.3390/agriculture13020451.
46. Zhao, X.L.; Yu, L.; Zhang, S.D.; Ping, K.; Ni, H.Y.; Qin, X.Y.; Zhao, C.J.; Wang, W.; Efferth, T.; Fu, Y.J. Cryptochlorogenic acid attenuates LPS-induced inflammatory response and oxidative stress via upregulation of the Nrf2/HO-1 signaling pathway in RAW 264.7 macrophages. *Int. Immunopharmacol.* **2020**, *83*, doi:10.1016/j.intimp.2020.106436.
47. Escribano-Ferrer, E.; Regué, J.Q.; Garcia-Sala, X.; Montañés, A.B.; Lamuela-Raventos, R.M. In Vivo Anti-inflammatory and Antiallergic Activity of Pure Naringenin, Naringenin Chalcone, and Quercetin in Mice. *J. Nat. Prod.* **2019**, *82*, 177-182, doi:10.1021/acs.jnatprod.8b00366.
48. Xu, Y.; Cao, Y.P.; Tie, F.F.; Kong, X.Y.; Liu, Y.C.; Zhang, Y.R.; Guan, W.N.; Hu, N.; Wang, H.L.; Qin, X.C.; et al. Generation of suspension cell cultures with high syringin content and anti-inflammatory activity through overexpressing glycotransferase SiUGT72BZ2 in *Saussurea involucrata*. *Plant Biotechnol. J.* **2025**, *23*, 1713-1724, doi:10.1111/pbi.70001.
49. Mitic, V.D.; Ilic, M.D.; Jovanovic, V.P.S.; Djordjevic, A.S.; Markovic, M.S.; Stojanovic, G.S. Volatiles composition and antioxidant activity *Inula oculus-christi* L. from Serbia. *Nat. Prod. Res.* **2020**, *34*, 2698-2701, doi:10.1080/14786419.2018.1550767.
50. Jin, Z.X.; Mollica, F.; Huang, Y.Q.; Guernelli, S.; Baschieri, A.; Diquigiovanni, C.; Rizzardi, N.; Valenti, F.; Pincigher, L.; Bergamini, C.; et al. Pro-aromatic Natural Terpenes as Unusual "Slingshot" Antioxidants with Promising Ferroptosis Inhibition Activity. *Chem.—Eur. J.* **2024**, *30*, doi:10.1002/chem.202403320.

51. Guo, Y.F.; Baschieri, A.; Amorati, R.; Valgimigli, L. Synergic antioxidant activity of γ-terpinene with phenols and polyphenols enabled by hydroperoxyl radicals. *Food Chem.* **2021**, *345*, doi:10.1016/j.foodchem.2020.128468.
52. Fikadu, Y.; Yaya, E.E.; Chandravanshi, B.S. Chemical composition and antioxidant activities of the essential oils of *Lippia adoensis* Hochst ex. Walp and *Ocimum sanctum* Linn. *Bull. Chem. Soc. Ethiop.* **2022**, *36*, 95-108, doi:10.4314/bcse.v36i1.9.
53. Kumar, V.; Shriram, V.; Bhagat, R.; Khare, T.; Kapse, S.; Kadoo, N. Phytochemical profile, anti-oxidant, anti-inflammatory, and anti-proliferative activities of *Pogostemon deccanensis* essential oils. *3 Biotech* **2019**, *9*, doi:10.1007/s13205-018-1560-0.
54. Elsharkawy, E.R. GC-MS analysis of chemical composition, cytotoxicity and antioxidant activities of essential oils of *Senecio glaucus* under drastic conditions. *Main Group Chem.* **2022**, *21*, 233-241, doi:10.3233/mgc-210125.
55. Jannat, N.; Haque, M.A.; Sharna, J.F.; Fatema, K.; Rahman, M.; Sarker, M.S.; Uddin, M.N.; Uddin, A.; Patwary, M.A.M. Chemical profiling, antioxidant potential, and thrombolytic activity of extracts from entire plant of *Nelsonia canescens*: An in vitro and chemoinformatic investigation. *S. Afr. J. Bot.* **2025**, *186*, 497-509, doi:10.1016/j.sajb.2025.09.015.
56. Wang, P.; Zhao, Y.N.; Xu, R.Z.; Zhang, X.W.; Sun, Y.R.; Feng, Q.M.; Li, Z.H.; Xu, J.Y.; Xie, Z.S.; Zhang, Z.Q.; et al. Sesquiterpene Lactams and Lactones With Antioxidant Potentials From *Atractylodes macrocephala* Discovered by Molecular Networking Strategy. *Front. Nutr.* **2022**, *9*, doi:10.3389/fnut.2022.865257.
57. Song, G.Y.; Kim, S.M.; Back, S.; Yang, S.B.; Yang, Y.M. Atractylodes Lancea and Its Constituent, Atractylodin, Ameliorates Metabolic Dysfunction-Associated Steatotic Liver Disease via AMPK Activation. *Biomol. Ther.* **2024**, *32*, 778-792, doi:10.4062/biomolther.2024.083.
58. Akcakavak, G.; Kazak, F.; Deveci, M.Z.Y. Eucalyptol Protects against Cisplatin-Induced Liver Injury in Rats. *Biol. Bull.* **2023**, *50*, 987-994, doi:10.1134/s106235902360085x.
59. Tsai, M.C.; Wang, C.C.; Tsai, I.N.; Yu, M.H.; Yang, M.Y.; Lee, Y.J.; Chan, K.C.; Wang, C.J. Improving the Effects of Mulberry Leaves and Neochlorogenic Acid on Glucotoxicity-Induced Hepatic Steatosis in High Fat Diet Treated db/db Mice. *J. Agric. Food Chem.* **2024**, *72*, 6339-6346, doi:10.1021/acs.jafc.3c09033.
60. Li, Y.J.; Guo, M.Y.; Qin, W.Q.; Li, J.N.; Li, Y.F.; Zhang, F.K.; Xue, X.Y.; Li, S.; Qu, J.R.; Liu, R.P.; et al. Senkyunolide A ameliorates cholestatic liver fibrosis by controlling CLCC1-mediated endoplasmic reticulum Ca2+ release. *Acta Pharmacol. Sin.* **2025**, *46*, 3257-3272, doi:10.1038/s41401-025-01615-6.
61. Ji, D.; Wang, Q.H.; Zhao, Q.; Tong, H.J.; Yu, M.T.; Wang, M.; Lu, T.L.; Jiang, C.X. Co-delivery of miR-29b and germacrone based on cyclic RGD-modified nanoparticles for liver fibrosis therapy. *J. Nanobiotechnol.* **2020**, *18*, doi:10.1186/s12951-020-00645-y.

62. Xie, T.; Shu, Y.Q.; Huang, W.; Ren, A.B.; Lin, J.; Tan, Y.J.; Zhao, S.F.; Bu, J.G. β-eudesmol inhibits cell growth and enhances cell chemosensitivity of NPC through targeting FGF1/FGFR signaling. *Oral Oncol.* **2025**, *162*, doi:10.1016/j.oraloncology.2024.107168.
63. Zhong, W.X.; Zhang, Q. Atractylodin: An Alkyne Compound with Anticancer Potential. *Am. J. Chin. Med.* **2024**, *52*, 1729-1757, doi:10.1142/s0192415x24500551.
64. Zhao, P.; Wang, J.G.; Zou, C. Synergistic anticancer potential of biogenic nanoparticles and cryptomeridiol from *Sphaeranthus indicus*: targeting gastric cancer through apoptosis and cell cycle arrest. *Front. Pharmacol.* **2025**, *16*, doi:10.3389/fphar.2025.1565308.
65. Acikgul, F.C.; Duran, N.; Kutlu, T.; Ay, E.; Tek, E.; Bayraktar, S. The therapeutic potential and molecular mechanism of Alpha-pinene, Gamma-terpinene, and P-cymene against melanoma cells. *Heliyon* **2024**, *10*, doi:10.1016/j.heliyon.2024.e36223.
66. Zeng, Y.Y.; Pan, Y.B.; Zhang, B.; Luo, Y.B.; Tian, J.H.; Wang, Y.L.; Ju, X.D.; Wu, J.C.; Li, Y. Integrating Network Pharmacology, Molecular Docking, and Experimental Validation to Investigate the Mechanism of (-)-Guaiol Against Lung Adenocarcinoma. *Med. Sci. Monit.* **2022**, *28*, doi:10.12659/msm.937131.
67. Cao, Y.J.; Wu, Y.H.; Tu, H.B.; Gu, Z.; Yu, F.Z.; Huang, W.L.; Shen, L.P.; Wang, L.X.; Li, Y. (-)-Guaiol inhibit epithelial-mesenchymal transition in lung cancer via suppressing M2 macrophages mediated STAT3 signaling pathway. *Heliyon* **2023**, *9*, doi:10.1016/j.heliyon.2023.e19817.
68. Li, R.; Zhan, Y.; Ding, X.; Cui, J.J.; Han, Y.X.; Zhang, J.L.; Zhang, J.; Li, W.B.; Wang, L.L.; Jiang, J.D. Cancer Differentiation Inducer Chlorogenic Acid Suppresses PD-L1 Expression and Boosts Antitumor Immunity of PD-1 Antibody. *Int. J. Biol. Sci.* **2024**, *20*, 61-77, doi:10.7150/ijbs.83599.
69. Zhou, Y.X.; Wang, Q.; Wang, Q.; Zhou, Z.Y.; Peng, X.Y.; Qu, B.T.; Zhang, R.P. Self-assembled copper chlorogenic acid nanoparticles: Inducing pyroptosis and cuproptosis to activate antitumor immunity. *J. Controlled Release* **2025**, *384*, doi:10.1016/j.jconrel.2025.113941.
70. Bai, X.; Liu, Y.; Cao, Y.; Ma, Z.; Chen, Y.; Guo, S. Exploring the potential of cryptochlorogenic acid as a dietary adjuvant for multi-target combined lung cancer treatment. *Phytomedicine : international journal of phytotherapy and phytopharmacology* **2024**, *132*, 155907, doi:10.1016/j.phymed.2024.155907.
71. Chen, Y.E.; Liang, J.; Chen, S.X.; Lin, N.; Xu, S.X.; Miao, J.D.; Zhang, J.; Chen, C.; Yuan, X.; Xie, Z.Y.; et al. Discovery of vitexin as a novel VDR agonist that mitigates the transition from chronic intestinal inflammation to colorectal cancer. *Mol. Cancer* **2024**, *23*, doi:10.1186/s12943-024-02108-6.
72. Chen, Y.X.; Shen, J.Y.; Yuan, M.Y.; Li, H.Z.; Li, Y.Q.; Zheng, S.S.; Han, B.; Zhang, C.C.; Liu, S.L.; Sun, Q.M.; et al. Dehydrocostus lactone suppresses gastric cancer progression by targeting ACLY to inhibit fatty acid synthesis and autophagic flux. *J. Adv. Res.* **2025**, *67*, 331-348, doi:10.1016/j.jare.2024.01.028.

73. Zhang, Y.; Liu, Y.; Wang, J.; Jiang, Z.; Zhang, L.; Cui, Y.; Zhao, D.; Wang, Y. Atractylenolide II inhibits tumor-associated macrophages (TAMs)-induced lung cancer cell metastasis. *Immunopharmacol. Immunotoxicol.* **2022**, *44*, 227-237, doi:10.1080/08923973.2022.2037629.
74. Lin, Y.; Chen, K.; Zhu, M.; Song, W.; Wu, G.; Pan, A. Atractylenolide II regulates the proliferation, ferroptosis, and immune escape of hepatocellular carcinoma cells by inactivating the TRAF6/NF-κB pathway. *Naunyn-Schmiedeberg's Arch. Pharmacol.* **2024**, *397*, 7697-7710, doi:10.1007/s00210-024-03046-2.
75. Tian, S.; Ren, L.; Liu, C.; Wang, Z. Atractylenolide II Suppresses Glycolysis and Induces Apoptosis by Blocking the PADI3-ERK Signaling Pathway in Endometrial Cancer Cells. *Molecules* **2024**, *29*, doi:10.3390/molecules29050939.
76. Wu, Y.; Dai, S.; Zhang, Y.; Li, Z.; Zhu, B.; Liu, Q.; Wo, L.; Yu, Z.; Yuan, X.; Dou, X. Atractylenolide II combined with Interferon-γ synergistically ameliorates colorectal cancer progression in vivo and in vitro by blocking the NF-kB p65/PD-L1 pathway. *J. Cancer* **2024**, *15*, 4328-4344, doi:10.7150/jca.96647.
77. Li, Q.; Zeng, K.; Chen, Q.; Han, C.; Wang, X.; Li, B.; Miao, J.; Zheng, B.; Liu, J.; Yuan, X.; et al. Atractylenolide I inhibits angiogenesis and reverses sunitinib resistance in clear cell renal cell carcinoma through ATP6V0D2-mediated autophagic degradation of EPAS1/HIF2α. *Autophagy* **2025**, *21*, 619-638, doi:10.1080/15548627.2024.2421699.
78. Fan, M.; Gu, X.; Zhang, W.; Shen, Q.; Zhang, R.; Fang, Q.; Wang, Y.; Guo, X.; Zhang, X.; Liu, X. Atractylenolide I ameliorates cancer cachexia through inhibiting biogenesis of IL-6 and tumour-derived extracellular vesicles. *J. Cachexia, Sarcopenia Muscle* **2022**, *13*, 2724-2739, doi:10.1002/jcsm.13079.
79. Yu, R.; Yu, B.X.; Chen, J.F.; Lv, X.Y.; Yan, Z.J.; Cheng, Y.; Ma, Q. Anti-tumor effects of Atractylenolide I on bladder cancer cells. *J. Exp. Clin. Cancer Res.* **2016**, *35*, 40, doi:10.1186/s13046-016-0312-4.
80. Chen, W.; Li, P.; Liu, Y.; Yang, Y.; Ye, X.; Zhang, F.; Huang, H. Isoalantolactone induces apoptosis through ROS-mediated ER stress and inhibition of STAT3 in prostate cancer cells. *J. Exp. Clin. Cancer Res.* **2018**, *37*, 309, doi:10.1186/s13046-018-0987-9.
81. Song, G.Q.; Wu, P.; Dong, X.M.; Cheng, L.H.; Lu, H.Q.; Lin, Y.Y.; Tang, W.Y.; Xie, T.; Zhou, J.L. Elemene induces cell apoptosis via inhibiting glutathione synthesis in lung adenocarcinoma. *J. Ethnopharmacol.* **2023**, *311*, 116409, doi:10.1016/j.jep.2023.116409.
82. Aati, H.; Al-Qahtani, J.; Al-Taweel, A.; Farshori, N.N.; Orfali, R.; Perveen, S. Green synthesis of silver nanoparticles using Saudi *Xanthium strumarium* extract: anticancer potential against breast and lung cancer cells. *Front. Pharmacol.* **2025**, *16*, 1653711, doi:10.3389/fphar.2025.1653711.
83. Pant, J.; Singh, L.; Mittal, P.; Kumar, N. Valencene as a novel potential downregulator of THRB in NSCLC: network pharmacology, molecular docking, molecular dynamics simulation, ADMET analysis, and in vitro analysis. *Mol. Diversity* **2025**, *29*, 2543-2563, doi:10.1007/s11030-024-11008-2.

84. Li, Y.X.; Liu, J.; Li, F. Hinesol attenuates DSS-induced ulcerative colitis through the suppression of Src-mediated NF-κB and chemokine signaling pathway. *Cell Biochem. Biophys.* **2024**, *82*, 2747-2757, doi:10.1007/s12013-024-01391-w.
85. Qu, L.; Lin, X.; Liu, C.; Ke, C.; Zhou, Z.; Xu, K.; Cao, G.; Liu, Y. Atractylodin Attenuates Dextran Sulfate Sodium-Induced Colitis by Alleviating Gut Microbiota Dysbiosis and Inhibiting Inflammatory Response Through the MAPK Pathway. *Front. Pharmacol.* **2021**, *12*, 665376, doi:10.3389/fphar.2021.665376.
86. Heo, G.; Kim, Y.; Kim, E.L.; Park, S.; Rhee, S.H.; Jung, J.H.; Im, E. Atractylodin Ameliorates Colitis via PPARα Agonism. *Int. J. Mol. Sci.* **2023**, *24*, doi:10.3390/ijms24010802.
87. Yu, C.C.; Xiong, Y.J.; Chen, D.P.; Li, Y.L.; Xu, B.; Lin, Y.; Tang, Z.Y.; Jiang, C.L.; Wang, L. Ameliorative effects of atractylodin on intestinal inflammation and co-occurring dysmotility in both constipation and diarrhea prominent rats. *Korean J. Physiol. Pharmacol.* **2017**, *21*, 1-9, doi:10.4196/kjpp.2017.21.1.1.
88. Wang, X.J.; Zhang, G.H.; Bian, Z.W.; Chow, V.; Grimaldi, M.; Carivenc, C.; Sirounian, S.; Li, H.; Sladekova, L.; Mott, S.; et al. An abundant ginger compound furanodienone alleviates gut inflammation via the xenobiotic nuclear receptor PXR in mice. *Nat. Commun.* **2025**, *16*, doi:10.1038/s41467-025-56624-0.
89. Aslam, H.; Khan, A.U.; Qazi, N.G.; Ansari, S.F.; Minhas, A.M.; Alvi, A.M.; Ali, F. Plausible role of carveol in gastrointestinal disorders: Mechanistic insights via H(+)/K(+)-ATPase and voltage-gated calcium channel inhibition. *Biochem. Biophys. Res. Commun.* **2025**, *758*, 151615, doi:10.1016/j.bbrc.2025.151615.
90. Ni, J.; Zhang, L.; Feng, G.; Bao, W.; Wang, Y.; Huang, Y.; Chen, T.; Chen, J.; Cao, X.; You, K.; et al. Vanillic acid restores homeostasis of intestinal epithelium in colitis through inhibiting CA9/STIM1-mediated ferroptosis. *Pharmacol. Res.* **2024**, *202*, 107128, doi:10.1016/j.phrs.2024.107128.
91. Ghasemi-Dehnoo, M.; Lorigooini, Z.; Amini-Khoei, H.; Sabzevary-Ghahfarokhi, M.; Rafieian-Kopaei, M. Quinic acid ameliorates ulcerative colitis in rats, through the inhibition of two TLR4-NF-κB and NF-κB-INOS-NO signaling pathways. *Immun., Inflammation Dis.* **2023**, *11*, e926, doi:10.1002/iid3.926.
92. Zheng, C.; Zhong, Y.; Zhang, W.; Wang, Z.; Xiao, H.; Zhang, W.; Xie, J.; Peng, X.; Luo, J.; Xu, W. Chlorogenic Acid Ameliorates Post-Infectious Irritable Bowel Syndrome by Regulating Extracellular Vesicles of Gut Microbes. *Adv. Sci.* **2023**, *10*, e2302798, doi:10.1002/advs.202302798.
93. Shentu, C.; Mao, M.; Zhu, J.; Meng, Q.; Qian, H.; Li, X.; Zhang, S.; Ding, B.; Dai, S.; Yuan, X. Atractylenolide III Ameliorates Ulcerative Colitis By Targeting IL-17RA to Suppress Macrophage M1 Polarization. *J. Agric. Food Chem.* **2025**, *73*, 30761-30776, doi:10.1021/acs.jafc.5c09397.
94. Sun, M.; Zhan, H.; Long, X.; Alsayed, A.M.; Wang, Z.; Meng, F.; Wang, G.; Mao, J.; Liao, Z.; Chen, M. Dehydrocostus lactone alleviates irinotecan-induced

intestinal mucositis by blocking TLR4/MD2 complex formation. *Phytomedicine* **2024**, *128*, 155371, doi:10.1016/j.phymed.2024.155371.
95. Liang, S.; Chu, C.; Li, R.; Jiang, G.; Du, L. Costunolide and dehydrocostus lactone alleviate ulcerative colitis via regulating TLR4, NF-κB and PI3K expression. *Sci. Rep.* **2024**, *14*, 29777, doi:10.1038/s41598-024-77021-5.
96. Gao, Z.; Huang, P.; Yu, X.; Dong, R.; Lin, Y.; Tu, J.; Chen, L.; Cao, Y.; Liu, Y.; Yang, D.; et al. Ethanolic Extract of Atractylodis Rhizoma Ameliorates DSS-Induced Ulcerative Colitis via Improving Intestinal Barrier Function Through the Rho-Associated Kinase/Myosin Light Chain Pathway. *J. Med. Food* **2025**, *28*, 694-707, doi:10.1089/jmf.2024.k.0281.
97. Zhang, N.; Zhang, M.; Song, X.; Zhang, X.; Geng, Z.; Wang, L.; Ge, S.; Li, J.; Zuo, L.; Hu, J. [α-Cyperone Antagonizes Intestinal Mucosal Inflammatory Response Through Modulation of TLR4/NF-κB Signaling Pathway to Alleviate Crohn's Disease-Like Colitis in Mice]. *J. Sichuan Univ., Med. Sci. Ed.* **2024**, *55*, 1166-1174, doi:10.12182/20240960104.
98. Zhang, H.; Zou, Y.; Xue, Q.; Li, M.; Yang, H.; Cheng, H.; Gu, Y.; Shen, C.; Tian, Q.; Wang, S. Elemene oral emulsion attenuates colitis in mice by altering gut microbiome and regulating amino acids metabolism. *Microb. Pathog.* **2022**, *173*, 105821, doi:10.1016/j.micpath.2022.105821.
